# Transcriptome meta-analysis reveals the hair genetic rules in six animal breeds and genes associated with wool fineness

**DOI:** 10.3389/fgene.2024.1401369

**Published:** 2024-06-14

**Authors:** Xue Pu, Shengchao Ma, Bingru Zhao, Sen Tang, Qingwei Lu, Wenna Liu, Yaqian Wang, Yunlin Cen, Cuiling Wu, Xuefeng Fu

**Affiliations:** ^1^ Key Laboratory of Special Environments Biodiversity Application and Regulation in Xinjiang, Xinjiang Key Laboratory of Special Species Conservation and Regulatory Biology, College of Life Sciences, Xinjiang Normal University, Urumqi, Xinjiang, China; ^2^ Key Laboratory of Genetics Breeding and Reproduction of Xinjiang Wool-Sheep Cashmere-Goat (XJYS1105), Institute of Animal Science, Xinjiang Academy of Animal Sciences, Urumqi, Xinjiang, China; ^3^ College of Animal Science, Xinjiang Agricultural University, Urumqi, Xinjiang, China; ^4^ Jiangsu Livestock Embryo Engineering Laboratory, College of Animal Science and Technology, Nanjing Agricultural University, Nanjing, Jiangsu, China; ^5^ Key Laboratory of Herbivorous Livestock Genetics, Ministry of Agriculture, Institute of Biotechnology, Xinjiang Academy of Animal Sciences, Urumqi, Xinjiang, China

**Keywords:** Ordos fine wool sheep, six livestock breeds, wool fineness, meta-analysis, WGCNA

## Abstract

Wool plays an irreplaceable role in the lives of livestock and the textile industry. The variety of hair quality and shape leads to the diversity of its functions and applications, and the finer wool has a higher economic value. In this study, 10 coarse and 10 fine ordos fine wool sheep skin samples were collected for RNA-seq, and coarse and fine skin/hair follicle RNA-seq datasets of other five animal breeds were obtained from NCBI. Weighted gene co-expression network analysis showed that the common genes were clustered into eight modules. Similar gene expression patterns in sheep and rabbits with the same wool types, different gene expression patterns in animal species with different hair types, and brown modules were significantly correlated with species and breeds. GO and KEGG enrichment analyses showed that, most genes in the brown module associated with hair follicle development. Hence, gene expression patterns in skin tissues may determine hair morphology in animal. The analysis of differentially expressed genes revealed that 32 highly expressed candidate genes associated with the wool fineness of Ordos fine wool sheep. Among them, *KAZALD1* (grey module), *MYOC* (brown module), *C1QTNF6* (brown module), *FOS* (tan module), *ITGAM, MX2, MX1,* and *IFI6* genes have been reported to be involved in the regulation of the hair follicle cycle or hair loss. Additionally, 12 genes, including *KAZALD1, MYOC, C1QTNF6,* and *FOS,* are differentially expressed across various animal breeds and species. The above results suggest that different sheep breeds share a similar molecular regulatory basis of wool fineness. Finally, the study provides a theoretical reference for molecular breeding of sheep breeds as well as for the investigation of the origin and evolution of animal hair.

## 1 Introduction

Hair is a distinctive physical characteristic of vertebrates, shaped by long-term natural selection and human influence, which has further resulted in the development of diverse phenotypes, including color, form, and composition, across species and breeds, enhancing their adaptability, fertility, and vitality (Leite et al., 2020). In vertebrates, mammalian hair is typically characterized by its fine and silky texture, and certain livestock species exhibit softer, more elastic, and glossy hair fibers with exceptional thermal insulation and moisture-wicking properties (Mohan et al., 2014). Notably, sheep wool represents the most representative example of such characteristics, while cashmere from cashmere goats (*Capra hircus*) (Meilin et al., 2020) and rabbit wool from rabbits (*Oryctolagus cuniculus*) (MENGÜÇ, 2016) share similar attributes. These unique qualities make them ideal materials for crafting garments, quilts, blankets, decorative items, and industrial fabrics. However, the current pursuit of more comfortable, lightweight, and soft apparel and textiles necessitates higher overall quality standards for wool tops. Simultaneously, fiber fineness significantly impacts the overall quality of wool tops. Hence, enhancing the fineness of livestock hair poses a challenge for wool-type livestock species breeding such as sheep (Zenda et al., 2024). Numerous domestic and international studies have been conducted on hair fineness in livestock at present, primarily focusing on the influence of genetic factors (MENGÜÇ, 2016; Ma et al., 2017; Fu et al., 2020; Li et al., 2020; Meilin et al., 2020; Hui et al., 2021; Qin et al., 2022; Shi et al., 2022; Zhang and Wong, 2022; Costa-Silva et al., 2023; Huang et al., 2023; Ma et al., 2023; Zhang et al., 2023; Zenda et al., 2024). The identification of candidate genes influencing hair fineness provides a valuable reference for the exploration of molecular markers that directly regulate fineness phenotype variation. These findings have significant implications for improving hair fineness and implementing molecular breeding strategies in sheep and other animal with woolly coats.

Hair is an appendage of the skin, derived from the skin, and the lower part of the hair is composed of dermal papillae and hair follicles and penetrates the skin tissue. In previous hair fineness studies in livestock, RNA-seq was employed in skin or hair follicle tissues to compare gene expression differences between the coarse hair (C) and fine hair (F) groups, aiming to identify candidate genes associated with hair fineness. For instance, Ma et al. (2023) and Shi et al. (2022) conducted RNA-seq analysis on skin tissues obtained from Merino sheep and Hetian sheep with low follicle density (C) as well as high hair follicle density (F), respectively. The comparative analysis between F and Cerevealed the association of genes such as *COL1A1* and *LOC101116863* with wool fineness in Merino sheep, while genes like *TNF* and *MAP2K2* were found to be associated with hair follicle density in Hetian sheep. Similarly, Qin et al. (2022) and Fu et al. (2020) conducted analogous studies in Liaoning white cashmere goats and Tibetan northwest white cashmere goats, respectively. In their research, Fu et al. (2020) identified genes such as *KRT26* and *KRT28* associated with the fineness of cashmere in Tibetan northwest white cashmere goats. Additionally, Huang et al. (2023) discovered that genes like *LEF1* and *ITGB6* are linked to rabbit wool fineness through a comparative analysis of gene expression differences in hair follicle tissues from C and F Angora rabbits. Although various wool-type livestock species and breeds have been studied, there is a lack of systematic and comprehensive meta-analysis to elucidate the commonalities and discrepancies among their findings. Given that different breeds or species share a common ancestor, which results in them sharing some conserved genes. Conducting meta-analysis enables us to identify the common genes associated with hair fineness in diverse livestock species and breeds and enhances the accuracy and reproducibility of candidate gene identification. We consider that these candidate genes hold significant value, as they possess universality or broad-spectrum characteristics and potentially serve as key regulators in multiple minor genes or molecular signaling pathways governing hair fineness in livestock (Li et al., 2020). Simultaneously, these candidate genes offer a higher likelihood of identifying molecular markers (such as SNP, Indel, and QTL) associated with hair fineness (Huang et al., 2023), thereby presenting broader prospects for their application in the molecular breeding of wool-type livestock. In addition, various livestock species have both distinct and shared hair characteristics, with genetic factors governing the extent of similarity or divergence in hair morphology among breeds and species. Meta-analysis offers a preliminary explanation for these genetic factors and ultimately contributes novel insights into the study of adaptive evolution in animal hair.

The Ordos fine wool sheep is primarily cultivated in the hinterland of the Maowusu Desert and its surrounding areas in the southeast of Ordos City, serving as a significant local source of high-quality wool. Molecular breeding of a high-yielding and ultrafine population of Ordos fine wool sheep is currently the primary objective, with the identification of candidate genes associated with wool fineness in Ordos fine wool sheep through RNA-seq being crucial to this endeavor. Therefore, we focused on Ordos fine-wool sheep as the primary research subject in this study. Based on the population’s fiber diameter measurements, we carefully selected 20 ewes and divided them into two groups: the C group and the F group. Subsequently, skin tissues were collected from two groups for RNA-seq. Furthermore, we obtained skin or hair follicle RNA-seq datasets for Hetian sheep (Ma et al., 2023), Merino sheep (Shi et al., 2022), Northwest Tibetan white goats (Fu et al., 2020; Qin et al., 2022), Angora rabbits (Huang et al., 2023), and mallards from the SRA database. Based on the information provided by the data publisher, these datasets were classified into the C and F groups. Subsequently, employing weighted gene co-expression network analysis (WGCNA) (Zhang and Wong, 2022), differential gene expression (DEG) analysis (Costa-Silva et al., 2023), and GO and KEGG enrichment analyses, etc., we investigated the relationships and disparities in gene expression patterns between different breeds and species. Additionally, we revealed potential associations between gene expression patterns and animal hair morphological traits while identifying candidate genes associated with the wool fineness of Ordos fine wool sheep. Finally, the expression patterns of the candidate genes were verified by RT-qPCR in the C and F skin tissues of Ordos fine wool sheep. This study provides a theoretical reference for the molecular breeding of sheep breeds as well as research of the adaptive evolution of hair.

## 2 Materials and methods

### 2.1 Animals

The research included 45 female Ordos fine wool sheep, aged 12 months, randomly selected from Wuyin Surike Agriculture and Animal Husbandry Development Co., Ltd. All the ewes were kept under identical conditions and in good health.

### 2.2 Wool fiber diameter measurement and skin sample collection

The wool fiber diameter of 45 Ordos fine wool sheep was measured by collecting a 10 g sample of wool fiber from a point located 10 cm above the left center line of the shoulder blade for each individual. The average fiber diameter of all collected samples was determined using OFDA 2000 (2000 AFIS, United Kingdom) and recorded in a table for subsequent sorting purposes. Subsequently, individuals with average fiber diameters ranging from 15 to 17 μm and 18 to 21 μm were selected, with 10 individuals chosen from each range in sequential order to form the F and C groups, respectively. Finally, skin tissue samples measuring 2 cm^2^ were meticulously collected from the periphery of the scapula in a cohort of over 20 sheep and promptly cryopreserved in liquid nitrogen. Throughout the sampling procedure, strict adherence to ethical guidelines and relevant welfare policies was maintained.

### 2.3 Dataset acquisition for RNA-seq

The RNA-seq datasets used in this study were obtained from the Sequence Read Archive (SRA) of the National Center for Biotechnology Information (NCBI) (Table 1). Specifically, the datasets for Merino sheep (PRJNA889222), Hetian sheep (PRJNA754683), Tibetan northwest white cashmere goat (PRJNA643003), Angora rabbit (PRJNA916820), and Mallard duck (PRJNA679685) were downloaded. The Merino sheep dataset provides RNA-seq data from eight adult ewes’ skin tissues, with four samples belonging to the Fe group exhibiting fiber widths of 15 μm and the remaining four samples belonging to the C group exhibiting fiber diameters ranging from 20.1 to 21.5 μm. The Hetian sheep are a breed of semi-fine wool sheep, exhibiting both fine and coarse wool. Fine-hair-rich individuals possess higher hair follicle densities, while coarse-hair-rich individuals have lower hair follicle densities. The average fiber diameter for samples from the Fe group is 34.48 μm for coarse wool and 20.48 μm for fine wool, whereas the average fiber diameter for samples from the C group is 40.96 μm for coarse wool and 21.4 μm for fine wool. The dataset of Tibetan northwest white cashmere goats comprises RNA-seq data obtained from the skin tissues of eight adult ewes, with four samples representing F (mean fiber diameter of cashmere: 12.04 ± 0.03 µm) and the remaining four representing C (mean fiber diameter of cashmere: 14.88 ± 0.05 µm). Subsequently, we carefully selected three samples of dorsal skin tissue and three samples of head skin tissue from the mallard RNA-seq dataset. It is worth noting that while mallard head feathers possess exceptionally fine hair shafts, dorsal feathers exhibit coarser hair shafts, thus categorizing the dorsal samples as C and the head samples as F. A total of 34 RNA-seq datasets were acquired using SRA Toolkit 3.0.6. These 34 RNA-seq datasets underwent rigorous quality control measures to ensure their suitability for subsequent analysis.

**TABLE 1:** The details of the RNA-seq datasets that was obtained from the SRA.

Species	Accession number	Sample size	Coarse group (C)	Fine group (F)
Merino sheep	PRJNA889222	8	20.1∼21.5 μm	<15 μm
Hetian sheep	PRJNA754683	6	20.48∼21.4 μm	34.48∼40.96 μm
Tibetan northwest white cashmere goat	PRJNA643003	8	12.04 ± 0.03 µm	14.88 ± 0.05 µm
Angora rabbit	PRJNA916820	6	50∼60 µm	<20 µm
Mallard duck	PRJNA679685	6	Dorsal skin tissue (Coarse hairy shafts)	Head skin tissue (Fine hairy shafts)

### 2.4 Total RNA extraction, library construction, and RNA-seq

The Trizol-based RNA extraction method was employed to extract total RNA from 20 skin tissues. Subsequently, the NanoDrop 2000 (ND-2000, Thermo, United States) was utilized to measure the OD260/280 and OD260/230 of the extracted total RNA, while the Then, the Agilent 2100 (2100, Agilent, United States) was used to assess its integrity through RIN measurement. All 20 total RNA samples exhibited OD260/280 values within the range of 1.8–2.0, OD260/230 values exceeding 2.0, and RIN values ranging from 7.0 to 8.5. Thus rendering them suitable for library construction. Following the qualification of RNA samples for quality control, RNA-seq libraries were constructed for 20 samples using the method provided by Illumina as a reference. Subsequently, qPCR was conducted to quantify the effective concentration of each library (with an effective concentration >2 nm). After library qualification, sequencing of the 20 libraries was performed on the Illumina platform. Clean reads were obtained by eliminating poly-N regions, original reads containing adapters, and low-quality reads. The quality control results of the skin tissue RNA-seq data are presented in Supplementary Table S1.

### 2.5 Data pre-processing

The RNA-seq datasets of Ordos fine wool, Merino sheep, and Hetian sheep were mapped to the sheep reference genome (ARS-UI_Ramb_v2.0, GCF_016772045.1). Similarly, the Northwestern Tibetan white cashmere goat RNA-seq data were mapped to the goat reference genome (ARS1.2, GCF_001704415.2), while the Angora rabbit RNA-seq data were mapped to the rabbit reference genome (UM_NZW_1.0, GCF_009806435.1). Additionally, mallard RNA-seq data were mapped to the mallard reference genome (IASCAAS_PekingDuck_PBH1.5) using HISAT2 2.2.1 software (Kim et al., 2015). Notably, all samples exhibited mapping rates exceeding 91%. Subsequently, the output files of 60 RNA-seq data obtained from mapping were processed using SAMtools software (Li et al., 2009). For subsequent analysis, Htseq-count software (Anders et al., 2015) was employed to generate the read counts of all genes. Finally, we calculated the counts per million (CPM) values based on the read counts to assess gene expression levels, and utilized the following formula:

$$cpm=\frac{A}{mapped\;reads\times 1000000}$$

In the formula, A represents the read count of each gene. Subsequently, after generating cpm values for all genes, the cpm values of common genes across the six breeds were utilized for principal component analysis (PCA).

### 2.6 WGCNA

The read count values of common genes across six breeds were utilized as input data for weighted gene co-expression network analysis using WGCNA 1.72 software (Langfelder and Horvath, 2008), which was implemented in R 4.2.0 through RStudio. A clustering tree was constructed based on the correlations between the samples to identify and remove outliers. Subsequently, pairwise Pearson correlation matrices were generated for all genes. The pickSoftThreshold function was employed to determine an appropriate soft threshold (β) and establish neighbor-joining relationships among genes, thereby implementing a scale-free network. The gene modules were identified by calculating the correlation using the Topological Overlap Measure (TOM) (Zhang and Horvath, 2005). Subsequently, a hierarchical clustering tree was constructed based on the dissimilarity (1-TOM), with a minimum module size of 30 (Langfelder et al., 2008). The genes exhibiting similar expression patterns were grouped into the same module using modular gene (ME) (Ma et al., 2017). Modules displaying more than 75% similarity were merged by employing the default 0.25 tree height shear: MEDISSTHRES=0.25 in WGCNA (Chen et al., 2019; Guo et al., 2020).

The identification of key modules associated with fineness, species, and breeds involved the simultaneous calculation of gene significance (GS) and module membership (MM) values (Fuller et al., 2007), which was employed to characterize the correlation between ME and fineness, species, and breeds. MM represents the correlation between gene expression profiles and each ME (Forssmann et al., 1997); a higher correlation between GS and MM indicates greater importance of the module associated with the trait (Zhang and Horvath, 2005). The statistical significance of the correlation between modules and fineness, species, or breeds was assessed using Pearson correlation analysis (Zhang et al., 2018). Modules exhibiting |cor| > 0.50 (indicating a moderate relationship) and *p <* 0.05 were considered highly significant (Love et al., 2014).

### 2.7 Analysis of differentially expressed genes (DEGs)

The DEGs analysis was conducted using the DESeq 2.0 (Abe and Tanaka, 2017) package in R to identify DEGs between the C and F groups within each breed. For Ordos fine wool sheep (C) vs. Ordos fine wool sheep (F), Merino sheep (C) vs. Merino sheep (F), Tibetan northwest white cashmere goat (C) vs. Tibetan northwest white cashmere goat (F), and Mallard (C) vs. Mallard (F), and the Benjamini-Hochberg correction controls the error discovery rate. The DEG thresholds were set as fold change >0.75 or < −0.75 and *p <* 0.05, while for Hetian sheep (C) vs. Hetian sheep (F) and Angora rabbit (C) vs. Angora rabbit (F), the DEG thresholds were fold change >1 or < −1 with *p <* 0.05. The Venn diagram analyses of DEGs were performed using jvenn online tool (https://jvenn.toulouse.inrae.fr/app/example.html).

### 2.8 Gene function enrichment and protein interaction network analysis

The GO and KEGG enrichment analysis of genes in the module and DEGs was conducted using the DAVID online tool (https://david.ncifcrf.gov/), where pathways with a significance level of *p <* 0.05 were considered statistically significant. Additionally, protein-protein interaction network analysis of the DEGs was performed utilizing the STRING online database (https://string-db.org/)

### 2.9 RT-qPCR

The relative expression levels of 32 candidate DEGs were assessed by RT-qPCR to validate the RNA-seq results. The cDNA synthesis was performed using the FastKing RT Kit (with gDNase) kit (KR116 TIANGEN, Beijing, China). Primers were designed using primer premier 5.0 software. *GAPDH* served as a reference gene, and the primer information is provided in Supplementary Table S4. The qPCR was conducted on a CFX96TM Real-Time System instrument (CFX96, BIO-RAD, Hercules, CA, United States) using the Talent qRCR PreMix (SYBR Green) kit (FP209 TIANGEN, Beijing, China). The amplification process details are presented in Supplementary Table S5.

## 3 Results

### 3.1 PCA analysis

**FIGURE 1:**
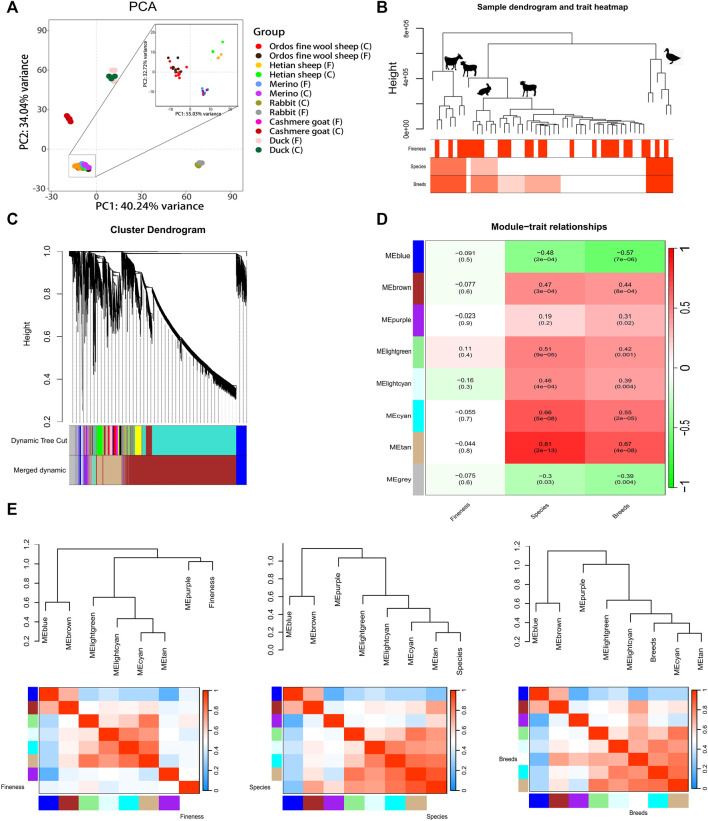
Analysis of gene expression patterns in 54 RNA-seq data of six breeds of livestocks. **(A)** PCA analysis of gene expression in 54 RNA-seq data of six breeds of livestocks; **(B)** Clustering dendrogram of samples based on their Euclidean distance; **(C)** Clustering dendrogram of genes, with dissimilarity based on topological overlap, together with assignedmerged module colors and the original module colors; **(D)** Module-trait associations. Each row corresponds to a module eigengene, column to a trait. Each cellcontains the corresponding correlation and *p*-value. The table is color-coded by correlation according to the colorlegend; **(E)** Visualization of the eigengene network representing the relationships among the modules and the clinical trait weight. Panel shows a hierarchical clustering dendrogram of the eigengenes in which the dissimilarity of eigengenes EI, EJ is given by 1-cor (EI, EJ). The heatmap in panel shows the eigengene adjacency AIJ = [1 + cor (EI, EJ)]/2.

The PCA analysis of six breeds revealed that PC1 accounted for 34.04% of the variance, while PC2 accounted for 40.24%, resulting in a cumulative contribution of 74.28%. The sample points representing sheep, goats, rabbits, and mallard were distributed across distinct regions on the plot (Figure 1A). Notably, the sample points corresponding to Ordos fine wool sheep, Merino sheep, and Hetian sheep formed a tight cluster (Figure 1A). Furthermore, within these three sheep breeds, it was evident that the sample points representing Ordos fine wool sheep, Merino sheep, and Hetian sheep could be clearly discriminated from each other (Figure 1A). However, except for Hetian sheep breed samples, there was no clear distinction between C and F group samples in other breeds (Figure 1A). These findings suggest that species-specific expression patterns differ significantly among the six breeds examined; however, the differences observed between the three sheep breeds are relatively small compared to those observed across all six species. In conclusion, RNA-seq data from these six breeds exhibits unique characteristics.

### 3.2 Expression pattern analysis of six breeds

The clustering tree (Figure 1B; Supplementary Figure S2) generated by WGCNA analysis revealed the distinct separation of the Tibetan northwest white cashmere goat from the other three species. The Angora rabbit and the three sheep breeds were further grouped together within a single branch, occupying the fourth level of the hierarchical clustering tree. In contrast, Ordos fine wool sheep and Merino sheep were found in close proximity on a lower-level branch. These findings indicate that gene expression patterns are more similar between the Angora rabbit and three sheep breeds compared to other species, with Hetian sheep showing closer similarity to the Angora rabbit and Ordos fine wool sheep exhibiting greater resemblance to Merino sheep. Furthermore, it was observed that certain C and F RNA-seq data points could not be clearly distinguished within their respective species branches.

The network structure of the co-expression network was constructed by setting a soft threshold (β value), with β set to 20, to ensure high connectivity between genes in the network (Supplementary Figure S1). Subsequently, gene clustering resulted in eight distinct modules (Figure 1C). A correlation analysis between these modules and phenotypes revealed that lightgreen and lightcyan modules exhibited the highest correlations with fineness, with correlation coefficients of 0.11 and −0.16, respectively (*p* > 0.05) (Figure 1D and Supplementary Figure S3). Furthermore, the blue, brown, light green, light cyan, cyan, and tan modules exhibited a highly significant correlation with species or breeds (*p <* 0.01). The grey module demonstrated a significant correlation with species (*p <* 0.05), while the purple module displayed a significant correlation with breeds (*p <* 0.05). These findings suggest that gene expression patterns within modules primarily reflect the specificity of species. Correlation analysis between modules revealed that the blue module was significantly correlated with the brown module; likewise, there were significant correlations observed among the light green and tan modules as well as among the tan, cyan, and light cyan modules (Figure 1E and Supplementary Figure S4).

Subsequently, GO and KEGG enrichment analyses were conducted to investigate the gene modules (Supplementary Tables S2, S3). The outcomes revealed that genes in the brown module exhibited enrichment in hair follicle development (GO: 0001942), canonical Wnt signaling pathway (GO: 0060070), positive regulation of canonical Wnt signaling pathway (GO: 0090263), positive regulation of I-kappaB kinase/NF-kappaB signaling (GO: 0043123), negative regulation of I-kappaB kinase/NF-kappaB signaling (GO: 0043124), Hedgehog signaling pathway (oas04340), ECM-receptor interaction (oas04512), NF-kappa B signaling pathway (oas04064), EGFR tyrosine kinase inhibitor resistance (oas01521), TGF-beta signaling pathway (oas04350), AMPK signaling pathway (oas04152), PI3K-Akt signaling pathway (oas04151), MAPK signaling pathway (oas04010), Wnt signaling pathway (oas04310) and other pathways; genes in the blue module were enriched for BMP receptor binding (GO: 0070700) and cell cycle (GO: 0007049) pathways; while genes in the purple module showed enrichment primarily in mTOR signaling pathway (oas04150) and other pathways. These findings suggest that the expression pattern of genes specifically related to hair morphology is observed within skin tissues and hair follicles.

### 3.3 DEGs analysis based on fineness phenotypic grouping

**FIGURE 2:**
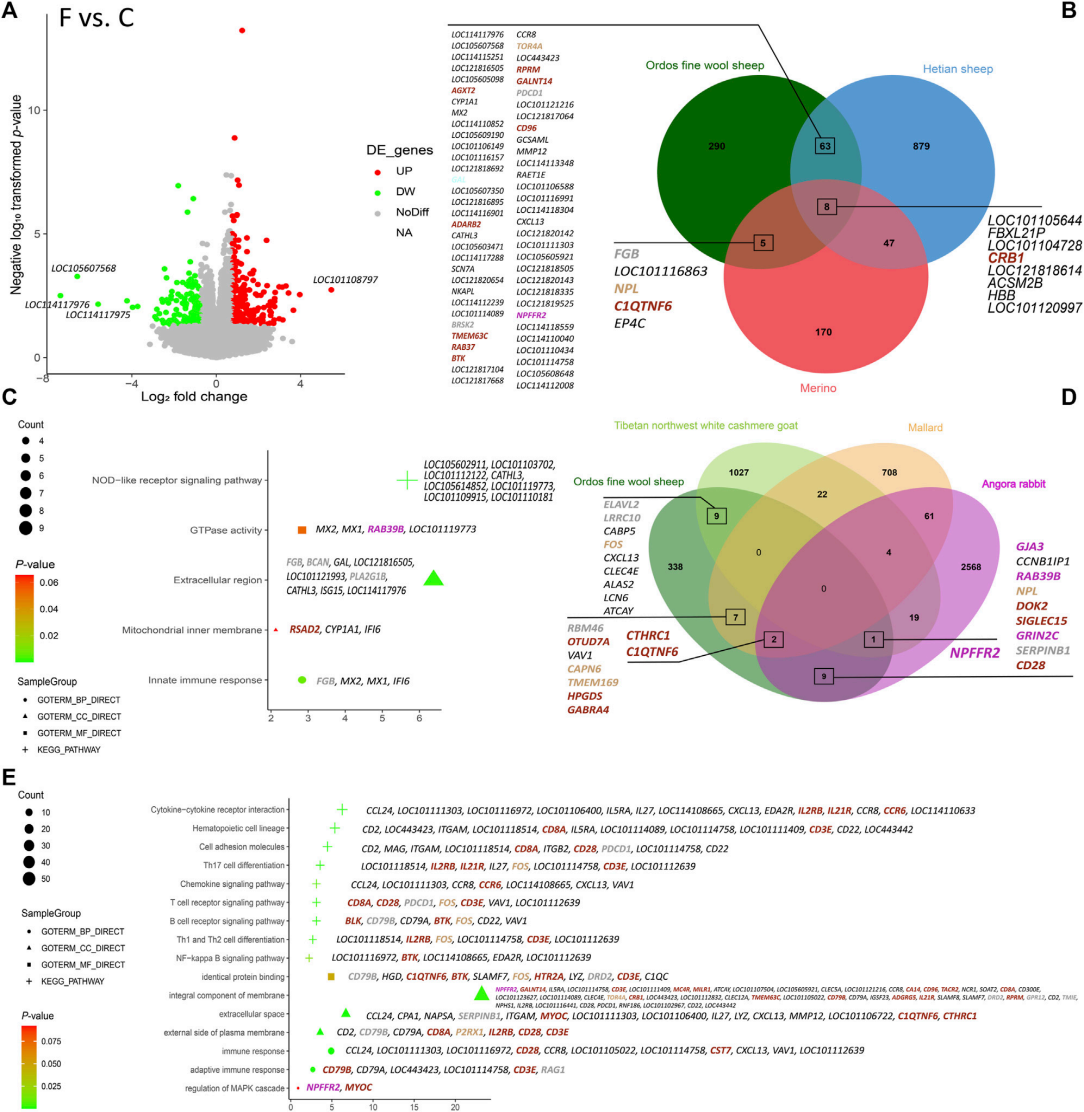
Results of the analysis of DEGs in the six varieties. **(A)** Volcano plot of DEGs in Ordos fine wool sheep (C) vs. Ordos fine wool sheep (F); **(B)** Venn diagram of Ordos fine wool sheep (C) vs. Ordos fine wool sheep (F), Hetian sheep (C) vs. Hetian sheep (F), and Merino sheep (C) vs. Merino sheep (F); **(C)** Bubble chart of functional enrichment analysis of downregulated expressed genes in the Ordos fine wool sheep (C) vs. Ordos fine wool sheep (F) group; **(D)** Venn diagram of Ordos fine wool sheep, Tibetan northwest white cashmere goat, Angora rabbit, and mallard; **(E)** Bubble chart of functional enrichment analysis of upregulated genes expressed in the Ordos fine wool sheep (C) vs. Ordos fine wool sheep (F) group.

Based on the thresholds of DEGs, a total of 366 DEGs were identified in the Ordos fine wool sheep (C) vs. Ordos fine wool sheep (F) group. Among these, 141 genes were downregulated and 225 genes were upregulated (Figure 2A). GO and KEGG enrichment analyses revealed that downregulated genes were primarily associated with pathways such as the NOD-like receptor signaling pathway (oas04621) and extracellular region (GO: 0005576). On the other hand, upregulated genes were mainly involved in processes related to immune response (GO: 0006955), integral component of membrane (GO: 0016021), extracellular space (GO: 0005615), external side of the plasma membrane (GO: 0009897), identical protein binding (GO: 0042802). Additionally, they also showed significant associations with pathways including hematopoietic cell lineage (oas04640), cytokine-cytokine receptor interaction (oas04060), cell adhesion molecules (oas04514), and the NF-kappa B signaling pathway (oas04064) (Figures 2C, E).

Venn diagram analysis (Figure 2B) revealed that the comparison groups of Ordos fine wool sheep (C) vs. Ordos fine wool sheep (F) and Hetian sheep (C) vs. Hetian sheep (F) shared a total of 71 DEGs. Similarly, the comparison groups of Ordos fine wool sheep (C) vs. Ordos fine wool sheep (F) and Merino sheep (C) vs. Merino sheep (F) had 13 common DEGs, while the three breed comparison groups shared eight DEGs in common. Additionally, when comparing Ordos fine wool sheep with Angora rabbit, mallard, and Tibetan northwest white cashmere goat individually, there were 12, 9, and 10 common DEGs, respectively, observed at their intersections with Ordos fine wool sheep. Furthermore, there was one common DEG found at the intersection set of Angora rabbit, Ordos fine wool sheep, and Tibetan northwest white cashmere goat; whereas two common DEGs were identified at the intersection set of Ordos fine wool sheep, Angora rabbit, and mallard.

In summary, we identified 32 significant and highly expressed DEGs based on the basemean values obtained from DEG analysis and the intersection set of different comparison groups. These candidate genes (*CCL24, ITGAM, LOC101121216, LOC114110633, LOC114113348, HBB, KAZALD1, ITGB2, BRSK2, RSAD2, C1QC, LOC101105154, LOC101102194, MX2, LOC101117112, LOC114118559, LOC105614852, SERPINB1, LOC114112239, LOC114110058, LOC101118514, LOC101116863, FOS, C1QTNF6, MYOC, LOC105602911, LOC101112122, IFI6, MX1, LOC101117229, LOC114118432,* and *LOC114110852*) were found to potentially influence wool fineness in Ordos fine wool sheep, as their base mean values were all greater than 100. Among these DEGs, the *KAZALD1* and *FOS* genes were located in the tan module, the *ITGB2, C1QTNF6,* and *MYOC* genes were located in the brown module, and the *BRSK2* and *SERPINB1* genes were located in the grey module. Furthermore, *FOS, BRSK2,* and *C1QTNF6* are common DEGs between Ordos fine wool sheep and other species.

**FIGURE 3:**
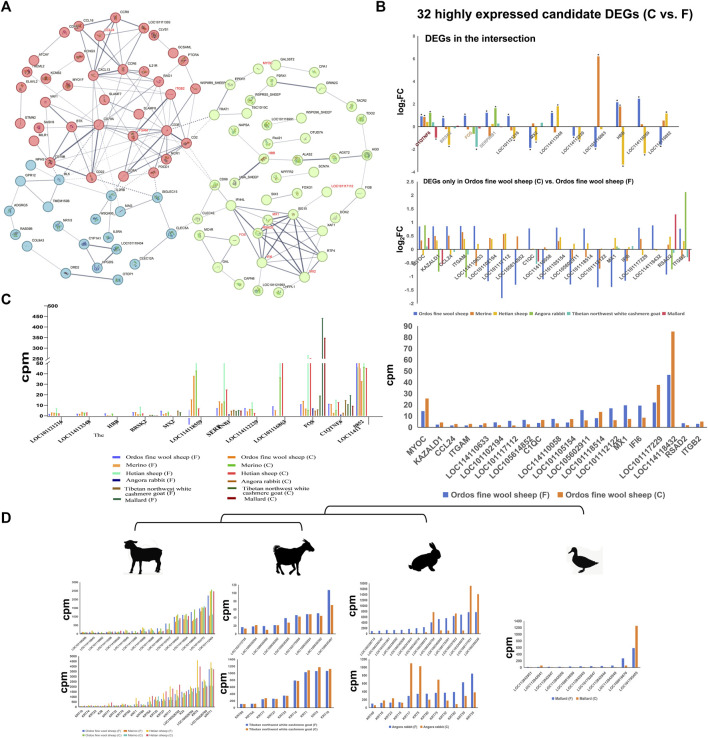
Expression patterns of DEGs, *KRTs* and *KRTAPs* and protein interaction network analysis of differential genes. **(A)** Protein interaction network analysis of differential genes in the Ordos fine wool sheep (C) vs. Ordos fine wool sheep (F) group; **(B)** |log_2_ fold change| values of 32 candidate DEGs in different comparison groups; **(C)** Expression patterns of 32 DEGs in different breeds; **(D)** Expression patterns of *KRTs* and *KRTAPs* in six breeds.

Furthermore, the protein interaction network revealed that several of these potential key genes (*ITGB2, MX1, RSAD2, IFI6*, and *MX2* genes) occupied pivotal positions in the network topology (Figure 3A). *C1QTNF6* gene exhibited upregulation across all three sheep breeds as well as in Angora rabbit and Tibetan northwest white cashmere goat. Conversely, it was downregulated in mallard (Figure 3B). Additionally, the |log_2_ fold change| values for *LOC114118559, HBB, SERPINB1*, and *LOC114113348* genes were significantly higher in the Ordos fine wool sheep (C) vs. Ordos fine wool sheep (F) group. Moreover, *SERPINB6, FOS, C1QTNF, LOC114110852* and *LOC114118432* genes exhibited elevated expression levels (Figure 3C).

### 3.4 The expression of keratin/keratin-associated gene family members in six breeds

The composition of hair, wool, and cashmere consists of keratin proteins (KRTs) and keratin-associated proteins (KRTAPs), with considerable variation in the number and types of KRT/KRTAP gene family members across different breeds. In this study, we specifically investigated the expression patterns of *KRT* and *KRTAP* genes in four species (Figure 3D). Our results revealed a striking similarity in the expression patterns of *KRT* and *KRTAP* genes among the three sheep breeds. Notably, genes such as *KRT35*, *KRT27*, *KRT17*, *LOC100526782* (keratin 33B), *K33*, *LOC100526784* (keratin 81), *KRT5*, *LOC100526780* (keratin 83), *KRT71*, *LOC101104027* (keratin-associated protein 7-1-like), *LOC114118004* (keratin-associated protein 8-1), *LOC101106046* (keratin-associated protein 13-1-like), *LOC101103772* (keratin-associated protein 11-1), and *LOC101112404* (keratin-associated protein 3-2) were consistently expressed at high levels across all three sheep breeds. While goats exhibited a greater diversity of both *KRT* and *KRATP* genes compared to sheep breeds, the number of highly expressed genes was significantly lower in goats. Additionally, it is worth noting that the *KTR5* gene was also found to be highly expressed in goats. Among the four species studied, Mallard displayed the lowest number of keratin genes, with only one gene showing high expression levels.

### 3.5 Verification of the sequencing data by RT-qPCR

**FIGURE 4:**
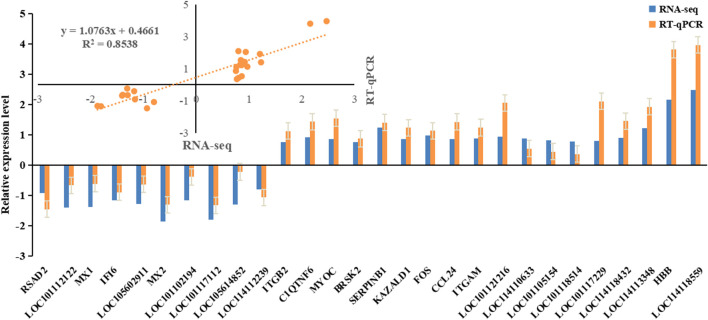
The expression level of candidate genes was detected by RT-qPCR.

In order to verify the reliability of the sequencing results, some candidate genes were selected for RT-qPCR (Figure 4). The results were in concordance with the RNA-seq data, indicating the sequencing results were reliable.

## 4 Discussion

Fineness is an important factor affecting the economic value of livestock hair. Accurate identification of candidate genes crucial for sheep wool fineness is essential for genetic marker development and molecular genetic advancement in hair fineness. Despite some progress in screening candidate genes across different wool-type animals, overall progress has been sluggish. This study aims to investigate the correlation between gene expression patterns and hair characteristics in animal species, specifically focusing on identifying key candidate genes associated with wool fineness in Ordos fine wool sheep through meta-analysis of RNA-seq data from six breeds. Ultimately, our findings provide theoretical references for further research on molecular regulatory mechanisms underlying hair morphology, the adaptive evolution of animal hair, and molecular breeding strategies for improving wool quality in sheep.

### 4.1 Gene expression patterns associated with the hair morphological characteristics of animals

Merino sheep and Ordos fine wool sheep are considered homogeneous wool breeds, while Hetian sheep is classified as a heterogeneous wool breed. Based on gene expression pattern clustering, the skin gene expression patterns of Ordos fine wool sheep were found to be similar to those of Merino sheep but distinct from those of Hetian sheep. This suggests that gene expression patterns may play a role in determining hair morphology characteristics in animal species. Angora rabbits, being a heterogeneous wool species, exhibit similarities with sheep in terms of hair type, and their gene expression patterns show comparability to those of sheep, particularly Hetian sheep, based on the clustering tree analysis. On the other hand, Tibetan northwest white cashmere goats and Mallards are both heterogeneous hair species; however, they possess unique hair characteristics that differentiate them from Angora rabbits and Hetian sheep. The Tibetan northwest white cashmere goat primarily produces cashmere without medulla structure, whereas mallard feathers have medulla structure along with branch and skeleton features. Expression pattern clustering further confirms the distinction between these two species’ gene expression patterns compared to those observed in sheep and Angora rabbits, supporting our hypothesis. Interestingly, we also discovered that Mallards exhibit closer similarity to both sheep and rabbits in terms of their gene expression pattern clustering tree analysis than Tibetan northwest white cashmere goats do. This intriguing finding can potentially be explained by the significant difference between cashmere (lacking medulla) and wool traits when compared to mallard feathers, which possess medulla structure; this could account for this interesting observation. In conclusion, each species’ unique hair morphological traits appear to be determined by specific patterns of gene expression within their skin tissue.

Subsequently, we noted that the brown, blue, tan, and purple modules were highly significantly correlated with species or breeds. Notably, several genes (*PIAS4, FZD3, TGFB2, FZD6, TNFRSF19, LRP4, INHBA, HOXC13, LDB2, ACVR1B, APCDD1, CELSR1, DKK1, EDAR, CD109, SOS1, LGR5,* and *LGR4)* within the brown module were associated with hair follicle development. Additionally, some other genes (*NFKB6, PRKCB, IKBKB, FGF2,* etc.) were enriched in signaling pathways regulating hair follicle development, including Hedgehog (Krieger et al., 2018), NF-kappa B (MOON et al., 2019), AMPK (Chen et al., 2020), PI3K-Akt (Öztürk et al., 2015), MAPK (Andl et al., 2002), and Wnt (Xin et al., 2023). Some genes within the blue module were found to be associated with BMP receptor binding, such as *CDH3*, and *SCUBE3*. This suggests that the expression patterns of those within the brown module play a crucial regulatory role in hair morphogenesis among these genes in common. However, the cluster analysis of expression patterns and correlation analysis between modules and fineness revealed a weak relationship between these common gene expressions and fineness. Therefore, further investigation is required to determine which specific genes influence wool fineness. In the WGCNA, we only focused on the genes shared by the four species, and the influence of sheep-specific genes on wool fineness also needs further discussion.

### 4.2 The influence of DEGs on wool fineness

In DEG analysis, we identified 366 DEGs in Ordos fine wool sheep. The intersection of DEGs between Ordos fine wool sheep and Hetian sheep is greater than that between Ordos fine wool sheep and Merino sheep. It is evident that the phenotypic differences between the F and C groups were more pronounced in Hetian sheep, followed by Ordos fine wool sheep, while the differences were least significant in Merino sheep. This pattern was also reflected in the principal component analysis (PCA) results at the gene expression level. Consequently, a total of 997 DEGs were identified in Hetian sheep, whereas 230 DEGs were detected in Merino sheep. Furthermore, there was a greater intersection of DEGs between Ordos fine wool sheep and Hetian sheep compared to other species. Interestingly, the intersection between Ordos fine wool sheep and other species was relatively smaller than that observed with Hetian or Merino breeds. These findings suggest that, while there may be some shared mechanisms involved in regulating fineness among different species, it appears that each breed of sheep possesses its own unique mechanism for this trait.

Subsequently, we further discovered that 32 DEGs exhibited high expression levels in the skin tissues of Ordos fine wool sheep. In comparison to the low-expressed genes, these highly expressed DEGs displayed more pronounced activities, suggesting their potentially crucial roles in skin tissue functions. Among the 32 DEGs, *RSAD2*, *ITGB2, C1QTNF6,* and *MYOC* were identified within the brown module. This observation implies their potential involvement in regulating wool fineness and morphology. Previous studies have reported that the *RSAD2* gene (Chen et al., 2012; Li et al., 2016) is associated with immunity as well as cell proliferation and differentiation processes; the *BRSK2* gene plays a significant role in neuronal polarization (Silverman et al., 2001); the *SERPINB1* protein acts as an inflammation regulator and is linked to cancer development (Son et al., 2015); and the *C1QC* gene exhibits similar functionality to the *SERPINB1* gene (Forssmann et al., 1997). Additionally, the *CCL24* gene serves as a chemokine, promoting immune cell transport and activation along with fibrotic activity (Wang et al., 2013). Notably, the *KAZALD1* gene encodes a member of the *IGFBP* gene superfamily, which may contribute to bone development and regeneration (Batch et al., 1996) and hair follicle cycle regulation (He et al., 2022). Henceforth, it can be inferred that the *KAZALD1* gene might play a comparable role in hair follicle cycle regulation.

In addition to the aforementioned genes, the *ITGB2* gene is situated within the Hippo signaling pathway, which has been associated with hair follicle development in sheep and goats by He et al. (Li et al., 2022) and Wang et al. (2021), respectively. Furthermore, the *MYOC* gene is enriched in the bone development (GO:0060348) and regulation of the MAPK cascade (GO:0043408) pathways. The MAPK signaling pathway plays a crucial role in regulating hair follicle development. Hence, it is plausible that the *ITGB2* and *MYOC* genes may also contribute to this process. Wang et al. (Fisher et al., 1991) and Xin et al. (2023) have reported the *C1QTNF6* and *MX2* genes as being involved in hair follicle cycle regulation, respectively. Additionally, Fisher et al. (Premanand and Reena Rajkumari, 2023) discovered that *FOS* protein expression patterns during keratinocyte formation are indicative of its pivotal role in transforming active epithelial cells into keratinocytes, an important marker for hair follicles, inner root sheaths, and differentiated hair follicles. Moreover, Premanand et al. (Tazi-Ahnini et al., 2000) found an association between the *ITGAM* gene and androgenetic alopecia, while Tazi-Ahnini et al. (Harel et al., 2015) identified polymorphisms in the *MX1* gene linked to hair loss. The *IFI6* gene, regulated by the JAK/STAT signaling pathway, is a type I interferon-stimulated gene that plays a crucial role in hair follicle growth. Previous research conducted by Matsunaga et al. (2020) has demonstrated a significant correlation between the expression levels of IFI6 and the absence of hair cells. These findings substantiate our conclusion regarding these candidate genes’ significance for wool fineness. However, further investigation is required to elucidate their specific roles, as some *LOC* genes remain uncharacterized.

Finally, we observed a consistent relative upward or downward trend in the average expression level of 32 DEGs between the two groups (C and F). This finding was further validated by qPCR analysis, demonstrating concordant expression patterns for most genes with only a few exceptions. These results provide evidence for a significant correlation, either positive or negative, between candidate gene expression and phenotype, thereby supporting their potential influence on wool fineness.

## 5 Conclusion

Gene expression patterns may determine the morphological characteristics of domestic animal hair. Meanwhile, the expression pattern of genes in the brown module may play a crucial regulatory role in hair morphogenesis. The fineness regulation mechanisms of various breeds share some similarities, among which 32 candidate genes (*RSAD2, ITGB2, C1QTNF6, MYOC, BRSK2, SERPINB1, KAZALD1, FOS, CCL24, ITGAM, LOC101121216, LOC114110633, LOC101102194, MX2, LOC114113348, LOC101117112, LOC105614852, C1QC, LOC114110058, LOC114112239, LOC101116863, LOC101105154, HBB, LOC105602911, LOC101118514, LOC101112122, MX1, IFI6, LOC114118559, LOC101117229, LOC114110852,* and *LOC114118432*) associated with wool fineness of Ordos fine wool sheep, with the *KAZALD1, MYOC, C1QTNF6, FOS, ITGAM, MX2, MX1* and *IFI6* genes being the most critical. The results of this study can provide theoretical reference for the molecular breeding of sheep and other wool domestic animals, as well as further research on the origin and evolution of animal hair.

## Data Availability

The raw data supporting the conclusion of this article will be made available by theauthors, without undue reservation.
