# Supplementary material for: Transcriptome meta-analysis reveals the hair genetic rules in six animal breeds and genes associated with wool fineness
Source: Front Genet. 2024 Jun 14;15:1401369. doi: 10.3389/fgene.2024.1401369 (PMC11211574; doi:10.3389/fgene.2024.1401369)
Supplement: Supplementary file 1 [file DataSheet1.ZIP › attachments/Figure S1.docx]

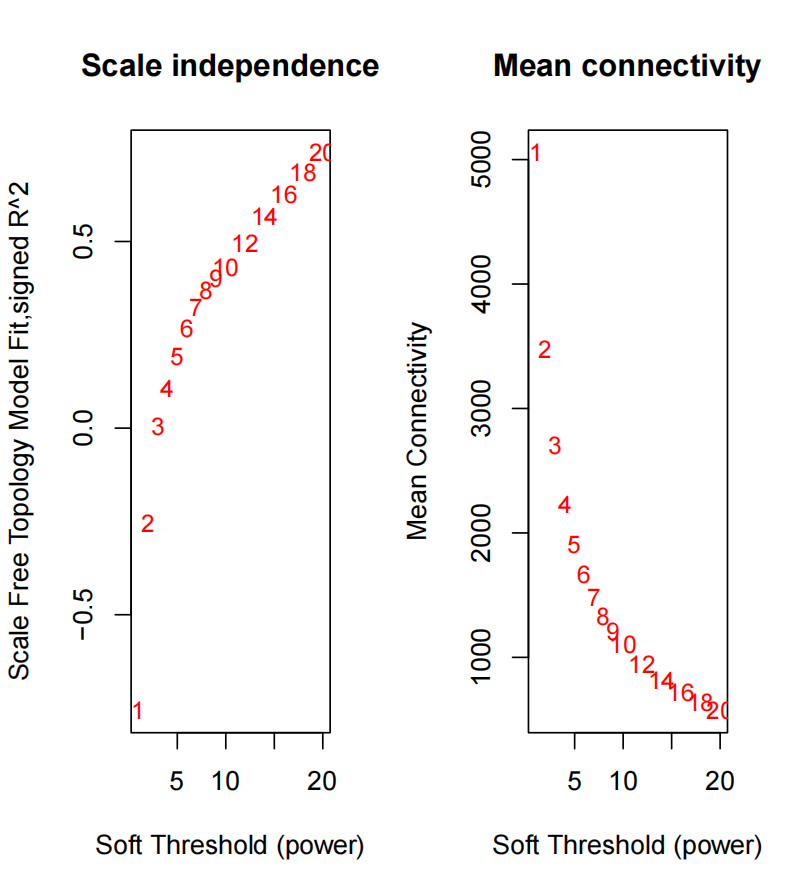


**Figure S1 Analysis of network topology for various soft-thresholding powers.** The left panel shows the scale-free fitindex (y-axis) as a function of the soft-thresholding power (x-axis). The right panel displays the mean connectivity(degree, y-axis) as a function of the soft-thresholding power (x-axis).
