# Supplementary material for: Transcriptome meta-analysis reveals the hair genetic rules in six animal breeds and genes associated with wool fineness
Source: Front Genet. 2024 Jun 14;15:1401369. doi: 10.3389/fgene.2024.1401369 (PMC11211574; doi:10.3389/fgene.2024.1401369)
Supplement: Supplementary file 1 [file DataSheet1.ZIP › attachments/Figure S3.docx]

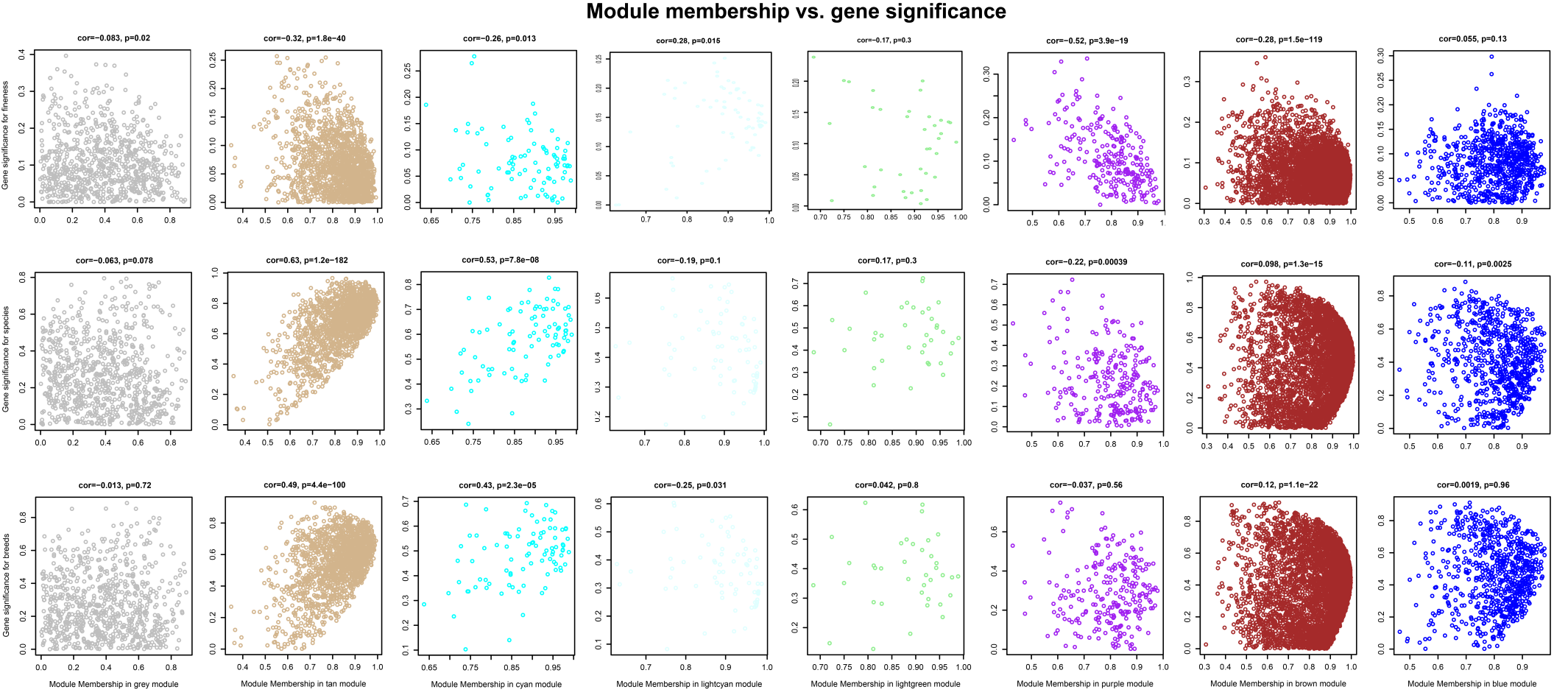


**Figure S3 A scatterplot of Gene Significance (GS) for weight vs. Module Membership (MM) in the 8 module. There is a highly significant correlation between GS and MM in these module.**
