# Supplementary material for: Transcriptome meta-analysis reveals the hair genetic rules in six animal breeds and genes associated with wool fineness
Source: Front Genet. 2024 Jun 14;15:1401369. doi: 10.3389/fgene.2024.1401369 (PMC11211574; doi:10.3389/fgene.2024.1401369)
Supplement: Supplementary file 1 [file DataSheet1.ZIP › attachments/Figure S4.docx]

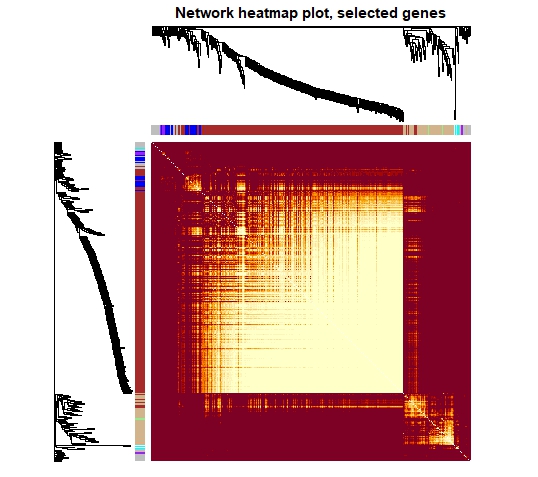


**Figure S4 Visualizing the gene network using a heatmap plot.** The heatmap depicts the Topological Overlap Matrix (TOM) among all genes in the analysis. Light color represents low overlap and progressively darker red colorrepresents higher overlap. Blocks of darker colors along the diagonal are the modules. The gene dendrogram andmodule assignment are also shown along the left side and the top.
