# Supplementary material for: Transcriptome meta-analysis reveals the hair genetic rules in six animal breeds and genes associated with wool fineness
Source: Front Genet. 2024 Jun 14;15:1401369. doi: 10.3389/fgene.2024.1401369 (PMC11211574; doi:10.3389/fgene.2024.1401369)
Supplement: Supplementary file 1 [file DataSheet1.ZIP › attachments/Table S1、S2.docx]

**附件**

**Table S1 The results of the transcriptome data quality control.**

**Table S2 GO enrichment analysis of genes in different modules.**

| **Module** | **Category** | **Term** | **Count** | **%** | ***P*** | **Genes** |
| --- | --- | --- | --- | --- | --- | --- |
| **blue** | **BP** | GO:0051301~cell division | 19 | 2.52 | 0.00 | *ANAPC13, SPAG5, CDCA3, MIS12, KIF14, CDCA8, KIF11, CHFR, NDC80, AURKA, SGO1, CDC20, ASPM, TPX2, CDC45, ENSA, CKS2, FBXO5, LZTS2* |
| **blue** | **BP** | GO:0006270~DNA replication initiation | 6 | 0.80 | 0.00 | *CDC45, MCM3, MCM4, MCM5, MCM10, MCM2* |
| **blue** | **BP** | GO:0007094~mitotic spindle assembly checkpoint | 6 | 0.80 | 0.00 | *IK, ZWILCH, BUB1B, TRIP13, BUB1, MAD2L1* |
| **blue** | **BP** | GO:0006260~DNA replication | 8 | 1.06 | 0.00 | *POLB, ING4, GINS1, SUPT16H, SLX4, RPA1, WRNIP1, TICRR* |
| **blue** | **BP** | GO:0042127~regulation of cell proliferation | 10 | 1.33 | 0.00 | *CNN2, SQLE, LAMA5, FA2H, EGLN3, SHC1, PLAU, CDCA7, E2F4, TCFL5* |
| **blue** | **BP** | GO:0046726~positive regulation by virus of viral protein levels in host cell | 3 | 0.40 | 0.00 | *DDB1, TBC1D20, STAU1* |
| **blue** | **BP** | GO:0021510~spinal cord development | 4 | 0.53 | 0.01 | *MED12, VIT, PHGDH, ZPR1* |
| **blue** | **BP** | GO:0000398~mRNA splicing, via spliceosome | 10 | 1.33 | 0.01 | *SF3A3, HNRNPM, IK, HNRNPA2B1, CWC25, SNRPA1, DDX41, SNRNP200, HTATSF1, TXNL4A* |
| **blue** | **BP** | GO:0006506~GPI anchor biosynthetic process | 5 | 0.66 | 0.01 | *PIGC, PIGO, PIGQ, MPPE1, PIGV* |
| **blue** | **BP** | GO:0051315~attachment of mitotic spindle microtubules to kinetochore | 3 | 0.40 | 0.01 | *CHAMP1, KIF2C, NDC80* |
| **blue** | **BP** | GO:0045927~positive regulation of growth | 3 | 0.40 | 0.01 | *WFS1, TFCP2L1, ZPR1* |
| **blue** | **BP** | GO:0097421~liver regeneration | 3 | 0.40 | 0.01 | *GLI1, AURKA, SULF2* |
| **blue** | **BP** | GO:0007049~cell cycle | 11 | 1.46 | 0.01 | *ANAPC13, TPX2, CDC45, ENSA, MCM3, MCM4, MCM5, KIF11, LZTS2, MKI67, MCM2* |
| **blue** | **BP** | GO:0006281~DNA repair | 10 | 1.33 | 0.01 | *SUPT16H, RRM1, NSMCE4A, FANCD2, RUVBL1, SWI5, RPA1, RAD1, WRNIP1, DMAP1* |
| **blue** | **BP** | GO:0007080~mitotic metaphase plate congression | 5 | 0.66 | 0.01 | *PSRC1, DCTN2, KIF14, CDCA8, RAB11A* |
| **blue** | **BP** | GO:0090307~mitotic spindle assembly | 5 | 0.66 | 0.02 | *CDC20, MYBL2, NEK2, AAAS, RAB11A* |
| **blue** | **BP** | GO:0030968~endoplasmic reticulμm unfolded protein response | 5 | 0.66 | 0.02 | *WFS1, AMFR, DERL1, HERPUD1, TMTC4* |
| **blue** | **BP** | GO:0048704~embryonic skeletal system morphogenesis | 5 | 0.66 | 0.02 | *MYCN, HOXB2, HOXC9, HOXB6, HOXB5* |
| **blue** | **BP** | GO:0008283~cell proliferation | 9 | 1.19 | 0.02 | *DAZAP1, TXNRD1, PPP1R8, CKS2, MCM10, BAK1, DDX41, BYSL, E2F8* |
| **blue** | **BP** | GO:0051220~cytoplasmic sequestering of protein | 3 | 0.40 | 0.02 | *YWHAB, DZIP1, DBN1* |
| **blue** | **BP** | GO:0051726~regulation of cell cycle | 11 | 1.46 | 0.02 | *TFDP1, FIGNL1, MADD, RUVBL1, BAK1, DMAP1, FOXM1, MASTL, PRR11, KAT14, PPM1G* |
| **blue** | **BP** | GO:0010971~positive regulation of G2/M transition of mitotic cell cycle | 4 | 0.53 | 0.02 | *RRM1, RCC2, FBXO5, RAB11A* |
| **blue** | **BP** | GO:0007018~microtubule-based movement | 7 | 0.93 | 0.03 | *DNAH10, KIF18B, KIF14, KIF2C, KIF11, KIF1A, KIF15* |
| **blue** | **BP** | GO:0032147~activation of protein kinase activity | 4 | 0.53 | 0.03 | *TPX2, PRKCD, KIF14, CLSPN* |
| **blue** | **BP** | GO:0006885~regulation of pH | 4 | 0.53 | 0.03 | *EDN1, EDNRB, ATP6V0A4, RHCG* |
| **blue** | **BP** | GO:0090267~positive regulation of mitotic cell cycle spindle assembly checkpoint | 3 | 0.40 | 0.03 | *GEN1, NDC80, MAD2L1* |
| **blue** | **BP** | GO:0070294~renal sodiμm ion absorption | 3 | 0.40 | 0.03 | *EDN1, EDNRB, WNK4* |
| **blue** | **BP** | GO:0007019~microtubule depolymerization | 3 | 0.40 | 0.03 | *KIF18B, NCKAP5L, KIF2C* |
| **blue** | **BP** | GO:0030433~ubiquitin-dependent ERAD pathway | 6 | 0.80 | 0.03 | *WFS1, AMFR, DERL1, TRIM25, RNF185, HERPUD1* |
| **blue** | **BP** | GO:0035307~positive regulation of protein dephosphorylation | 4 | 0.53 | 0.03 | *CDH5, PTBP1, PRKCD, PPP2R5D* |
| **blue** | **BP** | GO:0045892~negative regulation of transcription, DNA-templated | 13 | 1.72 | 0.03 | *SMARCE1, SHC1, NONO, ING4, ILF3, CENPF, TFAP4, DEPDC1, PDCD4, DMAP1, NELFCD, CRYAB, CDK5R1* |
| **blue** | **BP** | GO:0007224~smoothened signaling pathway | 6 | 0.80 | 0.03 | *DYRK2, IFT27, HES1, DZIP1, IFT46, GLI1* |
| **blue** | **BP** | GO:0031146~SCF-dependent proteasomal ubiquitin-dependent protein catabolic process | 4 | 0.53 | 0.04 | *CCNF, KIF14, FBXO48, RBX1* |
| **blue** | **BP** | GO:2001022~positive regulation of response to DNA damage stimulus | 3 | 0.40 | 0.04 | *NSMCE4A, MYC, PRKCD* |
| **blue** | **BP** | GO:0001666~response to hypoxia | 7 | 0.93 | 0.04 | *EGLN3, EDN1, PLAU, CHRNA7, SRF, MMP2, CRYAB* |
| **blue** | **BP** | GO:0006264~mitochondrial DNA replication | 3 | 0.40 | 0.05 | *MGME1, RRM1, DNAJA3* |
| **blue** | **BP** | GO:0021612~facial nerve structural organization | 3 | 0.40 | 0.05 | *EGR2, NRP2, HOXB2* |
| **blue** | **BP** | GO:0002218~activation of innate immune response | 4 | 0.53 | 0.05 | *SFPQ, MAVS, NONO, TLR4* |
| **blue** | **CC** | **GO:0005654~nucleoplasm** | **110** | 14.59 | 0.00 | *SMARCB1, ATN1, NOC2L, CDC20, EDC4, CDH5, MYC, DAG1, RCC1, NEK2, FBXO5, TXNL4A, CDKL2, MED1, GTF3A, RBX1, ACLY, ILF3, MYCN, EWSR1, HOXB2, MPPE1, SNRNP200, SRSF7, GLYR1, HOXB5, CDCA7, CDCA8, AAAS, DHX33, RAD21, DPH3, DND1, TSPAN2, CLSPN, PATZ1, HOXC9, HOXC8, HOXC6, TFAP2C, JUN, EGR2, NOP14, NDC80, MLXIPL, TOP2A, DAZAP1, DYRK2, BNC1, MCM10, GLI1, CHTF18, IRF2BPL, LMNB1, SPR, XPO5, MLX, MYBL2, CTSC, RBM17, DFFB, TSFM, DDX54, VEZF1, PPRC1, MASTL, ETV5, DUSP6, MED28, DDB1, GCHFR, PSRC1, TRAF4, HNRNPH1, NUP50, OAT, FAAP24, SPTY2D1, HOXD11, SDAD1, PPM1G, AURKA, NOL6, CHRND, SLX4, EXOSC10, TCOF1, STX6, HES1, E2F4, BUB1, E2F8, SMARCE1, TRMT10C, NFYA, WDR18, TXNRD1, ARID3A, TICRR, BYSL, CENPF, TFAP4, RFWD3, HNRNPA2B1, RAD1, CENPN, PTPN6, EIF3A, CDK5R1, MAD2L1* |
| **blue** | **CC** | GO:0016363~nuclear matrix | 10 | 1.33 | 0.00 | *HNRNPM, CENPF, SFPQ, NONO, RAD21, PRKCD, ATN1, HNRNPU, DCAF7, LMNB1* |
| **blue** | **CC** | GO:0005730~nucleolus | 37 | 4.91 | 0.00 | *TOP2A, DDX24, CDCA8, MCM10, SPTY2D1, NOC2L, SDAD1, NOL6, EXOSC10, PDCD11, TERT, DHX33, MYC, NUSAP1, RRP12, UTP14A, E2F8, MED1, DFFB, NOM1, NOP14, WDR18, NSUN2, DDX54, BYSL, RSL1D1, WEE1, ILF3, MYCN, WDR82, FANCD2, EWSR1, GEMIN4, TCEA1, PTPN6, EIF3A, ZPR1* |
| **blue** | **CC** | GO:0000922~spindle pole | 10 | 1.33 | 0.00 | *TPX2, CENPF, PSRC1, DCTN1, CEP19, UNC119, NEK2, AAAS, RAE1, RAB11A* |
| **blue** | **CC** | **GO:0005634~nucleus** | **103** | 13.66 | 0.00 | *IPO13, GABPB2, CCNF, NAB2, HNRNPU, CWC25, NOC2L, MKI67, MYC, SOX4, SGO1, ILF3, EWSR1, SRSF7, PRR11, HOXB6, RHNO1, HOXB5, DLX1, L3MBTL2, ZC4H2, CDCA7, SUPT6H, BAG5, ALX4, PATZ1, HOXC9, HOXC8, HOXC6, TFAP2C, JUN, EGR2, BRPF1, VDR, RCC2, TPX2, LHX2, EIF4G1, DAZAP2, FOXE3, LDB1, BNC1, CITED2, SETD7, ARPC1A, NCAPG2, HSPB1, TFCP2L1, GLI1, FOXM1, IRF2BPL, POLB, PTBP1, LBH, NOB1, CASP2, NELFCD, TRIM66, ADIG, TEAD3, SREBF1, PPRC1, MASTL, ASPM, TFDP1, NSMCE4A, POLR1B, MCM3, DEPDC1, MCM4, CRYAB, MCM2, POLDIP2, SRF, MIS12, PBXIP1, TYMS, HOXD11, CDC45, SERTAD4, PLAGL2, IGF2BP2, MSX1, RBBP7, EGLN3, LIMK2, IRX4, DONSON, RPA1, ARID3C, HNRNPAB, TICRR, HEYL, WEE1, RAD51, DNAJA3, IMPDH2, HNRNPA2B1, ZYX, CENPL, CENPM, RAD1, PFKM* |
| **blue** | **CC** | GO:0000228~nuclear chromosome | 5 | 0.66 | 0.00 | *TOP2A, IK, SMARCB1, FIGNL1, HNRNPU* |
| **blue** | **CC** | **GO:0005737~cytoplasm** | **90** | 11.94 | 0.00 | *TOP2A, IPO13, DAZAP2, KIAA1191, CEP19, CCNF, ARPC1A, CIB2, HSPB1, TFCP2L1, KIF15, POLB, LBH, ENSA, NUSAP1, DAG1, CASP2, RCC1, FBXO5, PHLDA2, SKP2, GTSE1, CEP55, YWHAG, ADIG, DFFB, SREBF1, EDN1, PABPC4, USP2, PGD, DDB1, ASPM, ILF3, TRAF4, MADD, FSCN1, BIRC5, ELMO3, TTLL12, EZR, IFT46, EIF4E2, PRR11, CRYAB, ARF5, DCTN2, SHC1, ZC4H2, SRF, RGS16, TSR3, WNK4, CDCA7, MTIF3, IQGAP3, CRACR2A, TERT, NRIP2, PRKAR2A, MICAL1, HES1, E2F4, DCAF12, DTD2, TOE1, EGR2, DNAH10, PMM1, VDR, NDE1, EIF2AK1, ARID3C, HNRNPAB, BYSL, HEYL, TPX2, WEE1, CENPF, MYBBP1A, IMPDH2, HNRNPA2B1, PDCD4, IFT27, PTPN6, EPPK1, GALK1, ZPR1, EIF4G1, OTUD3* |
| **blue** | **CC** | GO:0000775~chromosome, centromeric region | 6 | 0.80 | 0.00 | *SGO1, TOP2A, CENPF, RAD21, CENPL, CDCA8* |
| **blue** | **CC** | GO:0000776~kinetochore | 9 | 1.19 | 0.00 | *DCTN2, NDE1, MIS12, CHAMP1, HNRNPU, KIF2C, NEK2, BUB1, MAD2L1* |
| **blue** | **CC** | GO:0042555~MCM complex | 4 | 0.53 | 0.00 | *MCM3, MCM4, MCM5, MCM2* |
| **blue** | **CC** | GO:0071013~catalytic step 2 spliceosome | 6 | 0.80 | 0.01 | *SF3A3, HNRNPM, HNRNPA2B1, HNRNPU, DDX41, SNRNP200* |
| **blue** | **CC** | GO:0035371~microtubule plus-end | 4 | 0.53 | 0.01 | *KIF18B, DCTN1, NCKAP5L, KIF2C* |
| **blue** | **CC** | GO:0071005~U2-type precatalytic spliceosome | 6 | 0.80 | 0.01 | *SF3A3, IK, SNRPA1, SNRNP200, TXNL4A, PRPF8* |
| **blue** | **CC** | GO:0031965~nuclear membrane | 11 | 1.46 | 0.02 | *GCHFR, CDH5, CMTM3, TBC1D20, RB1CC1, LMNA, MLX, RCC1, TMEM97, HACD3, TXNL4A* |
| **blue** | **CC** | GO:0015030~Cajal body | 5 | 0.66 | 0.02 | *COIL, TOE1, GEMIN4, NOLC1, ZPR1* |
| **blue** | **CC** | GO:0005694~chromosome | 9 | 1.19 | 0.02 | *RSL1D1, POLR1B, SETD7, MCM3, MCM4, RAD1, NOC2L, BYSL, MCM2* |
| **blue** | **CC** | **GO:0005829~cytosol** | **97** | 12.86 | 0.02 | *PANK3, UBXN2A, HNRNPU, TTF2, NOC2L, DCAF7, CDC20, RUVBL1, TRIM25, TXNL4A, LZTS2, TPM3, PRKCD, CMBL, DGKZ, RBX1, ACLY, FANCD2, MAP7D1, MADD, GEMIN4, GLYR1, HOXB5, CHODL, CDCA7, AAAS, STRIP2, BAG5, PDCD11, DPH3, DMAP1, HOXC6, PMM1, UBE2C, MLXIPL, MEX3A, KIF18B, TUBGCP3, DZIP1, EIF4G1, B4GALT3, DAZAP1, MOCS2, DYRK2, YWHAB, KIF14, PYGL, CHTF18, ING4, SPR, RB1CC1, XPO5, MLX, VCPKMT, RRP12, AMPD2, DUSP6, GCHFR, SLC7A5, CREB3, RAP2B, PSRC1, TMEM214, TRAF4, POLR1B, CCDC43, CRYAB, ARF6, PDXK, DCTN2, STAU1, PBXIP1, TOB2, AURKA, CHRND, SLX4, EXOSC10, TCOF1, STX6, NCDN, BAK1, RBBP7, SEC23B, BUB1, E2F8, EGLN3, CMTM3, TXNRD1, ARID3A, TICRR, DNAJA3, IMPDH2, ZYX, CENPM, PFKM, EIF3A, MAD2L1* |
| **blue** | **CC** | GO:0042382~paraspeckles | 3 | 0.40 | 0.02 | *HNRNPM, SFPQ, NONO* |
| **blue** | **CC** | GO:0090543~Flemming body | 4 | 0.53 | 0.02 | *CHAMP1, KIF14, CEP55, ARF6* |
| **blue** | **CC** | GO:0005813~centrosome | 20 | 2.65 | 0.03 | *CDKL2, CHODL, GEN1, DCTN2, LIMK2, NCKAP5L, CCNF, HNRNPU, AAAS, MASTL, NDC80, PRKAR2A, MCM3, IFT27, UNC119, TUBGCP3, NEK2, IFT46, DCAF12, CTSC* |
| **blue** | **CC** | GO:0005819~spindle | 6 | 0.80 | 0.03 | *ASPM, MYC, CHAMP1, NUSAP1, HSPB1, KIF15* |
| **blue** | **CC** | GO:0001650~fibrillar center | 9 | 1.19 | 0.03 | *DUSP11, COIL, SMARCB1, TCOF1, TRAF4, NONO, TXNRD1, NOLC1, HOXB5* |
| **blue** | **CC** | GO:0031464~Cul4A-RING E3 ubiquitin ligase complex | 3 | 0.40 | 0.03 | *DDB1, CUL4A, RBX1* |
| **blue** | **CC** | GO:0000940~condensed chromosome outer kinetochore | 3 | 0.40 | 0.03 | *CENPF, BUB1B, NDC80* |
| **blue** | **CC** | GO:0042645~mitochondrial nucleoid | 5 | 0.66 | 0.04 | *HADHA, TRMT10C, POLDIP2, SHMT2, DNAJA3* |
| **blue** | **CC** | GO:0071006~U2-type catalytic step 1 spliceosome | 3 | 0.40 | 0.04 | *CWC25, SNRNP200, PRPF8* |
| **blue** | **CC** | GO:0016607~nuclear speck | 17 | 2.25 | 0.04 | *SF3A3, DAZAP2, IK, POLDIP3, NONO, NCAPG2, HNRNPU, CWC25, DGKZ, DUSP11, SFPQ, STK17A, PPP1R8, RNPS1, SNRPA1, DZIP1, E2F7* |
| **blue** | **MF** | **GO:0005524~ATP binding** | **70** | 9.28 | 0.00 | *TOP2A, DYRK2, PANK3, KIF14, HNRNPU, BUB1B, TTF2, PYGL, KIF11, DDX41, CHTF18, KIF15, RUVBL1, NEK2, TK1, KIF1A, XYLB, CDKL2, CCT3, ENTPD3, PRKCD, ENTPD6, DDX54, WRNIP1, KSR2, MASTL, DGKZ, NRBP1, AFG3L2, ACLY, MCM3, MCM4, TSSK6, KIF2C, MCM5, EPHA1, TRIB1, SNRNP200, MCM2, DDX5, PDXK, DDX24, WNK4, ADCY7, AURKA, DHX33, FIGNL1, BUB1, MYH7B, RRM1, DNAH10, UBE2I, UBE2C, LIMK2, EIF2AK1, ABCA4, DCLK3, WEE1, P2RX5, RAD51, KIF18B, STK17A, DNAJA3, MYO5C, HYOU1, TRIP13, PFKM, GALK1, ITM2C, EIF4G1* |
| **blue** | **MF** | GO:0016887~ATPase activity | 19 | 2.52 | 0.00 | *CCT3, DDX5, DDX24, ABCA4, KIF14, DDX54, WRNIP1, DDX41, CHTF18, AFG3L2, DHX33, FIGNL1, RUVBL1, MCM3, MCM4, MCM5, HYOU1, TRIP13, MCM2* |
| **blue** | **MF** | GO:0010997~anaphase-promoting complex binding | 4 | 0.53 | 0.00 | *CDC20, CCNF, CLSPN, FBXO5* |
| **blue** | **MF** | GO:0008017~microtubule binding | 14 | 1.86 | 0.00 | *DCTN1, RCC2, NDE1, KIF14, KIF11, GLI1, RAB11A, KIF15, POLB, CENPF, PSRC1, KIF18B, NUSAP1, KIF1A* |
| **blue** | **MF** | GO:0003723~RNA binding | 29 | 3.85 | 0.01 | *TRUB2, DAZAP1, STAU1, POLDIP3, HTATSF1, NOL6, DUSP11, PTBP1, EIF4H, RBBP7, SMARCE1, SF3A3, RBM17, NOM1, PABPC4, NONO, ADAT1, DDX54, PPRC1, HNRNPAB, HNRNPM, SFPQ, MEX3A, HNRNPH1, EWSR1, HNRNPA2B1, RNPS1, RBMX, SRSF7* |
| **blue** | **MF** | GO:0001228~transcriptional activator activity, RNA polymerase II transcription regulatory region sequence-specific binding | 19 | 2.52 | 0.01 | *SREBF1, EGR2, JUN, TFAP2C, SRF, VEZF1, ETV5, CREB3, HEYL, MYCN, TFAP4, PLAGL1, MYC, PLAGL2, MYBL2, HOXB2, E2F4, PATZ1, HOXB5* |
| **blue** | **MF** | GO:0003743~translation initiation factor activity | 7 | 0.93 | 0.01 | *EIF2AK1, EIF4H, EIF4EBP2, MTIF3, EIF4E2, EIF3A, EIF4G1* |
| **blue** | **MF** | GO:0042802~identical protein binding | 43 | 5.70 | 0.01 | *DAZAP2, GABPB2, OAT, DCTN2, YWHAB, NAB2, DERL1, HNRNPU, MCM10, PYGL, TNFAIP1, SMPD3, COIL, SCUBE3, KCTD21, CASP2, KIF1A, CEP55, E2F7, E2F8, RRM1, JUN, IK, TRMT10C, DYNLT1, NONO, NDE1, AMFR, USP2, ARID3A, SμmF2, NDC80, CREB3, TMX2, TRAF4, EWSR1, TRIP13, SNRNP200, RHCG, TLR4, PFKM, HCN2, EIF4G1* |
| **blue** | **MF** | GO:0030674~protein binding, bridging | 6 | 0.80 | 0.01 | *APH1A, DDB1, TCOF1, AMFR, FSCN1, NOLC1* |
| **blue** | **MF** | GO:0019901~protein kinase binding | 17 | 2.25 | 0.02 | *SREBF1, DCTN2, RCC2, PRKCD, KIF14, FOXM1, AURKA, MAVS, BAG5, TRAF4, MICAL1, RB1CC1, PTPN6, RAC1, FBXO5, EPHA1, CDK5R1* |
| **blue** | **MF** | GO:0003677~DNA binding | 45 | 5.97 | 0.02 | *TOP2A, DLX1, SMARCB1, LDB1, POLDIP2, DDX41, SPTY2D1, HOXD11, SUPT6H, POLB, SLX4, TERT, MYC, NUSAP1, ALX4, MSX1, HOXC9, HOXC8, SOX4, HOXC6, SAMD11, TEAD3, SMARCE1, MED1, DFFB, JUN, TFAP2C, EGR2, NFYA, IRX4, RPA1, ARID3A, WRNIP1, ARID3C, ILF3, TFDP1, LHX2, POLR1B, MCM3, MCM4, TCEA1, HOXB6, MCM2, GLYR1, HOXB5* |
| **blue** | **MF** | GO:0051117~ATPase binding | 6 | 0.80 | 0.02 | *WFS1, RUVBL1, DERL1, VCPKMT, ATP6V0A4, TMTC4* |
| **blue** | **MF** | GO:0000981~RNA polymerase II transcription factor activity, sequence-specific DNA binding | 13 | 1.72 | 0.02 | *DLX1, IRX4, GLI1, HOXD11, LHX2, MYC, ALX4, MYBL2, MSX1, HOXC9, HOXC8, HOXB6, HOXC6* |
| **blue** | **MF** | GO:0097602~cullin family protein binding | 4 | 0.53 | 0.03 | *DDB1, KLHL36, KCTD21, RBX1* |
| **blue** | **MF** | GO:0003777~microtubule motor activity | 5 | 0.66 | 0.03 | *KIF18B, KIF2C, KIF11, KIF1A, KIF15* |
| **blue** | **MF** | GO:0019904~protein domain specific binding | 10 | 1.33 | 0.03 | *MED12, HNRNPM, RAP2B, YWHAB, PRKAR2A, RCC2, ATN1, CASP2, E2F4, SRSF7* |
| **blue** | **MF** | GO:0070700~BMP receptor binding | 3 | 0.40 | 0.04 | *CDH5, BMP3, SCUBE3* |
| **blue** | **MF** | GO:0031996~thioesterase binding | 3 | 0.40 | 0.04 | *TRAF4, RAC1, ARF6* |
| **blue** | **MF** | GO:0003682~chromatin binding | 15 | 1.99 | 0.04 | *TOP2A, SREBF1, DLX1, EGR2, LDB1, SHMT2, NONO, FAAP24, ARID3A, ARID3C, GLI1, TICRR, MED12, SFPQ, CKS2* |
| **blue** | **MF** | GO:0008022~protein C-terminus binding | 8 | 1.06 | 0.05 | *MED12, CDC20, COIL, TOP2A, CENPF, PEX12, PFKM, MAD2L1* |
| **blue** | **MF** | GO:0044877~macromolecular complex binding | 9 | 1.19 | 0.05 | *DDB1, MED1, POLDIP3, MYC, TSSK6, KCTD2, RAC1, RNF185, CRYAB* |
| **blue** | **MF** | GO:0031267~small GTPase binding | 12 | 1.59 | 0.05 | *IPO13, CYFIP2, AP3M1, RIMS4, TBC1D20, ATP6AP1, MICAL1, RCC2, XPO5, RCC1, TNFAIP1, IQGAP3* |
| **brown** | **BP** | GO:0035556~intracellular signal transduction | **116** | 1.84 | 0.00 | *DGKE, DGKD, TRAF3IP1, DGKB, ITSN1, CBLB, RPS6KA3, PREX1, RPS6KA5, RPS6KA2, AKT3, RGS6, RGS7, UNC13B, GUCY1A2, PRKCI, PRKCH, WSB1, WSB2, DAPK1, PRKCB, PRKCE, PRKCA, ADCY9, PRKD3, PRKCQ, SIK2, PRKD1, TNIK, RAF1, DGKI, DGKH, SHC4, SHC3, ARHGEF28, NPR2, STK39, RASAL1, PSEN1, PRKCZ, ABR, INPP5D, DVL1, DVL3, STK38L, PLEK2, ECT2, SOCS6, PDZD8, SOCS4, PAG1, SOCS5, SRPK2, LYN, SMAD4, STAC, ARHGAP29, DCLK2, BRAF, BMX, SRPK1, NET1, PLEKHM1, CSPG4, GSK3B, ITK, PLEK, PRKAG2, TLK2, BLNK, JAK2, JAK1, SS18, PLEKHG7, SYK, TGFBR1, CIT, TGFBR3, PLCB4, TRAF6, RASA2, KIT, ARHGEF3, DEPDC5, PLCB1, PLCB2, ARHGEF5, ARHGEF6, GUCY2C, RGS14, CAB39, SRC, ADCY3, ADCY2, ASB11, NOD1, ADCY8, RAB40B, RAB40C, CHN2, MKNK1, CHN1, GPR155, ARFGEF2, MAP3K3, PLCL2, STAC3, CDC42BPB, DEF8, CDC42BPA, MYO9A, PIKFYVE, ASB7, ASB2, PLCD4, ASB3* |
| **brown** | **BP** | GO:1905515~non-motile ciliμm assembly | 31 | 0.49 | 0.00 | *C2CD3, INTU, GORAB, IFT172, IFT74, IFT52, PCM1, ARL13B, TBC1D32, POC1A, NPHP3, CEP250, BBS7, DISC1, IFT57, PIBF1, CC2D2A, BBS4, DYNC2H1, BBS2, IFT140, CEP350, IFT122, CSNK1D, IFT80, TTC8, BBS10, CENPJ, MKS1, EXOC5, CEP89* |
| **brown** | **BP** | GO:0018105~peptidyl-serine phosphorylation | 47 | 0.75 | 0.00 | *GSK3B, SMG1, CAB39, ROCK1, PRKDC, TNKS, ROCK2, LRRK2, SLC1A1, PDGFB, STK39, TTK, IKBKB, MAPK9, MAPK8, TBK1, GRK2, MKNK1, TLK2, MAPK1, RICTOR, PAK2, IKBKE, SRPK2, HIPK4, MORC3, PRKCI, SYK, PRKCE, NEK6, CSNK2A2, PLK1, DYRK1A, LMTK2, MAPK14, HIPK3, MTOR, TGFBR1, TGFBR2, HIPK2, UHMK1, MAPKAPK3, RPS6KB1, WNK1, BCL2, MAP3K13, ATR* |
| **brown** | **BP** | **GO:0006511~ubiquitin-dependent protein catabolic process** | **72** | 1.14 | 0.00 | *USP37, RNF11, USP31, USP32, USP33, USP34, UBE2D1, UBE3A, ARIH2, ARIH1, FBXO8, FBXO7, USP46, USP47, USP8, USP48, USP7, AREL1, USP49, DTX3L, USP42, USP4, USP3, USP45, UCHL3, UCHL5, PSMA6, RNF125, WDR81, USP40, CACUL1, DTL, USP13, USP15, USP16, CUL5, UBA6, USP10, CUL3, CUL2, CUL1, RNF8, UBR4, NEDD4L, CBL, RCHY1, USP19, PSMA7, USP1, RBBP6, USP24, RNF20, USP25, HACE1, SMURF2, SMURF1, USP9X, SIAH2, SIAH1, WWP1, KLHL3, NTAN1, USP28, FBXL12, CNOT4, RNF146, NPLOC4, TTC3, KLHL8, UBE2N, LNX1, CUL4B* |
| **brown** | **BP** | GO:0043547~positive regulation of GTPase activity | 55 | 0.87 | 0.00 | *BCAR3, DENND1B, GSK3B, DOCK8, WDR41, SNX13, PREX1, EVI5L, RALBP1, RALGAPA1, RALGAPA2, ELMOD1, ARAP2, TSC2, RGMA, TBC1D2, RP2, RASA1, RAPGEF1, RAPGEF2, ITGA6, PKP4, MAPRE2, ARHGEF5, RABGAP1, RGS14, RAP1GDS1, ASAP3, ASAP1, ASAP2, ABR, ADAP2, CHN2, CHN1, DVL3, SNX9, RSU1, RAB11FIP2, SRGAP2, EVI5, RABGAP1L, RIC1, SEMA4D, MYO9A, NET1, RGP1, AFDN, SGSM3, SGSM2, RGS10, NF1, PLXNB1, FERMT2, RAB3GAP1, PICALM* |
| **brown** | **BP** | GO:0006974~cellular response to DNA damage stimulus | 50 | 0.79 | 0.00 | *CCNK, FMR1, ASH2L, SMC6, CHD2, AATF, ALKBH8, CASP9, UFL1, NIPBL, STK11, HELB, ZC3H12A, TLK2, CTLA4, TOPBP1, VAV3, NCOA6, SIRT4, BAZ1B, WDR76, KIN, RNF169, TRAF6, YAP1, DCUN1D5, STXBP4, BRAT1, BOD1L1, TANK, ZBTB40, RBBP5, UBR5, MAPK1, RBBP6, SUSD6, MORC2, UBQLN4, CTC1, LYN, TIGAR, SLF1, SLF2, NEK4, ETAA1, BCL6, APC, CDK3, SPDYA, NRDE2* |
| **brown** | **BP** | GO:0045944~positive regulation of transcription from RNA polymerase II promoter | **190** | 3.02 | 0.00 | *RB1, PID1, CCNT2, KDM1A, PRDM5, ARID4A, NUCKS1, NR3C1, GABPB1, IKZF2, FGF2, IKZF4, DCAF6, ZNF609, TBK1, RPS6KA5, ZMIZ1, CREB3L1, CREB3L2, ZC3H12A, EPC1, HOXA2, KAT7, SUPT20H, PITX2, PKNOX1, CDON, MEF2A, ACVR1, LMO1, SMARCC1, SLC30A9, MEF2C, WNT5A, CASK, RFX3, TBR1, HNF1A, GTF2F2, SOX10, PYGO1, HOXB9, PRPF6, SMO, TBL1XR1, RAF1, ZFPM2, ATF6, CBFB, KMT2A, TNKS, PRKDC, GATA4, PIK3R1, PRDM10, SBNO2, ATXN7, DVL1, HIVEP1, DVL3, RIPK1, RPRD1B, TP53BP1, PPARGC1A, WNT2, PPARGC1B, BCL9L, BPTF, TFAP2A, AKIRIN2, WWTR1, POU2F1, TFAP2D, JAG1, ECD, LIF, INHBA, INO80, SMARCA2, BMP6, RAX2, MLLT10, BCL9, IL6, FUBP3, CCPG1, AGO2, CAPRIN2, SP3, CD28, ATM, BMPR1B, OGT, ZNF410, FGF10, ACVRL1, CIITA, THRB, BMPR2, SETD3, AKAP8L, SRA1, PSIP1, ARRB1, BRCA1, WASL, LDB2, PKD2, AATF, GLI3, IKBKB, PPP3CA, FLCN, NIPBL, PPP3CB, HEY2, NCK2, NOS1, HTATIP2, ACTR3, NCOA2, ACTR2, BCAS3, SS18, FOXD3, MYOCD, KLF12, PPP1R12A, PARP1, NCOA6, NCOA3, ARNT, AP3B1, PROX1, SENP2, SIRT1, PAX2, SENP1, NR5A1, KAT2B, TOX3, TOX2, ZEB1, THRAP3, TRAF6, TFEC, MTF2, RARA, RARB, CDH13, ITGA6, NCOA7, ITGB1BP1, MET, TLR3, YAP1, KDM3A, RNASEL, NUFIP1, TMPRSS6, ZBTB49, LRP5, NLRC5, RNF4, EGFR, LRP6, MLLT6, IFT74, ABLIM3, NRIP1, HSF4, APBB2, RXRG, EGLN1, ZNF462, TCF7L2, STAT1, MACC1, WWP2, RRP1B, ARID3B, MAPK14, ACVR2A, HIPK2, ASXL1, TEF, ASXL2, CAMK1, PAXBP1, CCDC62* |
| **brown** | **BP** | GO:0046777~protein autophosphorylation | **60** | 0.95 | 0.00 | *ALK, GSK3B, SMG1, LRRK2, IGF1R, STK10, STK11, SLK, TRIM24, KDR, MAP3K9, HTATIP2, PDGFRB, EPHA4, PDGFRA, CDKL5, SYK, CSNK2A1, DAPK1, LMTK2, VRK2, ATG13, UHMK1, PEAK1, KIT, ULK3, TYRO3, SIK2, TNIK, DDR2, CSF1R, SRC, STK39, TTK, NLK, ACVR1B, GRK5, GRK7, TRPM7, CAMK2G, PAK2, LYN, HIPK4, MAP3K3, INSR, NEK6, EIF2AK3, CLK4, MTOR, CLK3, FER, STK24, FES, WNK2, CDK12, TEK, FGFR4, MAP3K13, FGFR2, ATR* |
| **brown** | **BP** | GO:0050852~T cell receptor signaling pathway | 31 | 0.49 | 0.00 | *ITK, DENND1B, CBLB, PTPN22, BCL10, CD3E, MALT1, STK11, NFKBIZ, ZC3H12A, PLCG2, PDE4B, ABL1, MAPK1, PLCG1, EIF2B3, RIPK2, PDE4D, THEMIS, RC3H1, BRAF, RC3H2, PTPRC, CACNB4, WNK1, CD8A, TRAF6, CD28, LCP2, CD247, NFKBID* |
| **brown** | **BP** | GO:0007030~Golgi organization | 30 | 0.48 | 0.00 | *RNFT2, SPTBN5, STX17, LRRK2, ATL2, HTT, SYNE1, ARHGAP21, LMAN1, GOLGA5, VPS51, TRAPPC12, TRAPPC11, CLASP1, CLASP2, DYNC2H1, RAB2A, ARFGEF1, BCAS3, HACE1, COG7, COG6, COG5, COG3, COG2, TRAPPC8, LYSMD3, GAK, ZW10, PLEKHM2* |
| **brown** | **BP** | **GO:0007165~signal transduction** | **108** | 1.71 | 0.00 | *FAM13B, ARHGAP39, FNBP1L, ARHGAP35, ARHGAP42, CCND3, PPP4R1, RASSF3, GRB14, RASSF5, RASSF6, PDE8A, FNBP1, MAGI3, THOC1, OCRL, ANK2, GAPVD1, ANK1, AKAP9, SPARCL1, PDE9A, GRB7, PDE1A, ARHGAP19, ARHGAP17, RASAL2, IQGAP1, ARHGAP15, RASAL3, STK3, INPP5B, ARHGAP22, ARHGAP21, ARHGAP20, GRK2, GRK4, GRK7, GRK6, RIPK1, RHPN2, SRGAP3, SRGAP2, SRGAP1, TGFB2, STARD13, RASSF8, ARHGAP28, RASSF9, ARHGAP26, ARHGAP24, AFDN, ARHGAP32, ARHGAP31, RHEB, GKAP1, PPP1R1C, GNB1, GNB4, NF1, GNB3, GNB5, ARHGAP9, TENM1, ECM1, TENM4, PDE3B, PTPRM, ARHGAP5, PPP2R5E, CD34, PPP1R12A, ARAP2, PPP2R5A, RRAS2, TRAF1, IRAK4, PPP2R5C, RANGAP1, GFRA2, YWHAZ, ARHGAP10, RASA1, TRAF5, TNFRSF25, TLR3, STOML3, PLEKHH3, PKN3, TGFBRAP1, PDE11A, SH3BP1, PTK2B, PCNT, MPZL1, MYO10, STAT3, ERBIN, PDE10A, RPS6KB1, DLC1, FAS, PKN2, KRAS, CAMK1, PDE7B, PDE7A, PIK3AP1* |
| **brown** | **BP** | GO:0035264~multicellular organism growth | 40 | 0.63 | 0.00 | *NOTCH2, WDR48, PKDCC, PLAG1, ANKRD11, XPA, ADD1, GIGYF2, TNKS2, FLVCR1, HESX1, EP300, RBBP6, SLC25A25, WDTC1, CTC1, WWTR1, SLC12A5, PDE4D, XRCC2, HEG1, ATRX, ARID5B, GRHL2, PALB2, RC3H2, MTOR, EXT1, TTC8, SMO, GPD2, SCNN1B, RARA, RARB, ATM, APBA1, ERCC6, SOS1, STK40, PLEC* |
| **brown** | **BP** | GO:0006897~endocytosis | 32 | 0.51 | 0.00 | *ITSN2, SH3GL3, LRRK2, USP33, ITSN1, LRP5, LRP4, SNX33, STON2, LRP2, FNBP1L, PIK3C2A, LRP8, RAB22A, LRP6, GHR, EEA1, MRC2, SNX2, ATP6V1H, WASF2, GTF2A1L, FNBP1, SORT1, GAPVD1, EPN2, MYO1E, NECAP2, NECAP1, RABEP1, STEAP2, PICALM* |
| **brown** | **BP** | GO:0018107~peptidyl-threonine phosphorylation | 27 | 0.43 | 0.00 | *ATF2, GSK3B, PRKDC, TNKS, ROCK2, STK39, TTK, NLK, ACVR1B, MAPK8, STK11, TBK1, GRK2, MAPK1, ACVR1, PDPK1, DYRK1A, LMTK2, HIPK3, MTOR, TGFBR1, TGFBR2, HIPK2, WNK1, BCL2, CDK10, PRKD1* |
| **brown** | **BP** | GO:0006886~intracellular protein transport | 73 | 1.16 | 0.00 | *RAB3B, RAB3C, CSE1L, CLTC, AP4E1, CHM, SNX13, SNX10, ABCA12, IPO8, AP1G1, TOM1, AP3S1, VPS39, WLS, NSF, COG7, COG3, STX7, MYO7A, FAM91A1, SYTL5, SYTL4, SYTL2, CTTN, EXOC6, STX12, NAPB, COPB2, SEC23A, STX17, STX18, TGFBRAP1, USO1, VPS26A, SNX33, VPS26B, SNX31, NAPG, STX11, GGA2, GGA1, SNX1, SNX2, BLOC1S2, VPS11, VTI1A, AP4S1, SNX9, STX3, STX2, MPDZ, SNX5, SNX6, AP1M1, VTI1B, GTF2A1L, RPH3AL, SEC24A, RIC1, RANBP17, EXOC6B, XPO7, AP2B1, AP4B1, WDR11, MLPH, XPOT, APPBP2, VIPAS39, APBA1, SEC24D, SEC24C* |
| **brown** | **BP** | GO:0007179~transforming growth factor beta receptor signaling pathway | 32 | 0.51 | 0.00 | *ACVRL1, USP15, ITGB5, HPGD, ZFYVE9, SRC, LRRC32, NLK, MTMR4, ZMIZ1, ITGB8, ITGB6, SKIL, APPL2, APPL1, ACVR1, SMAD2, TGFB2, SMAD4, SMAD3, USP9X, SMAD9, SMAD5, SIRT1, PML, TGFBR1, TGFBR2, SMAD7, HIPK2, TGFBR3, ADAM9, FERMT2* |
| **brown** | **BP** | GO:0043087~regulation of GTPase activity | 29 | 0.46 | 0.00 | *STMN3, RASAL1, CBLB, RASAL2, IQGAP1, EFNA5, IQGAP2, RASAL3, FGD6, SIPA1L1, RICTOR, EVI5, SBF2, PRKG1, TBC1D15, VAV3, NTRK2, OCRL, GAPVD1, MTOR, GPR137B, BCL6, TRAPPC6B, RASA2, NF1, RAB3GAP2, ITGB1BP1, DGKI, EPHA3* |
| **brown** | **BP** | GO:0050731~positive regulation of peptidyl-tyrosine phosphorylation | 30 | 0.48 | 0.00 | *NRP1, SRC, LRRK1, LRP4, HTR2A, EFNA5, CD3E, FGF8, NTF3, PLCG2, ENPP2, ABL1, PTK2B, RICTOR, ACVR1, SYK, ANGPT1, SEMA4D, RIPK2, IL15, HGF, MTOR, BMP6, PTPRC, BANK1, CSPG4, PLPP3, CD44, FGF10, HBEGF* |
| **brown** | **BP** | GO:0045669~positive regulation of osteoblast differentiation | 27 | 0.43 | 0.00 | *YAP1, FBN2, BMPR2, LRP5, FGF2, GLI3, TP63, CTHRC1, ACVR1, WWTR1, MEF2C, JAG1, SUCO, NPNT, ACVR2B, SMAD5, BMP6, ACVR2A, BMP4, IL6, CTNNB1, PRKD1, IL6ST, BMPR1B, FERMT2, BMPR1A, DDR2* |
| **brown** | **BP** | GO:0031532~actin cytoskeleton reorganization | 26 | 0.41 | 0.00 | *NRP1, GPR65, PLEK, ATP2C1, EPS8, SIPA1L1, PIP5K1A, PLEK2, RICTOR, FARP2, PTPN1, CXADR, GAB1, DTNBP1, PARVA, CDC42BPA, MKLN1, FER, NPHS2, PTK7, KIT, GRB2, TNIK, ASB2, FGF10, PLEC* |
| **brown** | **BP** | GO:0048015~phosphatidylinositol-mediated signaling | 15 | 0.24 | 0.00 | *PDGFRB, CSF1R, PDGFRA, PIK3C2G, PIK3C2A, PIK3C2B, IGF1R, INPP5F, PITPNM2, PI4KA, PIK3C3, PLCH1, PI4KB, OGT, EXOC1* |
| **brown** | **BP** | GO:0051592~response to calciμm ion | 15 | 0.24 | 0.00 | *SUCNR1, AHCYL1, HOMER1, TRPC1, CAV1, ALG2, ANXA11, THBS1, P2RX7, PPP3CA, CAPN3, ADAM9, MNAT1, SLC25A12, SLC25A13* |
| **brown** | **BP** | **GO:0001701~in utero embryonic development** | 73 | 1.16 | 0.00 | *ACVRL1, ATF2, FKBP10, PTPRR, C2CD3, ZFAND5, RTCB, OTUD7B, PTH1R, GLI3, GLI2, FLCN, EDNRA, ADAMTS3, SH3PXD2A, ZMIZ1, ALKBH1, FLVCR1, ACVR1, PDGFRB, PDGFRA, HEG1, ADAM10, ATP11A, MAPK8IP3, SOX10, TANC2, TGFBR2, ADCY9, POLG2, SMO, MSH2, TRAF6, ZFPM2, NOTCH2, RNASEH2B, YTHDC1, NXN, ANKRD11, HM13, SRSF1, UBR3, ACVR1B, ADD1, GNA13, INPP5B, EPB41L5, UNK, C6, MAN2A1, DHX35, RDH10, HSD17B2, RBBP6, PLCG1, MYH10, WDTC1, ARNT2, SMAD4, SMAD3, UBE2B, ANGPT1, KIDINS220, PTCH1, PCGF2, XRCC2, COPS3, MYO1E, CTNNB1, APBA1, SEC24D, ZNF335, BMPR1A* |
| **brown** | **BP** | GO:0060271~ciliμm assembly | 50 | 0.79 | 0.00 | *TRAF3IP1, CEP164, FNBP1L, TXNDC15, TCTN3, TCTN2, TEKT1, NEK1, BBS9, TEKT3, RAB8A, ACTR3, ACTR2, CCDC113, ARMC9, KIF24, RFX3, OCRL, NME5, WDR35, KIF27, IFT43, FAM161A, RFX4, NME8, ALPK1, FAM149B1, ARL6, FBF1, ASAP1, RSPH9, PKHD1, KIF3B, ABLIM3, TMEM67, CEP70, POC1B, PCNT, ZNF423, PLK4, WWTR1, DTNBP1, SCLT1, PARVA, KIAA0586, CEP83, CEP162, ATXN10, CCDC66, CEP89* |
| **brown** | **BP** | GO:0007169~transmembrane receptor protein tyrosine kinase signaling pathway | 19 | 0.30 | 0.00 | *ALK, LYN, NTRK2, CSF1R, CSF1, ANGPT1, FLT3, INSR, MST1R, GFRA2, IGF1R, DOK1, DOK5, NTF3, KIT, ROR1, ROR2, MET, ROS1* |
| **brown** | **BP** | **GO:0006357~regulation of transcription from RNA polymerase II promoter** | 63 | 1.00 | 0.00 | *RB1, SMARCAL1, ATF2, ECM1, CCNT2, CCNT1, CCNH, UBP1, SRA1, CHD5, MED17, EPC2, CCNL2, CCNL1, PRKCB, TSC22D2, MED8, MITF, HNF1A, MED4, MED26, MED6, PIAS2, RBL2, RBL1, CTDSP1, MED20, SOS1, LCORL, CGGBP1, HLF, MAX, LEF1, GLIS3, GATA6, GATA4, AMBRA1, NRF1, RAI1, BRIP1, ZNF704, TTC21B, HSF2, CAMTA1, HSF5, E2F5, BRD7, BPTF, TCF7L1, KDM4C, MYEF2, ELP3, INHBA, SNF8, BATF, FOSL2, MED13L, ZZZ3, TADA2A, HNRNPK, TEF, SP4, MNAT1* |
| **brown** | **BP** | GO:0016579~protein deubiquitination | 28 | 0.44 | 0.00 | *USP13, USP14, USP15, WDR48, USP31, USP53, USP54, USP32, USP10, USP34, USP19, ZC3H12A, USP24, USP46, USP47, USP25, USP48, USP7, USP42, USP4, USP9X, USP3, USP45, YOD1, USP28, JOSD1, UCHL5, USP40* |
| **brown** | **BP** | GO:0007098~centrosome cycle | 17 | 0.27 | 0.00 | *UVRAG, PPP1R12A, XRCC2, CEP120, PLK1, HEPACAM2, HAUS6, BRCA1, CKAP5, HAUS2, HAUS1, PCM1, HAUS8, FES, BNIP2, BBS4, SDCCAG8* |
| **brown** | **BP** | GO:0045893~positive regulation of transcription, DNA-templated | **81** | 1.29 | 0.00 | *KDM8, RSF1, CTCF, GPATCH3, BRCA2, ING2, EDRF1, SPIN1, CAPN3, BTRC, NFRKB, RNF111, CDK5RAP2, MAP3K5, IL10, PCID2, DTX3L, FBXW11, ACTR5, ACTL6A, TFEB, MSL2, AXIN1, ETV1, HNF1A, ACTR8, PARP9, ARID1A, SOX10, TGFBR1, PAX2, PPM1A, NIF3L1, ELF2, MDFIC, KAT6B, ZNF318, WAC, MAPRE3, ROR2, KDM7A, PPARD, KMT2E, BLM, INO80D, SMARCD3, ZBTB48, PDGFB, MLLT3, RAI1, HMBOX1, TASP1, HIVEP3, GPBP1L1, PCBD2, ZNF423, TFAP2A, MAP3K2, ZFHX3, AFAP1L2, NEK4, STAT1, PTCH1, ATAD2, NR1D2, GTF2H1, INHBA, ESR1, ESR2, MLLT11, IL4, CD4, NFIA, NFIB, SNAI1, REL, LHX5, QRICH1, NAA15, LGR4, PICALM* |
| **brown** | **BP** | GO:0006325~chromatin organization | 48 | 0.76 | 0.00 | *KDM5A, RB1, KDM5B, L3MBTL3, USP16, KDM1A, KMT2A, MEAF6, CHD9, KMT2C, CHD7, RNF8, CHD6, CHD5, NUCKS1, CHD4, CHD2, NR3C1, HDAC9, CHD1, HDAC7, MBTD1, ING2, ING3, NSD1, UIMC1, JAK2, BRD4, RNF20, BRD3, ZNF462, KDM4A, BRD1, KDM4C, KDM2B, SETDB1, BRPF3, ATAD2, DEK, LRWD1, TDRD3, RNF168, RBL2, DNAJC2, RBL1, WAC, EZH1, EZH2* |
| **brown** | **BP** | GO:0007368~determination of left/right symmetry | 19 | 0.30 | 0.00 | *DYNC2H1, NEK8, ACVR1, IFT140, DNAH5, PCSK6, DPCD, BICC1, ACVR2A, DISP1, IFT74, TBC1D32, DAAM2, MKS1, NPHP3, DRC1, CC2D2A, FGF10, PSKH1* |
| **brown** | **BP** | GO:0045995~regulation of embryonic development | 19 | 0.30 | 0.00 | *INO80D, LAMA2, INSR, LAMA4, ACTL6A, LAMA3, ACTR8, INO80, UCHL5, NIPBL, ZZZ3, YEATS2, RAB14, NOCT, DHX36, HESX1, TRIP12, NFRKB, NFE2L2* |
| **brown** | **BP** | GO:0042147~retrograde transport, endosome to Golgi | 23 | 0.37 | 0.00 | *SPAG9, RIC1, RHOBTB3, VPS26A, RGP1, GGA1, ARFRP1, SNX1, PIKFYVE, PREPL, SNX2, VPS51, VPS50, TBC1D5, VPS53, VTI1A, UBE2O, ANKFY1, VPS54, DENND5A, TBC1D23, EVI5, TBC1D10A* |
| **brown** | **BP** | GO:0000209~protein polyubiquitination | 27 | 0.43 | 0.00 | *CBFB, UBE3D, TNKS, MGRN1, RNF180, AMBRA1, JADE2, TNKS2, FBXO4, RNF111, SASH1, RNF20, FBXW11, SMURF1, RC3H1, UBE4B, RC3H2, DDB2, UBOX5, RNF125, MKRN2, PELI1, BCL2, ERCC8, DTL, FBXL7, BIRC2* |
| **brown** | **BP** | GO:0051497~negative regulation of stress fiber assembly | 15 | 0.24 | 0.00 | *MYOC, ARHGAP28, PIK3R1, CORO2B, TJP1, FRMD7, DLC1, RHPN2, MET, WASF2, PHLDB2, PAK2, CLASP1, CGNL1, CLASP2* |
| **brown** | **BP** | GO:0035024~negative regulation of Rho protein signal transduction | 13 | 0.21 | 0.00 | *KANK1, MYOC, ITGA3, CUL3, CCDC125, HEG1, ARHGAP35, FLCN, ARHGAP42, BCL6, DLC1, MET, SCAI* |
| **brown** | **BP** | GO:0090263~positive regulation of canonical Wnt signaling pathway | 35 | 0.56 | 0.00 | *YAP1, AMER1, KANK1, TNKS, SRC, LRRK2, LRRK1, USP34, FGF2, EGFR, SCEL, TNKS2, FGF9, TTC21B, UBR5, RSPO3, RSPO1, WLS, USP47, USP8, UBE2B, CAV1, NFKB1, PPM1A, TBL1XR1, PTK7, DAAM2, WNK2, GID8, CAPRIN2, ROR2, RECK, LGR5, LGR4, FGF10* |
| **brown** | **BP** | GO:0045737~positive regulation of cyclin-dependent protein serine/threonine kinase activity | 9 | 0.14 | 0.00 | *ADAM17, CCND3, CCNY, SPDYA, PDGFB, MAPRE3, PROX1, PKD2, EGFR* |
| **brown** | **BP** | GO:0043981~histone H4-K5 acetylation | 9 | 0.14 | 0.00 | *MEAF6, KANSL1, PHF20, KANSL2, KANSL3, JADE1, JADE3, KAT7, OGT* |
| **brown** | **BP** | GO:0071539~protein localization to centrosome | 11 | 0.17 | 0.00 | *PCM1, STIL, C2CD3, CEP350, CCDC14, KIAA0753, CEP83, CEP192, HOOK3, PIBF1, BBS4* |
| **brown** | **BP** | GO:0031398~positive regulation of protein ubiquitination | 22 | 0.35 | 0.00 | *AMER1, NDFIP2, NDFIP1, ANGPT1, RIPK2, RNF180, CUL3, CAV1, MYCBP2, WBP1L, UBE2D1, CBLB, BRCA1, BCL10, COMMD1, RCHY1, SENP2, MAPK9, SPRTN, PELI1, UBQLN1, RNF111* |
| **brown** | **BP** | GO:0071479~cellular response to ionizing radiation | 16 | 0.25 | 0.00 | *BARD1, BLM, HUS1, BRCA1, INO80, BRCA2, MAPK14, SPIDR, SIRT1, TANK, NET1, FBXO4, GRB2, INTS7, ITGB6, ECT2* |
| **brown** | **BP** | GO:0001843~neural tube closure | 30 | 0.48 | 0.00 | *IFT172, TULP3, BCL10, LRP2, ARHGAP35, HECTD1, MTHFD1L, SUFU, ABL1, IFT57, PRKACB, CC2D2A, BBS4, FZD3, APAF1, PTCH1, FZD6, MTHFR, IFT122, TSC1, SCRIB, ARID1A, PAX2, RGMA, BMP4, MTHFD1, TRAF6, RARA, MKS1, KIF20B* |
| **brown** | **BP** | GO:0001942~hair follicle development | 18 | 0.29 | 0.00 | *PIAS4, FZD3, TGFB2, FZD6, TNFRSF19, LRP4, INHBA, HOXC13, LDB2, ACVR1B, APCDD1, CELSR1, DKK1, EDAR, CD109, SOS1, LGR5, LGR4* |
| **brown** | **BP** | GO:0006275~regulation of DNA replication | 17 | 0.27 | 0.00 | *ANKRD17, USP37, INO80D, MEAF6, ESCO1, ACTR5, ACTL6A, JADE1, NUCKS1, JADE3, ACTR8, SMC3, ORC5, CCDC88A, ORC3, RBBP6, NFRKB* |
| **brown** | **BP** | GO:0032092~positive regulation of protein binding | 28 | 0.44 | 0.00 | *BLK, EPHB6, SPON1, RALB, LRP1, EPB41, NVL, LRRK2, USP33, ARRB1, IDE, ADD1, STK3, EPB41L5, KRIT1, NPHP3, EP300, CTHRC1, TCF7L2, DTX3L, RIPK2, CAV1, WNT5A, HIP1R, HIPK2, BMP4, TIAM1, CAPRIN2* |
| **brown** | **BP** | GO:0070936~protein K48-linked ubiquitination | 24 | 0.38 | 0.00 | *UBE2H, HACE1, DTX3L, UBE3C, UBE2B, CUL3, UBE2E3, UBE2D3, RNF8, UBE2D1, UBE2G1, UBE3A, KLHL3, UBE2G2, ZNRF1, ITCH, RNF216, UBE2R2, UBE2T, UBR5, TTC3, PELI1, ARIH2, BTRC* |
| **brown** | **BP** | GO:0051056~regulation of small GTPase mediated signal transduction | 13 | 0.21 | 0.00 | *RAP1GAP2, RALGAPA1, RALGAPA2, ARAP2, ARHGAP18, TSC2, SIPA1L2, ARHGAP26, ARHGAP20, RALGAPB, SIPA1L1, CHN2, GARNL3* |
| **brown** | **BP** | **GO:0000122~negative regulation of transcription from RNA polymerase II promoter** | **135** | 2.14 | 0.00 | *RB1, KDM1A, RIF1, PRDM6, WWC1, WWC2, SLA2, RBPJ, ELK4, CCND3, ZNF608, FGF9, MYB, SAP130, MAEL, EP300, EPC1, KMT5A, MYOZ1, MYOZ2, TP63, LMO1, PIAS4, MEF2C, KDM2B, ARID5B, MITF, DKK1, PIAS1, SMO, BIN1, TBL1XR1, RFX5, HOXB3, ZFPM2, NOTCH2, URI1, CBFB, CUL3, ZBTB1, RTF1, MTDH, HMBOX1, TPR, SUDS3, LYAR, SKIL, WDTC1, BPTF, TFAP2A, TFAP2B, CBX7, WWTR1, SMAD4, DR1, SMAD3, CAV1, PTCH1, PHF12, PLK1, ESR1, BMP6, ESR2, DNAJC17, IL4, YEATS2, CNOT1, CNOT2, PHF14, CPEB3, PKIG, KDM5A, THRB, TCF25, OTUD7B, FASLG, CDC73, GLI3, GLI2, FLCN, NIPBL, SUFU, JARID2, LMCD1, GABPA, NCOA2, MYOCD, ZHX2, TGIF2, EED, ARID1A, DUSP26, PARP9, SIRT1, NCOR2, NCOR1, ZEB1, RBL1, AEBP2, TRAF6, MTF2, RARA, RARB, LCOR, YAP1, TMPRSS6, SATB2, DDX20, RNF2, HDAC7, PURA, NRIP1, HSF4, BEND3, STAT6, APBB2, FNIP1, JAZF1, SPEN, SUZ12, TCF7L2, PCGF6, SEMA4D, PCGF2, MBD2, WWP2, ACVR2B, HIPK2, GATAD2A, KLF5, PER3, NR6A1, CTNNB1, TAF3, FRK* |
| **brown** | **BP** | GO:0051571~positive regulation of histone H3-K4 methylation | 11 | 0.17 | 0.00 | *SMAD4, KANSL1, KMT2A, PHF20, KANSL2, KANSL3, MYB, CTNNB1, RTF1, BRCA1, OGT* |
| **brown** | **BP** | GO:0007099~centriole replication | 11 | 0.17 | 0.00 | *PLK4, CEP135, CEP295, CEP152, KIAA0753, SASS6, POC1B, CCP110, CEP63, CEP44, CDK5RAP2* |
| **brown** | **BP** | GO:0043982~histone H4-K8 acetylation | 9 | 0.14 | 0.01 | *MEAF6, KANSL1, PHF20, KANSL2, KANSL3, JADE1, JADE3, KAT7, OGT* |
| **brown** | **BP** | GO:0042733~embryonic digit morphogenesis | 25 | 0.40 | 0.01 | *C2CD3, INTU, TRAF3IP1, LRP5, TULP3, LRP4, GLI3, GLI2, IFT52, LMBR1, TBC1D32, FLVCR1, LNPK, FBXW4, SMAD4, IFT140, WNT5A, IFT122, GRHL2, BMP4, MKS1, CTNNB1, ROR2, FREM2, BMPR1A* |
| **brown** | **BP** | GO:0070979~protein K11-linked ubiquitination | 13 | 0.21 | 0.01 | *UBE2H, AREL1, UBE2B, ANAPC7, UBE2E3, UBE2D3, CDC23, UBE2T, CDC16, CDC27, ANAPC4, ANAPC5, ANAPC1* |
| **brown** | **BP** | GO:0009411~response to UV | 16 | 0.25 | 0.01 | *ZRANB3, TIPIN, UBE2B, REV1, UBE4B, PML, PRIMPOL, MSH6, CASP9, MAPK8, CASP7, SPRTN, USP1, ERCC8, DTL, MAP4K3* |
| **brown** | **BP** | GO:0051726~regulation of cell cycle | 52 | 0.83 | 0.01 | *RB1, CCNI, JADE3, YEATS4, FGF2, ING3, CCND2, VPS72, EPC1, KAT7, EPC2, BTRC, NFRKB, NUP214, MYOG, ACTR5, ACTL6A, ACTR8, UHMK1, RBL2, RAD51D, RBL1, CCNE2, TFDP2, GAS1, PLCB1, BIRC2, USP16, INO80D, UHRF2, MEAF6, ZBTB49, CCNDBP1, MBTD1, MNT, GRK5, PES1, E2F5, SKIL, HSPA8, DR1, PNPT1, STAT3, CABLES1, SON, ZZZ3, PTPRC, YEATS2, TRIM36, MDM4, NF2, INHA* |
| **brown** | **BP** | GO:0000278~mitotic cell cycle | 18 | 0.29 | 0.01 | *PPP1R12A, NEK4, RGS14, USP3, XRCC2, PRDM5, SKA3, SKA1, RNF2, WAPL, CENPC, SKA2, LATS1, EDNRA, RECQL5, CEP250, NRDE2, AZI2* |
| **brown** | **BP** | GO:1990830~cellular response to leukemia inhibitory factor | 35 | 0.56 | 0.01 | *KDM5B, KDM3A, RIF1, PCOLCE2, PRDM5, ILDR1, MYNN, EPS8, SBNO2, BCLAF1, RNF138, PDCD10, SPOCK2, JARID2, HELLS, XRCC5, EED, SMARCA5, ARID5B, VEGFC, WDR35, SIRT1, PML, BSPRY, RNF125, MRAS, CACNB4, NR5A2, KLF5, KAT6B, PIGA, MTF2, B3GNT2, NUP35, EFHC2* |
| **brown** | **BP** | GO:0051894~positive regulation of focal adhesion assembly | 12 | 0.19 | 0.01 | *EPB41L5, NRP1, MYOC, ROCK1, KDR, ABL1, TSC1, FMN1, THY1, TEK, FERMT2, PPM1F* |
| **brown** | **BP** | GO:0007264~small GTPase mediated signal transduction | 39 | 0.62 | 0.01 | *BCAR3, DOCK4, DOCK3, DOCK9, RASGRF2, DOCK8, DOCK7, ITSN1, USO1, ARHGAP1, RHOBTB3, ARHGAP18, CHM, RND3, RASGRP1, RHOBTB1, DOCK10, RHOT1, RAC2, RALGDS, RALGPS1, RALGPS2, VAV3, RALBP1, SH2D3C, RHOH, RHOF, CCDC88A, ARHGAP31, CCDC88C, RHOJ, RAPGEF2, RAPGEF5, DOCK2, SOS1, SOS2, DOCK1, RAPGEF6, RHOQ* |
| **brown** | **BP** | GO:0016477~cell migration | 40 | 0.63 | 0.01 | *ITGB5, SRC, USP33, CUL3, WWC1, ASAP3, LAMC1, ADAMTS12, THBS1, PTPRF, LIMA1, PSTPIP2, MYO18A, ITGAV, SPDL1, PLCG1, TNS3, RHBDF1, CARMIL1, TGFB2, ZRANB1, PDPK1, USP45, SCRIB, CDC42BPB, CDC42BPA, FMNL3, CCDC88A, TIAM1, APC, PTK7, PEAK1, SNAI1, ELMO1, ELMO2, LCP1, FGFR4, AVL9, DOCK1, NOX1* |
| **brown** | **BP** | GO:0010976~positive regulation of neuron projection development | 23 | 0.37 | 0.01 | *LYN, NTRK2, PRKCI, CAMK1D, BDNF, WNT5A, NDNF, ARHGAP35, NCKIPSD, TNN, FES, PTK7, DVL1, RAPGEF1, CNTN1, ABL2, PTK2B, EP300, ITGA6, CAMK1, TOX, EPHA3, TRIM67* |
| **brown** | **BP** | GO:0006298~mismatch repair | 11 | 0.17 | 0.01 | *MSH6, MSH2, EXO1, MSH3, TDG, MSH4, MSH5, PMS2, ACYP1, MLH1, PMS1* |
| **brown** | **BP** | GO:0045740~positive regulation of DNA replication | 11 | 0.17 | 0.01 | *CTC1, BCAR3, ATAD5, BRPF3, FAF1, SMARCA5, KAT7, DNA2, BAZ1A, EGFR, GLI2* |
| **brown** | **BP** | GO:0001974~blood vessel remodeling | 14 | 0.22 | 0.01 | *ACVRL1, MEF2C, JAG1, BMPR2, SEMA3C, LIF, RBPJ, EXT1, EDNRA, FGF8, HOXA3, RSPO3, FGF10, ATG5* |
| **brown** | **BP** | GO:0000723~telomere maintenance | 14 | 0.22 | 0.01 | *PIF1, XRCC6, INO80D, XRCC5, ACTL6A, ACTR8, INO80, TERF1, UCHL5, RAD50, POT1, ATM, NFRKB, ZNF365* |
| **brown** | **BP** | GO:0045022~early endosome to late endosome transport | 10 | 0.16 | 0.01 | *ANKRD27, WDR81, STX8, WDR91, LMTK2, EMP2, HOOK3, SNF8, ATG14, RAB5A* |
| **brown** | **BP** | GO:0030155~regulation of cell adhesion | 16 | 0.25 | 0.01 | *PPP1R12A, YTHDF2, LAMA2, FAF1, LAMA4, LAMA3, TNC, CXCR4, LMO7, PML, PPP1CB, NUAK1, EMCN, TNN, FES, PTK2B* |
| **brown** | **BP** | GO:0051496~positive regulation of stress fiber assembly | 18 | 0.29 | 0.01 | *NRP1, CARMIL1, MYOC, ROCK2, GPR65, LPAR1, BRAF, PPM1E, PPM1F, MTOR, TGFBR1, CCDC88A, BAG4, ABL1, EVL, NF2, FERMT2, ARHGEF5* |
| **brown** | **BP** | GO:0072205~metanephric collecting duct development | 6 | 0.10 | 0.01 | *BMP4, CALB1, PTCH1, DLG5, AQP2, PAX2* |
| **brown** | **BP** | GO:0060831~smoothened signaling pathway involved in dorsal/ventral neural tube patterning | 6 | 0.10 | 0.01 | *TBC1D32, PTCH1, IFT122, TULP3, GLI3, GLI2* |
| **brown** | **BP** | GO:0006301~postreplication repair | 6 | 0.10 | 0.01 | *UBE2B, MSH2, POLI, BRCA1, RAD18, POLH* |
| **brown** | **BP** | GO:0051057~positive regulation of small GTPase mediated signal transduction | 6 | 0.10 | 0.01 | *RELN, FRMD7, SRC, BNIP2, ITGAV, CDON* |
| **brown** | **BP** | GO:0045910~negative regulation of DNA recombination | 6 | 0.10 | 0.01 | *ZRANB3, MSH6, RTEL1, BLM, MSH2, MSH3* |
| **brown** | **BP** | GO:0001953~negative regulation of cell-matrix adhesion | 8 | 0.13 | 0.01 | *ACER2, JAG1, MYOC, BCL6, RASA1, SEMA3E, PIK3R1, THBS1* |
| **brown** | **BP** | GO:0030210~heparin biosynthetic process | 8 | 0.13 | 0.01 | *NDST2, EXT1, EXT2, NDST1, SLC10A7, ANGPT1, GLCE, XYLT2* |
| **brown** | **BP** | GO:0034446~substrate adhesion-dependent cell spreading | 17 | 0.27 | 0.01 | *NRP1, ITGA4, SRC, FN1, LAMB1, PARVA, LAMC1, MERTK, SRCIN1, FER, PEAK1, AKIP1, TYRO3, ABL1, ITGA8, ITGAV, TEK* |
| **brown** | **BP** | GO:0050891~multicellular organismal water homeostasis | 7 | 0.11 | 0.01 | *EXT1, EXT2, SCNN1G, SCNN1B, SCNN1A, AQP4, CFTR* |
| **brown** | **BP** | GO:0010457~centriole-centriole cohesion | 7 | 0.11 | 0.01 | *CEP68, NIN, CEP135, ODF2, CEP250, CEP44, CNTLN* |
| **brown** | **BP** | GO:0007229~integrin-mediated signaling pathway | 37 | 0.59 | 0.01 | *NRP1, SEMA7A, ITGB5, ITGB4, SRC, CUL3, ITGA2B, PLEK, ITGB2, THY1, GPATCH8, PRAM1, ABL1, PTK2B, ITGB8, ITGAV, ITGB6, ADAMTS9, VAV3, SYK, ITGA4, ITGA3, ITGA2, ITGA1, FN1, FERMT1, ADAM17, CD40LG, PTPRA, ITGA11, ITGA8, ADAM9, ITGB1BP1, ITGA6, ITGB1BP2, FERMT2, ITGA9* |
| **brown** | **BP** | GO:0001822~kidney development | 27 | 0.43 | 0.01 | *TRAF3IP2, TRAF3IP1, LRP4, LRP2, GLI2, PKHD1, ADAMTS1, TBC1D32, CC2D2A, ADAMTS6, DYNC2H1, TFAP2A, HELLS, PTCD2, C1GALT1, WNT5A, DTNBP1, ARID5B, TSC1, PROX1, BICC1, TGFBR1, BMP6, PYGO1, PTK7, ITGA8, FREM2* |
| **brown** | **BP** | GO:0007155~cell adhesion | 58 | 0.92 | 0.01 | *SPON1, B4GALT1, TNFAIP6, ITGB5, TNC, AATF, HAPLN4, HAPLN1, GPATCH8, CHL1, NPTN, ITGAV, NRCAM, EMB, TINAG, EPHA4, ITGA3, PRKCE, ITGA2, ATP1B1, RGMB, VCAN, NINJ2, MXRA8, ITGA6, TLN2, VCL, FREM2, ITGA9, LAMA2, LAMA4, LAMA3, NEDD9, NEXN, THY1, PSEN1, THBS1, TM9SF4, DPP4, EFNB2, ACAN, RELN, NCAM1, SUSD5, NCAM2, CTNNAL1, MPDZ, LMLN, FARP2, FN1, GRHL2, FERMT1, KITLG, CD4, COL7A1, ITGA11, CTNNB1, FERMT2* |
| **brown** | **BP** | GO:0051865~protein autoubiquitination | 25 | 0.40 | 0.01 | *RNF11, UHRF2, UBE3D, CUL3, UBE2D3, RNF8, LTN1, JADE2, BRCA1, HECTD1, DTX3L, UBE2B, UBE4B, WWP2, DDB2, RNF146, CNOT4, SH3RF2, ITCH, NFX1, UBE2T, TRAF6, TRIM13, ERCC8, RAD18* |
| **brown** | **BP** | GO:0000724~double-strand break repair via homologous recombination | 28 | 0.44 | 0.01 | *EXD2, BLM, WDR48, PSMD14, MCM8, MCM9, SMC5, NUCKS1, BRCA1, BRCA2, RAD54B, ZFYVE26, RNF138, RBBP8, POLL, HELQ, RAD21L1, XRCC2, NSMCE2, INO80, SPIDR, PALB2, RAD51B, MMS22L, RAD51D, RAD51C, ATM, FAN1* |
| **brown** | **BP** | GO:0031623~receptor internalization | 14 | 0.22 | 0.01 | *GRIA1, CALCRL, TFRC, SYK, CAV1, ITGB2, SNX1, GRK2, GRK4, NEDD4, CNTN2, GRB2, LDLRAP1, PICALM* |
| **brown** | **BP** | GO:0031297~replication fork processing | 14 | 0.22 | 0.01 | *ZRANB3, SMARCAL1, EXD2, RTEL1, BLM, FANCM, ATRX, BOD1L1, THOC1, NUCKS1, PRIMPOL, ETAA1, MMS22L, WRN* |
| **brown** | **BP** | GO:0010506~regulation of autophagy | 18 | 0.29 | 0.02 | *USP13, UVRAG, MTCL1, USP10, USP33, WDR41, XPA, SOGA1, GPR137B, ACER2, MFSD8, DRAM1, TP53INP1, ATM, MAPT, FBXL2, DRAM2, RAB8A* |
| **brown** | **BP** | GO:0007411~axon guidance | 33 | 0.52 | 0.02 | *CSF1R, NRP1, SEMA3C, LAMA2, USP33, NEXN, GLI3, ARHGAP35, GLI2, CDH4, RELN, FLRT2, DPYSL5, CHL1, DVL1, BOC, NPTN, NRCAM, APBB2, EMB, NEO1, MYH10, LGI1, SMAD4, BDNF, ETV1, MAPK8IP3, EXT1, TTC8, LMX1A, CNTN2, B3GNT2, MATN2* |
| **brown** | **BP** | GO:0007160~cell-matrix adhesion | 24 | 0.38 | 0.02 | *CD96, ITGB5, ITGB4, ITGA2B, PXN, ITGB2, EMP2, TSC1, NPNT, ADAMTS12, NID2, TIAM1, MKLN1, BCL2L11, TNN, ITGA8, CTNNB1, ITGB8, ADAM9, ITGAV, ITGB1BP1, ITGA6, ITGB6, HPSE* |
| **brown** | **BP** | GO:0014068~positive regulation of phosphatidylinositol 3-kinase signaling | 24 | 0.38 | 0.02 | *PDGFRB, TGFB2, MYOC, ANGPT1, SEMA4D, UNC5B, HGF, FN1, PDGFB, PRR5L, CBL, SIRT1, FGF2, SELP, RELN, FSHR, NEDD4, CAT, KDR, CD28, PLXNB1, TEK, PIK3AP1, PPARD* |
| **brown** | **BP** | GO:0060348~bone development | 17 | 0.27 | 0.02 | *AMER1, SLC10A7, MIGA2, MYOC, NPR2, ANKRD11, IFT172, TULP3, SLC38A10, MAPK14, SMAD5, FGF8, SH3PXD2B, ITGB6, NOTμm, TMEM38B, TRIM45* |
| **brown** | **BP** | GO:0002062~chondrocyte differentiation | 15 | 0.24 | 0.02 | *RB1, MEF2C, WNT2B, PTH1R, ADAMTS12, MAPK14, GLI2, BMP4, COL2A1, SNX19, FGF9, NFIB, CREB3L2, MEF2D, BMPR1A* |
| **brown** | **BP** | GO:1901998~toxin transport | 15 | 0.24 | 0.02 | *COPB2, CD274, NRP1, MTMR12, TRIP4, ADAM10, ANTXR2, ARID1A, DNM1, DNAJC17, GGA1, RAB40B, VPS11, RAB28, ATP6V0A1* |
| **brown** | **BP** | GO:0043123~positive regulation of I-kappaB kinase/NF-kappaB signaling | 40 | 0.63 | 0.02 | *ANKRD17, TRAF3IP2, DDX1, LPAR1, NOD1, FASLG, BCL10, MTDH, IKBKB, TRIM8, TBK1, DHX36, TNFSF10, ABL1, RIPK1, IKBKE, BRD4, MAP3K3, PRKCB, RIPK2, PRKCE, TNFRSF19, IRAK4, MID2, CD4, TRAF6, TRAF5, TRIM13, PELI1, TAB3, CTNNB1, TAB2, ROR1, ALPK1, MAP3K14, MYD88, TLR3, CARD11, TMEM106A, BIRC2* |
| **brown** | **BP** | GO:0016567~protein ubiquitination | 52 | 0.83 | 0.02 | *TRAF3IP1, FBXO25, ARRB1, SH3RF1, RNF114, ATG3, RNF43, WSB1, WSB2, FBXW7, SIRT1, RNF125, GAN, UHRF2, CUL5, KLHL15, CUL3, CUL2, KLHL17, CUL1, TULP4, ASB11, VCPIP1, DCAF17, RAB40B, CAND1, RAB40C, SOCS6, DCAF13, SOCS4, SOCS5, WWTR1, CRBN, SIAH2, PLK1, SIAH1, KLHL2, FBXO32, ZZEF1, SPSB3, SPSB1, KLHL7, KLHL8, RNF182, ASB7, TRIM37, ASB2, FBXL5, ASB3, TRIM33, CUL4B, NFE2L2* |
| **brown** | **BP** | GO:0001764~neuron migration | 33 | 0.52 | 0.02 | *BARHL1, GPM6A, TOP2B, TUBGCP2, SATB2, CEP85L, CELSR1, PCM1, RELN, UNK, CHL1, ALKBH1, APBB2, DISC1, CCR4, MYH10, KIAA0319, PRKG1, BBS4, NDEL1, KIRREL3, NTRK2, FZD3, MEF2C, ITGA3, USP9X, ELP3, ACAP3, PEX7, CTNNB1, LRP12, SDCCAG8, MATN2* |
| **brown** | **BP** | GO:0009410~response to xenobiotic stimulus | 26 | 0.41 | 0.02 | *TRAF3IP2, SLC1A1, PTPRM, SLC1A3, GATA4, HTR2A, THBS1, RAD54B, GRIN2A, ATG5, VAV3, SS18, USP47, TIGAR, TGFB2, SLC12A5, TRPA1, UBE2B, BDNF, INHBA, SRP68, TGFBR2, P2RX7, SCNN1B, PDE3A, BCL2* |
| **brown** | **BP** | GO:0007162~negative regulation of cell adhesion | 12 | 0.19 | 0.02 | *EPHA4, ANGPT1, SEMA4D, PDE3B, CDH13, LPXN, ADAM10, PLXNB1, EPHB2, CBLL1, KNG1, JAM2* |
| **brown** | **BP** | GO:0045190~isotype switching | 9 | 0.14 | 0.02 | *MSH6, CD40LG, ATAD5, MSH2, EXO1, SWAP70, NBN, EXOSC3, BATF* |
| **brown** | **BP** | GO:0000245~spliceosomal complex assembly | 9 | 0.14 | 0.02 | *SRPK2, ZRSR2, GCFC2, NCBP1, SCAF11, GEMIN6, CRNKL1, SF3B1, RBM5* |
| **brown** | **BP** | GO:0043473~pigmentation | 9 | 0.14 | 0.02 | *EDAR, VANGL1, FIG4, ATRN, SZT2, VPS33A, HPS5, LYST, ARCN1* |
| **brown** | **BP** | GO:0070534~protein K63-linked ubiquitination | 14 | 0.22 | 0.02 | *TRAF3IP2, UBE2B, UBE2E3, RNF8, UBE2G1, WWP2, ITCH, HECTD1, UBE2T, TRAF6, PELI1, UBE2N, ARIH2, UBE2O* |
| **brown** | **BP** | GO:0007507~heart development | 35 | 0.56 | 0.02 | *CDKN1B, CALCRL, TRAF3IP2, DNAH5, PDGFB, GATA4, GLI3, GLI2, IFT74, ACAN, PPP3CB, CASP7, PDLIM3, GRK2, TBC1D32, SH3PXD2B, EP300, SMYD4, CC2D2A, MAP2K5, PSKH1, NEK8, TGFB2, IFT140, SENP2, BICC1, TGFBR1, MTHFD1, LOX, RBM20, TAB2, ATM, DRC1, FREM2, FBN1* |
| **brown** | **BP** | GO:0031175~neuron projection development | 24 | 0.38 | 0.02 | *RB1, LYN, GSK3B, MYOC, STMN3, UBE4B, LAMB1, GDPD5, CAMSAP1, RGMA, UHMK1, IL6, HERC1, VAPA, CLMN, FSHR, ALKBH1, NEDD4, MICALL1, CNTN1, ERCC6, ATXN10, TBC1D23, LRP12* |
| **brown** | **BP** | GO:0007224~smoothened signaling pathway | 22 | 0.35 | 0.02 | *EVC2, IFT172, PKD2L1, IFT80, HIPK1, TGFBR2, HIPK2, IFT52, EVC, SMO, PTCHD1, ARL13B, KIAA0586, CENPJ, TTC21B, BOC, ULK3, DZIP1L, ROR2, IFT57, CC2D2A, CDON* |
| **brown** | **BP** | GO:2000353~positive regulation of endothelial cell apoptotic process | 8 | 0.13 | 0.03 | *COL18A1, PRKCI, CD40LG, ITGA4, FASLG, ANO6, PLCG1, THBS1* |
| **brown** | **BP** | GO:0010165~response to X-ray | 8 | 0.13 | 0.03 | *BLM, XRCC4, MSH2, XRCC2, XRRA1, ERCC8, ERCC6, BRCA2* |
| **brown** | **BP** | GO:0097320~plasma membrane tubulation | 8 | 0.13 | 0.03 | *BIN2, MICALL1, PACSIN2, SNX9, SNX33, WHAMM, FNBP1L, PACSIN1* |
| **brown** | **BP** | GO:0003148~outflow tract septμm morphogenesis | 11 | 0.17 | 0.03 | *BMP4, NRP1, SMAD4, FGF8, BMPR2, SEMA3C, PARVA, LRP2, ZFPM2, TGFBR2, BMPR1A* |
| **brown** | **BP** | GO:0061512~protein localization to ciliμm | 11 | 0.17 | 0.03 | *DYNC2H1, FAM149B1, IFT140, TBC1D32, ARL6, DZIP1L, IFT122, TULP3, ZNF423, WDR35, RAB8A* |
| **brown** | **BP** | GO:0042149~cellular response to glucose starvation | 15 | 0.24 | 0.03 | *SZT2, MTMR3, PRKAA2, EIF2AK3, ASNS, ATG14, SIRT1, ITFG2, RRP8, TBL2, NUAK2, SESN1, ZC3H12A, BCL2, NFE2L2* |
| **brown** | **BP** | GO:0043010~camera-type eye development | 15 | 0.24 | 0.03 | *FBN2, MITF, GRHL2, PRSS56, ARHGAP35, PAX2, EFEMP1, GPD2, HSF4, HESX1, PKNOX1, CC2D2A, SLC25A25, RAB3GAP1, FBN1* |
| **brown** | **BP** | GO:0030032~lamellipodiμm assembly | 13 | 0.21 | 0.03 | *VAV3, CYFIP1, CARMIL1, CCDC88A, GOLPH3, ABLIM3, NUP85, KIT, CDH13, WHAMM, WASF2, WASF3, VCL* |
| **brown** | **BP** | GO:0048812~neuron projection morphogenesis | 13 | 0.21 | 0.03 | *KIRREL3, GPM6A, ANKRD27, MAP1S, LRRK2, SHTN1, EPB41L3, ZSWIM6, PLXNB1, NEK3, KIF20B, SRGAP2, ZNF335* |
| **brown** | **BP** | GO:0030335~positive regulation of cell migration | 48 | 0.76 | 0.03 | *GRB7, COL18A1, ATP8A1, LEF1, NEDD9, LAMC2, PIK3R1, CLDN1, EGFR, IGF1R, PPP3CA, PDCD10, MGAT5, NTF3, ITGAV, CPNE3, EPHB2, SPAG9, ACVR1, LYN, EPHA4, PDGFRA, CARMIL1, CARMIL2, MYOC, SEMA4D, HGF, CAV1, INSR, ARHGEF39, RRAS2, LAMB1, ELP3, TGFBR1, IL4, SH3RF2, TIAM1, FER, SMO, APC, RUFY3, SNAI1, NμmB, CDH13, ATM, ITGA6, ROR2, CBLL1* |
| **brown** | **BP** | GO:0016485~protein processing | 18 | 0.29 | 0.03 | *DYNC2H1, C2CD3, PTCH1, IFT172, TMPRSS4, ADAM10, PSEN1, PCSK6, CTSS, GLI3, P2RX7, ADAM17, CASP7, ADAMTS3, MMP16, XPNPEP3, LONP2, OGT* |
| **brown** | **BP** | GO:0006888~ER to Golgi vesicle-mediated transport | 18 | 0.29 | 0.03 | *STX17, COG3, GOSR1, ANK1, LMAN1, SPAST, ZW10, CREB3L2, TRAPPC12, TRAPPC11, WHAMM, SEC24C, SEC22A, CNIH4, SEC31B, ERGIC1, SEC22C, SEC31A* |
| **brown** | **BP** | GO:0034622~cellular macromolecular complex assembly | 7 | 0.11 | 0.03 | *TIAM1, RIC3, NEMF, MBD2, AXIN1, DNAJB14, ANKS4B* |
| **brown** | **BP** | GO:0006622~protein targeting to lysosome | 7 | 0.11 | 0.03 | *ZFYVE16, GGA3, SORT1, NEDD4, AP3B1, GCC2, SORL1* |
| **brown** | **BP** | GO:0003139~secondary heart field specification | 7 | 0.11 | 0.03 | *BMP4, MEF2C, SMARCD3, WNT5A, AXIN2, LRP2, RBPJ* |
| **brown** | **BP** | GO:0000290~deadenylation-dependent decapping of nuclear-transcribed mRNA | 7 | 0.11 | 0.03 | *PAN3, NOCT, DCP1A, DCPS, PATL1, DCP2, DCP1B* |
| **brown** | **BP** | GO:0010761~fibroblast migration | 7 | 0.11 | 0.03 | *TMEM201, ZFAND5, PIP5K1A, ARID5B, IQGAP1, PML, PLEC* |
| **brown** | **BP** | GO:0050727~regulation of inflammatory response | 21 | 0.33 | 0.03 | *LYN, SEMA7A, AREL1, SETD6, TNC, STK39, ADAMTS12, ESR1, ABHD12, LACC1, SBNO2, TNIP1, BCL6, SHPK, RICTOR, TBC1D23, JAK2, DAGLB, PIK3AP1, MYD88, BRD4* |
| **brown** | **BP** | GO:0043124~negative regulation of I-kappaB kinase/NF-kappaB signaling | 16 | 0.25 | 0.03 | *MAPKBP1, NLRX1, STAT1, RIOK3, USP10, RHOH, OTUD7B, SIRT1, ESR1, TANK, PPM1A, TNIP1, ZC3H12A, ABL1, RIPK1, NFKBID* |
| **brown** | **BP** | GO:0048589~developmental growth | 10 | 0.16 | 0.03 | *NIPBL, SMAD4, SMAD3, ZMIZ1, ALKBH1, KDF1, SOX10, GLI3, BMPR1A, GLI2* |
| **brown** | **BP** | GO:0009887~animal organ morphogenesis | 10 | 0.16 | 0.03 | *NEK8, NRP1, SMARCC1, GMNN, VEGFC, EP300, LHX4, E2F5, PALB2, FLI1* |
| **brown** | **BP** | GO:0097150~neuronal stem cell population maintenance | 10 | 0.16 | 0.03 | *SS18, PCM1, MMP24, JAG1, CDH2, FUT10, FANCC, PROX1, HOOK3, FOXO3* |
| **brown** | **BP** | GO:0060070~canonical Wnt signaling pathway | 19 | 0.30 | 0.03 | *TCF7L1, FZD3, WNT2B, WNT3A, LEF1, WNT5A, LRP5, DIXDC1, LRP6, PYGO1, EXT1, BCL9, EDNRA, STK11, DVL1, HESX1, DVL3, PLPP3, BCL9L* |
| **brown** | **BP** | GO:0045747~positive regulation of Notch signaling pathway | 14 | 0.22 | 0.03 | *YAP1, TGFB2, POGLUT1, JAG1, SRC, STAT3, EPN2, PDCD10, ZMIZ1, ITGB1BP1, IL6ST, NEPRO, TP63, FGF10* |
| **brown** | **BP** | GO:0032733~positive regulation of interleukin-10 production | 14 | 0.22 | 0.03 | *SASH3, CD274, CD83, SYK, HGF, IL20RB, IL4, IL6, CD40LG, IRF4, PLCG2, CD28, CD34, PIBF1* |
| **brown** | **BP** | GO:0070986~left/right axis specification | 6 | 0.10 | 0.03 | *NOTCH2, EPB41L5, SMO, ARL13B, IFT172, MNS1* |
| **brown** | **BP** | GO:0090091~positive regulation of extracellular matrix disassembly | 6 | 0.10 | 0.03 | *CARMIL2, IL6, MELTF, CLASP1, CLASP2, DDR2* |
| **brown** | **BP** | GO:0043984~histone H4-K16 acetylation | 6 | 0.10 | 0.03 | *KANSL1, PHF20, KANSL2, KANSL3, MSL2, OGT* |
| **brown** | **BP** | GO:0035137~hindlimb morphogenesis | 6 | 0.10 | 0.03 | *TFAP2B, PTCH1, FMN1, GDF5, HOXD10, BMPR1A* |
| **brown** | **BP** | **GO:0043066~negative regulation of apoptotic proces**s | **78** | 1.24 | 0.03 | *ANKLE2, CDKN1B, ADAR, NR2E1, GLI3, GLI2, IGF1R, DSTYK, MAEL, MYO18A, CAPN3, KIFAP3, PCID2, AREL1, CAMK1D, DAPK1, HGF, TNFRSF18, WNT5A, SOX10, DKK1, PAX2, ADAMTS20, DNAJC3, BTC, SUPV3L1, ARHGAP10, SMO, RASA1, CAT, YME1L1, BIRC2, STK40, FAIM, CSF1R, PTGFR, PRKAA2, BCL2A1, PRKDC, PIK3R1, LRP2, CBL, FSTL1, EGFR, NUGGC, HSP90B1, MTDH, MAPK8, NUAK2, GRK5, PDCD10, PTK2B, APBB2, ATG5, BARD1, TFAP2A, UBE2B, PLK1, HIP1R, ASNS, IGF1, PALB2, HIPK3, KLHL20, SH3RF2, IL6, GOLPH3, ITCH, CD40LG, SON, BCL6, IL2RB, BCL2, NAA16, MDM4, GHRL, LHX4, NAA15* |
| **brown** | **BP** | **GO:0008284~positive regulation of cell proliferation** | **79** | 1.25 | 0.03 | *KLB, COL18A1, CDKN1B, CDCA7L, TNC, ASH2L, LAMC2, PTH1R, IGF1R, FGF8, HOXA3, TNS3, EPHA4, MYOCD, TIPIN, TET1, ADAM10, EMP2, GAB2, AVPR1A, BTC, TIAM1, GAREM1, KIT, RARA, ITGB1BP1, BIRC6, KIF20B, CACUL1, FXN, SHC4, PTGFR, PDGFB, GNAI2, DPP4, EFNB2, PURA, GRK5, PDCD10, NTF3, PDGFC, HSF4, PTK2B, E2F3, RPRD1B, SRPK2, SUZ12, TFAP2B, WWTR1, TGFB2, KDM4C, ST8SIA1, STAT1, INSR, IL34, LIF, IL31RA, CDC7, IGF1, HIPK1, HIPK2, ACER2, IL4, GDF9, FER, CNOT6, KITLG, CCPG1, PRC1, GHRH, ACER3, GID8, SPDYA, AQP11, FAM98B, FGFR4, SLC25A33, FGFR2, NOX1* |
| **brown** | **BP** | GO:0072307~regulation of metanephric nephron tubule epithelial cell differentiation | 5 | 0.08 | 0.03 | *YAP1, WWTR1, LIF, FAT4, PAX2* |
| **brown** | **BP** | GO:0010706~ganglioside biosynthetic process via lactosylceramide | 5 | 0.08 | 0.03 | *B4GALT6, ST3GAL1, ST3GAL2, B4GALT5, ST3GAL3* |
| **brown** | **BP** | GO:1990116~ribosome-associated ubiquitin-dependent protein catabolic process | 5 | 0.08 | 0.03 | *NEMF, LTN1, TRIP4, ASCC2, ASCC3* |
| **brown** | **BP** | GO:0051224~negative regulation of protein transport | 5 | 0.08 | 0.03 | *SNX3, NDFIP2, NDFIP1, WWP2, PPM1F* |
| **brown** | **BP** | GO:0051574~positive regulation of histone H3-K9 methylation | 5 | 0.08 | 0.03 | *RIF1, MYB, BRCA1, JARID2, SIRT1* |
| **brown** | **BP** | GO:0031648~protein destabilization | 17 | 0.27 | 0.03 | *FBXW11, SRC, FBXW7, CUL3, KDM8, PLK1, HTT, SENP2, SIRT1, CDC73, GGA3, CCDC88C, FBXO4, CAPN3, EP300, FBXL3, BTRC* |
| **brown** | **BP** | GO:0042981~regulation of apoptotic process | 37 | 0.59 | 0.04 | *RBM25, DDX42, LRP5, SRA1, NOD1, IKZF3, MALT1, BCL2L13, ING2, WRN, CASP8, SLK, RASSF3, RASSF5, NTF3, RASSF6, TRIM24, HS1BP3, TNFAIP8L3, IFT57, LCMT1, RBM5, BCL2L14, BCL2L15, KPNA1, TOMM70, CFLAR, ESR1, ACER2, LOX, TRAF3, DLG5, TRAF6, TRAF5, NF2, CARD11, BCL2L1* |
| **brown** | **BP** | GO:0048286~lung alveolus development | 15 | 0.24 | 0.04 | *MYOCD, BMPR2, PKDCC, LIF, ABCA12, MAPK8IP3, RC3H2, BMP4, MAN1A2, MAN2A1, PHF14, ITGB6, TMEM38B, TNS3, STK40* |
| **brown** | **BP** | GO:0000281~mitotic cytokinesis | 15 | 0.24 | 0.04 | *USP8, PLK1, SNX33, CIT, ANLN, ZFYVE26, SPAST, SON, APC, TRIM36, SNX9, ECT2, MYH10, MAP9, BBS4* |
| **brown** | **BP** | GO:0010608~posttranscriptional regulation of gene expression | 9 | 0.14 | 0.04 | *EPB41L5, YTHDC1, CAV1, FBXO4, TDRD7, PARP9, ATG14, METTL16, RC3H2* |
| **brown** | **BP** | GO:0001818~negative regulation of cytokine production | 9 | 0.14 | 0.04 | *PTGER4, RNF128, NPTN, BTK, ATP2B1, MERTK, LRRC32, LGR4, N4BP1* |
| **brown** | **BP** | GO:0032148~activation of protein kinase B activity | 9 | 0.14 | 0.04 | *OSBPL8, CCDC88A, PDPK1, INSR, WNT5A, SLC1A1, NTF3, PDGFB, ITGB1BP1* |
| **brown** | **BP** | GO:0042110~T cell activation | 9 | 0.14 | 0.04 | *IL4, DPP4, SMAD3, CD8A, NEDD4, CBLB, SLA2, AZI2, CD44* |
| **brown** | **BP** | GO:0072583~clathrin-dependent endocytosis | 9 | 0.14 | 0.04 | *GAK, LMBRD1, INPP5F, SGIP1, FCHO2, CANX, GPR107, FNBP1L, PICALM* |
| **brown** | **BP** | GO:0002092~positive regulation of receptor internalization | 9 | 0.14 | 0.04 | *ATAD1, ANGPT1, SYK, TBC1D5, FMR1, INSR, NTF3, PLCG2, ARRB1* |
| **brown** | **BP** | GO:0002052~positive regulation of neuroblast proliferation | 9 | 0.14 | 0.04 | *FZD3, SMARCD3, SMO, KDM1A, CTNNB1, DISC1, ZNF335, SOX10, GLI3* |
| **brown** | **BP** | GO:0043536~positive regulation of blood vessel endothelial cell migration | 13 | 0.21 | 0.04 | *NUS1, ANGPT1, PDPK1, PDGFB, PRKCA, THBS1, SIRT1, FGF2, ETS1, ADAM17, P2RX4, AMOTL1, PLCG1* |
| **brown** | **BP** | GO:0046856~phosphatidylinositol dephosphorylation | 13 | 0.21 | 0.04 | *MTMR2, MTMR3, PTEN, OCRL, SYNJ2, MTMR7, MTM1, INPP5B, FIG4, INPP5A, SYNJ1, INPP5F, INPP5D* |
| **brown** | **BP** | GO:0070507~regulation of microtubule cytoskeleton organization | 13 | 0.21 | 0.04 | *PHLDB1, PRKAA2, ROCK1, TRAF3IP1, DIXDC1, EFNA5, BICD1, BICD2, KIF18A, CEP70, TRIM36, PHLDB2, EPHA3* |
| **brown** | **BP** | GO:0002040~sprouting angiogenesis | 11 | 0.17 | 0.04 | *ANGPT1, LEF1, OTULIN, CDH13, RSPO3, PTK2B, SEMA3E, PARVA, TEK, VSTM4, THBS1* |
| **brown** | **BP** | GO:1902476~chloride transmembrane transport | 11 | 0.17 | 0.04 | *GABRB2, GLRA1, ANO1, GLRA2, GLRA3, GLRB, SLC1A3, ANO6, CLCN3, CFTR, GABRG2* |
| **brown** | **BP** | GO:0031146~SCF-dependent proteasomal ubiquitin-dependent protein catabolic process | 11 | 0.17 | 0.04 | *WWTR1, FBXW11, FBXW7, FBXO38, FBXO4, FBXL3, FBXL2, BTRC, FBXL5, FBXO33, FBXL7* |
| **brown** | **BP** | GO:0048514~blood vessel morphogenesis | 8 | 0.13 | 0.05 | *SYK, CDH2, LOX, RASA1, NEDD4, SLC1A1, SOS1, FGF10* |
| **brown** | **BP** | GO:2000114~regulation of establishment of cell polarity | 8 | 0.13 | 0.05 | *ARFGEF1, SHTN1, KANK1, BCAS3, KRIT1, PTK2B, RICTOR, KIF20B* |
| **brown** | **BP** | GO:0001702~gastrulation with mouth forming second | 8 | 0.13 | 0.05 | *ACVR1, SMAD4, TENM4, LRP5, CTNNB1, PLPP3, RNF2, ACVR2A* |
| **brown** | **BP** | GO:2000251~positive regulation of actin cytoskeleton reorganization | 8 | 0.13 | 0.05 | *BCAS3, NRP1, BAIAP2L1, FES, NTF3, ABL1, STAP1, TEK* |
| **brown** | **BP** | GO:0033365~protein localization to organelle | 8 | 0.13 | 0.05 | *BBS2, CEP68, GRXCR2, PES1, COG3, CEP250, BICD1, CNTLN* |
| **brown** | **BP** | GO:1902018~negative regulation of ciliμm assembly | 8 | 0.13 | 0.05 | *YAP1, ODF2L, MPHOSPH9, KIF24, CDK10, CCP110, EVI5L, CEP97* |
| **brown** | **BP** | GO:0010842~retina layer formation | 8 | 0.13 | 0.05 | *TFAP2A, TFAP2B, CALB1, SLC1A1, PTPRM, HIPK1, SDK2, HIPK2* |
| **brown** | **BP** | GO:0050853~B cell receptor signaling pathway | 14 | 0.22 | 0.05 | *VAV3, LYN, BLK, MEF2C, SYK, PRKCB, PTPRC, PLCG2, BLNK, BCL2, ABL1, BTK, CTLA4, MAPK1* |
| **brown** | **BP** | GO:0032743~positive regulation of interleukin-2 production | 14 | 0.22 | 0.05 | *SASH3, CD83, RIPK2, PDE4D, CD3E, MALT1, PTPRC, IRF4, TRAF6, PLCG2, PDE4B, ABL1, CD28, CARD11* |
| **brown** | **BP** | GO:0001933~negative regulation of protein phosphorylation | 17 | 0.27 | 0.05 | *PID1, ANGPT1, TRAF3IP1, LRRK2, CTDSPL, PRR5L, ATG14, PTPN13, KLHL31, SPRED2, RABGEF1, SPRED1, CTDSP1, CD109, DNAJC10, ZC3H12A, PLPP3* |
| **brown** | **BP** | GO:0001947~heart looping | 17 | 0.27 | 0.05 | *NOTCH2, MEF2C, SMAD3, C2CD3, DNAAF1, IFT172, WNT5A, PKD2, TGFBR2, IFT52, FGF8, SMO, ARL13B, SUFU, HAND1, ASB2, IFT57* |
| **brown** | **BP** | GO:0060045~positive regulation of cardiac muscle cell proliferation | 10 | 0.16 | 0.05 | *YAP1, MEF2C, FGF9, HEY2, RBPJ, MAPK14, ZFPM2, WNT2, FGF2, BMPR1A* |
| **brown** | **BP** | GO:0070372~regulation of ERK1 and ERK2 cascade | 10 | 0.16 | 0.05 | *PKHD1, TRAF7, EPHA7, SYK, STYX, FN1, NEK10, ZDHHC17, YWHAZ, ROS1* |
| **brown** | **BP** | GO:0060324~face development | 10 | 0.16 | 0.05 | *EDNRA, ZFAND5, WNT5A, RARA, HOXB3, MAPK1, BRAF, RAF1, GRHL2, BBS4* |
| **brown** | **BP** | GO:0048854~brain morphogenesis | 10 | 0.16 | 0.05 | *BBS2, AFDN, VPS51, CDH2, AKT3, FANCC, ZNF335, FOXO3, BBS4, PAX2* |
| **brown** | **BP** | GO:0032880~regulation of protein localization | 21 | 0.33 | 0.05 | *USP8, RABGAP1L, PARP1, KDM1A, SYCP1, SWAP70, PTCH1, WNT5A, MYCBP2, WASL, UFL1, MAPK8, TTC8, RAB14, MAP2, DVL1, DZIP1L, BCL2, PLEKHM2, DVL3, EPS15* |
| **brown** | **BP** | GO:0006338~chromatin remodeling | 21 | 0.33 | 0.05 | *PBRM1, SATB1, SATB2, MBD2, ZBTB1, HNF1A, CHD1L, ACTR8, INO80, TTF1, BAZ1B, ARID1B, SMARCA2, GATAD2A, KAT2B, MIER1, MYB, SCMH1, MORC2, TP63, BPTF* |
| **brown** | **CC** | **GO:0005829~cytosol** | **939** | 14.90 | 0.00 | *ZFYVE9, ALKBH8, ALKBH3, PREX1, ANKFY1, WLS, ATG3, RBFOX2, GAPVD1, EML1, SND1, ZFYVE16, MTHFD1, RUFY3, PSME4, ORAI1, SKAP2, URI1, MTMR2, ABCB6, GIGYF2, MTMR7, EPB41L5, RNF216, AFMID, VTI1A, PIP5K1A, PIP5K1B, LTA4H, PSMF1, PPARGC1B, CTC1, PMM2, SNF8, FLI1, MLLT11, MOV10, FERMT1, MLLT10, AGO3, AGO2, SASS6, NMT2, PSMG1, FERMT2, PIGN, DPYS, KDM8, ARHGAP5, ECSIT, LRRC1, IKBKB, EEA1, NIPBL, DLGAP5, APPL1, APC2, UPF2, RIPK2, EMP2, SIRT1, RBSN, PSMA5, GPRC5B, BDH2, DPYD, PICK1, LYPLAL1, TRUB1, SYNPO2, SNX33, GGA1, RECQL5, PMS2, FLNB, STAT6, TRMT10A, RIC1, USP9X, MBD2, SAMD4A, YLPM1, VWA3B, TTLL5, CENPI, GID8, CCDC6, NCAPD2, PKN2, FRK, CCNT2, CCNT1, NCF2, CSE1L, GMNN, NCF4, GPR63, LTN1, TSEN2, EIF4ENIF1, STK11, HPGDS, TOM1, NEK1, MAP3K8, KMT5A, ZNF687, KPNA1, PSKH1, SH3GLB1, AGTPBP1, AREL1, IST1, STX8, FBXO11, MSH6, FRMD7, DMTF1, ARL8B, FTO, BLM, TUBGCP2, IREB2, STK39, GMPS, UAP1, FOXO3, SPATA7, HSP90B1, CTIF, ATXN1, ATXN7, PMVK, G3BP2, EVI5, BCL2L14, MDN1, BCL2L15, NEK4, NEK6, FBXO32, NFKB1, TFCP2, STK24, SP4, TUBGCP5, TUBGCP6, ACO1, TUBGCP4, OGT, LRCH3, NIT2, MYD88, MDM1, NFE2L2, POMT2, CIITA, SH2D4A, CDCA7L, LRRK1, ARRB1, BICD1, CDC73, BICD2, PPP6R3, ITGAV, UPP2, DNM1L, SPG11, UNKL, CTNNBL1, UNC45A, ACTR2, PPP1R12A, VPS13C, CEP295, ARFGAP3, RAD23B, ARFGAP2, MMS22L, PPM1A, PPM1B, NEMF, LCP2, LCP1, DTL, RNFT2, SEC23A, CARNMT1, SRC, ROCK2, ZBTB49, MLLT1, PPM1D, MLLT3, RCHY1, BARX2, PPM1F, EHBP1, MALT1, ZNRF1, PARD6B, TP53INP1, UBQLN1, CDC37L1, FEM1C, IGF2BP3, WHAMM, TRIM45, NTRK2, NOP58, SEC24A, MYO10, HIP1R, CABLES1, EFR3B, MYO19, TSPAN15, EIF3J, ATXN10, SEC24D, PIK3AP1, SEC24C, MYO1F, AHCYL2, AHCYL1, RTCB, THμmPD3, GCC2, IKZF3, EFR3A, CCDC91, BAIAP2L1, RASSF3, TOPAZ1, OGFOD1, FBXO3, KIF21A, CPNE3, LGALS8, SCAPER, FBXO7, SFMBT2, ALG2, MYO7A, BTK, ALPK1, KIF20B, EPHA3, CERKL, GRB7, DHX8, EPB41, PIK3R1, EVPL, PIK3R5, SNX3, DHX30, SNX1, SNX2, SEC14L1, PCBP4, DHX36, HIVEP1, STK38L, CEP192, SNX9, N4BP1, DECR1, SEC31A, TBC1D8B, WWTR1, YES1, XRCC4, SMARCA5, MICAL3, RASSF9, WAPL, POLA1, MSRA, WNK2, ECHDC1, FBXL3, RCOR3, INTS9, NRP1, PTPRR, HNF4G, ICA1, WASL, IDE, BRCA2, PPP3CA, PCM1, AKAP10, AKAP11, CNTRL, GIT1, BBS4, HTATIP2, ZHX2, SYK, NCOA6, IL15, NCOA3, IL16, ANO6, COMMD1, GAK, NR5A1, ENAH, TOX3, ARHGEF9, CTTN, VSNL1, G2E3, DEPDC5, STK40, CD44, ARHGEF5, YAP1, RABGAP1, NEDD9, TXLNG, MALSU1, GNAI2, EXOSC7, EXOSC9, PES1, EXOSC8, PCNT, EXOSC3, PAK2, EPS15, AASDHPPT, MAP3K2, GCH1, SORT1, PLEKHA5, KLHL3, UBE2G2, PLEKHA7, RGP1, ETAA1, ZDHHC9, KLHL7, SGSM3, KCNS3, TTC3, TACC2, SPC25, ICE2, MPG, WWC1, WWC2, UBE3A, NR3C1, TTF1, TMEM266, PPIP5K1, PPIP5K2, CAPN7, KIF5B, CAPN3, EP300, DENND5A, DIMT1, FBXW11, ACOT11, TTC7B, SSU72, LMTK2, CASK, TBC1D2B, DOK1, ADAM17, SHQ1, WDFY1, GEMIN5, WDFY3, TBC1D23, MASP1, RAF1, GEMIN8, DDX6, NDRG4, KLHL14, UBA5, DDX1, RASGRP1, ACACA, ADGRG2, GRK5, LPXN, CAMTA1, ECT2, MORC2, GCN1, CNTLN, VTA1, DENND4C, ECD, HOOK3, AP4B1, SYT7, KLHL20, AFDN, UBE2O, FGF13, LSM14A, CHD9, DDX42, CHD5, GHR, CALB2, CALB1, CA7, MAN1A1, LRRFIP1, SULT4A1, PLA2G4F, NCBP1, PDPK1, DDX55, PEX1, ETV6, PEX7, TBC1D2, ELF2, TBC1D4, ACOX2, IRF4, TBC1D5, PEX6, IRF2, CRY2, PLEC, PARG, DNAH7, NAA20, RAP1GDS1, DDX20, ASAP3, RNF8, CBL, ACVR1B, IL1RL1, PDLIM3, NWD1, TAF1D, CAND1, TGS1, MVB12B, MXI1, NUP43, ARFGEF1, ARFGEF2, EYA2, MAPK14, TTC17, GOLPH3, TNIP1, FAM131B, NAA16, NAA15, TAF4, CDA, IPO11, STMN3, GPATCH3, ABCA12, FNBP1L, SMC4, BACH2, RRP8, DCAF6, GLE1, ELK4, PCMT1, RNF114, TFG, ZMIZ1, MYB, WDR91, PLCE1, PIP4K2A, CEP97, RNF111, CDK5RAP2, PRKCI, ACOT7, PRKCH, MAP1S, PRKCE, DNAAF1, PRKCA, ARMC8, ATG14, HCFC2, ATG13, UNC13D, CLPX, NISCH, RNF123, DAAM1, WDR81, BIN1, PRKD3, NHLRC2, PRKD1, ZNF395, XPC, SLAIN2, VPS26A, BCL10, SKA3, SAMSN1, ABR, RIC8B, NUP85, PLCG2, PLCG1, PLK4, AFAP1L2, HOMER2, NFATC3, PRRC2C, UPF3A, INO80, GPN1, PPP5C, SNAI1, PLCH1, GTF2IRD1, CDK13, CPEB3, CDK14, ITPA, CEP89, SETD4, DENND1B, ITK, CDKN1B, PHF20, SETD6, ADK, CHM, SMG7, SMG6, TRIM8, AIFM2, SPIN1, LDLRAP1, CEP68, DENND2D, NUDC, MIOS, CIT, ITPKB, KAT2B, GCFC2, LSG1, AFTPH, NCOR1, RNF169, ZEB1, RARA, COL21A1, PLIN2, RCCD1, GAS8, PHLPP2, USO1, HOXD10, TANK, ARFRP1, ALOX5, SLC17A5, RPS2, BRD7, TIGAR, YTHDF2, CSNK1A1, GLYCTK, ELP2, ELP3, NMNAT2, SRP68, HIPK1, HIPK3, WDR11, RNF146, AGBL5, MKLN1, AGBL1, OSTM1, AGBL2, PLCD4, SLC28A1, DGKE, ANKRD37, DGKD, IRS1, FMR1, CTNND1, UBP1, LCLAT1, HID1, PPP1CB, GRAP2, PAPOLG, KCMF1, USP8, USP4, RALGAPA2, LARP4B, CHD1L, ZNF318, AAGAB, TNIK, PHLDB2, FAM114A1, TNKS, ANKRD11, ANKRD12, UBR4, RHOBTB3, RASAL1, AK5, AK6, GTF2E2, MTM1, ORC5, ORC4, BAG4, HMBOX1, BTBD3, DVL1, UBR5, ABL1, DVL3, RAB6A, SRGAP2, WDTC1, ANKRD27, OSBPL6, ATP8B1, OSBPL3, OSBPL2, ASNS, FANCC, FANCG, FAM217B, MOB1B, COPS3, CDK6, CNOT1, CNOT2, BCL2, TPP2, TADA1, SPIRE1, NF2, DRC1, RAB5A, MAP3K14, SERINC5, BCL2L1, PIK3C2A, PIK3C2B, PPP6C, OPA1, PSTPIP2, AP1G1, CEP170, HIF1AN, CCDC50, SH3D19, DTX3L, DIS3, ELAC1, KCNAB1, HAUS6, WIPI2, RANGAP1, PATL1, HAUS1, DNAJC3, DNAJC2, TRAF6, TRAF5, LANCL2, ASPA, GTF2A1, RNF32, NFAT5, PLEKHH2, NUFIP2, PLAG1, NLRC5, NOD1, NLRC3, AMBRA1, THSD1, HDAC7, DCAF17, VPS51, DNAJB6, VPS50, POLR2C, VPS53, VPS54, ASCC3, FNIP1, SLC13A5, CTNNAL1, DCAF13, JAZF1, SPAG9, TXNRD3, TXNRD2, DDHD2, PTPN12, PTPN13, PML, MTOR, KLF6, SNRNP40, RAB14, DNAJA4, SPAG1, SNRNP48, CCDC66, PPIL4, CUL4B, PANK2, MTRR, GORAB, DIXDC1, CBLB, GOLGA3, KAT7, TNS3, TBC1D10A, TANGO2, COG3, P3H2, AP1AR, DLEC1, TIAM1, CD109, ZW10, ALDH7A1, CFTR, TELO2, LLGL2, CASZ1, TEX264, DCUN1D1, RPAP2, FXR1, INPP5B, SPAST, PDCD10, INPP5D, TP53BP2, MAP7, STXBP5, ATP6V0A1, SRPK2, CARMIL1, ZRANB1, SS18L1, LIF, NMI, Pμm1, TRERF1, SRPK1, CCDC88A, PARP11, CDAN1, PDE3A, SPATS2L, TMCC1, CUTC, DCP1B, TOP2B, C2CD5, SLC44A1, FAF1, SRA1, SLC22A16, XPO4, XPO6, PARP4, PRMT7, PRMT3, PGGHG, PROX1, PPHLN1, PARP9, ITFG2, UCHL5, RAD51C, POC5, NFX1, NME8, MAPT, HAAO, TUBD1, LIMA1, NT5E, KIAA0753, CTNNA1, DCPS, APBB3, LCMT1, LMLN, SVIL, NQO1, STAC3, RANBP9, TJP1, DLC1, NBR1, RAB3GAP2, CTNNB1, MPHOSPH8, ARID4A, ARID4B, EFTUD2, CWC22, NARS2, KNTC1, NSF, ANGEL1, ANXA11, PPP4R3A, LZTFL1, PREPL, HGS, ELMO2, STAM2, STEAP2, PDE9A, RAI14, PXK, PXN, CLEC16A, ARHGAP18, ARHGAP17, CPLX3, CAMSAP2, HRH1, PDZD2, DPP8, DNAJC21, SUDS3, MYH10, RAP1GAP2, HSPA8, CBX7, SMAD4, SMAD3, PNPT1, EPHX2, IDH2, KLHDC1, SMAD9, AZIN2, DNAJC13, SMAD5, POU2F3, TDRD3, FER, CAPRIN1, CAPRIN2, CHMP2B, BRWD1, KDM5B, DOCK4, DOCK3, HSPB8, ELAVL1, CASP9, FLCN, HK3, SPRED1, CASP7, SENP5, NAPRT, KYNU, PDE4B, BLNK, POLH, RUBCN, USP48, KLF12, PDE4D, PPP2R5A, PPP2R5C, SENP2, ARID1B, CKAP4, IQCG, RBL2, ARHGAP10, PALLD, RBMS1, ITGB1BP1, MCTP2, DOCK1, FAN1, USP14, USP15, HPGD, USP10, UACA, USP19, GNA13, MAPK9, PPP2R3C, NRIP1, PDE6D, MAPK1, PDSS2, NCAM1, CLASP2, PTCD3, USP25, TCF7L1, NOS2, GDAP1, FMNL3, PIKFYVE, XPNPEP1, KANSL2, XPNPEP3, RGS10* |
| **brown** | **CC** | **GO:0005654~nucleoplasm** | **739** | 11.73 | 0.00 | *MAPKBP1, EHF, IPO11, HNRNPR, GPATCH3, SMC3, SLA2, GABPB1, RBPJ, PTPDC1, BACH2, RRP8, SMC2, ELK3, DCAF6, ALKBH3, ELK4, TBK1, PPP4R2, ZMIZ1, ALKBH1, CREB3L2, MYB, ZC3H12A, HOXA3, HOXA2, PIP4K2B, UBASH3A, NFRKB, STARD3, CXADR, ACOT7, RBFOX2, PRKCB, MAP1S, SOX13, DNTTIP2, ARMC8, SOX10, MED6, CLPX, NISCH, HOXB9, PRKD3, PSME4, AKIP1, HOXB3, LETMD1, SKAP2, URI1, ABCB6, PRKDC, MYNN, XPA, XPC, EPB41L5, RNF216, NUP85, MYO6, HOXC5, NUP88, LTA4H, PPARGC1B, CTC1, PHC2, PHC1, PRPF38A, PMM2, NFATC3, CDC7, UPF3A, NR1D2, SNF8, GPN1, PHC3, SNUPN, FOSL2, MLLT11, MLLT10, HNRNPK, AGO3, SNAI1, RAD17, PSMG1, GTF2IRD1, CPEB3, CDK14, FERMT2, ITPA, ATG2B, PHF2, SMG1, ZNF131, SETD6, KDM8, ADK, CTCF, OSBPL11, ECSIT, LRRC32, LITAF, CLINT1, NIPBL, CHAF1B, MED13, SPIN1, JAK2, CEP68, DUSP4, TEAD4, DENND2D, MYOG, DENND2C, UPF1, MIOS, WDR33, DDB2, PSMA6, GCFC2, FAM222B, GPRC5B, AFTPH, ZEB1, MTF1, RARA, RARB, PPARA, ERGIC1, MGLL, PHLPP2, SF3B3, PLPPR1, SYNPO2, HTT, HOXD10, GGA1, SCAF8, BTAF1, UBN2, ALOX5, USP1, PMS2, STAT6, RPS2, BRD7, ZNF346, MEF2D, BRD4, CENPU, TRMT10A, SLF1, MBD5, SLF2, TIMMDC1, ATAD2, SAAL1, ELP4, RRP1B, VWA3B, SCAF4, LYRM1, MKLN1, NFIA, CENPH, NFIB, CENPJ, GID8, NCAPD2, NCAPD3, LGR5, CENPQ, FRK, DGKE, ANKRD37, ZBTB25, IRS1, FMR1, CSE1L, GMNN, GPR63, UBP1, RORB, TSEN2, PPP1CB, HPGDS, STK11, GRAP2, SESN1, ZNF207, NEK1, KMT5B, KPNA4, ZNF326, KMT5A, PITX2, ZNF687, KPNA1, TLE4, MAGI1, PIAS4, TLE3, CDKL5, USP3, CHD1L, VRK3, THOC5, FBXO11, EMSY, MSH6, JAML, FRMD7, MAPKAPK3, DMTF1, MSH2, ZNF318, PKP2, TNIK, UTRN, CDKL1, ANKRD17, NOTCH2, PFKFB2, CD274, CSF1R, PFKFB3, FAM114A1, TUBGCP2, ANKRD11, CUL2, BOD1L1, ANKRD12, UBR4, STK39, ITPR2, UAP1, SPATA7, ORC5, ORC4, ATXN1, ORC1, ATXN7, UBR5, ORC2, HACL1, ZNF423, MDN1, IP6K2, AKIRIN2, FANCM, NEK6, NEK7, STYX, ZBTB10, ZBTB11, FOXN2, FBXO32, PALB2, NFKB1, FAM217B, ZC3HC1, MOB1B, COPS3, IBTK, TFCP2, CDK6, UBLCP1, CNOT2, SP4, BCL2, SPIRE1, GPR107, CSPG4, HPSE, XAF1, RAB5A, MAP3K14, NFE2L2, ZNF512B, POMT2, CIITA, BMPR2, BNC2, CELF1, CELF2, CDCA7L, WDR3, SNAP23, CEP164, PIK3C2A, DUSP16, SYNE1, PIK3C2B, DUSP12, METTL14, OPA1, PPP6R3, PHACTR3, HIF1AN, ITGB6, NEO1, CTNNBL1, SH3D19, PLEKHG1, RFC1, DTX3L, DIS3, COMMD10, ELAC1, ACTR8, WIPI2, DUSP26, RAD23B, METTL22, SMDT1, TFDP2, CTDSP1, ESRP2, ZNF638, C1D, HAND1, LANCL2, POLR1E, NAF1, DAGLB, BDP1, DTL, GTF3C1, NFAT5, SMARCD1, SMARCD2, GTF3C3, SMARCD3, GTF3C4, UHRF2, SRC, ZBTB49, RNF38, ATP10D, PPM1H, TULP3, PPM1D, RCHY1, PPM1E, BARX2, TMA16, EHBP1, DCAF17, DNAJB6, UBQLN1, BEND3, VPS54, FEM1C, E2F5, SLC13A5, PCDH1, UBQLN4, DCAF13, TRIM45, JAZF1, RNF20, CIZ1, TXNRD3, KRR1, CEP152, FOXJ2, PTPN13, PTPN14, KLF6, NIN, KLF5, POLR3A, APC, PRC1, ASXL2, APLF, POLR3E, SNRNP48, PPIL4, TNRC6A, CCDC62, CUL4B, SMARCAL1, CCNK, MTRR, GORAB, RTCA, RTCB, CBLB, GCC2, ZDHHC7, IKZF3, GOLGA3, ZNF609, GOLGA4, CCDC91, RPS6KA5, BAIAP2L1, OGFOD1, CHEK1, CDC27, TRIM24, DPF3, FBXO3, CPNE3, EPHB2, SOX6, FBXO7, TP63, SMARCC1, AQR, COG5, SFMBT1, RECQL, P3H2, ACSL5, UHMK1, INPP4A, NEIL3, SUB1, XRN2, SIK3, UPRT, DNA2, FAM76A, KIF20B, BCORL1, ZFPM2, EPHA3, CERKL, CASZ1, L3MBTL3, FUT10, MYCBP2, DCUN1D1, NMD3, NUAK1, BRIP1, SEC14L1, SPAST, PGBD5, MIER3, MIER1, CSTF1, DPH6, HIVEP2, RAB11FIP2, RAB11FIP3, LYAR, SCAI, DECR1, ZRANB3, TRAP1, ZRANB1, XRCC4, SETDB1, XRCC5, BCL11A, SS18L1, XRCC2, SMARCA5, MICAL3, NMI, SMARCA1, Pμm1, TRERF1, SMARCA2, WAPL, Pμm3, SRPK1, POLA1, ANLN, MSRA, ZBTB6, SNW1, FUBP1, SPDYA, SPATS2L, GRB2, ATM, INTS6, INTS9, RCOR3, CUTC, RCOR1, TECPR1, SUGP1, TOP2B, SLC44A1, FAF1, MCUB, HEPACAM2, HNF4G, SRA1, RSF1, BRCA2, AATF, PPP3CA, ZMYM3, XPO4, PCF11, SUFU, XPO6, ARIH2, GABPA, PBRM1, ZHX2, NCOA5, NCOA3, COMMD1, PROX1, PPHLN1, IL17RD, PARP9, ITFG2, SREBF2, UCHL5, NAALADL2, NR5A1, RPUSD4, ANO1, TOX3, KIN, TOX2, RAD51C, POC5, NFX1, TFEC, WAC, STK40, DCP2, ARHGEF5, STX12, TUBD1, NEDD9, GNAI2, MNT, NT5E, EXOSC9, PES1, HSF2, EXOSC8, PAK6, RAD54L, EXOSC3, DCPS, LNPK, PAK5, MAP3K2, TSNAX, ARNT2, RANBP3, GCH1, PLEKHA5, STAC3, MLH1, GRHL1, GRHL2, PLEKHA7, PLEKHA8, BATF, NEDD1, SH3RF2, ETAA1, GFI1B, ITCH, KLHL7, TTC3, KLHL8, TRIP12, TACC2, MPHOSPH8, NEK11, DCLRE1A, NARF, KDM1A, KDM1B, MPG, DSCC1, ARID4A, ARID4B, NUCKS1, JADE2, TTF1, CWC27, ETS1, TATDN1, CCND3, NARS2, TRAPPC12, ARID2, CMTR1, PDGFRA, MORC4, DIMT1, SLC30A5, ANXA13, SSU72, ANXA11, LIG3, LMO7, ACYP1, CAMKMT, SCNN1G, SHQ1, ADAM12, UBE2V2, MASP1, LRIF1, STAM2, PDE9A, RAI14, YTHDC1, DDX1, ZCCHC9, DTX2, ARHGAP17, PDS5B, PDS5A, RAI1, STAP1, MORC2, PDZD7, KLHDC10, BCL9L, MFAP3L, BPTF, CNTLN, TFAP2A, CBX7, SMAD4, POU2F1, CPSF6, SMAD3, RHBDD2, VTA1, PCIF1, ECD, SMAD9, SMAD5, TDRD3, CLK3, NMRAL1, ARHGAP32, APPBP2, UBE2T, CAPRIN2, UBA2, TCF3, KDM5B, GSK3B, NVL, CHD9, USP33, CHD6, ADAR, LDB2, ELAVL1, SPRED1, SENP5, SNAPC1, TRPS1, KYNU, POLL, NOS1, MYBL1, RBM5, POLH, RUBCN, USP48, KLF12, TGIF2, NCBP1, DDX55, RBM19, DARS2, PPP2R5C, ARID1A, SPIDR, UTP18, ARID1B, DCK, DNM1, SENP1, RBL2, TBC1D2, RBL1, PEX3, IRF4, IRF2, MCTP2, KDM7A, FAN1, KDM3A, PARG, HLF, RBM48, KDM3B, HPGD, USP10, ASAP3, RNF8, LIN9, UACA, IWS1, ATAD2B, STRN3, TAF1D, RMND5A, CAND1, TAF1B, PPP2R3C, TDG, BMS1, MKNK1, TAF1A, MXI1, ARFGEF1, PTCD3, SPEN, TCF7L2, TCF7L1, EYA3, KDF1, ARID3B, LRWD1, ATP2B1, TBX18, WARS2, ADGRD1, RPS6KB1, TNIP1, FAM131B, NUP35, FAM98B, QRICH1, RBM45, CCDC174, CLUAP1, NUP37* |
| **brown** | **CC** | **GO:0005813~centrosome** | **164** | 2.60 | 0.00 | *DCLRE1B, ITSN2, TRAF3IP1, CEP120, CCP110, TSEN2, TPGS1, GLE1, TBC1D31, CAPN7, CSPP1, CHEK1, CDC27, CEP250, CEP95, RTTN, KIFAP3, PRKACB, CDKL5, CEP135, IST1, FBXW11, IFT80, PPP4R3B, NUP93, PROCR, DYNC1LI2, CLIP1, IFT88, KATNAL1, FAM161A, IFT81, AKAP9, KIF20B, ALPK1, TUBGCP2, CUL3, FBF1, ZBTB1, UBR4, SLAIN2, AK6, SKA3, CAMSAP2, PKHD1, HMBOX1, SPAST, KIF3B, CEP70, ORC2, CCDC141, CNTLN, PLK4, UVRAG, SMAD4, CEP112, STIL, ODF2, CEP350, XRCC2, TRIP4, WAPL, AHI1, CDK6, CDC16, CAPRIN2, TUBGCP5, MKS1, TUBGCP6, ATM, TUBGCP4, NIT2, FBXL7, RAD18, SERINC5, CEP89, NFE2L2, MDM1, CEP57, GSK3B, USP33, CEP164, HEPACAM2, BRCA2, BICD1, BICD2, FLCN, IFT52, ACTR1A, SENP5, CNTRL, PDE4B, CEP170, ITGB6, IFT57, RAB8A, SLC18A2, CTNNBL1, TAPT1, GIT1, NDEL1, LRRCC1, TGIF2, PPP1R12A, IFT140, CEP295, PDE4D, PPP2R5A, KIF24, AXIN2, ACTR8, CKAP5, WDR35, HAUS2, HAUS1, WRAP73, KAT2B, RAD51D, KIF2A, POC5, NME7, TRAF5, CEP63, DTL, KIZ, DYNC1I2, RGS14, ROCK2, PLAG1, DCTN4, NLRC5, GNAI2, IFT74, WRN, HAUS8, PPP2R3C, KATNA1, TMEM67, POC1A, LEO1, SNCG, POC1B, RBBP6, DISC1, DCAF13, DYNC1H1, SLF1, EYA3, SCLT1, HIPK1, TTC39A, MZT1, MPHOSPH9, NIN, TTLL5, APC, KIAA0586, CENPJ, ALMS1, CTNNB1, CEP162, PKN2, CCDC66, CEP44* |
| **brown** | **CC** | **GO:0005794~Golgi apparatus** | **221** | 3.51 | 0.00 | *EHF, SLC35B4, GORAB, STMN3, ZDHHC7, AQP2, SLA2, ZFYVE1, ZDHHC3, GOLGA4, KDR, UBASH3A, KIFAP3, CDK5RAP2, DYNC2H1, CUBN, PRKCE, TANGO2, GLCE, SLC35C1, COG2, P3H2, AP1AR, ACSL3, FNDC3A, UHMK1, SPATA16, RNF128, AKAP9, UNC50, ATF6, PDXDC1, PKHD1, SNX1, PRRC1, SEC14L1, ST3GAL4, VTI1A, ST8SIA4, ST3GAL5, ST8SIA6, KIAA0319L, VTI1B, ATP6V0A1, ST3GAL3, ABCA1, LYN, RABGAP1L, ST8SIA1, SLC35E1, RAB27A, ABCA7, WAPL, EXT1, EXT2, CCDC88A, GKAP1, NAPEPLD, CALU, NMT2, CEP83, GRB2, PSMG1, CDK13, ITM2B, ATR, CEP57, HEPACAM2, AP4E1, OSBPL11, CLINT1, LITAF, SH3RF1, WDR44, CTLA4, APC2, MYOC, PPHLN1, IL17RD, ZDHHC15, GAK, RNF144A, BACE2, CTTN, CSDE1, G2E3, FAM20B, VAMP4, ERGIC2, CD44, SLC29A3, TLR2, RAB7A, STX12, HTT, TM9SF4, STX11, LMAN1, GGA1, ZDHHC18, APBB2, EXTL3, TSNAX, GOLM1, SORT1, GLYCTK, YIPF4, PLEKHA3, PLEKHA8, TM9SF2, MPHOSPH9, KCNS3, NEDD4, APBA1, RAB3GAP1, ATP8A1, FAIM2, CXCL14, TRAPPC12, TRAPPC11, LARGE1, RAB2A, PDGFRB, IGFBP1, SLC30A7, NSF, SLC30A6, PDGFRA, FBXW7, SEMA6D, SLC30A5, TSC2, LMTK2, MSH6, SMO, TBC1D23, RAF1, TRAPPC3L, PKDCC, FAM114A1, TNKS, CUL3, UBA5, CLEC16A, SLC38A10, PLD1, CAMSAP2, ARHGAP21, ASIC1, ATP8B2, SYT1, ATP8B1, PTCH1, DENND4C, TRAPPC9, ESR1, COQ6, TDRD3, ARHGAP32, BCL6, GPR107, ACO1, FGFR4, SERINC3, SERINC5, NFE2L2, DOCK4, ST6GALNAC2, CPQ, USP33, DYSF, PKD2, BICD2, SYNE1, SGCE, FYCO1, IFT57, DNM1L, NEO1, PRKG1, UNC45A, VPS13A, PPP2R5C, ARFGAP3, DUSP26, ARFGAP2, GORASP2, TBC1D5, TRAPPC6B, RP2, GORASP1, DNAJC5, CDH15, RNFT2, PRKAA2, GALT, LRP2, CBL, CLCN3, BARX2, LRP6, STRN3, CAND1, RAB20, RAB40C, PPP2R3C, MAPK1, CLASP1, TMEM30A, RNF24, DDX31, HS3ST5, LYSMD3, HS3ST2, RAB10, FMNL3, KLF5, APC, VAPB, VPS41, TNRC6A* |
| **brown** | **CC** | **GO:0005737~cytoplasm** | **648** | 10.29 | 0.00 | *MAPKBP1, PID1, FNBP1L, RBPJ, SMC2, RNF114, TBK1, TFG, DPYSL5, DIP2B, ANKS1A, PKNOX1, ACOT8, MEF2C, PRKAB2, STARD7, DAPK1, FNBP1, PRKCB, SOX13, ARMC9, ARMC8, DYNC1LI1, ACE2, DYNC1LI2, BIN2, RBP2, BIN1, RUFY2, TBPL1, PRKCQ, TARS2, PRKD1, IDO2, CLOCK, GPLD1, URI1, MTMR3, PDGFB, NCAPG, MTMR9, COPS7B, IQGAP2, SKA3, PRKCZ, SKA2, RIC8B, CEP70, CFAP69, LTA4H, CEP76, PLK4, FZD3, STARD13, AFAP1L1, DNASE2B, PMM2, TRIP4, CDC7, NR1D2, BTBD10, GPN1, SNUPN, CPEB1, HNRNPK, REL, MKS1, ITPA, CEP57, PYGB, SMG1, BTG1, SETD3, MTMR11, FMN1, SMG5, SMG6, IFIH1, IKBKB, DSTYK, IMPA2, TRIM2, HEY2, JAK2, ENOPH1, JAK1, NDC1, UPF1, NUDC, CNOT6L, RIPK4, TCF12, NSUN6, PTGR2, LRRC41, CIT, PSMA5, PSMA6, COL2A1, MTF1, MTF2, RGN, PLEKHO1, CEP63, PLCB1, PLCB2, PAFAH1B1, KANK1, CDC123, PRUNE2, HTT, ADCY2, PSMA7, FGD2, FGD3, FGD4, FGD6, UNK, ALOX5, ANKRD54, MEF2D, DTD1, RPRM, FARP2, EVC2, HIPK4, CENPU, SLF1, YTHDF3, STAT1, AGL, STAT3, BBOX1, WWP2, ELP4, STXBP5L, HIPK2, FABP2, SNX19, ALMS1, HYLS1, PKN2, CEP44, PUS10, UBE2D1, AHR, BZW2, ALDH1L2, MYLK3, IPO8, MAEA, IPO9, GRB14, SESN1, TUBB1, MAEL, KPNA4, KPNA3, MAP3K4, RARS2, MAGI1, PIAS4, RALGAPA1, SWAP70, SSUH2, THOC1, VASH2, SPICE1, DYRK1A, FRMD4A, MITF, FRMD4B, CLIP1, TXLNB, UTRN, ANKRD17, FTO, DRC7, RASAL3, NEUROD1, HNRNPDL, BTBD1, TPR, SMYD4, SLC38A2, ODF2, TNK2, NEK7, STYX, NEB, COPS3, PTPRE, MFHAS1, IRAK1BP1, CNOT6, IBTK, SLC7A6OS, COPS2, RNF182, NF2, MAP3K13, GDPGP1, MYD88, TUBA8, MDM1, NFE2L2, OSCP1, TRIM71, CELF1, CELF2, CDCA7L, AKAP8L, PTEN, PTPN22, DUSP16, DUSP12, SIN3B, HYDIN, RNF17, ERC1, SPDL1, TNPO1, CEP290, TRIM67, ACTR3, EIF5B, CDADC1, PPP1R12A, TRPC6, ACTR5, HAUS3, ARNT, TRAF1, KCNAB2, PATL1, NT5C3B, IMPACT, EVC, TRAF3, TRAF6, C1D, TRAF5, HAND1, SOS1, BIRC2, ITIH4, PRKAA2, ROCK1, GTPBP10, TULP4, PPM1H, TTK, CXXC4, ABLIM1, PARD6B, ABLIM2, ABLIM3, SH3BP1, KATNA1, KRIT1, CDC37L1, LRRC8D, LRRC8B, E2F5, WASF2, WASF3, CTHRC1, SPAG9, KCNIP1, KCNIP2, IRX3, KCNIP4, CDC5L, CFLAR, CDC42BPB, MERTK, CDC42BPA, PTPN14, FBXL12, GSTZ1, DBNDD1, PFKL, PTPN9, TRIM37, PDCD2, CD247, PTPN4, PPP1R14D, MMACHC, SMAP1, PTPN3, PANK1, SLC23A1, RTCB, AQP4, NADSYN1, PDCD2L, CCAR1, RPS6KA5, HERC1, CHEK1, CDC27, DHX58, TRIM24, FBXO4, EIF2D, CPNE2, BTRC, DYNC2H1, JMJD4, SMARCC1, TIPIN, IL1R2, YOD1, HNF1A, ANK1, MTURN, INPP4A, SHTN1, NIF3L1, KATNAL1, ULK3, TLN2, ZFPM2, CBLL1, PTGFR, GGNBP2, INTU, NMD3, PPL, FXR1, NUAK1, BRIP1, MAP2, XRRA1, MIER2, STXBP5, MAP4, KCNN3, TP53BP1, RIMKLB, SCAI, BBS2, GTF2A1L, CARMIL1, DTNB, SETDB1, XRCC5, SIAH2, INTS2, RANBP17, SIAH1, PARVA, NTAN1, DERA, LACC1, GDF9, HAL, PEX5L, FUBP3, WNK1, ID2, CDC16, CCDC88C, SPATS2L, INTS7, DHX29, FBXL5, TECPR2, FBXL4, FBXL7, DCP1B, AHCTF1, TENM4, CCDC125, OTUD7A, OLA1, OTUD7B, BRCA1, AATF, SH3RF1, ACTR1A, XPO1, SH3PXD2A, SUFU, XPO6, SLC22A18, SH3PXD2B, ARIH2, KIF13B, KIF1B, ARIH1, BBS7, BBS5, SPTAN1, LMCD1, NDEL1, ANKRD6, VAV3, NCOA2, CCDC113, RHOH, GAB2, MRPS5, PDRG1, MRPS9, NR5A2, KIF2A, MDFIC, GAN, CNKSR3, ARHGEF3, FAM193B, μmPS, DEPDC5, MAPRE2, PRMT9, KIZ, TLR3, SDCCAG8, IGHMBP2, NEDD9, VWCE, PURA, PAK1, RELN, HSF2, PAK6, CTNNA3, METTL8, PAK3, MAP4K5, DCPS, DYNC1H1, GABBR2, ARNT2, MON2, RYK, ERBIN, KLHL1, KLHL2, XPO7, TUBE1, MYO9A, BATF, NEDD1, KITLG, PER3, XPOT, TTC4, OTULIN, RAB3GAP2, TACC2, ACTR10, RAB3GAP1, PRDM4, JADE1, NUCKS1, DEUP1, NR3C1, ARHGAP35, KLHL31, NR3C2, CCND3, HELB, RGS3, CCND2, KIF5C, MEIOC, PPFIA1, BAALC, CAPN3, AZI2, PPFIA4, SH3GL2, CMTR2, NSF, GUCY1A2, CD96, PCID2, TBCCD1, ANGEL2, ANXA13, THEMIS, ANXA11, LMO7, UBE4B, CDYL, PPP4R3B, LZTFL1, MID2, CAMKMT, PSPC1, SHPK, SCNN1A, SPECC1L, ELMO1, ELMO2, PLAA, FBN2, DDX4, UBA6, ARHGEF28, KLHL10, DTX1, DTX2, ARHGAP17, DTX4, PLD1, CAMSAP1, ZFP36L1, STK3, VCPIP1, ODF2L, GRK2, SNTG1, CASKIN2, LONRF3, CCDC141, LONRF1, WNT2, TBC1D15, ANKZF1, FAM83B, MFAP3L, SMAD2, SMAD4, ZFHX3, CPSF6, SMAD3, ARHGEF37, UBE2B, HSPA4, DENND4A, ARHGEF38, ARHGAP29, IFT122, SMAD9, TDRD7, CNOT10, TBCA, HOOK3, TDRD5, ARHGAP26, SMAD7, QDPR, TBCK, SEPSECS, TSGA10, KIF18A, DIAPH3, CNOT11, CAPRIN1, TBCE, BHLHE40, PI4KA, MAP6D1, DYNLRB2, ZCCHC4, FGF13, GALK2, GSK3B, DOCK8, NGLY1, PLEK, SLC2A2, ADAR, MTR, ELAVL1, CHD1, PITPNC1, SENP7, CASP8, CASP3, KYNU, USP47, SUN1, CAMK1D, ARHGEF12, DESI2, USP45, RBM19, ARAP2, AXIN2, SHROOM1, IQCD, IRAK4, GPCPD1, CKAP5, PJA2, IRF4, CDH11, PFDN1, CDH13, IRF5, NCBP3, IRF6, DOCK2, DOCK1, RBKS, BLVRA, SASH3, KDM3A, DDC, COLEC10, USP15, RGS14, DNAH8, ANKS3, LEF1, RNF8, PARN, ASAP1, ASAP2, CBL, RNF4, RNF2, ADD1, MAPK8, RMND5A, CLMN, PPP2R3C, POC1A, BNIP2, SNCG, EPS8L3, SASH1, TRDMT1, MAPK4, ATE1, EYA4, DTNBP1, ATP2B2, ACVR2B, ACVR2A, NAP1L4, RSPH3, GNPDA2, KATNBL1, CFAP36, LNX1, CLNK, FAM98B, NAA16, NAA15, RBM45, CCDC172, MBTPS2* |
| **brown** | **CC** | **GO:0005769~early endosome** | **70** | 1.11 | 0.00 | *NRP1, DYSF, SNX13, RAB22A, EEA1, TOM1, TMEM108, ANKFY1, LDLRAP1, CCDC93, WLS, EPHA4, RUBCN, USP8, DTX3L, STX8, STX7, LMTK2, COMMD1, ZFYVE16, RABEP1, SLC9A6, RAPGEF1, NμmB, WDFY1, RAB38, RIN3, WDFY2, ATP6V0D1, STEAP2, EPHA3, CFTR, LRP1, USP10, TGFBRAP1, CXCR4, HTT, VPS26A, TRAK1, TM9SF4, LRP6, TRAK2, FGD2, SNX3, INPP5F, RPS6KC1, MVB12B, VPS11, PACSIN2, MAPK1, APBB2, WASF2, KIAA0319, PTPN1, UVRAG, NTRK2, RABGAP1L, ANKRD27, SORT1, PPP1R21, MIB2, IGF2R, ITCH, MYO1B, VPS41, VIPAS39, GPR107, NF2, NOX1, PICALM* |
| **brown** | **CC** | GO:0005814~centriole | 57 | 0.90 | 0.00 | *C2CD3, CEP164, CEP120, CCP110, CEP128, GLE1, IFT52, PCM1, DZIP1L, CEP250, CEP170, CEP97, BBS4, CEP135, IFT140, CEP295, ARMC9, SPICE1, KIF24, IFT88, POC5, KIF2A, RP2, SDCCAG8, CCDC68, FBF1, CCDC146, TUBD1, HTT, POC1A, BNIP2, POC1B, CEP192, PCNT, CEP76, CNTLN, PLK4, STIL, ODF2, CEP350, CEP152, PLK1, SCLT1, CCDC88A, NEDD1, MPHOSPH9, NIN, CENPJ, ALMS1, SASS6, MKS1, CEP83, CEP162, HYLS1, CEP44, CEP89, MDM1* |
| **brown** | **CC** | **GO:0016604~nuclear body** | **103** | 1.63 | 0.00 | *DCLRE1B, CSF1, RIF1, PRDM5, TESK2, IKZF4, ALKBH8, PAPOLG, EIF2D, SCAF11, KNL1, HCFC2, SGO2, MAU2, YME1L1, GEMIN5, NUP98, TELO2, ZCCHC8, KMT2E, DHX8, TNKS, EPB41, CSTF2, ZBTB1, GATA4, MTDH, HMBOX1, ORC3, XRRA1, ABL1, HIVEP1, SUDS3, TP53BP1, ECT2, WWTR1, ZFHX3, HACE1, ATP8B1, FANCL, TRIP4, INO80, POU2F3, FLI1, POU6F1, PARP11, STAG1, UBOX5, BHLHE40, TPP2, UBE2O, FBXL3, INTS7, RAD18, THRB, HSPB8, PPWD1, TCF20, ARRB1, BRCA1, SμmO1, EXO1, UBXN7, POLK, ARIH1, RAB8B, NCOA2, PARP1, ARNT, BAZ1B, TERF2, NCOR2, ELF2, DBF4, RNF169, KIF2A, SLTM, RP2, ESRP1, ITGB1BP1, TKT, AMER1, RNF32, PLEKHH2, RGS14, NUFIP2, IGHMBP2, CCNDBP1, RNF2, ADD1, UBN1, NCAM2, CORIN, SUZ12, PCGF2, RANBP9, NUDT9, TSPAN15, CENPI, POLE2, NBR1, PKN2, NAA15* |
| **brown** | **CC** | **GO:0005925~focal adhesion** | **66** | 1.05 | 0.00 | *BCAR3, NRP1, USP33, ITGA2B, DCAF6, SLC6A4, ITGAV, PGM5, CPNE3, ITGB6, TNS4, JAK2, TNS3, GIT1, ACTN1, ITGA2, ITGA1, IL16, SENP1, ENAH, ADAM17, PEAK1, PALLD, IRF2, MTF2, EVL, LCP1, TLN2, MAPRE2, PHLDB2, VCL, PLEC, GRB7, FOCAD, SRC, SYNPO2, PXN, ASAP3, IQGAP1, THSD1, LIMA1, IL1RL1, EPB41L5, FLRT2, LPXN, MAPK1, PTK2B, RSU1, CLASP1, CLASP2, LMLN, CAV1, PARVA, SRP68, ARHGAP26, CORO2B, FERMT1, UBOX5, PTPRC, PTPRA, FES, DLC1, ITGA11, TADA1, FERMT2, LIMS1* |
| **brown** | **CC** | GO:0000151~ubiquitin ligase complex | 26 | 0.41 | 0.00 | *RNF11, UBE3D, RNF8, DCUN1D1, UBE2D1, UBR2, UBR1, UBE2J2, RCHY1, RMND5A, FBXO8, RNF20, SMURF2, MIB2, RANBP9, UBE4A, UBE4B, ARMC8, FBXO11, FBXL12, RNF168, MKLN1, TRAF7, NEDD4, GID8, ASB2* |
| **brown** | **CC** | **GO:0098978~glutamatergic synapse** | **90** | 1.43 | 0.00 | *NRP1, GSK3B, DGKE, DGKB, LRRK2, CLSTN1, GRIK3, ARHGAP39, PLAT, ELAVL1, CDH8, EPS8, GRM3, GRIP1, PPP3CA, PPP3CB, PPP3CC, CALB1, NETO1, PSD2, CAMKV, RNF19A, FLOT2, TNR, BSN, ERC2, EPHB2, KPNA1, UNC13B, EPHA4, GRID2, EPHA7, CDKL5, ACTN1, WNT5A, ADAM10, SORCS3, DNM1, GRIP2, PLCB4, CACNB4, CDH11, NμmB, VAMP1, SPARCL1, DGKI, GRIA1, NAPB, RGS14, PLPPR4, PPM1H, PLG, ADCY8, FBXL20, FXR1, ARHGAP22, ABR, PURA, ACAN, GRIN2A, ABLIM3, SV2A, DVL1, PLCG1, LRRC4C, PAK3, PAK2, MYH10, WASF3, LGI1, EGLN1, ATAD1, NTNG1, HSPA8, HOMER1, SYT1, HOMER2, CADPS, PLEKHA5, DTNBP1, ERBIN, ATP2B2, ATP2B1, MAPK14, SYT7, PTPRD, SLC4A8, DLG2, NEDD4, APBA1* |
| **brown** | **CC** | **GO:0000139~Golgi membrane** | **95** | 1.51 | 0.00 | *GALNT12, GALNT11, SCYL3, GALNT14, SFT2D3, TMEM167A, PGAP3, GALNT18, CLSTN1, GOLIM4, XYLT2, ICA1, ABCA12, MANEA, GALNT10, HS2ST1, NDST2, NDST1, GOLGA5, GNPTAB, MAN1A2, MYO18A, MAN1A1, ARFIP1, A4GALT, SLC35A5, GALNT8, WLS, RHBDF1, SH3GLB1, ST6GAL1, COG7, COG6, COG4, ADAM10, ZDHHC13, EMP2, MANEAL, MAPK8IP3, ZDHHC17, TPST1, RNF125, GORASP2, B3GNT4, B3GNT2, KDELR2, CHST4, SFT2D2, SFT2D1, B4GALT4, CHST3, COPB2, SEC23A, RTN1, ABCB6, FUT10, GOSR2, GOSR1, GCNT1, USO1, FURIN, PSEN1, GLG1, UGCG, PDCD10, MAN2A1, MGAT5, GCNT3, GCNT4, WHAMM, RAB6A, ST3GAL2, B3GALNT2, ARFGEF1, ARFGEF2, SLC35A1, GALNT5, NDFIP2, SLC10A7, NDFIP1, HACE1, GALNT2, RHBDD2, CAV1, B3GAT2, GALNT1, B3GALT5, SORL1, ARCN1, GOLPH3, RAB14, VAPA, UST, VPS45, ACBD3* |
| **brown** | **CC** | **GO:0005730~nucleolus** | **197** | 3.13 | 0.00 | *MAPKBP1, GORAB, THμmPD3, RBPJ, RRP8, SMC2, GLE1, GOLGA3, CPNE3, DDX18, COG7, SCAF11, MAP1S, MTUS1, DNTTIP2, HUS1, WDR75, ACSL5, SUB1, XRN2, KIF20B, LETMD1, L3MBTL3, MEAF6, PRKDC, XPC, NMD3, RTF1, RPAP2, DPH6, KIAA0319L, LYAR, N4BP1, SRPK2, PLK4, XRCC5, Pμm3, ZZZ3, RAD17, SPATS2L, INTS4, NOL10, GRB2, ATM, NOL11, CUTC, PHF2, TOP2B, OLA1, NAT10, CTCF, AATF, GLI3, GLI2, SMG6, AKAP11, XPO6, SPIN1, REV3L, WDR36, MRPS27, PARP1, GNL2, UCHL5, GCFC2, GPRC5B, MRPS9, RNF169, KIF2A, MDFIC, NFX1, AGPS, FAM193B, ERGIC2, SF3B3, HEATR1, MRPS31, NOL8, NOL4, EXOSC7, WRN, EXOSC9, EXOSC3, ZNF346, SUZ12, TRMT10A, UTP6, MTX2, STAT1, ELP3, CENPH, KLHL7, TTC3, TSR1, MPHOSPH8, NEK11, ZNF330, NARF, FMR1, PRDM5, NUCKS1, TTF1, ZNF207, CAPN3, NBN, NEPRO, MAGI1, AGTPBP1, FBXW7, DIMT1, SLC30A5, LARP4B, VRK3, FBXO11, PSPC1, WDFY3, IPPK, BLM, ZCCHC9, ITPR1, RPF2, ORC4, ATXN1, DNAJC21, ABL1, CAMTA1, UTP20, MDN1, BCL9L, POU2F3, FANCG, MOB1B, RCL1, LIN28B, DIAPH2, STK24, BCL6, UBLCP1, UBE2T, PLEKHM1, NF2, BRWD1, ZCCHC4, POMT2, DOCK4, NVL, ITGB4, WDR3, ADAR, LDB2, SYNE1, SENP5, SNAPC1, SμmO1, SPG11, UTP15, PPP1R12A, DDX55, BCKDHB, RBM19, ETV4, UTP18, CKAP5, ETV6, RBL2, METTL22, C1D, HAND1, DTL, KDM7A, GTF3C1, GTF3C3, TULP3, PPM1D, PPM1E, TMA16, NWD1, VPS51, RBBP5, BMS1, MXI1, BEND3, RPP14, PCDH1, DCAF13, RNF20, ARFGEF1, NOP58, MYO10, KRR1, DDX31, LRWD1, OXR1, PML, NIN, ESF1, NRDE2* |
| **brown** | **CC** | **GO:0016607~nuclear speck** | **117** | 1.86 | 0.00 | *JADE1, GPATCH2, NR3C1, SMC4, EFTUD2, PNN, CWC22, SAP130, CCNL1, SCAPER, PSKH1, FNBP4, MEF2C, DNAAF1, SFMBT2, THOC1, PRPF4B, PPP4R3A, PPP4R3B, CDYL, CDC40, PIAS1, PRPF4, PRPF6, PSPC1, SRSF4, FAM76B, AAGAB, SREK1, RAF1, CBLL1, FTO, ZNF395, ARHGAP18, AK6, GTF2E2, GRK5, DHX36, ZC3H18, LPXN, PIP5K1A, ZC3H13, ATP6V0A1, SRPK2, BARD1, CARMIL1, CPSF6, SMURF2, API5, BDNF, DNAJC11, COPS4, AFDN, SON, SPRTN, HBP1, CDK12, CDK13, DENND1B, RBM25, DDX46, AKAP8L, DDX42, CHD5, GLI3, GLI2, AFF2, VPS72, NFKBIZ, DHX15, POLI, UNC45A, BRD1, IL15, TCF12, IL16, HAUS6, PLRG1, CKAP4, PRPF18, THRAP3, NME8, CRY2, LUC7L3, NCBP3, FAM193B, MAPT, NSRP1, PLCB1, DOCK1, FYTTD1, PRKAA2, PLAG1, WBP4, SRSF1, NSL1, WRN, BCLAF1, NRIP1, HSF4, RBBP6, SNRPB2, NUP43, SF3B1, SRSF11, RBM39, SF3A1, CSNK1A1, YLPM1, ERBIN, CDC5L, HIPK1, MAPK14, SNRNP40, TRIP11, NRDE2, SLC28A1* |
| **brown** | **CC** | **GO:0030425~dendrite** | **66** | 1.05 | 0.00 | *ZDHHC5, HTR2A, PTPRK, SGCE, TMEM266, ALCAM, CALB1, PSD2, DPYSL5, CHL1, KIF21A, DIP2B, BSN, EPHB2, VSTM5, SPG11, EPHA4, KCND3, ELOVL5, ACAD9, DYRK1A, UNC5C, MAPK8IP3, PRSS12, TANC1, PLCB4, SLC9A6, RIN3, MAPT, IL6ST, PLEC, PRKAA2, RGS14, SLC1A1, HTT, THY1, CACNA1C, LRP2, ADCY8, GNAI2, GLRA1, RELN, CNNM1, INPP5F, N4BP3, MARK3, MARK1, GABRA2, SKOR1, FZD3, BDNF, KCNB1, KLHL1, RAB27A, AZIN2, SYT7, MTOR, SLC4A8, NIN, AGO2, NF1, GNB3, ATXN10, FGF13, KCNK1, CPEB4* |
| **brown** | **CC** | GO:0036064~ciliary basal body | 56 | 0.89 | 0.00 | *C2CD3, TRAF3IP1, JADE1, PKD2, KIF17, ARHGAP35, GLE1, IFT52, PCM1, DZIP1L, RTTN, BBS7, KIFAP3, IFT57, BBS5, RAB8A, BBS4, TAPT1, CDKL5, IFT140, CCDC113, ARMC9, IFT80, WDR35, WRAP73, IFT88, EVC, DAAM1, FAM161A, RP2, IFT81, TTLL11, EFHC2, SDCCAG8, SPACA9, INTU, CFAP206, SPATA7, PKHD1, ODF2L, POC1A, POC1B, BBS2, CSNK1A1, IFT122, WDR11, CCDC88A, AHI1, NEDD1, TTLL4, KIAA0586, DLG5, CENPJ, CDK10, CCDC66, TTLL9* |
| **brown** | **CC** | GO:0031901~early endosome membrane | 37 | 0.59 | 0.00 | *SH3GL3, CD274, ABCB6, ZFYVE9, SLC1A1, PSEN1, LDLRAD4, CLCN3, TMEM163, EGFR, GGA2, SNX3, INPP5B, GGA1, SNX1, GGA3, SNX2, WDR91, ATP9A, APPL2, APPL1, VTI1B, CLVS1, EPHA4, OSBPL6, ATP11B, OCRL, DNAJC13, STAM, SORL1, TPCN1, PIKFYVE, WDR81, RAB14, HGS, STAM2, CFTR* |
| **brown** | **CC** | GO:0034451~centriolar satellite | 46 | 0.73 | 0.00 | *PXK, C2CD5, TMEM63A, C2CD3, DYSF, NLRC3, KLHL12, AK5, PIK3R5, TAF1D, ODF2L, PCM1, PDZD2, KIF5B, KIAA0753, UBN1, NEK1, PCNT, CEP97, BBS5, RAB11FIP3, PIBF1, BBS4, DLGAP5, ZNF365, SPAG9, CEP68, RBM39, CENPU, YTHDF2, NEK6, CCDC14, CCDC113, PLK1, DDHD2, TAP1, HAUS6, HOOK3, PAX2, IFT43, SASS6, ITGB1BP1, LRIF1, CCDC66, TEK, SDCCAG8* |
| **brown** | **CC** | GO:0005783~endoplasmic reticulμm | **153** | 2.43 | 0.00 | *ANKRD13C, KCNG3, SLC27A1, SLC35B4, ATP8A1, PKD2L1, FAIM2, AQP5, ZFYVE1, CREB3L1, ALKBH1, KDR, TMEM38B, KIFAP3, TP63, SEC63, WLS, CUBN, SLC30A9, FBXW7, ACSL1, PRKCE, ANGEL1, ALG2, P3H2, DNAJB12, ATP11C, ACSL5, ATP11B, PRKCA, DNAJB14, VASH1, ACSL3, LEMD2, FNDC5, CPED1, DPM1, RNF128, PNLDC1, KCNQ1, COL4A3, FKBP9, KCTD17, TRAPPC3L, GRIA1, UBIAD1, CALCRL, SLC35D1, KLHL14, FUT10, YTHDC2, TMTC3, TMTC2, THADA, TAPBPL, PKHD1, PGS1, GRIN2A, SPAST, TMEM247, AGMO, KDSR, PSMF1, PROM1, CCR10, FDFT1, UVRAG, NUS1, NDFIP2, POU2F1, HACE1, ATP8B1, CAV1, Pμm3, CCDC88A, DIAPH2, TBL2, EHD4, ARMC10, TMEM117, TMCC3, TMCC2, PSMG1, ACO1, FGFR4, CNIH4, CPEB4, SRPX, FKBP10, PHTF2, FLT3, CPQ, FKBP14, ADPGK, RASGRF2, CD3E, ARHGAP5, ELAVL1, PHTF1, PANX1, GSG1, NCK2, DNM1L, ADAMTS9, UTP15, VWF, MAMDC2, ZDHHC13, ELOVL6, ZDHHC14, BACE2, LSG1, DNAJC1, PEX3, TRAPPC6B, CANX, ITGA8, ACSBG1, PIGL, NBAS, MGRN1, ATP10D, RAP1GDS1, LAMA3, MOSPD2, GDPD1, SEZ6, ATP10B, HTT, ASB11, FURIN, LRP2, LMAN1, FAM172A, GPAT4, SV2A, UBQLN1, STOM, HS1BP3, APBB2, AGA, FKTN, PTPN1, RIC3, USP25, YIPF4, TMEM64, UBE2G2, SGPP1, ARCN1, AQP11, PLCD4, PTPN2* |
| **brown** | **CC** | GO:0048471~perinuclear region of cytoplasm | **129** | 2.05 | 0.00 | *ANKRD13C, RAB3B, CYFIP1, RAB3C, ANKRD13D, CCNT2, CSF1, FMR1, WWC1, AQP2, SLK, GOLGA1, TRAPPC12, MAEL, ROS1, CDK5RAP2, SLC30A7, FBXW7, MAP1S, PRKCE, STX8, ANGEL1, STX7, ALG2, TSC2, LMTK2, ACSL3, SHTN1, KIF20B, DGKI, CERKL, BCL10, HSP90B1, MTDH, PKHD1, FXR1, CTIF, SPAST, UBR5, ATP6V0A2, PLCG2, TP53BP2, ABL1, PSMF1, ATP6V0A1, VTI1B, LYN, HSPA8, NDFIP2, NDFIP1, BDNF, RHBDD2, CAV1, PTCH1, AZIN2, INHBA, SNF8, COPS3, EHD4, NF2, FBXL5, PICALM, MTMR14, USP33, FAF1, ARHGAP1, PTPRM, BICD1, SH3RF1, ATP7B, TLK2, EPG5, CTLA4, HIF1AN, DNM1L, ATP7A, CD34, APC2, EXOC8, AXIN1, RANGAP1, RAD51C, BANK1, TRAF6, RAPGEF2, ITGB1BP1, LCP1, DEPDC5, RNFT2, SEC23A, SRC, USO1, GDPD1, SEZ6, HTT, ATP2C2, EGFR, SCEL, RAB40B, TNKS2, VPS53, NOCT, ALOX5, UBQLN1, PTK2B, VPS54, ATP9B, ATP9A, CHGA, ARFGEF1, ARFGEF2, NOS2, GALNT2, SORT1, GALNT1, HIP1R, MYO1B, PIKFYVE, P2RX4, APC, VAPA, KCNS2, KLHL7, NEDD4, TAOK1, TAF8, AQP11, PKN2, ATXN10* |
| **brown** | **CC** | GO:0014069~postsynaptic density | 46 | 0.73 | 0.00 | *BMPR2, RGS14, CLSTN1, ITPR1, ABHD17C, ADCY8, EPS8, GRM3, PCLO, CDH2, MAP2, DVL1, EPB41L3, TMEM108, PDE4B, DLGAP1, MAP4, BSN, SRGAP2, PAK3, CAMK2G, PAK2, SPTBN1, KPNA1, CAP2, EGLN1, NTRK2, HOMER1, HOMER2, PDPK1, PLEKHA5, DTNBP1, TSC2, ADAM10, TSC1, KCNAB2, ARHGAP32, ARHGEF9, DLG2, PLCB4, ABHD17B, DNAJC6, RHEB, DLG5, PKP4, CPEB4* |
| **brown** | **CC** | GO:0030424~axon | 50 | 0.79 | 0.00 | *NRP1, HTR2A, PTPRK, SLC6A1, GRM3, ALCAM, CALB1, KIF13B, KIF1B, KIF21A, DIP2B, BSN, EPHB2, VSTM5, SPG11, KIRREL3, EPHA4, DYRK1A, KCNAB2, GABRG2, UHMK1, SHTN1, BIN1, RIN3, PLEC, PRKAA2, MYCBP2, HTT, LPAR3, PSEN1, LRP2, ADCY8, GDPD5, FXR1, ABR, INPP5F, SNCG, GABRA2, FZD3, HOMER1, SYT1, BDNF, KCNB1, IRX3, AZIN2, MYO1D, NF1, GHRL, ADGRL2, ADGRL3* |
| **brown** | **CC** | GO:0005802~trans-Golgi network | 47 | 0.75 | 0.00 | *LRRK2, GCNT1, GCC2, FURIN, PIK3C2A, ATP2C1, BICD1, GGA2, ARFRP1, CCDC91, GGA3, CNST, VPS53, GOLGA1, MYO18A, VPS54, SNX9, DENND5A, RAB6A, ATP7A, ATP9B, ATP9A, CLASP2, WLS, CLVS1, SLC10A7, STX8, AZIN2, OCRL, NMNAT2, SCAMP1, FAM91A1, AP4B1, SORL1, IGF2R, WDR11, KLHL20, PLEKHA8, GOLPH3, PREPL, RAB14, RAB38, BIRC6, PRKD1, TBC1D23, CHST4, SLC24A5* |
| **brown** | **CC** | GO:0016605~PML body | 30 | 0.48 | 0.00 | *RB1, BLM, SATB1, PTEN, SMC6, RNF4, TRIM8, ZMYM2, TP53INP1, TOPBP1, NBN, PPARGC1A, IKBKE, N4BP1, RNF111, MORC3, ATRX, NSMCE2, SENP2, HIPK1, SIRT1, PIAS2, HIPK3, MTOR, PML, PIAS1, KLHL20, HIPK2, WDFY3, ATR* |
| **brown** | **CC** | **GO:0032991~macromolecular complex** | **97** | 1.54 | 0.00 | *N6AMT1, ZFYVE9, CCP110, NR3C1, CLDN1, IKZF4, CNST, CHEK1, ZC3H12A, CEP250, CAPN3, CEP97, RNF111, SH3GLB1, PDGFRA, MEF2C, CXADR, MITF, VRK2, CAMKMT, STIM1, ORAI1, VCL, KMT2E, PXK, EPB41, ILDR1, MTMR9, FOXO3, LTBP1, HSP90B1, STK3, MIER3, MIER2, UBR5, ABL1, STAP1, SKIL, LYN, NEK6, ARHGAP29, MLLT10, TFCP2, SPATS2L, ALK, UFL1, AKAP10, PCM1, TRPS1, XPO6, C1QTNF6, DNM1L, DTX3L, RIPK2, NCOA3, CCDC113, SORD, KIF24, WIPI2, PARP9, CKAP5, PAX2, PARD3B, METTL22, KIN, RABEP1, COL6A2, USP10, HTT, CXCR4, MFF, SNX31, TANK, PPM1E, PPM1F, STRN4, PRAM1, GGA1, GGA3, KRIT1, UBQLN1, RAD54L, RBBP6, STX2, UGGT2, UGGT1, SASH1, PTPN1, RBM39, GCH1, PEX14, TJP1, ITCH, NPHS2, RAB3GAP2, PKN2, RAB3GAP1* |
| **brown** | **CC** | GO:0030054~cell junction | 40 | 0.63 | 0.00 | *PTPRR, NLRX1, EPB41, CXCR4, PTPRK, SLC7A2, EGFR, DPP4, ARHGAP21, HOXC5, TP53BP2, HMCN2, PAK6, HMCN1, PCDH1, DCAF13, CDK5RAP2, IP6K2, MFAP3L, CEP68, PDGFRA, MIOS, KDF1, SLC16A10, ABCA7, PARD3B, DDB2, NUDT9, TSPAN15, FER, TBC1D2, RAD51C, NFIA, CCDC88C, ABI1, GID8, FAT1, RAPGEF2, CCDC66, DCP2* |
| **brown** | **CC** | GO:0005604~basement membrane | 29 | 0.46 | 0.00 | *COL18A1, LAMA2, LAMC3, TNC, LAMC2, LAMC1, EFNA5, NID2, ACAN, EGFLAM, ADAMTS1, HMCN2, HMCN1, TINAG, P3H2, FN1, CASK, NPNT, COL2A1, CCDC80, COL7A1, COL4A3, MMRN2, COL4A5, VWA2, ITGA6, FREM2, MATN2, FBN1* |
| **brown** | **CC** | GO:0000781~chromosome, telomeric region | 36 | 0.57 | 0.00 | *DCLRE1B, PIF1, RTEL1, BLM, KDM1A, TNKS, ZBTB48, RNF8, SMC6, BRCA2, PPP1CB, ORC4, HMBOX1, WRN, SMCHD1, ORC1, ORC2, CHEK1, DHX36, NBN, TP53BP1, UPF1, XRCC5, ATRX, NSMCE2, ZBTB10, THOC1, LRWD1, THOC5, PML, RAD51D, RAD17, POT1, ATM, DNA2, LRIF1* |
| **brown** | **CC** | GO:0030136~clathrin-coated vesicle | 18 | 0.29 | 0.00 | *VPS33A, HIP1R, OCRL, STON2, PIK3C2A, GGA2, SNX3, MYO6, DVL1, FCHO2, VPS41, NμmB, VPS11, SH3BP4, GPR107, SNX9, CLVS1, PICALM* |
| **brown** | **CC** | GO:0031410~cytoplasmic vesicle | 45 | 0.71 | 0.00 | *USP14, DENND1B, ATP8A1, TRAF3IP2, MAPKAP1, ARRB1, HTR2A, ATP2C2, FNBP1L, PKD2, ELAVL1, BICD2, SPRED2, SPRED1, SH3TC2, RUSC2, ADAMTS1, SLC17A5, UBQLN1, MAN1A1, N4BP3, SPG11, MPDZ, KIAA0319L, AGFG1, PDGFRB, SLC30A7, SLC30A9, GCH1, MYOC, PDPK1, CADPS, EMP2, FAM91A1, PRSS12, WDR11, CCDC88A, TBC1D2, RP2, FES, BTK, AMOTL2, ATM, AMOTL1, TBC1D23* |
| **brown** | **CC** | GO:0030659~cytoplasmic vesicle membrane | 26 | 0.41 | 0.00 | *C2CD5, DYSF, HTT, SNX33, AQP5, AQP2, TMEM67, PDE6D, KIF1B, SNX9, AP3S1, SLC18A1, SLC12A2, GABRA2, GABRA1, FZD6, MYOF, ENTPD7, GABRG2, APPBP2, KCNQ1, SCNN1B, TBC1D24, SPIRE2, VOPP1, SPIRE1* |
| **brown** | **CC** | **GO:0005856~cytoskeleton** | **88** | 1.40 | 0.00 | *ARHGAP39, FNBP1L, LMOD3, SGCE, EPB41L4A, SGCD, PSTPIP2, ZC3H12A, NOS1, PLEKHN1, JAK2, CEP290, TRIM67, JAK1, SLC30A9, FNBP1, RIPK2, PRKCE, DYRK1A, ANK2, FRMD4A, IQCD, FRMD4B, KCNAB2, ENAH, SHTN1, FRMD5, PREPL, FRMD6, FRMD7, BIN1, PICK1, EVL, TLN2, TNIK, UTRN, DRC7, PLEKHH3, ROCK1, EPB41, PXN, PPL, GLG1, FGD2, FGD3, EPB41L5, FGD4, FGD6, SNTG1, EPB41L2, EPB41L3, PDE6D, PACSIN2, KRIT1, MAPK1, PTK2B, STOM, CTNNA3, GAS2L1, WASF2, PLEKHH1, CTNNAL1, WASF3, FARP2, EVC2, FARP1, MICAL3, HIP1R, KLHL3, PARVA, ARHGAP26, PTPN13, PTPN14, RSPH3, KITLG, PRC1, KATNBL1, ABI1, TBCE, SPIRE2, TRIM36, SPIRE1, NF2, TACC2, PTPN4, ACTR10, CDC42SE2, PTPN3* |
| **brown** | **CC** | GO:0015629~actin cytoskeleton | 51 | 0.81 | 0.00 | *FKBP15, ARHGAP35, RAB22A, MYLK3, BAIAP2L1, KNTC1, TOPBP1, MYOZ1, MYOZ2, NDC1, PPP1R12A, NCOA5, DAPK1, SWAP70, ALG2, MTSS1, CTTNBP2NL, ADAM17, PEAK1, RARA, EPHA3, DGKH, DDR2, CD274, KLHL14, NEXN, BARX2, ACACA, ARHGAP21, PDLIM3, ARSJ, ABL1, CTNNA1, STK38L, PDLIM5, APBB3, CORIN, SVIL, TRMT10A, CARMIL2, KLHL2, EFR3B, TTC17, POU6F1, MSRA, ARHGAP32, FER, KANSL2, INTS6, CENPQ, RAB5A* |
| **brown** | **CC** | GO:0005637~nuclear inner membrane | 26 | 0.41 | 0.00 | *AHCTF1, NUP205, NUP107, ITPR1, NUP160, GLE1, NUP85, TPR, NUP88, NUP43, NUP214, NDC1, SMAD3, NUP155, LEMD3, LEMD2, SIRT1, TERB1, ERN1, NEMP2, UNC50, NUP35, NUP54, NEMP1, NUP58, NUP37* |
| **brown** | **CC** | GO:0097431~mitotic spindle pole | 15 | 0.24 | 0.00 | *MAPKBP1, TNKS, PLK1, RMDN3, SMC6, RMDN1, SMC3, EML1, STAG1, NIN, FAM161A, KATNA1, KIF20B, GIT1, CDK5RAP2* |
| **brown** | **CC** | GO:0005911~cell-cell junction | 39 | 0.62 | 0.00 | *ITK, PTPRU, USP53, CTNND1, SLC2A2, IQGAP1, PIK3R1, PKD2, CD3E, FLRT2, SV2A, NPHP1, EPB41L3, CSK, PLCG1, WASF2, PAK2, ACTR3, PRKCH, TRPC4, ACTN1, PLEKHG5, HEG1, THEMIS, GAB1, GRHL2, PLEKHA7, P2RX7, IGSF11, ADAM17, PIKFYVE, PTK7, KIT, GRB2, LCP2, PRKD1, TEK, FGFR4, SDCCAG8* |
| **brown** | **CC** | GO:0001726~ruffle | 27 | 0.43 | 0.00 | *ROCK1, MTMR14, ASAP3, ARHGAP18, IQGAP1, MTM1, FGD2, LIMA1, TRPM7, WASF2, APPL1, PDGFRB, CARMIL2, ACTN1, FRMD4B, MKLN1, KIF18A, ACAP2, CDK6, CTTN, RASA1, ITGB1BP1, CSPG4, LCP1, TLN2, NF2, RAB5A* |
| **brown** | **CC** | **GO:0005739~mitochondrion** | **180** | 2.86 | 0.00 | *ANKRD37, ISCA1, OSGEPL1, ARID4B, ABAT, NR3C1, ZFYVE1, GLS, THG1L, ELK3, ALKBH3, STK11, MTRF1, ALKBH1, NARS2, FDXR, CDK5RAP1, SACS, CPNE3, SLC25A42, SLC25A44, FBXO7, NUDT13, EARS2, STARD3, RALBP1, MECR, AGTPBP1, MIGA2, MIGA1, FBXW7, ACSL1, PRKCE, TANGO2, RSAD1, MRPS18A, ACSL5, GTPBP8, ATG13, ERN1, TMEM135, WDR81, PPA2, GPD2, RAB38, ALDH7A1, PIF1, NLRX1, DIABLO, DDX1, XPC, SPATA7, PGS1, BCL2L11, BLOC1S2, HIVEP1, STAP1, RIPK1, RAB11FIP3, PDZD8, PPARGC1B, DECR1, NDFIP2, SMURF1, BDNF, ADHFE1, NRDC, IDH2, AZIN2, BRAF, GPN1, FANCG, NFKB1, ESR2, DHFR, ZBTB6, P4HA1, STAR, NDUFAF7, CAPRIN2, SFXN4, ACO1, ALDH18A1, XAF1, TRMU, GFM1, SLC44A1, FASTKD1, GFM2, SLC44A2, LRRK1, SNAP23, RMND1, IDE, MRPL37, ECSIT, IFIH1, PPP3CA, NADK2, AKAP10, NNT, PCF11, KYNU, FLVCR1, KIF1B, ME2, NOS1, JARID2, SULT4A1, AASS, DLGAP5, GIT1, TOMM70, USP48, ARG2, NCBP1, PARP1, SORD, DARS2, AP3B1, PARP9, QRSL1, SIRT1, RPUSD4, VWA8, MRPS9, ALDH5A1, RAD51C, IVD, CRY2, L2HGDH, MICU1, MTRF1L, FXN, GTF3C4, STX17, RAP1GDS1, HEATR1, AMBRA1, PPM1K, TRAK1, MRM1, NAPG, TRAK2, MRM3, TMEM71, MAPK9, SLC25A28, MTHFD1L, OXCT1, NOCT, MAPK1, STOM, HS1BP3, BCO2, DISC1, DCPS, PAK5, PTCD3, METAP1D, TIMMDC1, PTCD2, EYA2, TXNRD2, NUBPL, MDH2, MACC1, HIP1R, GATB, TCAIM, MAPK14, SUGCT, GSTZ1, NUDT9, GOLPH3, RPS6KB1, XPNPEP3, SLC25A32, COQ8A, TRMT61B* |
| **brown** | **CC** | GO:0030496~midbody | 46 | 0.73 | 0.00 | *ZNF330, RALB, MTCL1, CTNND1, RNF8, HEPACAM2, NAT10, IQGAP1, HSP90B1, GNAI2, FLCN, ZFYVE26, ALKBH4, SPAST, KATNA1, ANKRD54, ECT2, MYH10, RAB11FIP3, RAB8A, APC2, SH3GLB1, UVRAG, USP8, NUDC, MBD5, PTCH1, PLK1, DTNBP1, ANXA11, UHMK1, AGBL5, ANLN, TBCK, LYRM1, KATNAL1, PRC1, BIRC6, PKN2, PIK3C3, MITD1, MAPRE3, KIF20B, CPEB3, CEP44, ARL8B* |
| **brown** | **CC** | **GO:0000785~chromatin** | **68** | 1.08 | 0.00 | *ATF2, AHCTF1, SETD3, KDM1A, BNC2, RIF1, DSCC1, AKAP8L, ARRB1, NUCKS1, SMC3, NIPBL, CHAF1B, PPP4R2, TRPS1, CREB3L1, CHEK1, MAEL, TP63, GABPA, UPF1, FOXD3, MYOCD, TIPIN, ESCO1, IST1, NCOA3, USP3, TCF12, RFX3, PPP4R3B, LEMD2, NCOR2, MSH6, MAU2, NCOR1, PPARD, ANKRD17, CASZ1, UBR2, PDS5B, PDS5A, PRDM10, ORC5, ORC3, NRIP1, HSF4, DVL3, STAT6, SRPK2, TCF7L2, CBX7, SMAD4, SLF2, SMAD3, KDM4C, PLK1, SMARCA5, HMGA2, SMARCA1, WAPL, PHOX2A, FER, STAG1, NEDD4, SPRTN, TAF4, NFE2L2* |
| **brown** | **CC** | GO:0032587~ruffle membrane | 27 | 0.43 | 0.00 | *KANK1, C2CD5, SRC, WWC1, PLEK, EGFR, EPS8, EPB41L5, PSD2, SNTG1, MYO6, PSD3, PLCG2, PACSIN2, PIP5K1A, ITGAV, PLCG1, APPL2, PACSIN1, CLASP2, PLA2G4F, HIP1R, SH3YL1, ADAM17, APC, DLC1, PLEKHO1* |
| **brown** | **CC** | GO:0030027~lamellipodiμm | 50 | 0.79 | 0.00 | *CYFIP1, SCYL3, ITSN1, DYSF, PTPRM, WASL, PKD2, SH3RF1, CDH2, PLCE1, ACTR3, PLEKHG5, TSC1, UNC5C, SHTN1, CTTN, PALLD, ITGB1BP1, EVL, ARHGEF6, PLEKHH2, ROCK1, IQGAP2, SNX1, SNX2, ABLIM3, PIP5K1A, PTK2B, CTNNA3, PLCG1, STX2, WASF2, WASF3, CARMIL1, CARMIL2, PARVA, CDC42BPB, CDC42BPA, PTPN13, CCDC88A, FER, ARHGAP31, KITLG, APC, ABI1, FAT1, CTNNB1, PKN2, NF2, AMOTL1* |
| **brown** | **CC** | GO:0042995~cell projection | 25 | 0.40 | 0.00 | *CLSTN1, ITSN1, LAPTM4B, GLRA2, PCLO, GLRA3, SH3PXD2A, SH3PXD2B, PTK2B, CEP290, IMPG1, IMPG2, AGFG1, MAGI1, AFAP1L1, ODF2, PDPK1, MAP1S, SLC2A13, COQ6, ENAH, FCHSD2, TBC1D24, OGT, KIZ* |
| **brown** | **CC** | GO:0000776~kinetochore | 33 | 0.52 | 0.00 | *PHF2, AHCTF1, NUP107, MEAF6, FBXO28, TTK, NUP160, NSL1, CENPC, NUP85, TPR, TRAPPC12, NUP43, TP53BP1, CLASP1, DYNC1I1, CLASP2, NDEL1, PPP1R12A, CSNK1A1, SS18L1, LRWD1, KNL1, RANGAP1, CKAP5, KAT2B, KIF18A, APC, CENPH, CENPI, CENPO, NUP98, NUP37* |
| **brown** | **CC** | GO:1990391~DNA repair complex | 7 | 0.11 | 0.00 | *KDM1A, ATM, TP53BP1, BRCA1, BRCA2, PALB2, RCOR1* |
| **brown** | **CC** | GO:0001650~fibrillar center | 43 | 0.68 | 0.00 | *DCLRE1A, NUFIP1, DDX46, CDCA7L, HEATR1, USO1, MLLT1, WDR43, ACACA, MALT1, MTDH, NUAK1, SESN1, EXOSC8, NRIP1, LEO1, PAK6, SNRPB2, E2F5, JAZF1, IP6K2, NOP58, RSAD2, FOXJ2, SMARCA5, SAMD4A, PLRG1, URB1, PTPN13, TRERF1, SIRT1, PEX14, WDR33, ARHGAP32, KLF6, NFIB, PSPC1, KIT, SNAI1, TAF4B, POLR1E, MAP3K14, RAI14* |
| **brown** | **CC** | GO:0055037~recycling endosome | 23 | 0.37 | 0.00 | *GRIA1, ARFGEF2, STX8, STX7, LMTK2, ATP11B, SNF8, COMMD1, AQP2, MCOLN2, RAB10, RAB14, SLC9A6, INPP5F, AP1G1, GPR161, SLC9A9, DENND6A, TUBGCP4, LDLRAP1, AVL9, KCNK1, DYNC1I1* |
| **brown** | **CC** | GO:0005905~clathrin-coated pit | 17 | 0.27 | 0.00 | *CUBN, BMPR2, DGKD, SORT1, ITSN1, HIP1R, LRP2, VLDLR, NECAP1, INPP5F, MYO6, FCHO2, NμmB, SH3BP4, AP1S3, DNM1L, REPS1* |
| **brown** | **CC** | GO:0005929~ciliμm | 30 | 0.48 | 0.00 | *NOTCH2, STOML3, ANKS3, TUBD1, CBL, TXNDC15, IFT74, FLCN, KIF3B, TTC21B, SUFU, P2RY1, PDZD7, DYNC2H1, PDGFRA, RABGAP1L, MYOC, PTCH1, CAV1, WDR19, KIF27, RAB10, RSPH3, ACE2, IFT43, GPR161, HYLS1, ALPK1, CLUAP1, GPR19* |
| **brown** | **CC** | GO:0043025~neuronal cell body | 47 | 0.75 | 0.01 | *GRIA1, SLC22A3, PRKAA2, KLHL14, CPNE5, SLC1A1, SEZ6, SLC1A3, PTPRK, GDPD5, SLC6A1, PTPRF, PURA, CNNM1, ALCAM, CALB1, INPP5F, PSD2, TNN, KIF5C, DPYSL5, SNCG, LRIG2, EPHB2, MYH10, AGFG1, SLC12A2, GABRA2, FZD3, SKOR1, SLC12A5, ELOVL5, PMM2, KLHL1, UNC5C, SYT7, MYO1D, P2RX7, TANC1, GRIN3B, FRMD7, CNTN2, RIN3, MAPT, ATXN10, IL6ST, PICALM* |
| **brown** | **CC** | GO:0045111~intermediate filament cytoskeleton | 19 | 0.30 | 0.01 | *EXD2, BCAS3, CARMIL2, ZNF131, MTRR, USP10, PRKCE, CTNS, SMG7, EVPL, SMARCA2, CLK3, PJA2, TTBK2, PKN2, DISC1, PHLDB2, MDN1, PLEC* |
| **brown** | **CC** | GO:0005640~nuclear outer membrane | 19 | 0.30 | 0.01 | *NUP214, NDC1, AHCTF1, NUP205, NUP107, RETSAT, NUP155, SYNE3, NUP160, SYNE1, GLE1, NUP85, TPR, NUP35, NUP43, NUP88, NUP54, NUP58, NUP37* |
| **brown** | **CC** | GO:0005938~cell cortex | 23 | 0.37 | 0.01 | *C2CD5, NDFIP1, EPB41, CTTNBP2, CAV1, PXN, KDF1, HIP1R, ITPR2, TSC1, EPS8, ARHGAP32, CNKSR1, MKLN1, PARD6B, ITCH, NEDD4, SPIRE2, HMCN2, PRKD1, HMCN1, MYH10, FGFR2* |
| **brown** | **CC** | GO:0008023~transcription elongation factor complex | 9 | 0.14 | 0.01 | *NUFIP1, ICE1, ICE2, ELP2, MLLT1, ERCC6, MLLT3, ELL2, AFF1* |
| **brown** | **CC** | GO:0015630~microtubule cytoskeleton | 25 | 0.40 | 0.01 | *ZBTB49, MYCBP2, SRA1, LYST, NUAK1, ATXN7, DVL1, TAF1A, TEKT1, MAP7, TEKT3, MFN2, SPTAN1, SS18, PRKCI, PCIF1, MTUS1, DTNBP1, GTF2F2, NISCH, FER, FES, TAOK1, VPS41, CCSER2* |
| **brown** | **CC** | GO:0030014~CCR4-NOT complex | 8 | 0.13 | 0.01 | *CNOT4, CNOT6, CNOT11, CNOT10, CNOT8, CPEB3, CNOT9, PATL1* |
| **brown** | **CC** | GO:0031080~nuclear pore outer ring | 7 | 0.11 | 0.01 | *AHCTF1, NUP107, NUP85, NUP43, NUP98, NUP160, NUP37* |
| **brown** | **CC** | GO:0043235~receptor complex | 34 | 0.54 | 0.01 | *NOTCH2, VIPR1, LRP1, GPR119, GPR63, LRP5, ITPR2, PKD2L1, VLDLR, LRP2, LRP8, EGFR, IGF1R, RIPK1, IMPG2, ABCG8, CUBN, NTRK2, ABCG5, SMAD3, TRPC1, ACVR2B, ACVR2A, TGFBR2, TGFBR3, PEX5L, PTPRB, PTPRA, CD8A, FSHR, NBR1, CAPRIN2, ROR1, PLA2R1* |
| **brown** | **CC** | GO:0005930~axoneme | 24 | 0.38 | 0.01 | *DYNC2H1, IFT140, TRAF3IP1, DNAAF1, TULP3, AMBRA1, CFAP206, ATG14, WDR35, KIF17, DNALI1, AK8, GLI3, SPATA7, GLI2, ARL13B, DZIP1L, MNS1, KIFAP3, CFAP43, IFT57, BBS5, GAS8, ATG5* |
| **brown** | **CC** | GO:0035861~site of double-strand break | 20 | 0.32 | 0.01 | *ATF2, ACTR2, SMARCAL1, SLF1, SLF2, PARP1, RIF1, XRCC4, RNF8, SMC6, UFL1, HELB, SMCHD1, RNF169, RNF138, APLF, POLL, NBN, TP53BP1, RAD18* |
| **brown** | **CC** | GO:0043005~neuron projection | 26 | 0.41 | 0.01 | *CYFIP1, TPH2, AHCYL2, TENM4, KLHL14, CPNE5, SNAP23, SLC1A3, IQGAP1, PTPRF, GRIP1, UFL1, GRIN2A, SV2A, KIF5B, SLC12A2, RAP1GAP2, GABBR2, SLC12A5, ANKRD27, BRAF, TSGA10, ABI1, CDH13, NF2, CPEB3* |
| **brown** | **CC** | GO:0032039~integrator complex | 9 | 0.14 | 0.01 | *NIPBL, INTS1, INTS10, INTS2, INTS4, INTS7, INTS6, INTS9, INTS8* |
| **brown** | **CC** | GO:0016514~SWI/SNF complex | 13 | 0.21 | 0.01 | *SS18, SMARCD1, SMARCD2, PBRM1, SMARCC1, SMARCD3, BCL11A, ACTL6A, ARID1A, ARID1B, SMARCA2, BCL7A, ARID2* |
| **brown** | **CC** | GO:0005912~adherens junction | 30 | 0.48 | 0.01 | *BMPR2, SNAP23, PTPRM, CD99L2, EFNA5, ADD1, EPB41L5, CDH2, CCDC85A, CTNNA1, CTNNA3, MAGI1, CXADR, JAG1, ADAM10, FRMD4B, MPP7, TJP1, MYO1E, AFDN, AHI1, FRMD5, APC, DLG5, FAT2, CTNNB1, NF2, NECTIN3, FERMT2, VCL* |
| **brown** | **CC** | GO:0000242~pericentriolar material | 11 | 0.17 | 0.01 | *NEDD1, PCM1, NIN, TNKS2, CEP152, CEP85L, NEK1, CEP192, HOOK3, BBS4, CDK5RAP2* |
| **brown** | **CC** | GO:0005887~integral component of plasma membrane | **119** | 1.89 | 0.01 | *EPHB6, SCARB1, SLC27A1, PKD2L1, AQP4, AQP5, AQP2, HTR4, CLDN1, TMEM266, CDH4, HTR7, CDH2, KDR, SLC16A7, SLC16A3, KCNH1, CDON, IFNAR2, ABCG8, PDGFRA, EPHA4, ABCG5, EPHA7, SLC6A19, MIGA2, ATP11C, GABRG2, SCNN1G, ADAM17, ADCY9, SCNN1B, SCNN1A, ORAI1, EPHA3, CFTR, IFNAR1, NOTCH2, PLGRKT, NPR2, ABCB5, SLC1A1, LPAR1, SLC1A3, APCDD1, SLC6A20, ANKH, FLRT2, BTLA, PLXNA2, DRD3, SLC19A1, ASIC1, ABCA1, ATP8B2, ATP8B1, CAV1, SLC16A12, SLC16A10, SELP, PLXNB1, FGFR4, SLC26A3, LRP12, FGFR2, ALK, TENM1, BMPR2, TENM4, TNFRSF13B, GRIK3, HTR2A, PTH1R, SLC6A1, PKD2, SLC6A2, SLC6A4, MMP24, BOC, CTLA4, NEO1, TMC1, TNFRSF18, SLC2A13, TMC7, ANO6, TMEM150C, MMP16, TLR2, HBEGF, DDR2, NPSR1, ATP10D, MOSPD2, GLRA1, GLRA2, GLRA3, P2RY1, LRRC8D, STOM, LRRC8B, MPZL1, JAM2, NTRK2, SLC12A5, TRPA1, SEMA4D, GPR55, SYT13, DCBLD2, P2RX7, CD4, P2RX4, ESYT3, TEK, SLC28A1, LGR5, LGR4, SLC28A3* |
| **brown** | **CC** | GO:0016020~membrane | **156** | 2.48 | 0.01 | *ANKRD13C, PI4K2B, DGKE, DENND5B, SSC4D, STK11, CCND3, RIMS3, CCND2, CREB3L1, TOM1, DENND5A, BORCS7, SH3GL2, ACVR1, TMOD1, MAGI1, RALBP1, COG5, PRKCB, BROX, STX8, FIBCD1, COG3, HPCAL1, COG2, ANXA11, TSC2, FRS2, TSC1, DNAJB14, ANK2, NPNT, GAPVD1, SYTL4, RC3H2, CLIP1, BIN1, PRKD3, COL4A3, ELMO2, COL4A5, YME1L1, RAB38, PRKD1, DGKI, SEMA7A, CFI, PDGFB, MTMR4, MTMR7, RAP1B, GRK2, BCL2L11, RAP1A, PDGFD, PDGFC, RAB11FIP2, FAM83B, EDEM3, WSCD1, SUCO, NMI, CORO2B, ESR1, DNAJC16, PEX5L, RHEB, NDUFAF2, MKS1, PDE3A, PLCH1, FBXL2, HPSE, BMPR1B, MAP3K13, NOX1, BCAR3, FKBP10, FKBP15, LAMC3, ITGB2, LAMC2, SNX10, LAMC1, CLINT1, BICD1, GLI2, TNFSF13B, SPRED2, SPRED1, MAP1LC3C, PSTPIP2, PDE4B, BBS9, FLOT2, ITGAV, ERC1, JAK1, ARHGEF12, MAMDC2, SORD, PPP2R5A, RRAS2, TGFBR1, TGFBR2, ITPKB, PPM1B, MRAS, VSNL1, LRRC7, GORASP2, GORASP1, CNKSR3, RAPGEF2, BIRC6, PLEKHO1, BPI, PIGL, PLCB1, PLCB2, YAP1, AMER1, TGFBRAP1, LAMA3, SRL, NAPG, STX11, STRN4, GNA13, ZNRF1, ARFRP1, OGFRL1, PARD6B, VPS51, PALMD, GNA12, UBQLN1, APBB3, LMLN, RIC1, SEMA4D, ATRNL1, MDH2, DDHD2, VEGFC, LAMB1, ACVR2B, PLEKHA8, FIG4, TTC8, CD4, DNAJA4, ABI1, KRAS, COQ8A* |
| **brown** | **CC** | GO:0005635~nuclear envelope | 25 | 0.40 | 0.01 | *FAF1, RTCB, AGPAT3, RAB40B, TNKS2, SLC22A18, BNIP1, TNPO3, HTATIP2, TMPO, PARP1, FANCL, IST1, INSR, ANXA11, LMO7, VRK2, TMEM170A, MTOR, PLPP7, PARP11, CLIP1, NAPEPLD, WDFY3, MNS1* |
| **brown** | **CC** | GO:0005768~endosome | 39 | 0.62 | 0.01 | *RNF32, CDKN1B, CALCRL, FKBP15, CLTC, LPAR1, LRP2, PLD1, HTR4, MTMR4, THSD1, ZNRF1, SPAST, GRAP2, SNX25, KDR, FLOT2, ITGB8, RAB11FIP2, ATP9A, SNX6, CUBN, CD164, CAV1, ARRDC3, WDR72, AP1AR, RASSF9, AVPR1A, SYTL4, TGFBR1, BACE2, PLEKHM1, GRB2, BIRC6, KCNK1, ATP6V0D1, ATP6V0D2, DGKH* |
| **brown** | **CC** | GO:0005770~late endosome | 31 | 0.49 | 0.01 | *WDR48, ANKRD13D, SRC, DYSF, CXCR4, HTT, SLA2, CTSS, MTM1, TMEM25, FYCO1, ATP7B, MICALL1, MAPK1, ATP7A, UVRAG, HSPA8, RUBCN, ANKRD27, STX8, STX7, TMEM192, CTNS, NMNAT2, IGF2R, SLC9A6, RNF128, NBR1, RAPGEF2, VIPAS39, IFNAR1* |
| **brown** | **CC** | GO:0005657~replication fork | 12 | 0.19 | 0.01 | *WRN, RAD51D, RAD51C, UBE2B, BCL6, TEX264, XRCC2, CHEK1, NBN, TP53BP1, RAD18, PRIMPOL* |
| **brown** | **CC** | GO:0045335~phagocytic vesicle | 22 | 0.35 | 0.02 | *STX12, STXBP4, STX8, ZDHHC5, ANXA11, NOD1, AMBRA1, CLCN3, ATG14, MTOR, CTSS, RAB22A, SNX3, RAB20, RAB14, KIF5B, RAB38, EVL, SRGAP2, RAB8A, APPL1, RAB8B* |
| **brown** | **CC** | GO:0005819~spindle | 22 | 0.35 | 0.02 | *RB1, NUDC, DCUN1D5, RGS14, ANAPC7, MAP1S, CSNK1A1, SPICE1, ANXA11, HAUS3, SKA3, NR3C1, MZT1, SPAST, PRC1, SPECC1L, ATM, ANAPC5, MYH10, MAP2K5, NDEL1, PAFAH1B1* |
| **brown** | **CC** | GO:0097539~ciliary transition fiber | 7 | 0.11 | 0.02 | *NIN, ODF2, FBF1, CEP164, SCLT1, CEP83, CEP89* |
| **brown** | **CC** | GO:0034399~nuclear periphery | 7 | 0.11 | 0.02 | *NUP205, NUP107, MAP2, TPR, NUP98, MAPT, TELO2* |
| **brown** | **CC** | GO:0005874~microtubule | 59 | 0.94 | 0.02 | *CEP57, DYSF, KIF5C, CSPP1, KIF5B, TUBB1, TCP11L1, DNM1L, NDEL1, DYNC2H1, NUDC, MAP1S, KIF25, HAUS3, KIF23, SHROOM1, DNM1, MID2, SHTN1, DYNC1LI1, DYNC1LI2, CLIP1, KATNAL1, KIF2A, MAPT, MAPRE2, GAS8, PAFAH1B1, DYNC1I2, DNAH1, DNAH7, STAU2, DNAH8, TUBGCP2, DNAH5, TUBD1, DNAH9, IQGAP1, SPAST, KIF3B, MAP2, KATNA1, KIF3C, MAP4, DYNC1H1, DNAH17, TUBE1, HOOK3, TBCA, APC, TTLL1, TUBGCP5, TUBGCP6, MAP6D1, DYNLRB2, CCDC66, TUBGCP4, FGF13, TUBA8* |
| **brown** | **CC** | GO:0072686~mitotic spindle | 28 | 0.44 | 0.02 | *PHLPP2, EPB41, CUL3, CLTC, HNF4G, HEPACAM2, PKD2, SKA3, PKHD1, FLCN, TAF1D, CDC27, TPR, CEP170, MAP9, CLASP2, NUDC, KDF1, CDC7, WAPL, AGBL5, TBCK, NCOR1, YEATS2, TFDP2, TBL1XR1, CDC16, LSM14A* |
| **brown** | **CC** | GO:0017053~transcriptional repressor complex | 18 | 0.29 | 0.02 | *SPEN, PHF12, GMNN, ARID4A, LIN9, CORO2A, RBPJ, PRDM10, GLI3, NCOR2, NCOR1, TBL1XR1, MIER1, C1D, SP3, HEY2, RCOR1, JAZF1* |
| **brown** | **CC** | GO:0005664~nuclear origin of replication recognition complex | 6 | 0.10 | 0.02 | *ORC5, ORC4, ORC1, ORC3, ORC2, LRWD1* |
| **brown** | **CC** | GO:0098684~photoreceptor ribbon synapse | 6 | 0.10 | 0.02 | *CACNB2, HSPA8, CLTC, DNM1, PACSIN1, CPLX3* |
| **brown** | **CC** | GO:0044354~macropinosome | 6 | 0.10 | 0.02 | *CARMIL1, CARMIL2, CLIP1, ANKFY1, APPL2, APPL1* |
| **brown** | **CC** | GO:0005901~caveola | 22 | 0.35 | 0.02 | *SCARB1, BMPR2, TRPC4, SRC, IRS1, INSR, PTCH1, CAV1, MYOF, HTR2A, TFPI, LRP8, TGFBR2, LRP6, KIF18A, SMO, DLC1, CDH13, MAPK1, CDH15, NOS1, BMPR1A* |
| **brown** | **CC** | GO:0000922~spindle pole | 26 | 0.41 | 0.02 | *MTCL1, TUBGCP2, FBF1, NEDD9, CEP128, CSPP1, POC1A, POC1B, CEP95, KNTC1, SPDL1, DYNC1I1, MAPK14, CKAP5, LATS1, NEDD1, KATNAL1, KIF2A, ZW10, TUBGCP5, TUBGCP6, BIRC6, TUBGCP4, ALPK1, CEP44, CEP89* |
| **brown** | **CC** | GO:0008305~integrin complex | 12 | 0.19 | 0.02 | *ITGB5, ITGB4, ITGA2, ITGA2B, ITGB2, ITGA11, ITGA8, ITGAV, ITGA6, ITGB6, GPATCH8, ITGA9* |
| **brown** | **CC** | GO:0045180~basal cortex | 5 | 0.08 | 0.02 | *PHLDB1, PKD2, PHLDB2, CLASP1, CLASP2* |
| **brown** | **CC** | GO:0030991~intraciliary transport particle A | 5 | 0.08 | 0.02 | *IFT43, IFT140, TTC21B, IFT122, WDR35* |
| **brown** | **CC** | GO:0030056~hemidesmosome | 5 | 0.08 | 0.02 | *COL17A1, ITGB4, ERBIN, ITGA6, PLEC* |
| **brown** | **CC** | GO:0005776~autophagosome | 17 | 0.27 | 0.02 | *SRPX, VPS33A, HTT, WIPI2, ATG14, FYCO1, MAP1LC3C, TBC1D5, NBR1, TP53INP1, VTI1A, UBQLN1, VPS11, PIP4K2A, PIP4K2B, UBQLN4, ATG5* |
| **brown** | **CC** | GO:0036464~cytoplasmic ribonucleoprotein granule | 18 | 0.29 | 0.02 | *UPF2, BARD1, ROCK2, USP3, IQGAP1, GABPB1, HOXD10, CKAP4, GHR, EIF4ENIF1, MAP1LC3C, NFKBIZ, BLNK, POLI, PMS2, SNRPB2, MAPT, DYNC1I1* |
| **brown** | **CC** | GO:0016323~basolateral plasma membrane | 44 | 0.70 | 0.02 | *NDRG4, SLC47A1, B4GALT1, LRP1, HPGD, EPB41, PDGFB, STK39, NOD1, LIN7A, IDE, AQP2, ADCY8, ABCC10, CLDN1, EGFR, DSTYK, ATP7B, CDH2, STX2, CNNM2, ATP7A, SLC19A1, SLC13A3, CXADR, TRPC4, ITGA3, ABCC5, SLC16A12, ERBIN, CASK, SLC16A10, SCRIB, ATP2B1, ATP1B1, TSHR, SLC2A9, SLC4A7, KCNQ1, NμmB, CTNNB1, ORAI1, ITGA6, TEK* |
| **brown** | **CC** | GO:0097546~ciliary base | 13 | 0.21 | 0.02 | *NEK8, TRAF3IP1, MOK, TULP3, IFT122, DNALI1, IFT52, CFAP36, NPHP3, DISC1, PRKACB, RAB8A, SPACA9* |
| **brown** | **CC** | GO:0032839~dendrite cytoplasm | 8 | 0.13 | 0.03 | *ABHD12, PURA, KIF3B, LRRK2, FLOT2, KIF17, TRAK2, UHMK1* |
| **brown** | **CC** | GO:0000932~P-body | 22 | 0.35 | 0.03 | *DDX6, YTHDF2, RC3H1, PATL1, RC3H2, EDC3, SYNE1, EIF4ENIF1, PSMA6, MOV10, PAN3, AGO3, CNOT1, NOCT, BTBD2, AGO2, ZC3H12A, DCP1A, CNOT9, LSM14A, DCP2, TNRC6A* |
| **brown** | **CC** | GO:0045121~membrane raft | 37 | 0.59 | 0.03 | *STX12, STOML3, ITGB2, SLC1A1, FAIM2, FURIN, THY1, BCL10, EGFR, SLC6A4, DPP4, GRIP1, PGK1, KDR, STOM, TNR, PAG1, LYN, CXADR, ANGPT1, CAV1, EMP2, TGFBR1, TGFBR2, ADAM17, PIKFYVE, CD4, PTPRC, NPHS2, BTK, FAS, HPSE, TEK, IL6ST, RAB5A, CARD11, TLR2* |
| **brown** | **CC** | GO:0031965~nuclear membrane | 47 | 0.75 | 0.03 | *ANKRD17, GTF3C3, RIF1, MRPS14, TNKS, PHF20, ITGB4, WDR3, ZBTB1, TXLNG, YEATS4, DTX2, PCM1, BRIP1, SPAST, GRK5, SμmO1, ALOX5, SPIN1, EPC1, KPNA4, TMEM38A, CLCC1, SCAI, RAP1GAP2, OSBPL8, OSBPL6, GCH1, OSBPL3, DNAJB12, ALG14, PLRG1, RANGAP1, UTP18, SENP1, NUP93, INPP4A, ZC3HC1, NUDT9, RNF123, BCL2, NUP35, NUP98, TAF3, EPHA3, PAFAH1B1, BCL2L1* |
| **brown** | **CC** | GO:0005847~mRNA cleavage and polyadenylation specificity factor complex | 7 | 0.11 | 0.03 | *CPSF6, FIP1L1, ZC3H3, CPSF3, CPSF2, CSTF2, PIP5K1A* |
| **brown** | **CC** | GO:0010008~endosome membrane | 25 | 0.40 | 0.03 | *OCA2, VAC14, NCF4, ARHGAP1, VPS26A, TMEM165, SLC6A4, VPS53, TMEM108, ANKFY1, NDFIP2, NDFIP1, SORT1, VTA1, VPS13A, MCOLN2, IGF2R, MYO1B, EHD4, SLC9A6, TBC1D5, SLC9A9, PLEKHM2, VPS45, STEAP2* |
| **brown** | **CC** | GO:0031514~motile ciliμm | 23 | 0.37 | 0.03 | *DYNC2H1, BBS2, DRC7, INTU, IFT172, SORD, IQCD, PKD2, GLI2, IFT43, DAAM1, ARL13B, SCNN1A, NPHP1, TEKT1, TEKT2, CFAP36, DRC1, CFAP97, GAS8, BBS4, CEP89, CFAP77* |
| **brown** | **CC** | GO:0005801~cis-Golgi network | 13 | 0.21 | 0.03 | *SLC10A7, COG3, GOSR1, ANGEL1, AZIN2, PIK3R1, HOOK3, BCL9, GOLGA5, MAN2A1, MAP6D1, TRIP11, FKTN* |
| **brown** | **CC** | GO:0005643~nuclear pore | 14 | 0.22 | 0.04 | *NDC1, NUP205, NUP133, NUP155, SENP2, NUP160, BICD2, GLE1, NUP93, NUP35, NUP88, NUP54, NUP98, NUP58* |
| **brown** | **CC** | GO:0009897~external side of plasma membrane | 53 | 0.84 | 0.04 | *B4GALT1, CD83, ITGA2B, AQP4, FASLG, IDE, CD3E, ANTXR2, ALCAM, CTLA4, ITGAV, ITGB6, CD34, CCR4, TMC1, PDGFRA, LAG3, ITGA3, ITGA2, HEG1, ITGA1, ADGRA3, TGFBR2, TGFBR3, SCNN1G, CD8A, SCNN1B, KIT, CDH13, ITGA6, IL6ST, CD274, SEMA7A, CXCR5, LRP2, CLCN3, THBS1, ENOX2, IL1RL1, NT5E, SCUBE1, TRPM8, ABCA1, IL31RA, ENOX1, SELP, P2RX7, CD4, CD40LG, PTPRC, IL2RB, CD28, BMPR1A* |
| **brown** | **CC** | GO:0005721~pericentric heterochromatin | 11 | 0.17 | 0.04 | *KDM4A, HELLS, UHRF2, ZNF618, ATRX, SMARCA5, SNAI1, LRWD1, NCAPD3, BAZ1B, CENPC* |
| **brown** | **CC** | GO:0009925~basal plasma membrane | 8 | 0.13 | 0.04 | *OSCP1, SLC12A2, SLC27A1, SLC23A1, AQP5, LDLRAP1, PKD2, MET* |
| **brown** | **CC** | GO:0035770~ribonucleoprotein granule | 6 | 0.10 | 0.04 | *FXR1, RPUSD4, DHX30, FASTKD2, TDRD7, GRSF1* |
| **brown** | **CC** | GO:0034464~BBSome | 6 | 0.10 | 0.04 | *BBS2, TTC8, BBS9, BBS7, BBS5, BBS4* |
| **brown** | **CC** | GO:0002102~podosome | 12 | 0.19 | 0.04 | *AFAP1L1, HNRNPK, SH3PXD2A, CTTN, BIN2, SRC, SH3PXD2B, LPXN, ASAP1, LCP1, PTPN12, VCL* |
| **brown** | **CC** | GO:0090575~RNA polymerase II transcription factor complex | 25 | 0.40 | 0.05 | *ATF2, THRB, MAX, GATA4, BACH1, MXI1, E2F3, STAT6, E2F6, DR1, POU2F1, STAT1, TCF12, ARNT, BATF, FOSL2, HIPK2, NR5A1, HOXB9, NR5A2, TFDP2, HAND1, RFX5, ATF6, NFE2L2* |
| **brown** | **CC** | GO:0005667~transcription factor complex | 39 | 0.62 | 0.05 | *PRKDC, SATB2, SRA1, LDB2, AATF, ETS1, EP300, E2F5, PKNOX1, PITX2, SMAD2, WWTR1, ARNT2, NCOA2, SMAD4, ZFHX3, SKOR1, TFAP2D, SMAD3, PARP1, ASCC1, EYA3, STAT3, RFX3, SMAD9, HNF1A, SNF8, ESR1, SMAD5, SMAD7, RBL2, MEIS1, NR6A1, KLF5, RBL1, SUB1, RARA, NAA15, CLOCK* |
| **brown** | **CC** | GO:0030139~endocytic vesicle | 14 | 0.22 | 0.05 | *CUBN, PLEKHG5, ITSN1, STX7, DYSF, LPAR1, LRP2, AVPR1A, PLD1, IGF2R, PIK3C2B, DPP4, RABEP1, RIN3* |
| **brown** | **CC** | GO:0009898~cytoplasmic side of plasma membrane | 14 | 0.22 | 0.05 | *DIABLO, PTPN22, ATP2C2, LITAF, ANK1, PPP3CA, TRAF3, TRAF6, PGM5, LDLRAP1, PTPN4, MYH10, BIRC2, PTPN3* |
| **brown** | **CC** | GO:0030992~intraciliary transport particle B | 9 | 0.14 | 0.05 | *IFT52, IFT74, IFT88, IFT81, TRAF3IP1, IFT172, IFT80, IFT57, CLUAP1* |
| **brown** | **CC** | GO:0032580~Golgi cisterna membrane | 15 | 0.24 | 0.05 | *B4GALT1, FUT10, UXS1, CSGALNACT2, GOLPH3L, ATP2C1, FUT4, FUT8, GOLPH3, CHSY1, B4GALNT3, B4GALT6, B4GALT7, B4GALT4, B4GALT5* |
| **brown** | **MF** | GO:0005524~ATP binding | **589** | 9.35 | 0.00 | *PI4K2B, CLPB, TESK2, SMC5, SMC3, ABCA12, SMC4, SMC2, TBK1, AKT3, AKT1, PIP4K2A, PIP4K2B, PRKACB, EARS2, DDX17, DDX18, PRKCI, PRKCH, CSNK2A1, DAPK1, PRKCB, PRKCE, DAPK2, STARD9, CSNK2A2, DDX10, PRKCA, DICER1, CLPX, LARS2, DYNC1LI1, DYNC1LI2, MTHFD1, PRKD3, TYRO3, YME1L1, PRKCQ, IDNK, TARS2, PRKD1, PIF1, ABCB1, ABCB6, PRKDC, ABCB7, ABCB5, PRKCZ, MYO6, PIP5K1A, PIP5K1B, LYN, PLK4, ABCA1, CDK17, CDK19, DNAH17, PLK1, EIF2AK3, CDC7, ABCA7, BRAF, EIF2AK4, INO80, UCK2, PPP5C, EHD4, RAD17, CDK10, CDK12, CDK13, CDK14, CDK15, FARSB, TRMU, ITK, SMG1, PRKAG2, ABCC10, IFIH1, IKBKB, DSTYK, TK2, JAK2, IKBKE, JAK1, HELLS, UPF1, SHPRH, RIPK2, RIPK4, ITPK1, CIT, RNASEL, PKN3, ADCY3, ADCY2, ADCY8, EGFR, PAPSS2, WRN, RECQL5, BTAF1, PMS2, PMS1, ATAD1, HIPK4, ATAD5, TPK1, RIOK3, CSNK1A1, ATAD2, GATB, HIPK1, HIPK3, HIPK2, TAOK1, PKN2, TTLL9, FRK, DGKE, DGKD, PLXND1, ATP8A1, DGKB, UBE2D2, UBE2D3, UBE2D1, MYLK3, THG1L, STK10, NRBP2, SMCHD1, STK11, SLK, PAPOLG, PAPOLA, NEK1, SMARCAD1, MAP3K8, MAP3K9, NEK3, STK32A, ROS1, MAP3K4, PSKH1, MAP3K5, RARS2, TOR3A, CSNK1G3, MAP2K4, CDKL5, UBE2E3, SWAP70, TAP2, DYRK1A, TAP1, ATP11C, ATP11B, PRPF4B, VRK1, VRK2, ATP11A, CHD1L, VRK3, GTF2F2, MSH6, MAPKAPK3, MSH2, MSH3, MSH4, MSH5, ROR1, ROR2, RAD54L2, TNIK, TSSK2, DGKI, CSNK1G1, CDKL1, DGKH, IPPK, PFKFB2, BLK, CSF1R, BLM, PFKFB3, STK39, GMPS, RHOBTB3, AK4, AK5, AK6, AK7, AK8, HSP90B1, TKFC, ORC1, STK36, PMVK, ABL1, PLXNA2, PGK1, ABL2, HACL1, MDN1, MAP2K5, NEK8, NEK9, ATP8B2, NEK4, FANCM, ATP8B1, NEK6, TNK2, NEK7, ASNS, BMX, UCKL1, MAP3K15, CDK6, STK24, CDK3, PLXNB1, MAP3K13, MAP3K14, ALK, CIITA, BMPR2, SRXN1, FLT3, MCM8, MCMDC2, LRRK2, MCM9, LRRK1, NADK2, ATP7B, TLK2, PRKG2, ATP7A, PRKG1, ACTR3, RFC1, APAF1, ATRX, ACTR8, MYO3B, PCCA, BMP2K, PIK3C3, MET, PRKAA2, ROCK1, ROCK2, SRC, ATP10D, ATP10B, TTK, ATP10A, CKMT2, KATNA1, P2RY1, FYN, CSK, ASCC3, ACSS1, CNNM2, ATP9B, ATP9A, NTRK2, MYO10, STYK1, CDC42BPB, MERTK, CDC42BPA, MYO19, MTOR, P2RX7, MYO1D, MYO1E, SNRK, MYO1B, RAD50, PFKL, P2RX4, DNAJA4, MYO1F, EPHB6, SMARCAL1, PANK2, PANK1, RTCA, NADSYN1, UBE2L3, RPS6KA3, RPS6KA5, RPS6KA2, DHX57, CHEK1, CHORDC1, DHX58, KDR, LONP2, KIF21A, SEPHS1, EPHB2, DYNC2H1, ACVR1, EPHA4, EPHA7, MCCC1, RECQL, ENTPD7, CSNK1D, MYO7A, UHMK1, DHX40, KATNAL1, ULK3, SIK3, BTK, SUCLG2, SIK2, DNA2, KIF20B, ALPK1, EPHA3, CFTR, RTEL1, DHX8, TRANK1, MST1R, NLK, TTBK2, PGS1, DHX30, NUAK1, BRIP1, SPAST, NUAK2, DHX34, DHX35, DHX36, STK38L, TRPM7, DPH6, RIMKLB, SRPK2, ZRANB3, TRAP1, XRCC6, YES1, XRCC5, INSR, XRCC2, SMARCA5, MYO5A, DCLK2, SMARCA1, SMARCA2, DCLK1, SRPK1, WNK1, PTK7, ADCK1, WNK2, ATM, DHX29, ALDH18A1, ITM2B, ATR, ACVRL1, TOP2B, OLA1, NAT10, IDE, KIF17, CAMKV, GUK1, KIF13A, DHX15, KIF13B, KIF1B, SYK, STRADA, KIF25, KIF24, KIF23, KIF27, GAK, VWA8, RAD51B, HCK, RAD51D, BBS10, RAD51C, KIF2A, NME7, KIT, SGK2, STK40, DDR2, GUCY2C, IGHMBP2, CAMKK1, RAD54B, PAK1, MTHFD1L, PAK6, RAD54L, PAK3, MAP4K5, PAK2, PAK5, MAP4K3, MAP4K4, MAP3K2, DYNC1H1, MAP3K3, MAP3K1, NUBPL, RYK, MOK, UBE2G1, UBE2G2, MLH1, MYO9A, NEK10, CAMK1, NEK11, NRK, YARS2, IGF1R, PPIP5K1, PPIP5K2, KIF5C, KIF5B, NARS2, FPGS, ENPP1, PDGFRB, NSF, ABCG8, PDGFRA, ABCG5, HFM1, ACOT12, MATK, CASK, LMTK2, LIG3, UBE4B, ACYP1, ERN1, KIF16B, RAF1, ABCG1, DDX6, PXK, DDX4, PKDCC, UBA6, DDX1, NPR2, YTHDC2, ACACA, STK3, GRK2, KIF3B, GRK5, GRK4, GRK7, GRK6, MYH11, RIPK1, KIF3C, MORC2, MYH10, MARK3, MARK1, UBE2F, HSPA8, UBE2H, HELQ, UBE2B, HSPA4, TDRD9, CLK4, CLK3, TBCK, KIF18A, GCLC, FER, PAN3, FES, UBE2T, UBE2N, UBA2, BMPR1B, FGFR4, UBE2K, FGFR2, GALK2, BMPR1A, GSK3B, SCYL3, ABCD3, DDX46, NVL, CHD9, MAST2, CHD7, CHD6, DDX42, ATP2A2, CHD5, CHD4, CHD2, CHD1, GPHN, HK3, EEF2K, MYO18A, SCYL2, PSTK, ABCC3, HGSNAT, DDX59, CAMK1D, PDPK1, DDX55, ABCC5, HUNK, DARS2, DDX51, PEX1, IRAK4, QRSL1, DCK, TGFBR1, TGFBR2, LATS1, SUPV3L1, NAV3, UBE2R2, PEAK1, PEX6, MYH9, CAMK1G, RBKS, CAMK2D, DNAH1, DNAH7, DNAH8, DNAH5, DDX20, DNAH9, ATP2C2, ATP2C1, ACVR1B, ATAD2B, MAPK9, MAPK8, RPS6KC1, TDG, MKNK1, MAPK1, PTK2B, MAPK6, ABCF3, CAMK2G, MAPK4, POLQ, DDX31, ATP2B2, ATP2B1, MAPK14, ACVR2B, ACVR2A, WARS2, MAPK11, PIKFYVE, RPS6KB1, ERCC3, ATP13A5, ERCC6, TEK, ATP13A3* |
| **brown** | **MF** | GO:0004712~protein serine/threonine/tyrosine kinase activity | **124** | 1.97 | 0.00 | *IGF1R, STK10, RPS6KA3, STK11, SLK, RPS6KA5, RPS6KA2, CHEK1, AKT3, KDR, AKT1, MAP3K9, EPHB2, PRKACB, ROS1, MAP3K5, PDGFRB, PDGFRA, EPHA4, CSNK1G3, EPHA7, PRKCI, PRKCH, DAPK1, PRKCB, PRKCE, DYRK1A, PRKCA, PRPF4B, ERN1, MAPKAPK3, PRKD3, BTK, TYRO3, PRKCQ, PRKD1, EPHA3, CSNK1G1, BLK, CSF1R, PRKDC, STK39, MST1R, NLK, PRKCZ, STK3, ABL1, ABL2, TRPM7, MARK3, MARK1, PLK4, LYN, YES1, INSR, PLK1, EIF2AK4, BMX, FER, MAP3K15, FES, WNK1, WNK2, ATM, MAP3K13, FGFR4, MAP3K14, FGFR2, ATR, ALK, ITK, SMG1, FLT3, MAST2, LRRK2, LRRK1, PRKG2, JAK2, PRKG1, JAK1, SYK, PDPK1, IRAK4, CIT, HCK, KIT, MET, STK40, DDR2, CAMK2D, PRKAA2, ROCK1, ROCK2, SRC, TTK, EGFR, MAPK9, PAK1, RPS6KC1, PTK2B, PAK6, FYN, CSK, PAK3, MAP4K5, MAPK6, CAMK2G, PAK2, MAPK4, MAP4K3, PAK5, NTRK2, RIOK3, CSNK1A1, CDC42BPB, MERTK, CDC42BPA, MAPK14, MTOR, MAPK11, RPS6KB1, PKN2, TEK, FRK* |
| **brown** | **MF** | GO:0005096~GTPase activator activity | 82 | 1.30 | 0.00 | *ARHGAP9, NRP1, LRRK2, ARHGAP1, CHM, ARRB1, SIPA1L2, ARHGAP35, ARHGAP42, SIPA1L1, EVI5L, RGS6, GIT1, GARNL3, RALBP1, RALGAPA1, RALGAPA2, ELMOD1, ARAP2, TBC1D8, ELMOD3, TSC2, TBC1D9, TBC1D2B, OCRL, RANGAP1, ARFGAP3, ARFGAP2, ACAP3, ARHGAP10, TBC1D1, ACAP2, TBC1D2, RABEP1, RALGAPB, TBC1D4, RP2, RASA1, RASA2, DEPDC5, PLCB1, RABGAP1, RGS14, RAP1GDS1, ASAP3, ARHGAP18, ASAP1, RASAL1, RASAL2, ASAP2, AGAP3, RASAL3, ARHGAP15, ARHGAP22, ARHGAP21, ARHGAP20, ADAP2, CHN2, CHN1, SRGAP2, SRGAP1, EVI5, TBC1D15, TBC1D8B, RAP1GAP2, RABGAP1L, STARD13, TBCD, ARHGAP29, ARHGAP26, MYO9A, ARHGAP24, ARHGAP31, SGSM3, DLC1, SGSM2, RGS10, NF1, RAB3GAP2, SMAP2, SMAP1, RAB3GAP1* |
| **brown** | **MF** | **GO:0031267~small GTPase binding** | **98** | 1.56 | 0.00 | *CYFIP1, NCKAP1, IPO11, NCF2, CSE1L, EPS8, IPO8, GOLGA5, IPO9, RIMS3, RUSC2, RASSF5, PLCE1, ANKFY1, DENND5A, RALBP1, PRKCH, TSC2, OCRL, SYTL5, SYTL4, UNC13D, SYTL2, ACAP2, DAAM1, DAAM2, RIN3, RAF1, DGKI, MYCBP2, RHOBTB3, IQGAP1, IQGAP2, KIF3B, DVL3, RAB11FIP2, SRGAP2, ECT2, RAB11FIP3, EVI5, RABGAP1L, ANKRD27, HACE1, CAV1, RANBP17, MICAL3, AFDN, DIAPH2, PEX5L, DIAPH3, NOX1, PICALM, CDC42SE2, DENND1B, LRRK2, USP33, DOCK7, ARHGAP1, CHM, BICD1, BICD2, XPO1, AP1G1, XPO6, EVI5L, DNM1L, TNPO1, TNPO3, RANGAP1, GAS8, RABGAP1, ROCK1, ROCK2, PKN3, STRN3, GGA2, GGA1, GGA3, MICALL1, PDE6D, SH3BP4, WHAMM, PAK3, PAK2, RPH3AL, RIC1, XPO7, SORL1, RGP1, FMNL3, FMNL2, MLPH, XPOT, SGSM3, SGSM2, RAB3GAP2, PKN2, RAB3GAP1* |
| **brown** | **MF** | **GO:0046872~metal ion binding** | **458** | 7.27 | 0.00 | *OSGEPL1, ZFYVE9, ZFYVE1, GPATCH8, CAPN15, ZC3H12C, ZFYVE26, RNF114, LIPC, TDO2, LIPI, LIPH, ZC3H12A, CDK5RAP1, ANKFY1, RNF111, MBNL1, GTPBP8, RC3H2, RUFY1, ACE2, RNF123, ZFYVE16, RNF125, RNF128, PRKD3, RUFY4, RUFY3, RUFY2, PRKD1, IDO2, DUS3L, MTMR3, XPA, AGAP3, BAZ2B, MTMR4, PRDM10, ADAMTS10, ADAMTS14, RNF214, RNF216, RNF215, ADAMTS17, RNF217, VPS11, BARD1, MEX3C, MLLT10, PPP5C, MEX3B, CPEB1, NPLOC4, ITPA, RAD18, RNF220, PHF3, ITK, GMEB1, LRSAM1, PHF20, TCF20, PHF6, GLI3, SLC6A4, RNF157, EEA1, TRIM8, ING2, ING3, IMPA2, RNF150, SHPRH, ARG2, BRD1, CNOT6L, MYOF, BAZ1A, BAZ1B, RBSN, PGM2L1, CIT, RNF168, PHF20L1, COL2A1, DPYD, MTF2, TKT, ADCY3, ADCY2, ADCY8, FGD2, FGD3, FGD4, NEURL1B, FGD6, UNK, NSD1, CHN2, MICALL1, RNF130, PCGF6, ANKIB1, CRBN, RIOK3, PCGF5, PCGF2, ELP3, GTF2H3, RNF149, RLIM, MSRB3, RNF141, YPEL2, LIMS1, DGKD, F13A1, SLC8A1, PPP1CB, MAEA, PAPOLG, RNF19A, PAPOLA, PMPCB, KMT5B, NUDT13, MAP3K5, NUDT12, RNF43, RNF44, FOXP4, SAP30, FOXP1, PYGO1, ACAP3, ACAP2, STIM1, KAT6B, STIM2, KAT6A, DGKH, KMT2E, FTO, KMT2C, RNF180, IREB2, GLIS3, GMPR, RASAL1, GLIS1, PHF21A, YBEY, SMYD4, RNF175, FANCL, BRPF3, NRDC, BMX, CNOT4, UBOX5, MAP3K15, CNOT6, ITGA11, RNF182, ACO1, LHX5, MDM4, LHX4, MNAT1, MAP3K13, PLEKHF2, DIDO1, RNF11, RNF13, RNF14, ZC3H3, ITGB2, ECE1, ZC3H6, PPP6C, ADAMTS3, EXO1, RNF17, ME3, ME2, YAF2, UNKL, ADAMTS6, ADAMTS7, DTX3L, ITGA2, ELAC1, ITGA1, ATRX, MSL2, QTRT2, PCCA, ASPA, RNFT2, RNF32, PRKAA2, ROCK1, UHRF2, FRRS1, PLAG1, ROCK2, RPE, RNF38, PPM1K, PPM1E, PPM1F, HDAC7, MLLT6, ABLIM1, ZNRF2, ABLIM2, HECTD1, ABLIM3, RSBN1L, BCO2, RFFL, RNF20, RPH3AL, METAP1D, DDHD2, DDHD1, ASXL1, RAD50, PFKL, POLR3B, DNAJA4, ASXL3, VPS41, ASXL2, SNRNP48, PDCD2, SMAP1, METAP1, SPON1, RFESD, ZMYND8, RTCB, ZDHHC5, ANTXR2, TOPAZ1, DHX57, CHORDC1, DZIP1L, TNFSF10, BSN, TP63, ACVR1, RSAD2, SCAF11, LIMCH1, RSAD1, MCCC1, EBF1, YOD1, OVOL2, BTK, ZNF711, DNA2, ZFPM2, CBLL1, RTEL1, PDE1A, OMA1, RPAP2, NLN, VPS8, TRPM7, RIMKLB, LYAR, ZRANB3, ZRSR2, FER1L5, ZRANB1, ZRANB2, ADHFE1, MICAL3, EXT1, POLA1, LACC1, SPRTN, PDE3A, TRMT13, INTS12, NRP1, TOP2B, RSF1, SH3RF1, PPP3CA, PPP3CC, PCLO, UIMC1, ARIH2, REV3L, IDS, ARIH1, GIT1, RSPRY1, VAV3, AMPD3, CBFA2T2, ZFX, RNF144A, RNF144B, NME7, KIT, G2E3, PRIM2, ATP23, SCEL, LIMA1, NT5E, RELN, DZANK1, LNPK, SUZ12, LMLN, FAH, MYO9A, PDP1, SH3RF2, PDP2, TTC3, ESYT3, ESYT2, DCLRE1A, PRDM5, JADE1, JADE3, JADE2, FPGS, ENPP2, ENPP1, PDE8A, LMO1, NSF, HGD, LMO4, ADAM10, LMO7, CDKAL1, SDHB, RSBN1, LMX1A, HGS, WDFY1, WDFY3, LMX1B, WDFY2, PDE9A, UBA5, PXN, CACNA1D, CACNA1C, DTX4, ACACA, ZFP36L1, STK3, RAI1, MAN2A2, MAN2A1, LPXN, ZC3H18, LONRF3, LONRF1, ZC3H13, ZC3H14, ANKZF1, BPTF, SMAD2, SMAD4, SMAD3, CPSF3, PHF12, SMAD9, HELZ, SMAD5, SMAD7, COL5A2, MKRN2, PHF14, UBA2, TAB3, STT3B, BMPR1B, RERE, RBM27, KDM5A, RBM26, PDE3B, ATP2A2, CHD5, CHD4, GPHN, FYCO1, ZC3H7A, ZC3H7B, PDE4B, POLL, POLK, NOS1, RBM5, PDE4D, PJA2, TGFBR2, RASA2, CAT, ITGB1BP2, ZMYND11, RBKS, FAN1, ASAP3, ETFDH, RNF8, PARN, ASAP1, ASAP2, ATP2C2, ATP2C1, NT5C2, USP19, NT5DC1, PDLIM3, PDE11A, RMND5A, TAF1B, PPP2R3C, NT5DC2, SAP30L, PDE6C, PDLIM5, MUTYH, KDM4A, TAF15, NOS2, B3GAT2, EYA3, EYA4, ZNF804B, CADPS, ATP2B2, ATP2B1, ACVR2B, ACVR2A, PIKFYVE, PDE10A, XPNPEP1, LNX1, ATP13A5, ATP13A3, PDE7B, PDE7A, TAF3, LNX2* |
| **brown** | **MF** | **GO:0003682~chromatin binding** | **113** | 1.79 | 0.00 | *CCNT2, KDM1A, CCNT1, GMNN, NUCKS1, SMC3, TTF1, RBPJ, SMC4, SMC2, ELK4, ZC3H12A, TRIM24, KMT5B, PKNOX1, TP63, SMARCC1, SLC30A9, MEF2C, PRKCB, MITF, HNF1A, ACYP1, CDYL, SOX10, MSH6, MSH2, ANKRD17, L3MBTL3, URI1, DLX3, KMT2A, DHX30, ORC1, ATXN7, TTC21B, TPR, HESX1, MORC2, SKIL, TFAP2A, SMAD4, FANCM, ESR1, NFKB1, FLI1, TDRD3, FOSL2, STAG1, UBE2T, SP3, RERE, TOP2B, MCM9, KDM8, HP1BP3, PSIP1, HOXC13, GLI3, ING2, SIN3B, EXO1, JARID2, GABPA, POLG, HELLS, NCOA2, UPF1, BCAS3, PBRM1, NCOA5, EED, ATRX, ACTL6A, NR5A1, RNF168, KAT2B, TOX3, NR5A2, ZEB1, POLR1A, WAC, LCOR, JDP2, YAP1, KDM3A, PRKAA2, SATB1, SATB2, LEF1, RNF8, MLLT3, HOXD10, RNF2, EGFR, HDAC7, PRIMPOL, IFT74, MNT, MTA3, BRD4, RNF20, PCGF2, MBD2, LRWD1, MLH1, MEIS1, POLR3A, NFIA, CTNNB1, NCAPD3, ERCC6, MPHOSPH8* |
| **brown** | **MF** | GO:0035091~phosphatidylinositol binding | 48 | 0.76 | 0.00 | *GRB7, PXK, NCF4, ITPR1, TULP3, ITPR2, PIK3C2G, SNX10, SNX33, SNX30, PIK3C2A, PLD1, SNX31, ZCCHC14, PIK3C2B, MTM1, GGA2, GGA1, SNX4, ING2, SNX1, GGA3, SNX2, SH3PXD2A, RPS6KC1, SNX25, SH3PXD2B, TOM1, HS1BP3, SBF2, APPL2, SNX5, APPL1, SNX6, BCAS3, PHF12, STAM, NISCH, MYO1E, SH3YL1, CCDC88A, SNX19, SNX16, HGS, FES, KIF16B, MITD1, STAM2* |
| **brown** | **MF** | **GO:0004674~protein serine/threonine kinase activity** | **102** | 1.62 | 0.00 | *STK10, RPS6KA3, STK11, SLK, TBK1, RPS6KA5, RPS6KA2, AKT3, AKT1, CPNE3, NEK3, STK32A, PSKH1, MAP2K4, CSNK1G3, CSNK2A1, CSNK2A2, DYRK1A, LMTK2, VRK1, CSNK1D, PRPF4B, VRK2, UHMK1, ERN1, MAPKAPK3, ULK3, SIK2, TNIK, ALPK1, RAF1, TSSK2, CSNK1G1, CDKL1, STK39, PRKCZ, STK3, NUAK1, NUAK2, STK38L, RIPK1, TRPM7, MARK3, MAP2K5, MARK1, SRPK2, PLK4, CDK19, NEK6, PLK1, NEK7, BRAF, CLK4, DCLK1, CLK3, SRPK1, STK24, WNK1, WNK2, CDK3, CDK14, ATR, GSK3B, SMG1, MAST2, LRRK1, IKBKB, DSTYK, TLK2, HTATIP2, CAMK1D, SYK, RIPK2, HUNK, IRAK4, CIT, LATS1, SGK2, STK40, ROCK1, PAK1, RPS6KC1, MKNK1, PAK6, PAK3, PAK2, PAK5, RIOK3, CSNK1A1, MOK, CDC42BPB, CDC42BPA, HIPK3, MTOR, HIPK2, SNRK, PIKFYVE, RPS6KB1, NEK10, CAMK1, NEK11, TAF1* |
| **brown** | **MF** | GO:0019903~protein phosphatase binding | 37 | 0.59 | 0.00 | *CSF1R, MTMR3, STX17, MTMR9, AMBRA1, IQGAP1, PIK3R1, MTMR4, EGFR, HSP90B1, SNX3, MAPK8, TBK1, CDH2, PPP6R2, PPP6R3, CDC27, STAT6, KIFAP3, ROS1, JAK1, MAP3K5, ANAPC7, ITGA1, EIF2AK3, AP3B1, VRK3, MAPK14, ANK1, PPP1R3C, TRAF3, CTNNB1, ANAPC4, GRB2, ANAPC5, FBXL2, MET* |
| **brown** | **MF** | **GO:0042802~identical protein binding** | **309** | 4.90 | 0.00 | *RB1, AHCYL1, GCC2, PKD2L1, AQP5, IKZF2, ACCS, CLDN1, GLE1, TBK1, ALCAM, TFG, RASSF3, UBASH3B, TDO2, FGFR1OP2, KDR, TNFSF10, LUC7L, EPHB2, TP63, EPHA4, IAH1, DAPK1, FNBP1, SOX13, HNF1A, SOX10, NISCH, ACE2, NIF3L1, PLPP4, DAAM1, BIN1, SUB1, XRN2, BTK, ORAI1, KCTD17, CBLL1, ATF6, KCTD15, GRB7, SPTBN5, MTMR2, L3MBTL3, DHX8, MYCBP2, MGST2, BCL10, APCDD1, HMGXB4, ZNHIT6, CEP70, TP53BP2, TRPM8, LYAR, DECR1, PLK4, ZRSR2, PHC2, XRCC4, PLK1, EIF2AK3, FN1, NMI, GDF5, UCK2, PPP5C, PPFIBP2, GKAP1, CCDC88C, NAPEPLD, TMCC3, GRB2, ATM, TMCC1, ALDH18A1, DCP1A, MRAP2, RAD18, ITPA, CEP57, CCDC125, PTPRM, CD3E, BRCA2, IFIH1, NNT, C1QTNF6, HEY2, GNPNAT1, JAK2, IKBKE, NDEL1, APPL1, APOBEC2, PARP1, KIF24, ZDHHC17, SIRT1, LRRC41, MTSS1, SμmF1, ALDH5A1, COL2A1, PICK1, MAPRE3, VWA2, MAPT, MAPRE2, TLR3, TLR2, SERPINC1, ATL2, IGHMBP2, HTT, SNX33, EGFR, PSMA7, ALS2CL, HSF2, EXOSC8, MICALL1, MFN1, HSF4, STAT6, FLNB, DCPS, EPS15, PAK2, LNPK, CAP2, NQO1, STAT1, TPK1, STAC3, KLHL2, PLEKHA3, UBE2G2, PEX14, MKLN1, CENPJ, CCDC6, MITD1, SH3GL3, SLC27A1, OLFML2B, PRDM6, ITGA2B, ZBTB26, FBXO28, DEUP1, OLFML2A, ETS1, FGF2, CDH8, IGF1R, EDC3, THG1L, CDH2, TNN, KIF5B, TMEM38A, TMEM38B, SBF2, HGD, FBXW7, USP4, HGF, DYRK1A, FAM118A, ACOT12, EMSY, LZTFL1, DCTD, PRPF6, STIM1, POLG2, FAM161A, RAF1, IL6ST, CUL3, NPR2, SLC1A1, ILDR1, KLHL12, UAP1, RASAL3, ACACA, STK3, HMBOX1, SCUBE1, ATXN1, BTBD3, DVL1, PLXNA2, TASP1, HACL1, RPRD1B, PDZD7, PACSIN1, TFAP2A, AKIRIN2, STIL, SMURF2, TFAP2E, CAV1, INHBA, HOOK3, ESR1, NFKB1, IGF2R, NMRAL1, CLK3, AHI1, BMP1, BCL6, P4HA1, SP4, BCL2, AMOTL2, AMOTL1, MYD88, FBN1, ALK, SCYL3, LRRK1, ADRA1D, HTR2A, ALAD, PSTPIP1, TLK2, RNF17, UPP2, TOPBP1, SULT4A1, TNPO3, GGPS1, VWF, APAF1, AXIN1, SORD, TRAF1, DNM1, PEAK1, TRAF3, TRAF6, TRAF5, IRF5, NAF1, MNS1, SLC25A12, MET, ASPA, TRIM55, BIRC2, RBKS, SLC25A13, USP15, NUFIP1, HPGD, ZBTB48, PPM1H, TTK, RNF4, MALT1, MRM3, PRTG, TAF1D, GMDS, FCHO2, UBQLN1, MAPK1, SH3BP4, STOM, CSK, NCAM2, PCBD2, UBQLN4, RNF20, SPAG9, ANGPT1, TRPA1, SYT16, FOXJ2, CDC5L, CDC42BPA, MCOLN2, MTOR, P2RX7, RAD52, PFKL, P2RX4, TNIP1, CD6, ZNF618, COL7A1, VPS41, EIF3J, LNX1, NUP35, FAM98B, CD247, TEK, CDR2L, KCNK1, PIK3AP1, LNX2* |
| **brown** | **MF** | **GO:0005085~guanyl-nucleotide exchange factor activity** | **88** | 1.40 | 0.00 | *ITSN2, BCAR3, DENND1B, DOCK4, DOCK3, DOCK9, DENND5B, RASGRF2, DOCK8, DOCK7, ITSN1, WDR41, THG1L, DOCK10, RABGEF1, FLCN, PREX1, PSD2, PSD3, PLCE1, DENND5A, FBXO8, MCF2L2, SBF2, RALGPS1, ARFGEF3, RALGPS2, VAV3, DENND2D, DENND2C, PLEKHG1, ARHGEF12, PLEKHG7, DIS3, ARHGEF17, PLEKHG5, SH2D3C, GAPVD1, TIAM1, ARHGEF9, ALS2, RAPGEF1, ARHGEF3, RAPGEF2, DENND6B, RIN3, DENND6A, DOCK2, SOS1, RAPGEF5, SOS2, DOCK1, RAPGEF6, ARHGEF5, ARHGEF6, ARHGEF26, ARHGEF28, MYCBP2, RASGRP1, RCC1L, FGD2, FGD3, ABR, FGD4, FGD6, ECT2, FNIP1, RALGDS, ARHGEF33, ARFGEF1, FARP2, ARFGEF2, FARP1, EIF2B3, RIC1, ANKRD27, ARHGEF37, DENND4A, ARHGEF39, DENND4C, GBF1, ARHGEF38, NET1, RGP1, CCDC88A, CCDC88C, RAB3GAP2, RAB3GAP1* |
| **brown** | **MF** | **GO:0019901~protein kinase binding** | **110** | 1.75 | 0.00 | *ATF2, CCNK, CCNT1, NR3C1, PPP1CB, CCND3, CDH2, CEP250, FBXO7, CDK5RAP2, MAP3K5, NSF, PDGFRB, IFNAR2, ADAM10, UNC5C, VRK2, ACSL3, ATP1B1, ATG13, FRMD5, CCNY, MSH2, CD226, UTRN, TELO2, PFKFB2, GRB7, IQGAP1, PDCD10, DVL1, RAB11FIP2, GCN1, FAM83B, CNTLN, TRAP1, SMAD3, CAV1, NEK6, PLK1, TRIP4, ESR1, ZC3HC1, IBTK, PTPRC, TBL2, RHEB, CD28, SPDYA, GRB2, CSPG4, CDK12, CDK13, FERMT2, NRP1, USP37, FAF1, ITGB2, CLTC, PRKAG2, ELAVL1, BICD1, PRKAG3, IKBKB, UFL1, SPRED2, SPRED1, SUFU, KIF13B, JAK2, CEP68, PPP1R12A, SYK, PARP1, EMP2, IRAK4, DNM1, CIT, LATS1, DNAJC3, KAT2B, TRAF3, CRY2, IRF5, MAPRE3, MAPT, CACUL1, MAPRE2, KIZ, PRAM1, SV2A, RNF138, RBBP6, RICTOR, PAK2, MAPK4, SLC12A2, MAP3K2, TCF7L2, SLC12A4, MAP3K1, CEP152, CADPS, STAT3, ELP2, APC, PRC1, CENPJ, CTNNB1, CCNYL1* |
| **brown** | **MF** | **GO:0016887~ATPase activity** | **101** | 1.60 | 0.00 | *CLPB, SMC5, SMC3, SMC4, SMC2, SMCHD1, DHX58, LONP2, NSF, DDX17, DDX18, ABCG8, TOR3A, ABCG5, RECQL, DDX10, CHD1L, ACYP1, GTF2F2, CLPX, DHX40, KATNAL1, MSH2, YME1L1, RAD54L2, DNA2, KIF20B, CFTR, DDX6, PIF1, BLM, RTEL1, DDX4, DHX8, ABCB6, YTHDC2, RHOBTB3, AK6, HSP90B1, SPAST, ORC1, DHX34, DHX35, DHX36, MORC2, MDN1, TRAP1, HSPA8, HSPA4, TDRD9, SMARCA5, INO80, SMARCA2, MOV10, DHX29, ABCD3, DDX46, CHD9, MCM8, NVL, MCM9, CHD7, OLA1, CHD6, DDX42, CHD5, CHD4, CHD2, CHD1, IFIH1, DHX15, RFC1, DDX55, ATRX, PEX1, VWA8, SUPV3L1, BBS10, NAV3, PEX6, DDX20, ATAD2B, WRN, BTAF1, RECQL5, KATNA1, PMS2, PMS1, ATAD1, ATAD5, NUBPL, ATAD2, ATP2B1, MLH1, MYO19, MYO1E, PIKFYVE, RAD50, ERCC3, ATP13A5, ATP13A3* |
| **brown** | **MF** | **GO:0008017~microtubule binding** | **73** | 1.16 | 0.00 | *CEP57, TRAF3IP1, MTCL1, DYSF, FMN1, KIF17, FNTA, KIF5C, DPYSL5, KIF5B, KIF13A, ZNF207, KIF13B, KIF1B, KIF21A, LZTS1, CDK5RAP2, NDEL1, APC2, SGIP1, MAP1S, CEP295, STARD9, VASH2, KIF25, KIF24, KIF23, EML1, MID2, KIF27, KATNAL1, NAV3, FAM161A, KIF2A, KIF16B, NME8, KIF20B, MAPRE2, GAS8, RGS14, JAKMIP3, SKA1, SKA2, SPAST, KIF3B, MAP2, KATNA1, KIF3C, WHAMM, MAP4, GAS2L1, MAP9, CEP350, PLK1, RMDN3, HOOK3, RMDN1, PEX14, CCDC88A, VAPA, VAPB, PRC1, KATNBL1, FES, VPS41, TUBGCP5, CCSER2, TUBGCP6, MAP6D1, CCDC66, FGF13, CEP44, MDM1* |
| **brown** | **MF** | **GO:0042803~protein homodimerization activity** | **171** | 2.71 | 0.00 | *DCLRE1B, CDA, ATF2, DGKD, CSF1, PANK1, FMR1, UXS1, SPPL2A, IKZF3, ZDHHC3, TMEM266, STK10, SMCHD1, HPGDS, GOLGA5, SLK, FBXO4, PIP4K2A, ENPP1, PIP4K2B, SOX6, IL6R, MAP3K5, ACVR1, PDGFRA, CUBN, STARD3, ST6GAL1, MIGA2, ACOT7, TYW5, ACTN1, GLCE, ADAM10, TSC2, MID2, ERN1, TPST1, JAML, MSH2, ZNF318, RABL3, TARS2, MASP1, KIF20B, EXD2, CSF1R, BLM, ABCB7, UBA5, ZBTB1, XPA, PDGFB, NRF1, FXR1, DPP4, SNX1, SNX2, INPP5F, PDCD10, TPR, HESX1, SNX9, RIPK1, PSMF1, RAB11FIP2, SRGAP2, ECT2, RAB11FIP3, MORC2, ATG7, ST3GAL2, BARD1, TFAP2B, WWTR1, SMAD4, TGFB2, SMAD3, EPHX2, SNF8, EXT1, EXT2, CCDC88A, PRPH2, BHLHE40, KYAT3, MAP3K13, CNOT9, FGFR2, BMPR1A, CEP57, TENM1, MYOM1, ABCD3, TENM4, LRRK2, HSPB8, PLEK, IDE, PTH1R, PKD2, ELAVL1, TRIM8, EEA1, IKBKB, NADK2, IMPA2, KYNU, MFSD1, LRRFIP1, HIF1AN, DNM1L, APPL2, APPL1, ZHX2, TRPC6, CDADC1, RIPK2, DARS2, ARNT, ANO6, COMMD1, TERF1, TERF2, SUPV3L1, TOX3, QTRT2, PEX7, RABEP1, DPYD, CAT, KIT, HAND1, CDH13, TKT, JDP2, CARNMT1, HM13, RNF8, LRP4, NOD1, RCHY1, MFF, ADD1, LRP6, WRN, TDG, CAMK2G, MEF2D, MAPK4, GCH1, NOS2, STAT3, HIP1R, GRHL1, SLC8B1, PML, TPCN1, TBX15, CD4, NR6A1, XPNPEP1, VAPB, XPNPEP3, KLHL7, GID8, CD247, CCDC66, NECTIN3, MMACHC* |
| **brown** | **MF** | GO:0003713~transcription coactivator activity | 59 | 0.94 | 0.00 | *KDM5A, PHF2, WWC1, JADE1, PSIP1, ARRB1, NUCKS1, BRCA1, MED16, TRIM8, MED14, MED13, ZMIZ1, ZXDC, TRIM24, EP300, YAF2, SS18, MYOCD, ARID5B, SIRT1, MID2, TOX3, KAT2B, TOX2, PRPF6, THRAP3, IRF4, KAT6A, SUB1, TRIM13, NUP98, ZFPM2, BIRC2, YAP1, USP16, CBFB, XPC, BCL10, MTDH, NRIP1, BRD7, BCL9L, BRD4, RNF20, WWTR1, SS18L1, TRIP4, RRP1B, SMARCA2, ZZEF1, TDRD3, HIPK2, ASXL1, BCL9, TADA2A, CENPJ, CTNNB1, TADA1* |
| **brown** | **MF** | GO:0016922~ligand-dependent nuclear receptor binding | 18 | 0.29 | 0.00 | *NCOA1, TCF7L2, BCAS3, NCOA2, SLC30A9, SMARCD3, NCOA3, PROX1, ARID1A, SIRT1, NCOR1, C1D, CRY2, TRIM24, CTNNB1, NCOA7, TACC2, PPARGC1A* |
| **brown** | **MF** | GO:0017124~SH3 domain binding | 30 | 0.48 | 0.00 | *KHDRBS2, WIPF1, FMN1, PTPN22, CBL, CD3E, FUT8, ABL1, OSTF1, WASF2, REPS1, LYN, AFAP1L2, SGIP1, LANCL1, HIP1R, ADAM10, PTPN12, ENAH, ADAM17, ARHGAP31, ABI1, ADAM12, RUFY2, ELMO1, ELMO2, ADAM9, GRB2, EVL, MAPT* |
| **brown** | **MF** | GO:0004842~ubiquitin-protein transferase activity | 46 | 0.73 | 0.00 | *RNFT2, WDSUB1, RNF8, NEDD4L, CBLB, BRCA1, CBL, MALT1, HECTD2, HERC1, RNF19A, RNF217, ARIH2, ARIH1, RSPRY1, BARD1, RNF20, PPIL2, HACE1, ANKIB1, NSMCE2, RC3H1, FBXO11, RC3H2, PJA2, KLHL20, DDB2, RNF168, RNF144A, CNOT4, RNF123, TRAF7, RNF144B, HECW2, TRAF6, TTC3, TRIM13, G2E3, LNX1, ERCC8, TRIM36, TRIP12, FBXL3, DTL, BIRC2, RNF220* |
| **brown** | **MF** | GO:0035064~methylated histone binding | 29 | 0.46 | 0.00 | *KDM5A, KMT2E, PHF2, FMR1, KDM8, CHD1, RRP8, MBTD1, ING2, ING3, RBBP5, SPIN1, TP53BP1, SUZ12, MORC3, MORC4, ATRX, CDYL, LRWD1, CDYL2, ZZEF1, TDRD3, MSH6, PYGO1, ZZZ3, MTF2, NCAPD3, MPHOSPH8, KDM7A* |
| **brown** | **MF** | **GO:0061630~ubiquitin protein ligase activity** | **66** | 1.05 | 0.00 | *RNF11, TRAF3IP2, UBE3C, RNF14, UBE3D, UBE2D3, LTN1, UBE2D1, UBE3A, JADE2, RABGEF1, MAEA, TRIM24, FBXO4, BTRC, RNF111, KCMF1, AREL1, DTX3L, FBXW11, MSL2, RC3H2, RNF125, RNF128, NFX1, TRAF6, PELI1, CBLL1, PDZRN3, RNFT2, UHRF2, MGRN1, RNF180, CUL3, MYCBP2, RNF38, UBR4, DTX1, NEDD4L, UBR3, UBR2, DTX2, UBR1, RCHY1, DTX4, RNF2, ZNRF1, RMND5A, HECTD1, RNF138, RBBP6, SMURF2, FANCL, SMURF1, MIB2, WWP1, WWP2, MEX3C, RNF146, SH3RF2, ITCH, NEDD4, MKRN2, UBE2O, ASB2, RAD18* |
| **brown** | **MF** | GO:0048487~beta-tubulin binding | 17 | 0.27 | 0.00 | *BCAS3, MAP1S, TBCD, HTT, DLEC1, TBCA, SMC3, PEX14, IFT74, SPAST, VAPB, TAOK1, FGF13, BBS4, NDEL1, APPL1, ARL8B* |
| **brown** | **MF** | GO:0045296~cadherin binding | 16 | 0.25 | 0.00 | *ACVR1, BMPR2, TRPC4, CTNND1, PTPRM, PTPRB, TBC1D2, P2RX4, MMP24, KDR, NμmB, CTNNA1, CDH13, CTNNB1, CTNNA3, PROM1* |
| **brown** | **MF** | GO:0004843~thiol-dependent ubiquitin-specific protease activity | 42 | 0.67 | 0.00 | *USP13, USP14, USP37, USP15, USP16, USP31, USP53, USP54, USP32, USP10, USP33, USP34, OTUD7A, OTUD7B, TANK, MYSM1, USP19, VCPIP1, ZC3H12A, USP1, USP24, USP46, USP47, USP25, USP8, USP48, USP7, ZRANB1, USP49, USP42, DESI2, USP4, USP9X, USP3, USP45, YOD1, USP28, JOSD1, UCHL3, UCHL5, OTULIN, USP40* |
| **brown** | **MF** | GO:0004715~non-membrane spanning protein tyrosine kinase activity | 22 | 0.35 | 0.00 | *LYN, BLK, ITK, YES1, PKDCC, SYK, SRC, TNK2, DYRK1A, BMX, HCK, FER, FES, PEAK1, ABL1, BTK, ABL2, PTK2B, FYN, CSK, FRK, JAK1* |
| **brown** | **MF** | **GO:0008270~zinc ion binding** | **239** | 3.79 | 0.00 | *CDA, IKZF4, ALKBH8, RABGEF1, ZMIZ1, CHORDC1, DHX58, TRIM24, TNFSF10, DPF3, KAT7, SCAPER, EARS2, ESCO1, PRKCB, NSMCE2, RC3H1, PRKCA, CLPX, NEIL3, RNF125, LACTB2, TRIM13, L3MBTL3, MYCBP2, GATA6, GATA4, GATA3, MBTD1, ERI2, LTA4H, PHC2, PHC1, DTNB, SETDB1, SIAH2, SIAH1, TRIP4, NR1D2, PHC3, ZZZ3, ACER3, NAPEPLD, PAM, PHF2, OTUD7A, HNF4G, OTUD7B, IDE, BRCA1, LITAF, ZCCHC14, GLI2, IFIH1, MMP24, ZMYM2, ZMYM3, TRIM2, ARIH1, LMCD1, ZCCHC24, UPF1, PARP1, ZFR, TET2, SIRT4, TET1, NR5A1, NR5A2, MMP16, NFX1, RARA, RGN, RARB, PPARA, PPARD, ZDHHC20, PRICKLE2, PRICKLE1, GLRA1, ZSWIM8, ZSWIM6, DMD, ZNF346, RXRG, MAP3K1, GCH1, MIB2, RNF146, AGBL5, RNF145, ZNFX1, NR6A1, AGBL1, QPCT, AGBL2, NBR1, TRIP12, RTN4IP1, ZNF330, ZFAND4, ZFAND3, KDM1B, ZFAND5, LTN1, RORA, RORB, NR3C1, ENPP2, EP300, KCMF1, PIAS4, MORC3, AGTPBP1, MORC4, KDM2B, ZGRF1, USP3, LIG3, FBXO11, MID2, PIAS2, PIAS1, DCTD, CLIP1, ZNF318, PGR, MATR3, UTRN, ZCCHC8, ZCCHC7, BLM, KMT2A, TNKS, UBA5, ZCCHC9, UBR4, UBR3, DTX1, DTX2, DNAJC24, UBR2, UBR1, PITRM1, ZMAT3, DNAJC21, UBR5, MORC2, BPTF, ZFHX3, SMAD3, NFXL1, SREK1IP1, ESR1, ZFHX4, ZZEF1, ESR2, ZC3HC1, LIN28B, BMP1, TAB3, TAB2, XAF1, ZCCHC4, KDM5A, TRIM71, KDM5B, THRB, CPXM2, USP33, MTR, NR2E1, ALAD, DUSP12, ADAMTS5, CALB1, PTER, ADAMTS1, TRPS1, ZNF407, GATAD1, CA7, ZNF385B, HIF1AN, ADAMTS9, TRIM67, CDADC1, USP49, USP45, LANCL1, SORD, TRAF1, ADAMTS20, DBF4, POLR1A, TRAF3, TRAF6, ZNF638, TRAF5, RBM20, ITGB1BP2, KDM7A, BLVRA, TRIM55, BIRC2, PAPPA2, USP13, SEC23A, USP16, GALT, WBP4, RNF8, RNF4, RCHY1, NR2C2, CXXC4, NR2C1, RNF2, PRIMPOL, RBBP6, MTA3, VAT1L, TRIM45, PTPN1, CIZ1, KDM4C, SEC24A, RNF24, ERAP1, PML, CD4, PIKFYVE, TRIM35, TLL2, TRIM36, TRIM37, SEC24D, SEC24C, TRIM33* |
| **brown** | **MF** | GO:0030983~mismatched DNA binding | 9 | 0.14 | 0.00 | *MSH6, MSH3, TDG, MSH4, MSH5, PMS2, ACYP1, MLH1, PMS1* |
| **brown** | **MF** | GO:0017056~structural constituent of nuclear pore | 14 | 0.22 | 0.00 | *NUP214, NDC1, NUP205, NUP107, NUP133, NUP155, NUP160, NUP93, NUP85, TPR, NUP35, NUP88, NUP98, NUP58* |
| **brown** | **MF** | GO:0003678~DNA helicase activity | 22 | 0.35 | 0.00 | *RTEL1, XRCC6, DDX4, XRCC5, MCM8, CHD9, MCM9, CHD7, ATRX, CHD6, RECQL, CHD5, CHD4, CHD1L, CHD2, GTF2F2, CHD1, SUPV3L1, RECQL5, ERCC3, SUB1, DHX36* |
| **brown** | **MF** | GO:0000976~transcription regulatory region sequence-specific DNA binding | 31 | 0.49 | 0.00 | *KDM5A, CCNT1, ZBTB48, PRDM5, ASH2L, GATA4, HOXC13, BRCA1, GABPB1, RBBP5, HSF2, HSF1, HSF5, SOX6, BRD7, PITX2, TP63, CDK5RAP2, SOX5, TFAP2A, XRCC5, ARID5B, RFX3, SMARCA2, PAX2, IRF4, TBL1XR1, CRY2, IRF5, IRF6, TAF2* |
| **brown** | **MF** | GO:0043130~ubiquitin binding | 31 | 0.49 | 0.00 | *USP13, USP16, FAF1, FAF2, RNF8, CXCR4, GGA2, GGA1, GGA3, UBR5, TOM1, UBXN7, SMARCAD1, ASCC2, JARID2, FBXO7, N4BP1, SMAD3, STAM, RAD23B, RNF168, HGS, NEDD4, NBR1, SPRTN, UBAP1, TAB3, TAB2, STAM2, CUEDC1, BIRC2* |
| **brown** | **MF** | GO:0042162~telomeric DNA binding | 12 | 0.19 | 0.00 | *UPF2, UPF1, PIF1, XRCC6, SMG1, ZBTB10, UPF3A, TP53BP1, TERF1, SMG7, SMG5, SMG6* |
| **brown** | **MF** | **GO:0031625~ubiquitin protein ligase binding** | **77** | 1.22 | 0.00 | *RB1, GSK3B, FAF1, FAF2, WBP1L, ARRB1, SMC6, BRCA1, SMG5, MAP1LC3C, SLC22A18, UBXN7, ARIH1, DNM1L, PRKACB, FBXO7, IKBKE, CCDC50, JAK1, PIAS4, FBXW7, STX8, AXIN1, YOD1, TRAF1, AXIN2, PIAS2, PIAS1, TRAF3, TRAF6, TRAF5, CACUL1, VCL, USP13, RALB, CUL5, HM13, CUL3, CUL2, CUL1, RNF8, CXCR4, PRR5L, AMBRA1, BCL10, UBE2J2, NLK, TANK, USP19, EGFR, UBE2J1, BAG4, RIPK1, SNX9, PPARGC1A, RNF20, HSPA8, USP25, SLF1, SLF2, SMAD3, UBE2B, FANCL, XRCC5, NEK6, FZD6, UBE2G1, SMAD5, PML, MFHAS1, UBOX5, PER3, APC, UBE2T, BCL2, RAD18, CUL4B* |
| **brown** | **MF** | GO:0046332~SMAD binding | 20 | 0.32 | 0.00 | *ACVR1, SKOR1, USP15, SMURF2, TGFBRAP1, TCF12, ACVR1B, PML, TGFBR2, HIPK2, TGFBR3, SKI, PURA, ZMIZ1, CREB3L1, COL5A2, BMPR1B, SKIL, FERMT2, BMPR1A* |
| **brown** | **MF** | GO:0004675~transmembrane receptor protein serine/threonine kinase activity | 10 | 0.16 | 0.00 | *ACVRL1, ACVR1, BMPR2, BMPR1B, ACVR1B, ACVR2B, ACVR2A, TGFBR1, TGFBR2, BMPR1A* |
| **brown** | **MF** | GO:0044877~macromolecular complex binding | 50 | 0.79 | 0.00 | *DCLRE1B, PTPRF, FLCN, ING2, HELB, EPHB2, APPL2, GIT1, APPL1, NSF, UPF1, PDGFRA, IST1, PEX1, TERF2, LZTFL1, DDB2, PEX6, TELO2, TLR2, SF3B3, LRP1, NOD1, XPC, STRN4, STRN3, HMBOX1, GGA3, DHX34, ANKRD54, RIPK1, LYN, CARMIL1, SLF1, CARMIL2, SLF2, XRCC5, PTCH1, CAV1, INSR, MTHFR, CDC42BPB, TSHR, MTOR, GNB1, GNB4, ERCC8, ATM, ERCC6, RAD18* |
| **brown** | **MF** | **GO:0004672~protein kinase activity** | **61** | 0.97 | 0.00 | *SCYL3, NRK, TESK2, MYLK3, NRBP2, CAMKV, TRIM24, NEK1, MAP3K8, SCYL2, HGSNAT, CDKL5, CAMK1D, STRADA, DAPK2, RIPK4, CASK, CSNK1D, VRK3, GAK, MYO3B, KIT, SIK3, BMP2K, ROR1, ROR2, TNIK, GUCY2C, RNASEL, PXK, NPR2, CAMKK1, STK3, TTBK2, NUAK2, STK36, RIPK1, MAP4K4, NEK8, SRPK2, MAP3K2, CDK17, NEK9, HIPK4, MAP3K3, MAP3K1, NEK4, RYK, CSNK1A1, STYK1, DCLK2, CDC7, HIPK1, TBCK, PAN3, STK24, ADCK1, PTK7, TAOK1, CDK12, NEK11* |
| **brown** | **MF** | GO:0043015~gamma-tubulin binding | 14 | 0.22 | 0.00 | *CEP57, TUBGCP2, DIXDC1, BRCA2, RAD51D, BLOC1S2, CENPJ, CEP70, PDE4B, TUBGCP5, TUBGCP6, TUBGCP4, GIT1, CDK5RAP2* |
| **brown** | **MF** | GO:0019899~enzyme binding | 58 | 0.92 | 0.00 | *THRB, BTG1, KDM1A, MCM9, ATP2A2, LDB2, SYNE1, SPDL1, IL6R, ATG3, PARP4, NCOA6, PRKCE, UBE4B, PRKCA, PUS7, TERF2, WDR76, MID2, ERN1, NR5A1, MSH6, PEX7, PCCA, MSH3, CAT, HAND1, PICK1, PLCB1, CFTR, NOTCH2, KMT2E, DDC, TULP3, MST1R, HACD2, HACD4, TNKS2, WDR70, METTL6, RPS2, SUDS3, FNIP1, ZNF346, PMS1, AKIRIN2, ZFHX3, KDM4C, SORT1, RANBP9, MLH1, ESR2, TSPAN15, GOLPH3, NDUFAF7, VAPB, PDCD2, MAP3K13* |
| **brown** | **MF** | GO:0047485~protein N-terminus binding | 29 | 0.46 | 0.00 | *EPB41, DCTN4, SLA2, RBPJ, NIPBL, PDCD10, HESX1, NBN, SMARCC1, VWF, PARP1, KCNIP2, NCOA3, CSNK2A2, ALG2, TSC1, SNF8, GTF2H3, PEX14, NCOR2, MAU2, TBL1XR1, TRAF6, CFAP36, ATM, EXOC5, ERCC6, MNAT1, BIRC2* |
| **brown** | **MF** | GO:0008013~beta-catenin binding | 30 | 0.48 | 0.00 | *AMER1, KANK1, PTPRU, GSK3B, CLSTN1, LEF1, ASH2L, GLI3, CDH2, SUFU, DVL1, CTNNA1, DVL3, EP300, CTNNA3, BTRC, BCL9L, APC2, TCF7L2, TCF7L1, CXADR, CSNK2A1, TRPC4, AXIN1, ESR1, BCL9, APC, TBL1XR1, DLG5, NμmB* |
| **brown** | **MF** | GO:0004386~helicase activity | 15 | 0.24 | 0.00 | *HELLS, HFM1, ZGRF1, TDRD9, DDX51, HELZ, SMARCA2, SETX, DHX32, ZNFX1, DHX34, DHX57, DHX58, ASCC3, RAD54L2* |
| **brown** | **MF** | GO:0042393~histone binding | 31 | 0.49 | 0.00 | *KDM5B, USP16, UHRF2, TNKS, LEF1, RNF8, RSF1, CHD2, MLLT6, VPS72, JAK2, WDTC1, RNF20, BCAS3, BRD1, USP49, DTX3L, PRKCB, SFMBT1, USP3, SFMBT2, SMARCA5, PARP9, BAZ1B, SMARCA2, SIRT1, RNF168, MLLT10, YEATS2, TBL1XR1, NCAPD2* |
| **brown** | **MF** | GO:0001784~phosphotyrosine binding | 19 | 0.30 | 0.00 | *BCAR3, SHC3, SYK, IRS1, SH2D3C, CBLB, PIK3R1, CBL, RASA1, PLCG2, NCK2, ABL1, ABL2, MAPK1, STAP1, GRB2, LDLRAP1, PTPN5, PTPN3* |
| **brown** | **MF** | GO:0004698~calciμm-dependent protein kinase C activity | 11 | 0.17 | 0.00 | *PRKCI, PRKCH, PKN3, PRKCB, PRKD3, PRKCE, PRKCQ, PRKCA, PKN2, PRKD1, PRKCZ* |
| **brown** | **MF** | GO:0004725~protein tyrosine phosphatase activity | 43 | 0.68 | 0.00 | *PTPRT, PTPRU, MTMR2, PTPRR, MTMR3, MTMR14, PTPRO, PTEN, PTPRM, PTPRJ, PTPN22, PTPRK, PTPN23, MTMR4, DUSP16, CDC14A, PTPRF, PTPDC1, MTM1, MTMR7, CDC14B, DUSP12, UBASH3B, PTPRZ1, DUSP4, PTPN1, EYA2, EYA3, EYA4, PTPN12, SSH2, DUSP26, PTPN13, PTPN14, PTPRD, PTPRE, PTPRB, PTPRC, PTPRA, PTPN9, PTPN5, PTPN2, PTPN3* |
| **brown** | **MF** | **GO:0000287~magnesiμm ion binding** | **70** | 1.11 | 0.00 | *ATP8A1, LRRK2, MAST2, XYLT2, ENO2, CLYBL, THG1L, RPS6KA3, STK11, HPGDS, RPS6KA5, RPS6KA2, PGM3, NUDT19, PGM5, PRKACB, ENOPH1, MAP3K5, ITPK1, ATP11C, ATP11B, IRAK4, ATP11A, ERN1, LATS1, MSH6, PPM1A, NT5C3B, PPM1B, PPA2, MSH2, RP2, SUCLG2, SIK2, TKT, EXD2, PIF1, ATP10D, GTPBP10, ATP10B, ATP10A, NLK, STK3, WRN, RNASEH1, NUAK2, TDG, DHX36, ABL1, ABL2, STK38L, HACL1, ATP9B, MORC2, ATP9A, AASDHPPT, SRPK2, ATP8B2, EYA2, ATP8B1, EPHX2, NEK6, PLK1, IDH2, CDC42BPA, SRPK1, SNRK, RHEB, UBA2, FARSB* |
| **brown** | **MF** | GO:0034452~dynactin binding | 7 | 0.11 | 0.00 | *GSK3B, SPTBN5, HTT, HOOK3, BICD1, BICD2, BBS4* |
| **brown** | **MF** | **GO:0000978~RNA polymerase II core promoter proximal region sequence-specific DNA binding** | **112** | 1.78 | 0.00 | *EHF, PRDM4, PRDM5, UBP1, RORB, NR3C1, RBPJ, ETS1, BACH1, CARF, BACH2, ELK3, ELK4, MYB, HOXA2, PKNOX1, MEF2C, KDM2B, TBR1, HNF1A, SOX10, RFX4, SUB1, HOXB3, ZNF395, GATA4, NRF1, FOXO3, DHX36, HESX1, HIVEP1, SKIL, TFAP2A, TFAP2B, ZBTB17, ZNF143, SMAD4, ZFHX3, POU2F1, TFAP2E, BCL11A, NFATC3, NR1D2, SMAD5, POU2F3, NFKB1, FLI1, RAX2, TFCP2, BCL6, SP3, GTF2IRD1, ZNF410, NFE2L2, GMEB1, HNF4G, NR2E1, CTCF, CHD2, LITAF, GLI3, GLI2, LRRFIP1, ZNF644, MYBL1, GABPA, NCOA2, KLF12, EED, ARNT, PROX1, RAD23B, ETV4, SIRT1, ETV6, NR5A1, ELF1, NR5A2, THRAP3, IRF4, MTF1, HAND1, PPARA, YAP1, NFAT5, PLAG1, SATB2, LEF1, NLRC5, HSF2, NRIP1, HSF4, E2F3, STAT6, E2F5, E2F6, MEF2D, SUZ12, TCF7L2, ARNT2, IRX3, FOXJ2, GRHL1, BATF, TBX15, KLF6, MEIS1, KLF5, NR6A1, NFIA, NFIB, ZNF335* |
| **brown** | **MF** | GO:0051219~phosphoprotein binding | 14 | 0.22 | 0.00 | *RB1, URI1, EPB41, SRC, DPYS, PKD2, IGF2R, MTOR, MID2, TOX3, TBK1, TBL2, UBASH3B, THRAP3* |
| **brown** | **MF** | GO:0019900~kinase binding | 25 | 0.40 | 0.00 | *RB1, BCAR3, MYOM1, DGKD, CAB39, SRC, WWC1, WWC2, HTT, PLG, PTPN22, JAKMIP3, EGFR, EIF4ENIF1, UBQLN1, PIP5K1A, PDE8A, PPP2R5A, MOB1B, TIAM1, NIN, PFKL, PER3, SNAI1, CNOT9* |
| **brown** | **MF** | GO:0005516~calmodulin binding | 36 | 0.57 | 0.00 | *CAMK2D, EPB41, CACNA1C, IQGAP1, IQGAP2, CAMSAP1, SLC8A1, STRN4, RYR3, CAMSAP2, STRN3, PPP3CA, PPP3CB, EEF2K, PPP3CC, CALD1, KCNN2, NOS1, KCNN3, CAMK2G, CDK5RAP2, KCNH1, NOS2, DAPK1, PHKB, PHKA1, MYO7A, PHKA2, SYT7, IQCG, ENKUR, MYO1E, KCNQ3, FAS, MAP6D1, PLCB1* |
| **brown** | **MF** | GO:0004535~poly(A)-specific ribonuclease activity | 9 | 0.14 | 0.01 | *CNOT6, PAN3, CNOT6L, CNOT1, CNOT2, PNLDC1, NOCT, PARN, CNOT8* |
| **brown** | **MF** | GO:0005545~1-phosphatidylinositol binding | 8 | 0.13 | 0.01 | *EEA1, ZFYVE16, EPB41, ZFYVE9, WDFY1, WDFY3, SNX9, PICALM* |
| **brown** | **MF** | GO:0061631~ubiquitin conjugating enzyme activity | 15 | 0.24 | 0.01 | *UBE2H, UBE2B, UBE2E3, UBE2D3, UBE2D1, UBE2G1, UBE2G2, UBE2J2, UBE2J1, UBE2R2, UBE2T, UBE2N, UBE2O, BIRC6, UBE2K* |
| **brown** | **MF** | GO:0043531~ADP binding | 11 | 0.17 | 0.01 | *MSH6, ERN1, PPP5C, APAF1, MSH2, CHORDC1, MYO18A, PRKAG2, TAP1, COQ8A, MYH10* |
| **brown** | **MF** | GO:0070064~proline-rich region binding | 7 | 0.11 | 0.01 | *YAP1, GAREM1, BAIAP2L1, NEDD4, WBP4, ITSN1, ABL1* |
| **brown** | **MF** | GO:0004970~ionotropic glutamate receptor activity | 10 | 0.16 | 0.01 | *GRIA2, GRID2, GRIN3B, GRIN2A, GRIK3, GRIK4, GRIK1, GRIA3, GRIN1, GRIA4* |
| **brown** | **MF** | GO:0008022~protein C-terminus binding | 39 | 0.62 | 0.01 | *YAP1, TOP2B, ECM1, SPTBN5, EPB41, NCF2, SRC, CEP120, YEATS4, BCL10, BRCA2, GRIP1, NIPBL, ATXN1, SNTG1, HESX1, ABL1, PTK2B, CEP250, EP300, KPNA3, SASH1, CEP135, XRCC4, XRCC5, VTA1, FN1, SNF8, PEX1, ATP1B1, TERF2, SIRT1, SREBF2, MSH2, PEX6, AGO2, PICK1, MAPRE3, ERCC6* |
| **brown** | **MF** | GO:0030374~ligand-dependent nuclear receptor transcription coactivator activity | 19 | 0.30 | 0.01 | *NCOA1, SS18, NCOA2, SLC30A9, KDM1A, PRKCB, NCOA6, NCOA3, ACTN1, SRA1, FGF2, ETS1, DCAF6, CNOT6, NCOA7, PPARGC1A, CNOT9, PPARGC1B, CCDC62* |
| **brown** | **MF** | GO:0070577~lysine-acetylated histone binding | 9 | 0.14 | 0.01 | *ZZZ3, PSME4, TRIM24, YEATS4, BRD7, MLLT3, ZZEF1, BRD4, ATAD2B* |
| **brown** | **MF** | GO:0003697~single-stranded DNA binding | 24 | 0.38 | 0.01 | *WDR48, MYEF2, XPC, RTF1, BRCA2, MLH1, SMC4, SMC2, RAD52, PURA, RAD51D, NEIL3, TEN1, FUBP3, MSH2, RNF138, MSH3, SUB1, DHX36, SPRTN, PMS2, SSBP3, SSBP4, RAD18* |
| **brown** | **MF** | GO:0005178~integrin binding | 31 | 0.49 | 0.02 | *SEMA7A, SRC, THY1, THBS1, FGF2, TNN, KDR, ITGAV, ITGB6, LGALS8, JAM2, CXADR, SYK, VWF, ACTN1, FN1, EMP2, NPNT, NISCH, ADAM17, FRMD5, CD40LG, JAML, COL4A3, ADAM9, ITGB1BP1, CD226, LCP1, PLPP3, UTRN, FBN1* |
| **brown** | **MF** | GO:0016301~kinase activity | 31 | 0.49 | 0.02 | *PLXND1, CCNH, ADPGK, PIK3CD, NUCKS1, PRKAG3, TBRG4, PACSIN2, PLXNA2, AKT1, IP6K1, GCN1, IP6K2, SPAG9, TPK1, MAPK8IP1, MOB1B, MOB2, ETNK1, FAM20B, PI4KA, FASTKD5, TAB3, PLXNB1, PI4KB, COQ8A, TAB1, MNAT1, PIK3AP1, SKAP1, SKAP2* |
| **brown** | **MF** | GO:0070530~K63-linked polyubiquitin binding | 10 | 0.16 | 0.02 | *ZRANB3, ZRANB1, RNF169, WDR81, UIMC1, SPRTN, ZBTB1, OTUD7B, TAB3, TAB2* |
| **brown** | **MF** | GO:0008094~DNA-dependent ATPase activity | 10 | 0.16 | 0.02 | *MSH6, RAD51B, RAD51D, BTAF1, RAD51C, MSH2, XRCC2, MYO18A, INO80, BPTF* |
| **brown** | **MF** | GO:0030165~PDZ domain binding | 20 | 0.32 | 0.02 | *NSF, FZD3, GRID2, CXADR, PLEKHA2, CRIM1, LPAR1, ARHGAP29, CLCN3, GNG12, ACVR2A, CIT, SLC9A3, ADAM17, CCDC88C, LNX1, TBC1D10A, CFTR, LNX2, LLGL2* |
| **brown** | **MF** | GO:0005049~nuclear export signal receptor activity | 7 | 0.11 | 0.02 | *NUP214, EIF4ENIF1, XPO1, XPO4, XPO6, RANBP17, XPO7* |
| **brown** | **MF** | **GO:0001228~transcriptional activator activity, RNA polymerase II transcription regulatory region sequence-specific binding** | **100** | 1.59 | 0.02 | *ATF2, EHF, PRDM4, UBP1, RORB, NR3C1, RBPJ, ETS1, IKZF3, BACH1, CARF, ELK3, ELK4, CREB3L2, MYB, TP63, MEF2C, MITF, LMX1A, RFX4, RFX5, HOXB3, ATF6, GCM2, CASZ1, ZNF395, DLX3, GATA4, FOXO3, GLIS1, TFAP2A, TFAP2B, ZBTB17, ZNF143, SMAD4, SMAD3, TFAP2E, NFATC3, ESR1, POU2F3, NFKB1, FLI1, FOSL2, RAX2, PHOX2A, TFCP2, ZFAT, LHX4, NFE2L2, BARHL1, GMEB1, CSRNP2, CSRNP3, HNF4G, NR2E1, HOXC13, LITAF, GLI2, MYBL1, GABPA, MYOG, TCF12, ETV4, ETV6, ELF1, NR5A2, TFDP2, IRF4, MTF1, IRF2, HAND1, IRF5, PPARA, NFAT5, HLF, PLAG1, SATB2, LEF1, HOXD10, BARX2, HSF2, E2F3, STAT6, MEF2D, ARNT2, NFYB, IRX3, FOXJ2, CDC5L, ZNF76, USF3, GRHL1, GRHL2, BATF, KLF6, MEIS1, KLF5, NR6A1, NFIA, NFIB* |
| **brown** | **MF** | GO:0015095~magnesiμm ion transmembrane transporter activity | 9 | 0.14 | 0.02 | *NIPAL4, MRS2, NIPAL1, NIPAL2, ZDHHC13, MAGT1, NIPA2, CNNM2, ZDHHC17* |
| **brown** | **MF** | GO:0030276~clathrin binding | 9 | 0.14 | 0.02 | *AFTPH, LRRK2, TOM1, AP2B1, PIK3C2A, AP4B1, SMAP1, CLINT1, PICALM* |
| **brown** | **MF** | GO:0005543~phospholipid binding | 22 | 0.35 | 0.02 | *UNC13B, CARMIL2, PRKCI, JAG1, SGIP1, MYOF, SYT16, RASAL1, ARHGAP26, SYTL5, SYTL4, ARHGAP35, NR5A1, PREX1, BAIAP2L2, NR5A2, BIN2, PSD2, RASA2, PSD3, SPTBN1, PACSIN1* |
| **brown** | **MF** | GO:0005547~phosphatidylinositol-3,4,5-trisphosphate binding | 12 | 0.19 | 0.02 | *ARHGAP9, ZFYVE16, HIP1R, BTK, ARAP2, DAPP1, ASAP1, IQGAP1, COMMD1, IQGAP2, OGT, FERMT2* |
| **brown** | **MF** | GO:0051539~4 iron, 4 sulfur cluster binding | 16 | 0.25 | 0.02 | *PRIM2, RTEL1, RSAD2, NUBPL, IREB2, RSAD1, ETFDH, ELP3, CDKAL1, SDHB, POLA1, DPYD, CDK5RAP1, ACO1, DNA2, MUTYH* |
| **brown** | **MF** | GO:0016740~transferase activity | 29 | 0.46 | 0.02 | *UHRF2, RNF13, UBE2D2, UBE2D3, CHM, UBE2D1, SGMS2, UBE2L3, RNF216, DNMT3B, PDSS2, PDSS1, CHAC1, UBE2F, RNF43, UBE2H, GGPS1, TSTD2, SIAH2, SIAH1, SIRT4, ALG14, UBE2G1, UBE2G2, NDUFAF6, RNF182, NAA35, NAA15, TKT* |
| **brown** | **MF** | GO:0002020~protease binding | 27 | 0.43 | 0.02 | *GSK3B, ECM1, SERPINC1, FURIN, BCL10, BRCA2, MALT1, DPP4, SH3PXD2A, CHL1, LONP2, DVL3, ITGAV, NFRKB, ATP9A, POLG, NTRK2, VWF, ITGA3, SLC2A13, FN1, TRIP4, BIN1, BANK1, KIT, CD28, BCL2* |
| **brown** | **MF** | GO:0003691~double-stranded telomeric DNA binding | 5 | 0.08 | 0.02 | *PURA, HMBOX1, XRCC5, ZBTB48, TERF2* |
| **brown** | **MF** | GO:0033192~calmodulin-dependent protein phosphatase activity | 5 | 0.08 | 0.02 | *PPM1A, PPP3CA, PPP3CB, PPP3CC, PPM1F* |
| **brown** | **MF** | GO:0070063~RNA polymerase binding | 10 | 0.16 | 0.03 | *TCERG1, CCNT2, CCNT1, BIN1, NEDD4, YTHDC2, GSG1, STOM, PKN2, BRCA1* |
| **brown** | **MF** | GO:0043014~alpha-tubulin binding | 13 | 0.21 | 0.03 | *DYSF, DLEC1, INO80, SPAST, FNTA, TAOK1, TBCE, TRIM36, DIP2B, DNAL1, BBS4, NDEL1, ARL8B* |
| **brown** | **MF** | GO:0019894~kinesin binding | 13 | 0.21 | 0.03 | *SPAG9, SPTBN5, KCNC1, NEK6, CLSTN1, AP1AR, MAPK8IP3, SHTN1, PRC1, AP1G1, PLEKHM2, KIFAP3, SYBU* |
| **brown** | **MF** | GO:0003684~damaged DNA binding | 17 | 0.27 | 0.03 | *XRCC6, XRCC5, XPA, REV1, XPC, BRCA1, RAD23B, DDB2, NEIL3, POLI, EP300, NBN, TP53BP1, POLK, TP63, POLH, CUL4B* |
| **brown** | **MF** | GO:0000993~RNA polymerase II core binding | 9 | 0.14 | 0.03 | *URI1, RECQL5, PCF11, AGO2, WAC, ELP2, RPRD1B, ZNF326, CDC73* |
| **brown** | **MF** | GO:0051721~protein phosphatase 2A binding | 9 | 0.14 | 0.03 | *MFHAS1, ANKLE2, PPME1, BCL2, SMG7, SMG5, STRN4, CTTNBP2NL, STRN3* |
| **brown** | **MF** | GO:0051010~microtubule plus-end binding | 9 | 0.14 | 0.03 | *CLIP2, KIF18A, CLIP1, APC, MAPRE3, CKAP5, MAPRE2, CLASP1, CLASP2* |
| **brown** | **MF** | GO:0004114~3',5'-cyclic-nucleotide phosphodiesterase activity | 7 | 0.11 | 0.03 | *PDE11A, PDE10A, PDE3B, PDE6C, PDE7B, PDE7A, PDE9A* |
| **brown** | **MF** | GO:0070679~inositol 1,4,5 trisphosphate binding | 7 | 0.11 | 0.03 | *TRPC5, TRPC6, TRPC4, TRPC1, PLCL2, ITPR1, ITPR2* |
| **brown** | **MF** | GO:0030145~manganese ion binding | 19 | 0.30 | 0.03 | *EXD2, B4GALT1, GALNT2, GALNT1, XYLT2, PEPD, PRIMPOL, PPM1A, WRN, PPM1B, XPNPEP1, XPNPEP3, MGAT5, ABL1, ABL2, LARGE1, B4GALT7, PPEF2, DCP2* |
| **brown** | **MF** | GO:0002039~p53 binding | 21 | 0.33 | 0.03 | *RNF20, GSK3B, BLM, KDM1A, USP10, HTT, RCHY1, DUSP26, SIRT1, NUAK1, STK11, RNF125, TP53BP2, TRIM24, EP300, KMT5A, TP53BP1, BRD7, TAF3, TP63, BRD4* |
| **brown** | **MF** | GO:0045505~dynein intermediate chain binding | 10 | 0.16 | 0.04 | *DYNC2H1, SPTBN5, DNAH17, DNAH8, DNAH5, HTT, DNAH9, DYNLRB2, HOOK3, BICD1* |
| **brown** | **MF** | GO:0003777~microtubule motor activity | 16 | 0.25 | 0.04 | *STARD9, KIF25, KIF24, KIF23, KIF17, KIF27, KIF3B, KIF2A, KIF16B, KIF5C, KIF5B, KIF13A, KIF13B, KIF21A, KIF1B, KIF3C* |
| **brown** | **MF** | GO:0003730~mRNA 3'-UTR binding | 16 | 0.25 | 0.04 | *RNF20, FMR1, ANGEL2, RC3H1, LARP4B, Pμm1, ELAVL1, TIAL1, CPEB1, PCBP4, GEMIN5, IGF2BP3, CPEB3, CPEB2, CPEB4, RBMS3* |
| **brown** | **MF** | GO:0005109~frizzled binding | 11 | 0.17 | 0.04 | *MYOC, RYK, CCDC88C, DVL1, WNT5A, DVL3, RSPO3, ROR2, WNT2, CTHRC1, LRP6* |
| **brown** | **MF** | GO:0005092~GDP-dissociation inhibitor activity | 6 | 0.10 | 0.04 | *GPSM2, CCDC88A, RGS14, CHM, SH3BP4, ITGB1BP1* |
| **brown** | **MF** | GO:0032454~histone demethylase activity (H3-K9 specific) | 6 | 0.10 | 0.04 | *PHF2, KDM3A, KDM3B, KDM1A, JMJD1C, KDM7A* |
| **brown** | **MF** | GO:0051959~dynein light intermediate chain binding | 8 | 0.13 | 0.04 | *DYNC2H1, DYNC1H1, DNAH17, DNAH8, DNAH5, DNAH9, RAB11FIP3, BICD2* |
| **brown** | **MF** | **GO:0003723~RNA binding** | **157** | 2.49 | 0.05 | *THμmPD2, THμmPD3, HNRNPR, YARS2, TIAL1, RBMX2, PAPOLG, CWC22, RAVER2, PAPOLA, CPNE1, PUS3, DDX18, RBFOX2, DIMT1, DDX10, DICER1, TRNT1, PUS7, CRNKL1, SND1, UHMK1, PRPF6, PSPC1, SRSF4, MATR3, SRSF5, SREK1, ANKRD17, ZCCHC8, KHDRBS2, YTHDC1, YTHDC2, CSTF2, RNPC3, HSP90B1, CTIF, LARP1, PCBP3, HNRNPDL, G3BP1, UBR5, G3BP2, PPARGC1A, PPARGC1B, BARD1, ZRSR2, ZRANB2, CPSF6, RBPMS, CPSF3, CPSF2, SYNJ2, Pμm1, LARP4, MEX3C, SNUPN, DNAJC17, Pμm3, CNOT4, MOV10, PPP5C, MEX3B, PAN3, SON, HNRNPK, SYNJ1, FUBP3, CAPRIN1, FUBP1, CAPRIN2, HBP1, GRSF1, SUGP1, FARSB, RBM27, RBM26, CELF1, CELF2, DDX42, ADAR, ZC3H6, BRCA1, TFB1M, RBM34, RBM5, RBM6, LRRC47, UPF2, UPF1, DIS3, DDX55, NSUN3, RBM19, NSUN7, RANGAP1, MRPS5, PTBP3, PSMA6, KIN, SLTM, ESRP2, ESRP1, ZNF638, C1D, RBMS1, RBM20, RBM24, DCP2, TRUB1, RNASEL, RBM47, RBM48, NUFIP2, STAU2, PARN, NOL8, MRM1, MRM3, MTHFSD, ENOX2, PURA, SCAF8, TRA2A, ATXN1L, DROSHA, GPBP1L1, RALY, SNRPB2, ZNF106, RPS2, EXOSC3, SRSF11, PAIP1, SPEN, SUZ12, RBM39, HNRNPA3, SF3A1, TAF15, YTHDF2, MYEF2, YTHDF3, ASCC1, KRR1, DDX31, SAMD4A, PUS7L, BICC1, SCAF4, TARBP1, ENOX1, ZNFX1, RBM41, RBM45, PPIL4, RBM43* |
| **cyan** | **BP** | GO:0007218~neuropeptide signaling pathway | 3 | 0.96 | 0.01 | *POMC, GAL, NTS* |
| **cyan** | **BP** | GO:0008209~androgen metabolic process | 2 | 0.64 | 0.03 | *AKR1D1, HSD17B7* |
| **cyan** | **CC** | GO:0005576~extracellular region | 8 | 2.57 | 0.02 | *POMC, DRAXIN, GAL, BGLAP, CILP2, NTS, C1QL2, PTHLH* |
| **cyan** | **CC** | GO:0005615~extracellular space | 7 | 2.25 | 0.02 | *POMC, GAL, WFIKKN2, DPEP1, SCG2, CBLN2, JAM3* |
| **cyan** | **MF** | GO:0015293~symporter activity | 3 | 0.96 | 0.01 | *SLC10A4, SLC6A15, SLC1A4* |
| **cyan** | **MF** | GO:0008375~acetylglucosaminyltransferase activity | 2 | 0.64 | 0.02 | *A4GNT, POMGNT1* |
| **cyan** | **MF** | GO:0005179~hormone activity | 3 | 0.96 | 0.04 | *POMC, GAL, PTHLH* |
| **cyan** | **MF** | GO:0005184~neuropeptide hormone activity | 2 | 0.64 | 0.04 | *GAL, NTS* |
| **grey** | **BP** | GO:1904851~positive regulation of establishment of protein localization to telomere | 4 | 0.42 | 0.00 | *DKC1, TCP1, CCT8, CCT7* |
| **grey** | **BP** | GO:0007268~chemical synaptic transmission | 6 | 0.63 | 0.01 | *GJD2, HTR1E, SYT10, HTR1D, SLITRK5, TAC1* |
| **grey** | **BP** | GO:0030326~embryonic limb morphogenesis | 5 | 0.53 | 0.01 | *HOXA10, SALL4, GJA5, ALX1, HOXC10* |
| **grey** | **BP** | GO:0042254~ribosome biogenesis | 5 | 0.53 | 0.01 | *NOP56, EBNA1BP2, NOP2, SNU13, NOC4L* |
| **grey** | **BP** | GO:0071320~cellular response to cAMP | 5 | 0.53 | 0.02 | *CRHBP, DMTN, AQP8, AQP9, INPP5K* |
| **grey** | **BP** | GO:1901998~toxin transport | 5 | 0.53 | 0.02 | *MEP1B, BNIP3, TCP1, CCT8, CCT7* |
| **grey** | **BP** | GO:0043065~positive regulation of apoptotic process | 12 | 1.27 | 0.02 | *PTPA, NR4A1, ALDH1A3, BMP2, PROC, WNT11, PIP5KL1, BNIP3, CALM1, MMP9, SLC27A4, SPDEF* |
| **grey** | **BP** | GO:0051258~protein polymerization | 3 | 0.32 | 0.03 | *FGB, FGA, CASQ2* |
| **grey** | **BP** | GO:0042730~fibrinolysis | 3 | 0.32 | 0.03 | *FGB, FGA, F2* |
| **grey** | **BP** | GO:0070072~vacuolar proton-transporting V-type ATPase complex assembly | 3 | 0.32 | 0.03 | *TMEM199, VMA21, ALDOB* |
| **grey** | **BP** | GO:0002027~regulation of heart rate | 4 | 0.42 | 0.03 | *CAV3, CASQ2, DRD2, CALM1* |
| **grey** | **BP** | GO:0009952~anterior/posterior pattern specification | 7 | 0.74 | 0.03 | *HOXA10, HOXA9, CDX4, HNF1B, ALX1, HOXB7, HOXC10* |
| **grey** | **BP** | GO:0007339~binding of sperm to zona pellucida | 4 | 0.42 | 0.03 | *TCP1, CCT8, CCT7, ZP2* |
| **grey** | **BP** | GO:0021520~spinal cord motor neuron cell fate specification | 3 | 0.32 | 0.04 | *ISL2, ISL1, HOXC10* |
| **grey** | **BP** | GO:0009749~response to glucose | 4 | 0.42 | 0.04 | *EGR1, HNF4A, HNF1B, SIDT2* |
| **grey** | **BP** | GO:0042531~positive regulation of tyrosine phosphorylation of STAT protein | 5 | 0.53 | 0.05 | *IL21, IFNG, ARL2BP, IL12A, ISL1* |
| **grey** | **BP** | GO:0051973~positive regulation of telomerase activity | 4 | 0.42 | 0.05 | *HSP90AA1, DKC1, TCP1, KLF4* |
| **grey** | **CC** | GO:0045202~synapse | 17 | 1.79 | 0.00 | *FGB, FGA, HTR1E, PTPRN, HTR1D, SLC6A11, CELF6, SLC6A5, GJD2, SRPX2, ARPC2, SYT10, SLITRK5, RPL37, DRD1, DRD2, TAC1* |
| **grey** | **CC** | **GO:0005634~nucleus** | **103** | 10.86 | 0.00 | *ERRFI1, NUDT1, FOXI1, PHAX, PRDM1, SOX21, ETS2, FERD3L, CRHBP, HOXA9, RASSF1, PTTG1, EME1, RAG2, HOXA4, RAG1, EOMES, NKX2-8, ZGPAT, DDX11, HARBI1, HNF1B, ISL1, CDC25A, ISL2, TBPL2, GMNC, SNRPB, SET, SYCP2L, HOXB13, RAD51AP1, STRA8, NTHL1, NEUROD6, INPP5K, OTX2, NKX2-5, NKX2-3, EIF2B5, EGR1, FOXN1, NR0B2, PRDX6, RPE65, CLK1, MAFB, ARL2BP, ADI1, PSMG2, VSX1, PIWIL2, HMX3, BARHL2, TRADD, PTPRN, ONECUT1, HMGB2, HSPB2, CELF6, AREG, HNF4A, SIX3, BOK, APOBEC1, KLF10, HSP90AA1, POU1F1, NME3, PAX5, PATL2, SMC1B, MLF2, DMRTA2, RIPPLY3, ELF3, HAND2, DMC1, HMX2, SF3B6, DBX2, DBX1, ASCL1, LRRC10, ATOH7, PARD6A, GBX2, PSMB3, ADAD1, SPDEF, DRGX, FOXB1, CDX4, SSB, GINS4, ZIC4, LBX1, TBX4, PSMC3, SERBP1, RBM46, MSRB1, TJP2* |
| **grey** | **CC** | GO:0005874~microtubule | 15 | 1.58 | 0.00 | *DNAH3, DNAH14, KATNB1, TUBB4B, CRHBP, TUBB6, RASSF1, PROC, HSPH1, TCP1, KIFC3, KIF2B, CCT8, CCT7, DNAL4* |
| **grey** | **CC** | GO:0005922~connexin complex | 5 | 0.53 | 0.00 | *GJD2, GJA1, GJD4, GJB1, GJA5* |
| **grey** | **CC** | GO:0031012~extracellular matrix | 9 | 0.95 | 0.00 | *TECTB, FBLN7, BCAN, MMP7, WNT11, MMP28, OTOG, MMP9, OC90* |
| **grey** | **CC** | GO:0005795~Golgi stack | 4 | 0.42 | 0.02 | *NSFL1C, TRAPPC4, MGAT2, GOLGA7* |
| **grey** | **CC** | GO:0005832~chaperonin-containing T-complex | 4 | 0.42 | 0.02 | *TCP1, CCT8, CCT7, CCT4* |
| **grey** | **CC** | GO:0101031~chaperone complex | 4 | 0.42 | 0.02 | *DNAAF2, PIH1D2, SDF2, PSMG2* |
| **grey** | **CC** | GO:0005689~U12-type spliceosomal complex | 4 | 0.42 | 0.02 | *SF3B6, SNRNP25, SNRNP35, SNRPB* |
| **grey** | **CC** | GO:0033116~endoplasmic reticulμm-Golgi intermediate compartment membrane | 4 | 0.42 | 0.03 | *TMEM199, VMA21, TMED3, TMED7* |
| **grey** | **CC** | GO:0005783~endoplasmic reticulμm | 23 | 2.43 | 0.03 | *FGB, CDS1, LRRC59, HHATL, CAV3, RPS8, TRAPPC4, HSD17B3, RNF19B, TMC6, LCTL, TECRL, HSPA13, AGPAT2, HSD11B2, GJA1, STC2, ALB, SERPINH1, AGR3, INPP5K, SDF2, ZP2* |
| **grey** | **CC** | GO:0005667~transcription factor complex | 9 | 0.95 | 0.04 | *HOXA10, NR4A1, HOXA9, HAX1, HAND2, HNF1B, ALX1, HOXB13, KLF4* |
| **grey** | **CC** | GO:0009897~external side of plasma membrane | 11 | 1.16 | 0.04 | *ITGB1, FGB, CD79B, FGA, CHRNA4, ATP6AP2, CCR7, ENPP3, SLC4A3, F2, IL17A* |
| **grey** | **MF** | GO:0001228~transcriptional activator activity, RNA polymerase II transcription regulatory region sequence-specific binding | 26 | 2.74 | 0.00 | *BARHL2, HOXC10, HOXA10, HOXA9, GBX2, NEUROD6, HNF4A, SIX3, E4F1, OTX2, ALX1, NKX2-5, KLF10, EGR1, CDX4, POU1F1, PAX5, KLF4, FOXN1, ISL1, NR4A2, NR4A1, ELF3, HAND2, HOXB7, ATF3* |
| **grey** | **MF** | GO:0000981~RNA polymerase II transcription factor activity, sequence-specific DNA binding | 19 | 2.00 | 0.00 | *NKX2-8, HMX3, POU1F1, DBX2, DBX1, SOX11, HNF1B, LBX1, HOXB13, NHLH2, ISL2, HOXA9, OTX2, NKX2-5, HMX2, VSX1, NKX2-3, HOXA4, DRGX* |
| **grey** | **MF** | GO:0044183~protein binding involved in protein folding | 5 | 0.53 | 0.00 | *CALR3, HSP90AA1, TCP1, CCT8, CCT7* |
| **grey** | **MF** | GO:0005102~receptor binding | 12 | 1.27 | 0.00 | *FGB, PTPA, FGA, WNT10A, SRPX2, DMTN, WNT11, PLA2G1B, HNF4A, REN, F2, BOK* |
| **grey** | **MF** | GO:0042803~protein homodimerization activity | 28 | 2.95 | 0.01 | *CCDC103, JCHAIN, PTPA, PSMD7, STC2, HNF4A, MGAT2, NKX2-5, XDH, GYG1, CER1, RAB11FIP4, BOK, RAG1, HSP90AA1, TPI1, BNIP3, GSS, IDH1, COQ9, NR0B2, ALDH1A3, NUDT21, HAND2, SYT10, CHMP4C, ATF3, IL17A* |
| **grey** | **MF** | GO:1990837~sequence-specific double-stranded DNA binding | 12 | 1.27 | 0.01 | *EOMES, ATOH7, FOXB1, DMRTA2, BARHL2, ISL2, POU1F1, ZIC4, HOXB13, NKX2-3, DRGX, SPDEF* |
| **grey** | **MF** | GO:0005540~hyaluronic acid binding | 5 | 0.53 | 0.01 | *BCAN, NCAN, STAB2, HMMR, LYVE1* |
| **grey** | **MF** | GO:0051082~unfolded protein binding | 8 | 0.84 | 0.01 | *CALR3, HSP90AA1, DNAJA2, TCP1, SERPINH1, CCT8, CCT7, CCT4* |
| **grey** | **MF** | GO:0005254~chloride channel activity | 5 | 0.53 | 0.03 | *CLIC6, GABRR3, CLIC3, TTYH2, GABRA3* |
| **grey** | **MF** | GO:0000978~RNA polymerase II core promoter proximal region sequence-specific DNA binding | 19 | 2.00 | 0.03 | *KLF10, CDX4, ZGPAT, ONECUT1, SOX11, PAX5, KLF4, HOXA10, NR4A2, HOXA9, ELF3, NEUROD6, HNF4A, SIX3, OTX2, MSC, NKX2-5, HOXB7, ATF3* |
| **grey** | **MF** | GO:0005504~fatty acid binding | 3 | 0.32 | 0.04 | *FABP1, HNF4A, ALB* |
| **grey** | **MF** | GO:0043565~sequence-specific DNA binding | 10 | 1.05 | 0.04 | *NR4A1, FOXB1, ZGPAT, ELF3, HNF4A, FOXI1, FOXN1, ETS2, RAG1, SPDEF* |
| **grey** | **MF** | GO:0030515~snoRNA binding | 3 | 0.32 | 0.05 | *NOP56, NUDT7, NUDT1* |
| **grey** | **MF** | GO:0032794~GTPase activating protein binding | 3 | 0.32 | 0.05 | *NKIRAS2, PIN1, PLCD1* |
| **grey** | **MF** | GO:0022857~transmembrane transporter activity | 9 | 0.95 | 0.05 | *SLC5A7, GJA1, SLC5A9, SLCO1A2, SLC17A6, SLCO5A1, SLC7A11, SLC18A3, MFSD10* |
| **lightcyan** | **BP** | GO:0006936~muscle contraction | 4 | 2.78 | 0.00 | *MYBPC1, ACTN2, MYL1, MYOM2* |
| **lightcyan** | **BP** | GO:0045214~sarcomere organization | 4 | 2.78 | 0.00 | *ACTN2, KLHL41, MYOM2, LMOD2* |
| **lightcyan** | **BP** | GO:0035914~skeletal muscle cell differentiation | 4 | 2.78 | 0.00 | *MYOD1, ANKRD1, KLHL41, MYF5* |
| **lightcyan** | **BP** | GO:0048741~skeletal muscle fiber development | 3 | 2.08 | 0.00 | *MYOD1, KLHL40, KLHL41* |
| **lightcyan** | **BP** | GO:0007517~muscle organ development | 3 | 2.08 | 0.00 | *UNC45B, MYOD1, MYF5* |
| **lightcyan** | **BP** | GO:0055008~cardiac muscle tissue morphogenesis | 2 | 1.39 | 0.02 | *MYLK2, ANKRD1* |
| **lightcyan** | **BP** | GO:0042438~melanin biosynthetic process | 2 | 1.39 | 0.02 | *TYRP1, TYR* |
| **lightcyan** | **BP** | GO:0055010~ventricular cardiac muscle tissue morphogenesis | 2 | 1.39 | 0.03 | *TNNC1, MYL3* |
| **lightcyan** | **BP** | GO:0007274~neuromuscular synaptic transmission | 2 | 1.39 | 0.04 | *CHRNA1, MYLK2* |
| **lightcyan** | **CC** | GO:0031430~M band | 3 | 2.08 | 0.00 | *KLHL41, MYOM2, LMOD2* |
| **lightcyan** | **CC** | GO:0031674~I band | 3 | 2.08 | 0.00 | *MYL3, ANKRD1, KLHL40* |
| **lightcyan** | **CC** | GO:0030016~myofibril | 3 | 2.08 | 0.00 | *MYL1, MYOD1, ABRA* |
| **lightcyan** | **CC** | GO:0005667~transcription factor complex | 4 | 2.78 | 0.00 | *GCM1, MYOD1, LHX1, ANKRD1* |
| **lightcyan** | **CC** | GO:0033162~melanosome membrane | 2 | 1.39 | 0.03 | *TYRP1, TYR* |
| **lightcyan** | **MF** | GO:0004503~monophenol monooxygenase activity | 2 | 1.39 | 0.01 | *TYRP1, TYR* |
| **lightcyan** | **MF** | GO:0005509~calciμm ion binding | 7 | 4.86 | 0.01 | *ACTN2, MYL1, CAPSL, TNNC1, MYL3, TNNC2, HABP2* |
| **lightcyan** | **MF** | GO:0001228~transcriptional activator activity, RNA polymerase II transcription regulatory region sequence-specific binding | 5 | 3.47 | 0.01 | *GCM1, NKX6-3, MYOD1, OTX1, MYF5* |
| **lightcyan** | **MF** | GO:0000981~RNA polymerase II transcription factor activity, sequence-specific DNA binding | 4 | 2.78 | 0.02 | *NKX6-3, BSX, LHX1, ARX* |
| **lightcyan** | **MF** | GO:0031432~titin binding | 2 | 1.39 | 0.03 | *ACTN2, ANKRD1* |
| **lightcyan** | **MF** | GO:0005179~hormone activity | 3 | 2.08 | 0.04 | *EDN3, NPY, CRH* |
| **lightgreen** | **BP** | GO:0000122~negative regulation of transcription from RNA polymerase II promoter | 4 | 3.45 | 0.01 | *SOX2, NKX2-1, FHL2, PRDM14* |
| **lightgreen** | **BP** | GO:0021983~pituitary gland development | 2 | 1.72 | 0.02 | *SOX2, NKX2-1* |
| **lightgreen** | **BP** | GO:0001501~skeletal system development | 2 | 1.72 | 0.04 | *HOXD13, HOXD12* |
| **purple** | **BP** | GO:1900264~positive regulation of DNA-directed DNA polymerase activity | 3 | 1.18 | 0.01 | *RFC5, RFC4, RFC2* |
| **purple** | **BP** | GO:0035924~cellular response to vascular endothelial growth factor stimulus | 3 | 1.18 | 0.01 | *MAP2K3, ANXA1, SPHK1* |
| **purple** | **BP** | GO:0090303~positive regulation of wound healing | 3 | 1.18 | 0.01 | *CLDN4, ANXA1, HRAS* |
| **purple** | **CC** | GO:0016324~apical plasma membrane | 10 | 3.92 | 0.00 | *SLC34A2, KCNE1, ANXA1, CLDND1, ATP6V1B2, ATP1A1, SLC38A3, SLC26A4, ATP12A, CTSB* |
| **purple** | **CC** | GO:0005663~DNA replication factor C complex | 3 | 1.18 | 0.00 | *RFC5, RFC4, RFC2* |
| **purple** | **CC** | GO:0005757~mitochondrial permeability transition pore complex | 3 | 1.18 | 0.00 | *PPIF, SLC25A5, SLC25A4* |
| **purple** | **CC** | GO:0031390~Ctf18 RFC-like complex | 3 | 1.18 | 0.00 | *RFC5, RFC4, RFC2* |
| **purple** | **CC** | GO:0031090~organelle membrane | 5 | 1.96 | 0.04 | *TSPO2, POLR3H, ATP1A1, EIF2S1, EXOSC2* |
| **purple** | **CC** | GO:0005905~clathrin-coated pit | 3 | 1.18 | 0.05 | *EPN3, SPHK1, AP1S2* |
| **purple** | **MF** | GO:0003743~translation initiation factor activity | 6 | 2.35 | 0.00 | *EIF2S3, EIF2B2, EIF3F, EIF2S2, EIF2S1, EIF1B* |
| **purple** | **MF** | GO:0005524~ATP binding | 30 | 11.76 | 0.00 | *NIM1K, TOR2A, DDX49, SPO11, KIF12, ATP1A1, ATP12A, AK9, MKNK2, PIM1, MAP2K3, RFC5, HSPA9, PLK3, CCT2, RFC4, EIF2B2, RFC2, STRADB, DCAKD, SPHK1, CTPS1, ASS1, MAPK13, NMNAT1, KATNAL2, PSMC1, STK17B, ATP6V1B2, ABCG4* |
| **purple** | **MF** | GO:0016887~ATPase activity | 10 | 3.92 | 0.00 | *HSPA9, RFC5, CCT2, ATP6V1G1, RFC4, DDX49, KATNAL2, RFC2, PSMC1, ABCG4* |
| **purple** | **MF** | GO:0003689~DNA clamp loader activity | 3 | 1.18 | 0.00 | *RFC5, RFC4, RFC2* |
| **purple** | **MF** | GO:0017116~single-stranded DNA-dependent ATP-dependent DNA helicase activity | 3 | 1.18 | 0.00 | *RFC5, RFC4, RFC2* |
| **purple** | **MF** | GO:0030170~pyridoxal phosphate binding | 4 | 1.57 | 0.02 | *ALAS1, CTH, TAT, GCAT* |
| **purple** | **MF** | GO:0019899~enzyme binding | 6 | 2.35 | 0.03 | *RFC5, RFC4, COPS5, RFC2, NOXO1, APBA3* |
| **purple** | **MF** | GO:0005391~sodiμm:potassiμm-exchanging ATPase activity | 2 | 0.78 | 0.05 | *ATP1A1, ATP12A* |
| **purple** | **MF** | GO:0017077~oxidative phosphorylation uncoupler activity | 2 | 0.78 | 0.05 | *SLC25A5, SLC25A4* |
| **tan** | **BP** | GO:0006412~translation | 51 | 2.27 | 0.00 | *RPL5, MRPS15, RPL3, MRPS16, RPL32, RPL31, MRPS11, RPL8, RPL10A, RPL9, MRPL32, MRPL33, RPS14, RPS17, MRPL2, RPS16, RPL18A, RPL35, RPS11, RPL39, RPS13, RPS12, RPS7, RPL21, RPL23, RPS6, RPL22, MRPS23, MRPS21, RPS3A, RPSA, MRPL51, MRPL18, MRPL17, MRPL20, RPS15A, RPS3, RPS27A, RPL19, RPL35A, RPL23A, EEF2, RPS28, RPS29, RPL27A, RPL22L1, RPS20, RSL24D1, RPS21, RPL26L1, RPS23* |
| **tan** | **BP** | GO:0071816~tail-anchored membrane protein insertion into ER membrane | 6 | 0.27 | 0.00 | *CAMLG, EMC3, EMC6, EMC7, EMC8, GET4* |
| **tan** | **BP** | GO:0032482~Rab protein signal transduction | 6 | 0.27 | 0.00 | *RAB21, RAB4A, RAB35, RAB39A, RAB9B, RAB33A* |
| **tan** | **BP** | GO:0007059~chromosome segregation | 9 | 0.40 | 0.00 | *CENPT, DSN1, CENPW, DDX3X, MIS18A, NTMT1, PPP1R7, RPS3, CIAO1* |
| **tan** | **BP** | GO:0006614~SRP-dependent cotranslational protein targeting to membrane | 5 | 0.22 | 0.00 | *SRP19, SRP72, SSR3, SRPRA, SRP9* |
| **tan** | **BP** | GO:0043065~positive regulation of apoptotic process | 22 | 0.98 | 0.00 | *ENDOG, DDX3X, DAPK3, RPS6, OSGIN1, TEX261, GADD45G, SAV1, SYCE3, LGALS2, SFRP2, ACVR1C, SMPD1, NEURL1, ARL6IP5, SAP18, EMILIN2, ZNF622, HRG, CALM2, RNF122, TGM2* |
| **tan** | **BP** | GO:0019229~regulation of vasoconstriction | 6 | 0.27 | 0.00 | *EDN2, TBXA2R, AGTR1, HTR1A, ADRA1B, ADRA2B* |
| **tan** | **BP** | GO:0006457~protein folding | 14 | 0.62 | 0.01 | *PPIL1, P3H1, DNAJB13, CCT6A, NPPC, DNAJB5, DNAJB4, GRPEL2, DNAJB11, PPIH, PPIB, FKBP6, PPID, PPIC* |
| **tan** | **BP** | GO:0019395~fatty acid oxidation | 4 | 0.18 | 0.01 | *PRKAA1, ADIPOQ, ADIPOR1, HAO2* |
| **tan** | **BP** | GO:0010906~regulation of glucose metabolic process | 6 | 0.27 | 0.01 | *IGFBP4, ADIPOQ, PDK4, MLYCD, ADIPOR1, ZMPSTE24* |
| **tan** | **BP** | GO:0001732~formation of cytoplasmic translation initiation complex | 5 | 0.22 | 0.02 | *EIF3M, EIF3L, EIF3H, EIF3D, EIF3B* |
| **tan** | **BP** | GO:0090297~positive regulation of mitochondrial DNA replication | 3 | 0.13 | 0.02 | *STOML2, ENDOG, SSBP1* |
| **tan** | **BP** | GO:0045050~protein insertion into ER membrane by stop-transfer membrane-anchor sequence | 4 | 0.18 | 0.02 | *EMC3, EMC6, EMC7, EMC8* |
| **tan** | **BP** | GO:0007193~adenylate cyclase-inhibiting G-protein coupled receptor signaling pathway | 7 | 0.31 | 0.02 | *HTR1F, PALM, S1PR1, GNAI3, S1PR3, OPRM1, AKAP5* |
| **tan** | **BP** | GO:0032981~mitochondrial respiratory chain complex I assembly | 7 | 0.31 | 0.02 | *NDUFS8, NDUFB10, NDUFB6, NDUFA12, NDUFB4, NDUFS3, TIMM21* |
| **tan** | **BP** | GO:0006120~mitochondrial electron transport, NADH to ubiquinone | 5 | 0.22 | 0.03 | *NDUFB8, NDUFS8, NDUFB6, NDUFS3, DLD* |
| **tan** | **BP** | GO:0006915~apoptotic process | 17 | 0.76 | 0.03 | *GSDMA, MTFP1, SIVA1, CIDEA, NAIF1, TNFRSF11B, CIB1, AKAP1, SAV1, LGALS1, SFRP2, IRF1, RPS3, VDAC1, CIAPIN1, EBAG9, MCL1* |
| **tan** | **BP** | GO:0042593~glucose homeostasis | 12 | 0.53 | 0.03 | *G6PC2, CNR1, GCGR, RPS6, ADIPOQ, CRY1, PDK4, GCG, PARK7, ADIPOR1, ADIPOR2, PDK2* |
| **tan** | **BP** | GO:2000179~positive regulation of neural precursor cell proliferation | 5 | 0.22 | 0.03 | *DLL4, DISP3, GNG5, MDK, FZD9* |
| **tan** | **BP** | GO:0032781~positive regulation of ATPase activity | 5 | 0.22 | 0.03 | *MYL4, CASR, ATP1B3, DNAJC9, HSPA2* |
| **tan** | **BP** | GO:0042832~defense response to protozoan | 5 | 0.22 | 0.03 | *CD40, IL4R, SLC11A1, IL12B, IRF8* |
| **tan** | **BP** | GO:0046321~positive regulation of fatty acid oxidation | 3 | 0.13 | 0.03 | *C1QTNF2, NR4A3, MLYCD* |
| **tan** | **BP** | GO:0009249~protein lipoylation | 3 | 0.13 | 0.03 | *LIPT2, LIPT1, LIAS* |
| **tan** | **BP** | GO:0042775~mitochondrial ATP synthesis coupled electron transport | 3 | 0.13 | 0.03 | *NDUFA12, NDUFV3, NDUFV1* |
| **tan** | **BP** | GO:0000398~mRNA splicing, via spliceosome | 13 | 0.58 | 0.04 | *DBR1, PPIL1, EIF4A3, U2AF1, CWC15, ZCRB1, LSM5, PRPF19, LSM4, LSM8, PHF5A, DHX38, SNIP1* |
| **tan** | **BP** | GO:0006099~tricarboxylic acid cycle | 6 | 0.27 | 0.04 | *MDH1, MRPS36, SUCLG1, SDHD, SDHA, IDH3A* |
| **tan** | **BP** | GO:0030308~negative regulation of cell growth | 11 | 0.49 | 0.04 | *CDKN1A, DDX3X, SFRP2, SERPINE2, HYAL2, NAIF1, GDF2, OSGIN1, NDUFS3, HRG, CRLF3* |
| **tan** | **BP** | GO:0006979~response to oxidative stress | 10 | 0.45 | 0.04 | *PRDX4, NDUFS8, RRM2B, NDUFA12, GPX3, NDUFB4, OGG1, NEIL1, GCLM, LIAS* |
| **tan** | **BP** | GO:0032006~regulation of TOR signaling | 4 | 0.18 | 0.04 | *LAMTOR3, TTI1, ZMPSTE24, FBXO9* |
| **tan** | **BP** | GO:0006885~regulation of pH | 5 | 0.22 | 0.04 | *SLC9A2, SLC9A4, SLC9A5, PDK4, PDK2* |
| **tan** | **BP** | GO:0001525~angiogenesis | 14 | 0.62 | 0.04 | *EDN2, EPAS1, FLT4, CIB1, SAT1, PLXDC1, TMEM100, PDCL3, HEY1, PECAM1, S1PR1, ACKR3, ANGPTL4, GLUL* |
| **tan** | **BP** | GO:0008286~insulin receptor signaling pathway | 7 | 0.31 | 0.05 | *CAV2, PDK4, IRS4, FOXO4, DNAI1, PDK2, SLC2A8* |
| **tan** | **BP** | GO:0016226~iron-sulfur cluster assembly | 5 | 0.22 | 0.05 | *ISCA2, CIAPIN1, NUBP1, CIAO1, NUBP2* |
| **tan** | **CC** | GO:0005747~mitochondrial respiratory chain complex I | 11 | 0.49 | 0.00 | *NDUFA13, NDUFB8, NDUFS8, NDUFB10, NDUFB6, NDUFA12, NDUFB4, NDUFS3, NDUFC1, NDUFV3, NDUFV1* |
| **tan** | **CC** | GO:0005840~ribosome | 41 | 1.83 | 0.00 | *RPL5, MRPS15, RPL3, RPL32, RPL31, RPLP1, HSPA14, RPL9, MRPL54, MRPL33, MRPL41, RPS14, MRPL20, RPS17, MRPL2, RPS16, RPS15A, RPL18A, RPS11, RPL39, RPS13, RPS12, RPS7, RPL21, MRPS25, RPL23, RPS6, RPL22, MRPS23, MRPL28, RPL35A, MRPS21, RPL23A, RPS25, RPS28, MRPL51, RPS29, RPL24, RPL22L1, RSL24D1, RPS21* |
| **tan** | **CC** | GO:0022627~cytosolic small ribosomal subunit | 9 | 0.40 | 0.00 | *MCTS1, DDX3X, RPS6, RPS3, RPS20, RPS3A, RPSA, RPS11, RPS27A* |
| **tan** | **CC** | GO:0005759~mitochondrial matrix | 17 | 0.76 | 0.00 | *MARS2, ETFA, PARK7, ERAL1, SOD2, COASY, COQ5, NFS1, ACADL, TRMT5, GRPEL2, RPS3, PDK4, DLAT, MLYCD, PDK2, IDH3A* |
| **tan** | **CC** | GO:0005739~mitochondrion | 59 | 2.63 | 0.00 | *ISCA2, SLC25A3, ACAA2, OGG1, TXN2, MRPL41, LACTB, HEBP2, CA5A, ANXA6, GLUL, TGM2, GSTK1, HSDL2, GABARAPL1, ENDOG, MRPS25, CMC2, PUS1, MRPS23, AMT, SIRT5, TFB2M, NIPSNAP1, HPDL, RRM2B, PEX5, DDIT4, CRY1, PGAM5, MTFR1, FKBP4, LIPT2, SLC22A4, AARS2, NAXE, MRPS35, TIMM9, NAIF1, LIAS, MRPL54, AKAP1, PRDX3, FAM124B, CRYM, PUSL1, TSPOAP1, MCL1, CIDEA, GATC, ACADSB, MCAT, ETNPPL, DHODH, TIMM10B, ALDH6A1, PINK1, AMACR, ABCE1* |
| **tan** | **CC** | GO:0072546~ER membrane protein complex | 5 | 0.22 | 0.00 | *MMGT1, EMC3, EMC6, EMC7, EMC8* |
| **tan** | **CC** | GO:0044297~cell body | 9 | 0.40 | 0.00 | *ACTA2, ACTA1, TMEM132E, NAXE, RPS6, SLC1A2, EVX1, PARK7, GLUL* |
| **tan** | **CC** | GO:0005762~mitochondrial large ribosomal subunit | 10 | 0.45 | 0.00 | *MRPL20, MRPL50, MRPL2, MRPL51, MRPL18, MRPL17, MRPL28, MRPL46, MRPL32, MRPL44* |
| **tan** | **CC** | GO:0005844~polysome | 6 | 0.27 | 0.01 | *RPS6, DRG1, RPS3, MSI1, VIM, EEF2* |
| **tan** | **CC** | GO:0022625~cytosolic large ribosomal subunit | 8 | 0.36 | 0.01 | *RPL30, RPL3, RPL32, RPLP1, RPL35, RPL8, RPL7, RPL19* |
| **tan** | **CC** | GO:0016282~eukaryotic 43S preinitiation complex | 5 | 0.22 | 0.01 | *EIF3M, EIF3L, EIF3H, EIF3D, EIF3B* |
| **tan** | **CC** | GO:0033290~eukaryotic 48S preinitiation complex | 5 | 0.22 | 0.01 | *EIF3M, EIF3L, EIF3H, EIF3D, EIF3B* |
| **tan** | **CC** | GO:0005783~endoplasmic reticulμm | 44 | 1.96 | 0.01 | *GHDC, SAR1A, GOLT1B, SAR1B, CTSZ, TECR, ZDHHC6, MLC1, CIB1, UFSP2, PARK7, ZDHHC22, CLN6, TMEM100, PRDX4, SRP72, RPS3, DNAJB9, UBXN4, HMGCLL1, TGM2, TRAPPC3, BCHE, LYPLA1, PCYT1B, MAP2K1, SRD5A1, RPS6, ADIPOQ, P3H1, DOLK, HPS6, FOS, KCNRG, CGRRF1, BACE1, MGAT4D, PDCL3, RCN2, FNDC4, FIBIN, FKBP7, STUB1, PLPP2* |
| **tan** | **CC** | GO:0005576~extracellular region | 79 | 3.52 | 0.01 | *CNTF, HSP90AB1, MSTN, WFDC1, ADM, SPATA20, QRFP, FGF5, NPPC, FGF7, C1QTNF2, LGALS1, MDK, PRADC1, SMPD1, C1QTNF5, EMILIN2, ADAMTS8, ENPP6, B2M, TSHB, CHGB, WNT5B, ANXA2, IGFBP4, ENTPD5, FST, ADIPOQ, NOG, IGFBP2, DCN, C1QL1, SFRP4, ADCYAP1, OLFM1, UTS2B, CFAP221, TWSG1, SFRP2, RBP3, NPC2, FIBIN, PLA1A, ZP4, FGL1, NTN4, WNT8B, PLA2G3, TXN, NID1, FSTL5, ADAMTS15, APOO, OLFML3, FRZB, ARSK, IGFBP7, SFN, DNAI1, BCHE, ST14, NMB, Lμm, TGFB3, LGI2, GDF2, APOC3, GCG, CP, KAZALD1, BMP5, MFAP5, FGF16, TCN2, SST, CD9, VIP, HRG, LGMN* |
| **tan** | **CC** | GO:0008250~oligosaccharyltransferase complex | 5 | 0.22 | 0.02 | *RPN2, DAD1, OSTC, STT3A, DDOST* |
| **tan** | **CC** | GO:0071541~eukaryotic translation initiation factor 3 complex, eIF3m | 4 | 0.18 | 0.02 | *EIF3M, EIF3H, EIF3D, EIF3B* |
| **tan** | **CC** | GO:0005852~eukaryotic translation initiation factor 3 complex | 4 | 0.18 | 0.02 | *DDX3X, EIF3L, EIF3H, EIF3D* |
| **tan** | **CC** | GO:0005838~proteasome regulatory particle | 4 | 0.18 | 0.02 | *PSMD12, PSMC5, PSMD13, PSMD3* |
| **tan** | **CC** | GO:0005777~peroxisome | 10 | 0.45 | 0.03 | *GSTK1, HSDL2, ABCD2, AMACR, EHHADH, HSD17B4, VIM, MLYCD, ACAA1, DDO* |
| **tan** | **CC** | GO:0005789~endoplasmic reticulμm membrane | 40 | 1.78 | 0.05 | *BECN1, TMED10, MYRF, INSIG2, EIF5A2, TECR, GNAI3, PSEN2, SRI, RTN4, ZMPSTE24, RAB21, APOO, PTDSS2, EXTL2, UCHL1, SERP1, DDRGK1, ORMDL3, BCAP29, TMEM208, TMED2, EMC7, TMED5, PEX16, ICMT, RNF26, SSR2, SSR3, ALG3, ERLIN2, TEX2, G6PC2, DHRS7, PEMT, DEGS1, PIGM, TMBIM6, SEC22B, TMEM35A* |
| **tan** | **CC** | GO:0005730~nucleolus | 50 | 2.23 | 0.05 | *SRP19, MRPS15, CDKN1A, RPL3, DDX47, PWP1, TXN2, RRP9, SNAPC5, HABP4, MAF1, METTL18, GET4, RPS6, DNTTIP1, HDHD3, JMJD6, FTSJ3, GNL3, RCN2, NCL, FRG1, CMPK1, FAM32A, RRN3, DDX27, MAK16, DCTN3, NOL9, CLN6, DDRGK1, RPS3, RRS1, TP53TG5, ZNF622, NOP16, NIFK, TAF13, UTP3, APTX, NLE1, WDR55, CPS1, EIF6, PIN4, CEP85, CIAPIN1, RSL24D1, POLR3K, RAN* |
| **tan** | **MF** | GO:0003735~structural constituent of ribosome | 57 | 2.54 | 0.00 | *RPL5, MRPS15, RPL30, RPL3, MRPS16, RPL32, RPL31, RPLP1, MRPS11, RPL8, RPL10A, RPL9, MRPL32, RPL7, MRPL33, RPS14, RPS17, MRPL2, RPS16, RPL18A, RPL35, RPS11, RPL39, RPS13, RPS12, RPS7, RPL21, MRPS25, RPL23, RPS6, RPL22, MRPS23, MRPS21, RPS3A, RPSA, MRPL46, MRPL51, MRPS35, MRPL18, MRPL17, MRPL20, RPS15A, RPS3, RPS27A, RPL19, MRPL28, RPL35A, RPL23A, RPS28, RPS29, RPL27A, RPL22L1, RPS20, RSL24D1, RPS21, RPL26L1, RPS23* |
| **tan** | **MF** | GO:0051536~iron-sulfur cluster binding | 6 | 0.27 | 0.00 | *ISCA2, NFS1, GLRX3, PPAT, GLRX5, NUBP2* |
| **tan** | **MF** | GO:0003743~translation initiation factor activity | 12 | 0.53 | 0.00 | *EIF4A2, EIF3M, RRN3, EIF6, EIF3L, EIF5A2, EIF4EBP1, FIBIN, EIF3H, EIF3D, EIF4G2, EIF3B* |
| **tan** | **MF** | GO:0003924~GTPase activity | 36 | 1.60 | 0.00 | *RAB1A, ARF4, ARF1, DDX3X, SAR1A, SAR1B, ATL1, RASL11A, DIRAS2, GNAI3, ARL1, RAB21, REM1, RASD1, RAB26, MTG1, ARHGDIB, RAB29, ARL16, ARL11, RAB39A, RHOC, EEF2, RHOA, GEM, RAB33A, RAB11B, RAB32, EEF1A1, RRAGD, RAB35, DRG1, SRPRA, RAB19, RAN, RAB9B* |
| **tan** | **MF** | GO:0042802~identical protein binding | 84 | 3.74 | 0.01 | *GMEB2, MSTN, HTR2C, MLC1, MSI1, PRPF19, SAT1, ACTB, PIK3CG, C1QTNF2, YWHAQ, TRIM29, MPC2, NAMPT, ANXA6, TNFSF11, IL12B, MLYCD, GLUL, YWHAH, SUN2, SH3GLB2, GLRX3, GPX3, APLP2, DYNLT3, RPL22, AMPD1, FOS, FRS3, JMJD6, PSME3, ANGPTL4, SPATA18, SQSTM1, FKBP6, STX1A, ATF4, ZP4, SMIM3, BECN1, NRN1, KHDRBS3, NAXE, BRI3, ATL1, HTRA1, FOXO4, SDSL, ADIPOR1, ADIPOR2, ARPP19, SAV1, CRYZ, PRDX3, PRDX4, NFIL3, ATG101, CNR1, PRDX1, APOH, ESD, SFN, BCHE, TOR1A, HELT, TGFB3, VEGFD, GCG, KCNRG, TOR1B, SOD2, ACADSB, AICDA, RPIA, NFKBIA, PM20D2, MIS18A, UBA3, DRG1, TAF5, VIM, CRLF3, RAB9B* |
| **tan** | **MF** | GO:0004896~cytokine receptor activity | 7 | 0.31 | 0.01 | *IL4R, FZD4, IFNGR1, MPL, IL12B, IL7R, IL13RA2* |
| **tan** | **MF** | GO:0008137~NADH dehydrogenase (ubiquinone) activity | 6 | 0.27 | 0.01 | *NDUFA13, NDUFB8, NDUFS8, NDUFS7, NDUFS3, NDUFV1* |
| **tan** | **MF** | GO:0003755~peptidyl-prolyl cis-trans isomerase activity | 10 | 0.45 | 0.01 | *PPIL1, FKBP7, PIN4, PPIH, PPIB, FKBP4, FKBP3, PPID, FKBP6, PPIC* |
| **tan** | **MF** | GO:0030955~potassiμm ion binding | 4 | 0.18 | 0.01 | *PKM, ADPRH, DRG1, KCNA4* |
| **tan** | **MF** | GO:0005525~GTP binding | 41 | 1.83 | 0.02 | *RAB1A, ARF4, ARF1, SAR1A, SAR1B, ATL1, RASL11A, DIRAS2, GNAI3, ARL1, RAB21, REM1, RASD1, RAB26, MTG1, RAB29, ANXA6, ARL16, TGM2, ARL11, RAB4A, RAB39A, ERAL1, RHOC, EEF2, RHOA, GEM, RAB33A, RAB11B, GNL3, RAB32, EEF1A1, RAP2C, EHD3, RRAGD, RAB35, DRG1, SRPRA, RAB19, RAN, RAB9B* |
| **tan** | **MF** | GO:0051787~misfolded protein binding | 4 | 0.18 | 0.02 | *TOR1A, SDF2L1, DNAJB9, STUB1* |
| **tan** | **MF** | GO:0004784~superoxide dismutase activity | 3 | 0.13 | 0.03 | *SOD2, SOD3, SOD1* |
| **tan** | **MF** | GO:0051920~peroxiredoxin activity | 3 | 0.13 | 0.03 | *PRDX4, PRDX1, PARK7* |
| **tan** | **MF** | GO:0051539~4 iron, 4 sulfur cluster binding | 7 | 0.31 | 0.03 | *NDUFS8, NDUFS7, CIAPIN1, LIAS, NDUFV1, NUBP1, NUBP2* |
| **tan** | **MF** | GO:0032977~membrane insertase activity | 4 | 0.18 | 0.03 | *EMC3, EMC6, EMC7, EMC8* |
| **tan** | **MF** | GO:0042803~protein homodimerization activity | 45 | 2.00 | 0.03 | *ABCD2, HSP90AB1, MSTN, FLT4, NPR3, HSD17B4, LPL, PARK7, PLD6, TXN, PHB2, CLN6, CLCN1, SRM, NFS1, IRAK2, GRPEL2, CRYM, B2M, PDK2, S100A10, CASR, ENDOG, HMGCS1, CAV2, FZD4, SLC11A1, NOG, SERPINF2, ADIPOQ, DAPK3, CIDEA, DNTTIP1, FZD9, NAGA, SLC39A13, SYT6, ADRB3, STK25, PECAM1, FIBIN, GOPC, STUB1, SSBP1, GPD1L* |
| **tan** | **MF** | GO:0051087~chaperone binding | 10 | 0.45 | 0.04 | *BAG2, DNAJB5, BAG3, DNAJB4, GRPEL2, BAG1, DNAJC9, STUB1, CP, GET4* |
| **tan** | **MF** | GO:0097718~disordered domain specific binding | 5 | 0.22 | 0.04 | *PPIL1, HSP90AB1, HSPA2, CALM2, ARPP19* |
| **tan** | **MF** | GO:0019955~cytokine binding | 4 | 0.18 | 0.04 | *FZD4, IFNGR1, NOG, PARK7* |
| **tan** | **MF** | GO:0000166~nucleotide binding | 8 | 0.36 | 0.04 | *NT5C3A, NAXE, POLR2D, VDAC3, CRY1, SUCLG1, HPRT1, TRPV1* |
| **tan** | **MF** | GO:0015288~porin activity | 3 | 0.13 | 0.04 | *VDAC3, VDAC2, VDAC1* |

**Table S3 8个模块中基因的KEGG富集分析结果**

| **Module** | **Term** | **Count** | **%** | ***P*** | **Genes** |
| --- | --- | --- | --- | --- | --- |
| **blue** | oas00100:Steroid biosynthesis | 5 | 0.66 | 0.01 | *SQLE, NSDHL, SC5D, DHCR24, LSS* |
| **blue** | oas00563:Glycosylphosphatidylinositol (GPI)-anchor biosynthesis | 6 | 0.80 | 0.00 | *PIGS, PIGC, PIGO, PIGQ, MPPE1, PIGV* |
| **blue** | oas03460:Fanconi anemia pathway | 8 | 1.06 | 0.00 | *RMI2, SLX4, RAD51, FANCD2, RPA1, FAAP24, HES1, FANCE* |
| **blue** | oas01232:Nucleotide metabolism | 8 | 1.06 | 0.04 | *RRM1, RRM2, ENTPD3, IMPDH2, ENTPD6, AMPD2, TK1, TYMS* |
| **blue** | oas04926:Relaxin signaling pathway | 11 | 1.46 | 0.02 | *CREB3, JUN, EDN1, GNGT2, EDNRB, GNG4, SHC1, COL4A4, MMP2, COL4A6, ADCY7* |
| **blue** | oas04114:Oocyte meiosis | 12 | 1.59 | 0.01 | *CDC20, SGO1, ANAPC13, YWHAB, PPP2R5D, FBXO5, BUB1, ADCY7, YWHAG, MAD2L1, AURKA, RBX1* |
| **blue** | oas04814:Motor proteins | 14 | 1.86 | 0.03 | *MYH7B, DNAH10, DYNLT1, DCTN2, TPM4, DCTN1, TPM3, KIF14, KIF11, KIF15, KIF18B, MYO5C, KIF2C, KIF1A* |
| **blue** | oas05205:Proteoglycans in cancer | 15 | 1.99 | 0.03 | *DDX5, FZD2, MMP2, FZD7, IGF2, WNT6, TFAP4, PLAU, MYC, PDCD4, GPC3, PTPN6, RAC1, EZR, TLR4* |
| **blue** | oas04141:Protein processing in endoplasmic reticulμm | 16 | 2.12 | 0.00 | *UBXN2A, DNAJC5B, WFS1, AMFR, EIF2AK1, DERL1, HERPUD1, RBX1, SEC61A1, ERLEC1, HYOU1, BAK1, RNF185, SEC23B, CRYAB, TXNDC5* |
| **blue** | oas04110:Cell cycle | 27 | 3.58 | 0.00 | *ANAPC13, YWHAB, BUB1B, CDC20, CDC45, MYC, RAD21, E2F2, E2F4, FBXO5, SKP2, BUB1, YWHAG, PPP2R5D, NDC80, TICRR, RBX1, SGO1, CCNA1, WEE1, TFDP1, MCM3, MCM4, MCM5, TRIP13, MCM2, MAD2L1* |
| **blue** | oas05200:Pathways in cancer | 34 | 4.51 | 0.01 | *LAMA5, PDGFA, GLI1, ADCY7, WNT6, EDNRB, GNGT2, TERT, GNG4, MYC, E2F2, HES1, RAC1, BAK1, SKP2, JAG2, JUN, EDN1, EGLN3, FZD2, TPM3, TXNRD1, MMP2, FZD7, PTCH2, IGF2, RBX1, HEYL, CCNA1, RAD51, TRAF4, COL4A4, COL4A6, BIRC5* |
| **brown** | oas00533:Glycosaminoglycan biosynthesis - keratan sulfate | 9 | 0.14 | 0.01 | *FUT8, B4GALT1, B3GNT2, CHST4, ST3GAL1, CHST2, B4GALT4, ST3GAL2, ST3GAL3* |
| **brown** | oas00532:Glycosaminoglycan biosynthesis - chondroitin sulfate / dermatan sulfate | 13 | 0.21 | 0.00 | *CSGALNACT1, CHST7, CSGALNACT2, XYLT2, XYLT1, CHSY1, DSEL, DSE, UST, CHST15, CHSY3, B4GALT7, CHST3* |
| **brown** | oas00515:Mannose type O-glycan biosynthesis | 13 | 0.21 | 0.01 | *POMT2, B3GALNT2, B4GALT1, B3GAT2, POMT1, B3GAT1, POMGNT2, FUT4, POMK, LARGE1, LARGE2, FKTN, ST3GAL3* |
| **brown** | oas00563:Glycosylphosphatidylinositol (GPI)-anchor biosynthesis | 13 | 0.21 | 0.03 | *PIGU, PGAP1, PIGN, PIGZ, PIGW, PIGX, PIGB, PIGA, PIGK, PIGL, PIGG, GPLD1, PIGH* |
| **brown** | oas04392:Hippo signaling pathway - multiple species | 14 | 0.22 | 0.02 | *TEAD4, YAP1, WWTR1, WWC1, STK3, MOB1B, LATS1, PAK1, FRMD6, DCHS2, RASSF6, FAT4, NF2, TEAD1* |
| **brown** | oas00534:Glycosaminoglycan biosynthesis - heparan sulfate / heparin | 15 | 0.24 | 0.00 | *GLCE, XYLT2, XYLT1, HS3ST5, HS6ST1, HS6ST2, HS6ST3, HS2ST1, HS3ST2, NDST2, EXT1, EXT2, NDST1, EXTL3, B4GALT7* |
| **brown** | oas04960:Aldosterone-regulated sodiμm reabsorption | 18 | 0.29 | 0.02 | *PRKCB, IRS1, PDPK1, INSR, PIK3CD, NEDD4L, PRKCA, PIK3R1, IGF1, PIK3CB, ATP1B1, NR3C2, SCNN1G, SCNN1B, SCNN1A, MAPK1, KRAS, KCNJ1* |
| **brown** | oas04710:Circadian rhythm | 19 | 0.30 | 0.01 | *PRKAB2, PRKAA2, FBXW11, BHLHE41, CUL1, PRKAG2, RORA, NR1D2, CSNK1D, RORB, PRKAG3, PER2, PER3, CREB1, BHLHE40, CRY2, FBXL3, BTRC, CLOCK* |
| **brown** | oas00512:Mucin type O-glycan biosynthesis | 20 | 0.32 | 0.00 | *GALNT7, GALNT12, GALNT11, GALNT5, GALNT14, ST6GALNAC2, GALNT13, GALNT2, GALNT1, GALNT18, GCNT1, C1GALT1, GALNT10, GCNT3, GCNT4, ST6GALNAC3, ST3GAL1, ST3GAL2, B4GALT5, GALNT8* |
| **brown** | oas03022:Basal transcription factors | 21 | 0.33 | 0.03 | *GTF2A1L, GTF2A1, TBP, TAF15, CCNH, GTF2H1, GTF2H3, GTF2F2, GTF2E1, TAF5L, GTF2E2, ERCC3, TAF8, TAF4B, TBPL1, GTF2IRD1, MNAT1, TAF4, TAF3, TAF2, TAF1* |
| **brown** | oas00514:Other types of O-glycan biosynthesis | 23 | 0.37 | 0.00 | *GALNT7, GALNT12, POMT2, GALNT11, GALNT5, ST6GAL1, GALNT14, COLGALT2, POGLUT1, ST6GAL2, B4GALT1, GALNT13, GALNT2, GALNT1, GALNT18, POMT1, C1GALT1, GXYLT1, B3GLCT, GALNT10, OGT, ST3GAL3, GALNT8* |
| **brown** | oas03440:Homologous recombination | 24 | 0.38 | 0.00 | *TOP3B, BARD1, BLM, XRCC2, XRCC3, BRCC3, BRCA1, BRCA2, PALB2, RAD54B, RAD52, POLD3, RAD51B, BRIP1, RAD51D, RAD50, RAD51C, UIMC1, RBBP8, RAD54L, ATM, TOPBP1, NBN, SYCP3* |
| **brown** | oas03460:Fanconi anemia pathway | 25 | 0.40 | 0.00 | *BLM, WDR48, BRCA1, BRCA2, BRIP1, USP1, REV3L, POLI, PMS2, POLK, POLH, FANCI, TOP3B, FANCM, FANCL, REV1, FANCC, MLH1, PALB2, FANCG, RAD51C, UBE2T, FAN1, TELO2, ATR* |
| **brown** | oas04370:VEGF signaling pathway | 26 | 0.41 | 0.01 | *SRC, PXN, PIK3CD, PIK3R1, PIK3CB, CASP9, PPP3CA, PPP3CB, PPP3R1, PPP3CC, AKT3, KDR, PLCG2, RAC2, AKT1, MAPK1, PLCG1, PLA2G4F, PRKCB, NFATC2, PRKCA, MAPK14, MAPK11, MAPKAPK3, KRAS, RAF1* |
| **brown** | oas04927:Cortisol synthesis and secretion | 26 | 0.41 | 0.03 | *SCARB1, ATF2, ITPR1, ITPR2, ADCY3, CACNA1D, ADCY2, ITPR3, CACNA1C, ADCY8, MC2R, CREB3L1, CREB3L2, PDE8A, PRKACB, PBX1, NR5A1, ADCY9, CREB1, PLCB4, STAR, ORAI1, PLCB1, PLCB2, KCNK2, CREB5* |
| **brown** | oas04929:GnRH secretion | 28 | 0.44 | 0.00 | *ITPR1, PIK3CD, ITPR2, ARRB1, CACNA1D, ITPR3, CACNA1C, PIK3R1, PIK3CB, GPER1, AKT3, AKT1, MAPK1, KCNN2, KCNN3, KCNJ3, GABBR2, TRPC5, TRPC4, PRKCB, TRPC1, PRKCA, ESR2, PLCB4, KRAS, RAF1, PLCB1, PLCB2* |
| **brown** | oas04730:Long-term depression | 29 | 0.46 | 0.00 | *GRIA1, GNAZ, GRIA2, ITPR1, ITPR2, ITPR3, IGF1R, GNAI2, PPP2CA, GNA13, GNA12, PRKG2, MAPK1, NOS1, PRKG1, GRIA3, LYN, GUCY1A2, GRID2, PLA2G4F, PRKCB, BRAF, PRKCA, IGF1, PLCB4, KRAS, RAF1, PLCB1, PLCB2* |
| **brown** | oas04213:Longevity regulating pathway - multiple species | 29 | 0.46 | 0.00 | *PRKAA2, CLPB, IRS1, PIK3CD, PRKAG2, ADCY3, ADCY2, IRS2, PIK3R1, PIK3CB, ADCY8, FOXO3, FOXO1, PRKAG3, IGF1R, AKT3, AKT1, PRKACB, ATG5, HSPA8, PRKAB2, INSR, IGF1, SIRT1, MTOR, ADCY9, RPS6KB1, CAT, KRAS* |
| **brown** | oas04330:Notch signaling pathway | 30 | 0.48 | 0.00 | *NOTCH2, NOTCH1, CTBP2, MAML2, MAML1, DTX1, DTX2, PSEN1, DTX4, RBPJ, DLL1, ATXN1, ATXN1L, DVL1, HEY2, DVL3, EP300, CIR1, TLE4, SPEN, TLE3, JAG1, DTX3L, NCOR2, KAT2B, ADAM17, ITCH, SNW1, NμmB, MAML3* |
| **brown** | oas05221:Acute myeloid leukemia | 30 | 0.48 | 0.00 | *CSF1R, BCL2A1, FLT3, LEF1, PIK3CD, PIK3R1, PIK3CB, IKBKB, AKT3, AKT1, MAPK1, TCF7L2, TCF7L1, ZBTB16, STAT3, BRAF, NFKB1, MTOR, PML, RUNX1, PER2, RPS6KB1, KIT, RARA, GRB2, KRAS, RAF1, SOS1, SOS2, PPARD* |
| **brown** | oas05213:Endometrial cancer | 31 | 0.49 | 0.00 | *GSK3B, LEF1, PTEN, PIK3CD, PIK3R1, PIK3CB, FOXO3, EGFR, CASP9, AKT3, CTNNA1, AKT1, MAPK1, CTNNA3, POLK, APC2, TCF7L2, TCF7L1, PDPK1, AXIN1, BRAF, AXIN2, MLH1, DDB2, APC, CTNNB1, GRB2, KRAS, RAF1, SOS1, SOS2* |
| **brown** | oas05217:Basal cell carcinoma | 31 | 0.49 | 0.00 | *GSK3B, WNT2B, LEF1, GLI3, GLI2, SUFU, DVL1, DVL3, POLK, WNT2, WNT3, WNT4, APC2, FZD1, TCF7L2, TCF7L1, FZD3, FZD5, WNT3A, PTCH1, FZD6, WNT5A, AXIN1, WNT7A, WNT9A, AXIN2, DDB2, BMP4, SMO, APC, CTNNB1* |
| **brown** | oas05031:Amphetamine addiction | 31 | 0.49 | 0.00 | *GRIA1, ATF2, GRIA2, DDC, CAMK2D, CACNA1D, CALML4, CACNA1C, PPP1CB, PPP3CA, PPP3CB, PPP3R1, GRIN2A, PPP3CC, CREB3L1, CREB3L2, CAMK2G, SLC18A1, PRKACB, GRIA3, SLC18A2, GRIA4, PRKCB, PRKCA, PDYN, SIRT1, GRIN1, GRIN3B, CREB1, CAMK4, CREB5* |
| **brown** | oas05100:Bacterial invasion of epithelial cells | 31 | 0.49 | 0.02 | *SHC4, SHC3, ARHGEF26, SRC, PXN, CLTC, ARPC5L, PIK3CD, PIK3R1, WASL, PIK3CB, CBL, ACTR3B, CTNNA1, CTNNA3, WASF2, ACTR3, ACTR2, CAV1, GAB1, FN1, DNM1, ARHGAP10, CTTN, ELMO1, ELMO2, CTNNB1, MET, DOCK1, VCL, BCAR1* |
| **brown** | oas04664:Fc epsilon RI signaling pathway | 32 | 0.51 | 0.00 | *PIK3CD, PIK3R1, PIK3CB, MAPK9, MAPK8, ALOX5, INPP5D, AKT3, PLCG2, RAC2, AKT1, MAPK1, FYN, PLCG1, VAV3, LYN, MAP2K4, PLA2G4F, SYK, PDPK1, PRKCA, GAB2, MAPK14, IL4, MAPK11, BTK, GRB2, KRAS, LCP2, RAF1, SOS1, SOS2* |
| **brown** | oas05218:Melanoma | 32 | 0.51 | 0.00 | *RB1, PDGFB, PTEN, PIK3CD, PIK3R1, PIK3CB, FGF2, EGFR, IGF1R, FGF8, FGF9, PDGFD, PDGFC, AKT3, AKT1, MAPK1, E2F3, POLK, FGF23, PDGFRB, PDGFRA, HGF, MITF, BRAF, IGF1, DDB2, CDK6, FGF18, KRAS, RAF1, MET, FGF10* |
| **brown** | oas05211:Renal cell carcinoma | 32 | 0.51 | 0.00 | *CUL2, PDGFB, PIK3CD, PIK3R1, PIK3CB, ETS1, RAP1B, FLCN, PAK1, RAP1A, AKT3, AKT1, MAPK1, PAK6, EP300, PAK3, PAK2, PAK5, EGLN1, ARNT2, TGFB2, HGF, GAB1, ARNT, BRAF, RAPGEF1, GRB2, KRAS, RAF1, SOS1, MET, SOS2* |
| **brown** | oas04727:GABAergic synapse | 32 | 0.51 | 0.04 | *GABRB2, SRC, ADCY3, CACNA1D, ADCY2, ABAT, CACNA1C, ADCY8, SLC6A1, GPHN, GLS, TRAK2, GNAI2, SLC38A2, PRKACB, NSF, GABRA2, GABRA1, GABBR2, SLC38A1, SLC12A5, PRKCB, GABRA4, PRKCA, GNG12, GABRG2, GABRG1, ADCY9, GNB1, GNB4, GNB3, GNB5* |
| **brown** | oas04720:Long-term potentiation | 33 | 0.52 | 0.00 | *GRIA1, GRIA2, CAMK2D, ITPR1, ITPR2, ITPR3, CALML4, CACNA1C, ADCY8, RAP1B, PPP1CB, RPS6KA3, PPP3CA, PPP3CB, PPP3R1, GRIN2A, RAP1A, PPP3CC, RPS6KA2, MAPK1, EP300, CAMK2G, PRKACB, PRKCB, BRAF, PRKCA, GRIN1, PLCB4, CAMK4, KRAS, RAF1, PLCB1, PLCB2* |
| **brown** | oas05220:Chronic myeloid leukemia | 33 | 0.52 | 0.01 | *RB1, SHC4, CDKN1B, SHC3, CTBP2, PIK3CD, PIK3R1, PIK3CB, CBL, IKBKB, AKT3, ABL1, AKT1, MAPK1, E2F3, POLK, TGFB2, SMAD4, SMAD3, BRAF, GAB2, NFKB1, TGFBR1, TGFBR2, RUNX1, DDB2, CDK6, GRB2, KRAS, RAF1, SOS1, SOS2, BCL2L1* |
| **brown** | oas03018:RNA degradation | 33 | 0.52 | 0.02 | *ZCCHC7, DDX6, BTG1, DIS3L, PARN, ENO2, EDC3, EXOSC7, EXOSC9, EXOSC8, DHX36, EXOSC3, DCPS, PNPT1, CNOT6L, DIS3, CNOT10, LSM3, PATL1, CNOT4, CNOT6, PFKL, PAN3, CNOT1, PNLDC1, CNOT2, XRN2, C1D, DCP1A, CNOT8, CNOT9, DCP2, DCP1B* |
| **brown** | oas04917:Prolactin signaling pathway | 33 | 0.52 | 0.02 | *SHC4, GSK3B, GALT, SHC3, SRC, SLC2A2, PIK3CD, PIK3R1, PIK3CB, TNFRSF11A, FOXO3, MAPK9, MAPK8, CCND2, AKT3, AKT1, MAPK1, SOCS6, JAK2, SOCS4, SOCS5, STAT1, STAT3, MAPK14, ESR1, NFKB1, ESR2, MAPK11, GRB2, KRAS, RAF1, SOS1, SOS2* |
| **brown** | oas05412:Arrhythmogenic right ventricular cardiomyopathy | 34 | 0.54 | 0.00 | *ITGB5, LAMA2, ITGB4, ITGA2B, LEF1, ATP2A2, CACNA1D, CACNA1C, SLC8A1, SGCD, CDH2, CTNNA1, ITGB8, ITGAV, CTNNA3, ITGB6, CACNG4, TCF7L2, TCF7L1, ITGA4, ITGA3, CACNA2D1, ITGA2, ITGA1, CACNA2D3, CACNB1, CACNB2, CACNB4, ITGA11, PKP2, ITGA8, CTNNB1, ITGA6, ITGA9* |
| **brown** | oas05410:Hypertrophic cardiomyopathy | 34 | 0.54 | 0.03 | *PRKAA2, ITGB5, LAMA2, ITGB4, ITGA2B, ATP2A2, PRKAG2, CACNA1D, CACNA1C, PRKAG3, SLC8A1, SGCD, ITGB8, ITGAV, ITGB6, CACNG4, MYBPC3, TGFB2, PRKAB2, ITGA4, ITGA3, CACNA2D1, ITGA2, ITGA1, CACNA2D3, IGF1, CACNB1, CACNB2, IL6, CACNB4, ITGA11, ITGA8, ITGA6, ITGA9* |
| **brown** | oas04340:Hedgehog signaling pathway | 35 | 0.56 | 0.00 | *GSK3B, MGRN1, CUL3, CUL1, ARRB1, LRP2, EFCAB7, GLI3, GLI2, SCUBE2, HHAT, GRK2, CCND2, SUFU, BOC, BTRC, PRKACB, CDON, EVC2, CSNK1G3, SMURF2, FBXW11, SMURF1, CSNK1A1, PTCH1, IQCE, CSNK1D, DISP1, EVC, SMO, GPR161, BCL2, SPOPL, GAS1, CSNK1G1* |
| **brown** | oas05223:Non-small cell lung cancer | 36 | 0.57 | 0.00 | *RB1, ALK, PIK3CD, PIK3R1, PIK3CB, FOXO3, FHIT, EGFR, CASP9, KIF5C, KIF5B, RASSF5, AKT3, PLCG2, AKT1, MAPK1, E2F3, PLCG1, POLK, RXRG, PRKCB, PDPK1, HGF, STAT3, BRAF, PRKCA, DDB2, EML4, CDK6, RARB, GRB2, KRAS, RAF1, SOS1, MET, SOS2* |
| **brown** | oas04662:B cell receptor signaling pathway | 36 | 0.57 | 0.00 | *BLK, GSK3B, DAPP1, PIK3CD, BCL10, PIK3R1, PIK3CB, MALT1, IKBKB, PPP3CA, PPP3CB, PPP3R1, PPP3CC, INPP5D, AKT3, PLCG2, RAC2, BLNK, AKT1, MAPK1, VAV3, LYN, SYK, PRKCB, NFATC3, NFATC2, NFKB1, BANK1, BTK, GRB2, KRAS, RAF1, PIK3AP1, SOS1, SOS2, CARD11* |
| **brown** | oas05032:Morphine addiction | 36 | 0.57 | 0.01 | *GABRB2, PDE1A, PDE3B, ADCY3, ARRB1, ADCY2, ADCY8, GNAI2, PDE11A, GRK2, GRK5, GRK4, GRK6, PDE4B, PDE8A, PRKACB, KCNJ3, GABRA2, GABRA1, GABBR2, PRKCB, PDE4D, GABRA4, PRKCA, GNG12, GABRG2, GABRG1, ADCY9, PDE10A, GNB1, PDE3A, GNB4, GNB3, GNB5, PDE7B, PDE7A* |
| **brown** | oas04658:Th1 and Th2 cell differentiation | 36 | 0.57 | 0.01 | *NOTCH2, NOTCH1, MAML2, MAML1, GATA3, CD3E, IL2RG, RBPJ, DLL1, IKBKB, PPP3CA, MAPK9, PPP3CB, PPP3R1, MAPK8, PPP3CC, STAT4, MAPK1, STAT6, PLCG1, JAK2, JAK1, JAG1, STAT1, NFATC3, NFATC2, MAPK14, NFKB1, IL4, MAPK11, CD4, MAF, IL2RB, PRKCQ, CD247, MAML3* |
| **brown** | oas05214:Glioma | 38 | 0.60 | 0.00 | *RB1, SHC4, CAMK2D, SHC3, PDGFB, PTEN, PIK3CD, CALML4, PIK3R1, PIK3CB, EGFR, IGF1R, AKT3, PLCG2, AKT1, MAPK1, E2F3, PLCG1, POLK, CAMK2G, PDGFRB, PDGFRA, CAMK1D, PRKCB, BRAF, PRKCA, IGF1, MTOR, DDB2, CDK6, CAMK4, GRB2, KRAS, CAMK1, RAF1, SOS1, SOS2, CAMK1G* |
| **brown** | oas04540:Gap junction | 38 | 0.60 | 0.00 | *SRC, ITPR1, LPAR1, PDGFB, ITPR2, ADCY3, ADCY2, ITPR3, HTR2A, ADCY8, EGFR, GNAI2, PDGFD, TUBB1, PDGFC, PRKG2, MAPK1, PRKACB, MAP2K5, PRKG1, PDGFRB, MAP3K2, GUCY1A2, PDGFRA, PRKCB, PRKCA, CSNK1D, TJP1, ADCY9, PLCB4, GRB2, KRAS, RAF1, SOS1, PLCB1, SOS2, PLCB2, TUBA8* |
| **brown** | oas04912:GnRH signaling pathway | 39 | 0.62 | 0.00 | *CAMK2D, SRC, ITPR1, ITPR2, ADCY3, CACNA1D, ADCY2, ITPR3, CALML4, CACNA1C, ADCY8, PLD1, EGFR, MAPK9, MAPK8, MAPK1, PTK2B, CAMK2G, PRKACB, MAP3K4, MAP3K2, MAP2K4, MAP3K3, PLA2G4F, MAP3K1, PRKCB, PRKCA, MAPK14, MAPK11, ADCY9, PLCB4, GRB2, KRAS, RAF1, SOS1, PLCB1, SOS2, PLCB2, HBEGF* |
| **brown** | oas05212:Pancreatic cancer | 40 | 0.63 | 0.00 | *RB1, RALA, RALB, PIK3CD, PIK3R1, PIK3CB, BRCA2, PLD1, EGFR, IKBKB, CASP9, MAPK9, MAPK8, AKT3, RAC2, AKT1, MAPK1, E2F3, POLK, RALGDS, JAK1, SMAD2, RALBP1, TGFB2, SMAD4, SMAD3, STAT1, STAT3, BRAF, NFKB1, MTOR, TGFBR1, TGFBR2, DDB2, CDK6, RPS6KB1, KRAS, RAF1, ARHGEF6, BCL2L1* |
| **brown** | oas04925:Aldosterone synthesis and secretion | 40 | 0.63 | 0.00 | *SCARB1, ATF2, CAMK2D, DAGLA, KCNK9, ITPR1, ITPR2, ADCY3, CACNA1D, ADCY2, ITPR3, CALML4, CACNA1C, ADCY8, MC2R, CREB3L1, CREB3L2, CAMK2G, PRKACB, CAMK1D, PRKCB, PRKCE, ATP2B2, PRKCA, ATP2B1, ATP1B1, ADCY9, CREB1, PLCB4, STAR, PRKD3, CAMK4, ORAI1, PRKD1, CAMK1, DAGLB, PLCB1, PLCB2, CAMK1G, CREB5* |
| **brown** | oas04512:ECM-receptor interaction | 41 | 0.65 | 0.00 | *SDC4, ITGB5, LAMA2, ITGB4, LAMC3, LAMA4, ITGA2B, LAMA3, TNC, LAMC2, LAMC1, THBS1, RELN, TNN, SV2A, ITGB8, TNR, ITGAV, ITGB6, VWF, LAMB3, ITGA4, ITGA3, ITGA2, ITGA1, FN1, LAMB1, NPNT, COL2A1, FRAS1, COL6A2, COL4A3, ITGA11, COL9A1, ITGA8, COL4A5, COL6A6, ITGA6, FREM2, CD44, ITGA9* |
| **brown** | oas04914:Progesterone-mediated oocyte maturation | 41 | 0.65 | 0.00 | *PDE3B, PIK3CD, ADCY3, ADCY2, PIK3R1, PIK3CB, ADCY8, ANAPC10, IGF1R, GNAI2, RPS6KA3, MAPK9, STK10, MAPK8, CDC23, RPS6KA2, AKT3, CDC27, AKT1, MAPK1, PRKACB, ANAPC7, PLK1, BRAF, IGF1, MAPK14, MAPK11, CPEB1, ADCY9, CDC16, SPDYA, ANAPC4, KRAS, PGR, ANAPC5, RAF1, CPEB3, CPEB2, MAD1L1, ANAPC1, CPEB4* |
| **brown** | oas04666:Fc gamma R-mediated phagocytosis | 41 | 0.65 | 0.00 | *ASAP3, ARPC5L, PIK3CD, ASAP1, ASAP2, PIK3R1, PIK3CB, PLD1, PLA2G6, ACTR3B, PAK1, INPP5D, AKT3, PLCG2, RAC2, AKT1, PIP5K1A, MAPK1, PIP5K1B, PLCG1, WASF2, WASF3, VAV3, LYN, ACTR3, ACTR2, PLA2G4F, SYK, MYO10, PRKCB, PRKCE, PRKCA, GAB2, HCK, PTPRC, RPS6KB1, BIN1, AMPH, PLPP3, RAF1, DOCK1* |
| **brown** | oas04713:Circadian entrainment | 41 | 0.65 | 0.00 | *GRIA1, GRIA2, ADCYAP1R1, CAMK2D, ITPR1, ADCY3, CACNA1D, ADCY2, ITPR3, CALML4, CACNA1C, ADCY8, RYR3, GNAI2, GRIN2A, RPS6KA5, PRKG2, MAPK1, NOS1, CAMK2G, PRKACB, PRKG1, GRIA3, GRIA4, KCNJ3, GUCY1A2, PRKCB, PRKCA, GNG12, GRIN1, PER2, PER3, ADCY9, CREB1, PLCB4, GNB1, GNB4, GNB3, GNB5, PLCB1, PLCB2* |
| **brown** | oas04064:NF-kappa B signaling pathway | 41 | 0.65 | 0.01 | *BCL2A1, LY96, BCL10, TNFRSF11A, MALT1, TNFSF13B, IKBKB, PLCG2, BLNK, RIPK1, PLCG1, ERC1, PIAS4, LYN, SYK, CSNK2A1, PARP1, PRKCB, CSNK2A2, IRAK4, TRAF1, CFLAR, NFKB1, EDAR, CD40LG, CXCL12, TRAF3, TRAF6, TRAF5, BCL2, BTK, TAB3, TAB2, PRKCQ, ATM, TAB1, MAP3K14, MYD88, CARD11, BIRC2, BCL2L1* |
| **brown** | oas04625:C-type lectin receptor signaling pathway | 41 | 0.65 | 0.01 | *SRC, ITPR1, PIK3CD, ITPR2, CBLB, ITPR3, CALML4, BCL10, PIK3R1, PIK3CB, MALT1, IKBKB, PPP3CA, MAPK9, PAK1, PPP3CB, PPP3R1, MAPK8, CASP8, PPP3CC, AKT3, PLCG2, AKT1, MAPK1, IKBKE, IL10, ARHGEF12, SYK, STAT1, NFATC3, NFATC2, RRAS2, MAPK14, NFKB1, MAPK11, IL6, MRAS, KRAS, IL17D, RAF1, MAP3K14* |
| **brown** | oas04750:Inflammatory mediator regulation of TRP channels | 41 | 0.65 | 0.01 | *PTGER4, CAMK2D, SRC, ITPR1, PIK3CD, ITPR2, ADCY3, ADCY2, ITPR3, CALML4, HTR2A, PIK3R1, PIK3CB, ADCY8, PLA2G6, KNG1, PPP1CB, MAPK9, MAPK8, HRH1, PLCG2, TRPM8, PLCG1, CAMK2G, PRKACB, ASIC1, PLA2G4F, PRKCH, TRPA1, PRKCB, PRKCE, PRKCA, IGF1, NGF, MAPK14, MAPK11, ADCY9, PLCB4, PRKCQ, PLCB1, PLCB2* |
| **brown** | oas04933:AGE-RAGE signaling pathway in diabetic complications | 42 | 0.67 | 0.00 | *CDKN1B, PIK3CD, PIK3R1, PIK3CB, PRKCZ, FOXO1, MAPK9, MAPK8, CASP3, AKT3, PLCG2, AKT1, MAPK1, PLCE1, PLCG1, JAK2, SMAD2, TGFB2, SMAD4, SMAD3, PRKCB, STAT1, PRKCE, STAT3, FN1, VEGFC, PRKCA, MAPK14, NFKB1, TGFBR1, TGFBR2, MAPK11, IL6, PLCB4, COL4A3, BCL2, COL4A5, KRAS, PLCB1, PLCB2, PLCD4, NOX1* |
| **brown** | oas04922:Glucagon signaling pathway | 42 | 0.67 | 0.01 | *ATF2, PYGB, CAMK2D, PRKAA2, PDE3B, ITPR1, SLC2A2, ITPR2, PRKAG2, ADCY2, ITPR3, CALML4, FOXO1, PRKAG3, ACACA, PPP3CA, PPP3CB, PPP3R1, PPP3CC, CREB3L1, CREB3L2, AKT3, AKT1, EP300, PPARGC1A, CAMK2G, PRKACB, PRKAB2, PPP4R3A, PHKB, PHKA1, PPP4R3B, PHKA2, SIRT1, PFKL, CREB1, PLCB4, SIK2, PPARA, PLCB1, PLCB2, CREB5* |
| **brown** | oas04659:Th17 cell differentiation | 42 | 0.67 | 0.01 | *RORA, GATA3, AHR, CD3E, IL2RG, IKBKB, PPP3CA, MAPK9, PPP3CB, PPP3R1, MAPK8, PPP3CC, MAPK1, STAT6, PLCG1, JAK2, IL6R, RXRG, JAK1, SMAD2, SMAD4, SMAD3, STAT1, STAT3, NFATC3, NFATC2, MAPK14, NFKB1, MTOR, TGFBR1, TGFBR2, RUNX1, IL4, MAPK11, IL6, CD4, IRF4, IL2RB, RARA, PRKCQ, CD247, IL6ST* |
| **brown** | oas04670:Leukocyte transendothelial migration | 42 | 0.67 | 0.03 | *ITK, ROCK1, NCF2, ROCK2, PXN, CTNND1, ITGB2, NCF4, CXCR4, PIK3CD, CD99L2, THY1, PIK3R1, PIK3CB, ARHGAP5, CLDN1, ARHGAP35, GNAI2, RAP1B, RAP1A, RASSF5, PLCG2, RAC2, CTNNA1, PTK2B, CTNNA3, PLCG1, JAM2, VAV3, ITGA4, PRKCB, ACTN1, RHOH, PRKCA, MAPK14, AFDN, MAPK11, CXCL12, CTNNB1, CLDN16, VCL, BCAR1* |
| **brown** | oas05235:PD-L1 expression and PD-1 checkpoint pathway in cancer | 43 | 0.68 | 0.00 | *ALK, CD274, PTEN, PIK3CD, PIK3R1, PIK3CB, CD3E, RASGRP1, EGFR, IKBKB, PPP3CA, PPP3CB, PPP3R1, PPP3CC, AKT3, AKT1, MAPK1, PLCG1, JAK2, JAK1, MAP3K3, CSNK2A1, STAT1, CSNK2A2, STAT3, NFATC3, NFATC2, MAPK14, NFKB1, MTOR, BATF, EML4, MAPK11, CD4, RPS6KB1, TRAF6, CD28, KRAS, PRKCQ, CD247, RAF1, MYD88, TLR2* |
| **brown** | oas04211:Longevity regulating pathway | 43 | 0.68 | 0.00 | *ATF2, PRKAA2, IRS1, EHMT1, PIK3CD, PRKAG2, ADCY3, ADCY2, IRS2, PIK3R1, PIK3CB, ADCY8, FOXO3, FOXO1, PRKAG3, IGF1R, STK11, SESN1, CREB3L1, CREB3L2, AKT3, AKT1, PPARGC1A, PRKACB, ATG5, APPL1, PRKAB2, INSR, TSC2, TSC1, IGF1, SIRT1, ATG13, NFKB1, MTOR, ADCY9, CREB1, RPS6KB1, RHEB, CAMK4, CAT, KRAS, CREB5* |
| **brown** | oas01522:Endocrine resistance | 43 | 0.68 | 0.00 | *RB1, NOTCH2, SHC4, CDKN1B, NOTCH1, SHC3, SRC, PIK3CD, ADCY3, ADCY2, PIK3R1, PIK3CB, ADCY8, DLL1, EGFR, IGF1R, MAPK9, MAPK8, GPER1, AKT3, AKT1, MAPK1, E2F3, PRKACB, JAG1, NCOA3, BRAF, IGF1, MAPK14, ESR1, MTOR, ESR2, MAPK11, NCOR1, ADCY9, RPS6KB1, BCL2, GRB2, KRAS, RAF1, SOS1, SOS2, HBEGF* |
| **brown** | oas00562:Inositol phosphate metabolism | 44 | 0.70 | 0.00 | *IPPK, PI4K2B, MTMR2, MTMR3, MTMR14, PTEN, PIK3CD, PIK3C2G, PIK3CB, PIK3C2A, MTMR4, PIK3C2B, MTM1, MTMR7, INPP5B, INPP5A, INPP5F, IMPA2, INPP5D, PLCG2, INPP5J, PIP5K1A, PLCE1, PIP4K2A, PIP5K1B, PIP4K2B, PLCG1, SACM1L, ITPK1, OCRL, SYNJ2, FIG4, INPP4A, ITPKB, PIKFYVE, PLCB4, SYNJ1, PI4KA, PIK3C3, PLCH1, PI4KB, PLCB1, PLCB2, PLCD4* |
| **brown** | oas04916:Melanogenesis | 44 | 0.70 | 0.00 | *GSK3B, CAMK2D, WNT2B, ASIP, LEF1, ADCY3, ADCY2, CALML4, ADCY8, GNAI2, CREB3L1, CREB3L2, DVL1, DVL3, MAPK1, EP300, WNT2, CAMK2G, PRKACB, WNT3, WNT4, FZD1, TCF7L2, TCF7L1, FZD3, FZD5, PRKCB, WNT3A, FZD6, WNT5A, WNT7A, MITF, PRKCA, WNT9A, KITLG, ADCY9, CREB1, PLCB4, KIT, CTNNB1, KRAS, RAF1, PLCB1, PLCB2* |
| **brown** | oas04928:Parathyroid hormone synthesis, secretion and action | 45 | 0.71 | 0.00 | *GCM2, ATF2, ITPR1, LRP5, ITPR2, ADCY3, ARRB1, ADCY2, ITPR3, PTH1R, GATA3, ADCY8, PLD1, EGFR, LRP6, GNAI2, GNA13, AKAP13, MMP24, CREB3L1, CREB3L2, GNA12, PDE4B, MAPK1, FGF23, MEF2D, RXRG, PRKACB, MEF2A, MEF2C, PRKCB, PDE4D, BRAF, PRKCA, RUNX2, ADCY9, CREB1, PLCB4, MMP16, BCL2, RAF1, PLCB1, PLCB2, CREB5, HBEGF* |
| **brown** | oas04725:Cholinergic synapse | 45 | 0.71 | 0.00 | *CHRNA3, CAMK2D, ITPR1, PIK3CD, ITPR2, ADCY3, CACNA1D, ADCY2, ITPR3, CACNA1C, PIK3R1, PIK3CB, ADCY8, PIK3R6, PIK3R5, GNAI2, CREB3L1, CREB3L2, AKT3, AKT1, MAPK1, FYN, JAK2, CAMK2G, PRKACB, KCNJ3, PRKCB, PRKCA, GNG12, ADCY9, CREB1, PLCB4, CAMK4, KCNQ1, GNB1, KCNQ3, BCL2, GNB4, GNB3, KRAS, KCNQ5, GNB5, PLCB1, PLCB2, CREB5* |
| **brown** | oas04668:TNF signaling pathway | 45 | 0.71 | 0.02 | *ATF2, CSF1, PIK3CD, PIK3R1, PIK3CB, IKBKB, MAPK9, BAG4, MAPK8, CASP7, CASP8, RPS6KA5, CASP3, CREB3L1, CREB3L2, AKT3, AKT1, MAPK1, MAP3K8, RIPK1, DNM1L, MAP3K5, MAP2K4, JAG1, IL15, LIF, VEGFC, TRAF1, CFLAR, MAPK14, NFKB1, MAPK11, IL6, ITCH, CREB1, TRAF3, TRAF5, FAS, TAB3, TAB2, TAB1, MAP3K14, IL18R1, BIRC2, CREB5* |
| **brown** | oas04931:Insulin resistance | 46 | 0.73 | 0.00 | *PYGB, GSK3B, SLC27A1, PRKAA2, IRS1, PTEN, SLC2A2, PIK3CD, PRKAG2, IRS2, PIK3R1, PIK3CB, PRKCZ, PTPRF, FOXO1, PRKAG3, IKBKB, PPP1CB, RPS6KA3, MAPK9, MAPK8, CREB3L1, RPS6KA2, CREB3L2, AKT3, AKT1, PPARGC1A, PPARGC1B, PTPN1, PRKAB2, PRKCB, PDPK1, INSR, PRKCE, STAT3, NFKB1, MTOR, IL6, CREB1, TBC1D4, RPS6KB1, PPP1R3C, PRKCQ, PPARA, OGT, CREB5* |
| **brown** | oas04012:ErbB signaling pathway | 47 | 0.75 | 0.00 | *SHC4, GSK3B, CAMK2D, CDKN1B, SHC3, SRC, PIK3CD, CBLB, PIK3R1, PIK3CB, CBL, EGFR, MAPK9, PAK1, MAPK8, ERBB4, AKT3, PLCG2, NCK2, ABL1, AKT1, ABL2, MAPK1, PAK6, PLCG1, PAK3, CAMK2G, PAK2, PAK5, MAP2K4, PRKCB, GAB1, BRAF, PRKCA, NRG2, MTOR, EREG, BTC, NRG3, RPS6KB1, NRG4, GRB2, KRAS, RAF1, SOS1, SOS2, HBEGF* |
| **brown** | oas05222:Small cell lung cancer | 47 | 0.75 | 0.00 | *RB1, CDKN1B, LAMA2, MAX, LAMC3, LAMA4, ITGA2B, LAMA3, PTEN, PIK3CD, LAMC2, PIK3R1, LAMC1, PIK3CB, FHIT, IKBKB, CASP9, CASP3, AKT3, AKT1, ITGAV, E2F3, POLK, RXRG, ZBTB17, NOS2, LAMB3, APAF1, ITGA3, ITGA2, FN1, LAMB1, TRAF1, NFKB1, DDB2, CDK6, CCNE2, TRAF3, TRAF6, COL4A3, TRAF5, BCL2, COL4A5, RARB, ITGA6, BIRC2, BCL2L1* |
| **brown** | oas04380:Osteoclast differentiation | 47 | 0.75 | 0.01 | *CSF1R, CSF1, NCF2, NCF4, PIK3CD, PIK3R1, PIK3CB, TNFRSF11A, IKBKB, PPP3CA, MAPK9, PPP3CB, PPP3R1, MAPK8, PPP3CC, AKT3, PLCG2, BLNK, AKT1, MAPK1, FYN, JAK1, IFNAR2, TGFB2, SYK, STAT1, NFATC2, MITF, GAB2, MAPK14, NFKB1, TGFBR1, FOSL2, TGFBR2, MAPK11, TEC, CREB1, CAMK4, TRAF6, BTK, GRB2, TAB2, LCP2, TAB1, MAP3K14, NOX1, IFNAR1* |
| **brown** | oas05145:Toxoplasmosis | 47 | 0.75 | 0.03 | *CIITA, LAMA2, LAMC3, LAMA4, LAMA3, LY96, LAMC2, LAMC1, PIK3R6, PIK3R5, GNAI2, IKBKB, CASP9, MAPK9, MAPK8, CASP8, ALOX5, CASP3, AKT3, AKT1, MAPK1, JAK2, JAK1, IL10, HSPA8, TGFB2, NOS2, LAMB3, STAT1, PDPK1, IL10RB, STAT3, LAMB1, IRAK4, MAPK14, NFKB1, MAPK11, CD40LG, TRAF6, BCL2, TAB2, ITGA6, TAB1, MYD88, BIRC2, TLR2, BCL2L1* |
| **brown** | oas01521:EGFR tyrosine kinase inhibitor resistance | 48 | 0.76 | 0.00 | *SHC4, GSK3B, SHC3, SRC, PDGFB, PTEN, PIK3CD, PIK3R1, PIK3CB, FOXO3, FGF2, EGFR, IGF1R, BCL2L11, PDGFD, PDGFC, AKT3, KDR, PLCG2, AKT1, MAPK1, PLCG1, JAK2, IL6R, JAK1, PDGFRB, PDGFRA, PRKCB, HGF, STAT3, GAB1, BRAF, PRKCA, NRG2, IGF1, MTOR, IL6, RPS6KB1, BCL2, NF1, GRB2, KRAS, RAF1, SOS1, MET, SOS2, FGFR2, BCL2L1* |
| **brown** | oas05215:Prostate cancer | 48 | 0.76 | 0.00 | *RB1, GSK3B, CDKN1B, LEF1, PDGFB, PTEN, TMPRSS2, PIK3CD, PLAT, PIK3R1, PIK3CB, FOXO1, EGFR, HSP90B1, IGF1R, IKBKB, CASP9, CREB3L1, PDGFD, CREB3L2, PDGFC, AKT3, AKT1, MAPK1, EP300, E2F3, PDGFRB, PDGFRA, TCF7L2, TCF7L1, PDPK1, IL1R2, BRAF, IGF1, NFKB1, MTOR, CREB1, ZEB1, CCNE2, BCL2, CTNNB1, GRB2, KRAS, RAF1, SOS1, SOS2, FGFR2, CREB5* |
| **brown** | oas05210:Colorectal cancer | 48 | 0.76 | 0.00 | *GSK3B, RALA, RALB, LEF1, PIK3CD, PIK3R1, PIK3CB, EGFR, CASP9, MAPK9, MAPK8, BCL2L11, CASP3, AKT3, RAC2, AKT1, MAPK1, POLK, RALGDS, APPL1, APC2, SMAD2, TCF7L2, TCF7L1, TGFB2, SMAD4, SMAD3, AXIN1, BRAF, AXIN2, MLH1, MTOR, TGFBR1, TGFBR2, EREG, DDB2, MSH6, APC, RPS6KB1, MSH2, MSH3, BCL2, CTNNB1, GRB2, KRAS, RAF1, SOS1, SOS2* |
| **brown** | oas05231:Choline metabolism in cancer | 49 | 0.78 | 0.00 | *SLC44A5, SLC44A3, DGKE, DGKD, SLC44A1, DGKB, SLC44A2, PIK3CD, PIK3CB, AKT3, RAC2, AKT1, PDGFRB, PDGFRA, PLA2G4F, PCYT1A, PDPK1, PRKCB, TSC2, TSC1, PRKCA, GPCPD1, SOS1, PLPP3, RAF1, SOS2, DGKI, DGKH, SLC22A3, PDGFB, PIK3R1, PLD1, EGFR, MAPK9, MAPK8, PDGFD, PDGFC, PIP5K1A, MAPK1, PIP5K1B, PLCG1, WASF2, RALGDS, WASF3, MTOR, RPS6KB1, RHEB, GRB2, KRAS* |
| **brown** | oas03013:Nucleocytoplasmic transport | 49 | 0.78 | 0.01 | *AHCTF1, IPO11, NUP107, CSE1L, GLE1, PNN, IPO8, XPO1, IPO9, SμmO1, XPO4, XPO6, KPNA4, TNPO1, KPNA3, TNPO3, KPNA1, NUP214, UPF2, NDC1, UPF1, NUP133, NCBP1, THOC1, THOC2, RANGAP1, THOC5, SENP2, NUP93, TMEM33, NUP54, NUP98, NUP58, NUP205, SEH1L, NMD3, NUP160, NUP85, TPR, NUP88, NUP43, NUP155, RANBP17, XPO7, UPF3A, SNUPN, XPOT, NUP35, NUP37* |
| **brown** | oas04270:Vascular smooth muscle contraction | 49 | 0.78 | 0.03 | *ADRA1D, CALML4, MYLK3, PPP1CB, EDNRA, PRKACB, PRKG1, GUCY1A2, PLA2G4F, ARHGEF12, PPP1R12A, PRKCH, PLA2G12B, PRKCB, PRKCE, PRKCA, AVPR1A, PLCB4, ADCY9, ADORA2B, KCNMA1, MYH9, PRKCQ, RAF1, PPP1R12B, PLCB1, PLCB2, CALCRL, ROCK1, ROCK2, NPR2, ITPR1, ITPR2, ADCY3, ADCY2, ITPR3, CACNA1D, CACNA1C, ADCY8, PLA2G6, GNA13, CALD1, KCNMB1, GNA12, MAPK1, KCNMB4, MYH11, MYH10, BRAF* |
| **brown** | oas04350:TGF-beta signaling pathway | 50 | 0.79 | 0.00 | *BMPR2, TFRC, ZFYVE9, LRRC32, SIN3A, EP300, PITX2, NEO1, TGIF1, ACVR1, TGIF2, RGMB, TGFBR1, RGMA, TGFBR2, ZFYVE16, NCOR1, RBL1, ROCK1, CUL1, ACVR1B, THBS1, LTBP1, THSD4, PPP2CA, MAPK1, E2F5, SKIL, SMAD2, SMAD1, SMAD4, TGFB2, SMAD3, SMURF2, SMURF1, SMAD9, INHBA, ACVR2B, SMAD5, GDF5, BMP6, ACVR2A, SMAD7, SKI, BMP4, RPS6KB1, ID2, BMPR1B, BMPR1A, FBN1* |
| **brown** | oas04114:Oocyte meiosis | 50 | 0.79 | 0.01 | *CALML4, SMC3, IGF1R, PPP1CB, RPS6KA3, PPP3CA, PPP3CB, SLK, PPP3CC, CDC23, PPP2R5E, RPS6KA2, CDC27, BTRC, PRKACB, ANAPC7, FBXW11, PPP2R5A, PPP2R5C, YWHAZ, ADCY9, CCNE2, ANAPC4, PGR, ANAPC5, MAD1L1, ANAPC1, CAMK2D, ITPR1, CUL1, ITPR2, ADCY3, ADCY2, ITPR3, ADCY8, ANAPC10, PPP2CA, PPP3R1, MAPK1, CAMK2G, PLK1, IGF1, MAPK14, MAPK11, CPEB1, CDC16, SPDYA, CPEB3, CPEB2, CPEB4* |
| **brown** | oas04152:AMPK signaling pathway | 51 | 0.81 | 0.00 | *CAB39L, IRS1, PIK3CD, PRKAG2, IRS2, PIK3CB, ELAVL1, PRKAG3, IGF1R, STK11, EEF2K, PPP2R5E, CREB3L1, CREB3L2, AKT3, AKT1, RAB8A, RAB2A, PRKAB2, PDPK1, STRADA, TSC2, PPP2R5A, TSC1, PPP2R5C, SIRT1, TBC1D1, CREB1, CFTR, CREB5, PFKFB2, PRKAA2, PFKFB3, CAB39, PIK3R1, FOXO3, FOXO1, ACACA, PPP2CA, PPP2R3C, PPARGC1A, INSR, IGF1, PPP2R3A, MTOR, RAB10, PFKL, RAB14, RPS6KB1, RHEB, PPP2R2B* |
| **brown** | oas04926:Relaxin signaling pathway | 53 | 0.84 | 0.00 | *ATF2, PIK3CD, ARRB1, PIK3CB, RXFP1, RXFP2, CREB3L1, CREB3L2, AKT3, AKT1, NOS1, PRKACB, MAP2K4, PRKCA, TGFBR1, TGFBR2, PLCB4, ADCY9, CREB1, COL4A3, COL4A5, SOS1, RAF1, PLCB1, SOS2, PLCB2, CREB5, SHC4, SHC3, SRC, ADCY3, ADCY2, PIK3R1, ADCY8, PRKCZ, EGFR, GNAI2, MAPK9, MAPK8, MAPK1, SMAD2, NOS2, VEGFC, GNG12, MAPK14, NFKB1, MAPK11, GNB1, GNB4, GNB3, GRB2, KRAS, GNB5* |
| **brown** | oas05017:Spinocerebellar ataxia | 54 | 0.86 | 0.04 | *PIK3CD, ATP2A2, RORA, VLDLR, PIK3CB, RBPJ, PSMD6, OPA1, AKT3, AKT1, PSMD1, MAP3K5, TBP, KCND3, PRKCB, PRKCA, WIPI2, PDYN, ATG14, ATG13, ERN1, PSMA5, PSMA6, PLCB4, TBPL1, PIK3C3, PLCB1, PLCB2, GRIA1, GRIA2, BEAN1, PSMD14, ITPR1, ITPR2, ITPR3, AMBRA1, OMA1, PIK3R1, PSMA7, MAPK9, GRIN2A, MAPK8, RELN, ATXN1, ATXN1L, GRIA3, Pμm1, MTOR, GRIN1, GRIN3B, FGF14, ATXN10, MCU, ATG2B* |
| **brown** | oas04520:Adherens junction | 55 | 0.87 | 0.00 | *CTNND1, PTPRM, PTPRJ, WASL, PTPRF, IGF1R, RAC2, EP300, CSNK2A1, ACTN1, HEG1, CSNK2A2, LMO7, TGFBR1, TGFBR2, PARD3, MET, VCL, ROCK1, ROCK2, SRC, LEF1, IQGAP1, NLK, EGFR, RAP1B, RAP1A, PDCD10, KRIT1, CTNNA1, MAPK1, CTNNA3, FYN, WASF2, WASF3, FARP2, PTPN1, TCF7L2, TCF7L1, SMAD4, SMAD3, YES1, SMURF2, SMURF1, INSR, ARHGAP29, SORBS1, TJP1, AFDN, FER, PTPRB, SNAI1, CTNNB1, NECTIN3, NECTIN1* |
| **brown** | oas04071:Sphingolipid signaling pathway | 55 | 0.87 | 0.00 | *PTEN, PIK3CD, PIK3CB, SPTLC2, PPP2R5E, AKT3, RAC2, AKT1, MAP3K5, CERS3, CERS5, CERS6, PDPK1, PRKCB, PRKCE, PPP2R5A, PRKCA, PPP2R5C, GAB2, PLCB4, RAF1, PLCB1, PLCB2, NSMAF, OPRD1, SGMS1, ROCK1, ROCK2, PIK3R1, SGMS2, PLD1, PRKCZ, KNG1, GNAI2, PPP2CA, GNA13, MAPK9, MAPK8, SGPL1, PPP2R3C, GNA12, MAPK1, FYN, BID, PPP2R3A, MAPK14, SGPP2, NFKB1, SGPP1, ACER2, MAPK11, PPP2R2B, BCL2, KRAS, DEGS2* |
| **brown** | oas04724:Glutamatergic synapse | 56 | 0.89 | 0.00 | *GRIK3, GRIK4, GRIK1, GLS, GRM3, PPP3CA, GRM2, PPP3CB, PPP3CC, GRM7, GRM8, DLGAP1, PRKACB, SLC38A1, PLA2G4F, PRKCB, TRPC1, PRKCA, PLCB4, ADCY9, PLCB1, SHANK3, PLCB2, SHANK2, GRIA1, GRIA2, SLC1A1, ITPR1, ITPR2, SLC1A3, ADCY3, ADCY2, ITPR3, CACNA1D, CACNA1C, ADCY8, PLD1, SLC1A7, GNAI2, PPP3R1, GRIN2A, GRK2, MAPK1, SLC38A2, GRIA3, GRIA4, KCNJ3, HOMER1, HOMER2, GNG12, GRIN1, GRIN3B, GNB1, GNB4, GNB3, GNB5* |
| **brown** | oas04935:Growth hormone synthesis, secretion and action | 56 | 0.89 | 0.00 | *GSK3B, ATF2, IRS1, PIK3CD, IRS2, PIK3CB, GHR, CREB3L1, CREB3L2, AKT3, AKT1, EP300, JAK2, PRKACB, MAP2K4, PRKCB, PRKCA, PLCB4, ADCY9, CREB1, SOS1, RAF1, PLCB1, SOS2, PLCB2, CREB5, SHC4, SHC3, ITPR1, ITPR2, ADCY3, ADCY2, ITPR3, CACNA1D, CACNA1C, PIK3R1, ADCY8, GNAI2, MAPK9, MAPK8, PLCG2, MAPK1, PLCG1, MAP3K1, STAT1, STAT3, IGF1, MAPK14, MTOR, MAPK11, GHRH, GRB2, KRAS, GHRL, MRAP2, BCAR1* |
| **brown** | oas04611:Platelet activation | 56 | 0.89 | 0.00 | *SNAP23, ITGA2B, PIK3CD, PIK3CB, ARHGAP35, MYLK3, PPP1CB, AKT3, AKT1, PRKG2, PRKACB, PRKG1, GUCY1A2, PRKCI, PLA2G4F, ARHGEF12, PPP1R12A, SYK, VWF, ITGA2, PLCB4, ADCY9, STIM1, BTK, ORAI1, LCP2, TLN2, PLCB1, PLCB2, ROCK1, ROCK2, SRC, ITPR1, ITPR2, ADCY3, ADCY2, ITPR3, PIK3R1, ADCY8, RASGRP1, PRKCZ, PIK3R6, PIK3R5, GNAI2, RAP1B, GNA13, RAP1A, P2RY1, PLCG2, MAPK1, FYN, LYN, MAPK14, MAPK11, TBXAS1, F2RL3* |
| **brown** | oas04070:Phosphatidylinositol signaling system | 57 | 0.90 | 0.00 | *PI4K2B, DGKE, DGKD, DGKB, MTMR14, PTEN, PIK3CD, PIK3C2G, CALML4, PIK3CB, PIK3C2A, PIK3C2B, PPIP5K1, IMPA2, PPIP5K2, PLCE1, PIP4K2A, PIP4K2B, PRKCB, SACM1L, ITPK1, OCRL, PRKCA, ITPKB, INPP4A, PLCB4, PIK3C3, PLCB1, PLCB2, DGKI, DGKH, IPPK, MTMR2, MTMR3, ITPR1, ITPR2, ITPR3, PIK3R1, MTMR4, MTMR7, MTM1, INPP5B, INPP5A, INPP5F, INPP5D, PLCG2, PIP5K1A, PIP5K1B, PLCG1, IP6K1, IP6K2, SYNJ2, PIKFYVE, SYNJ1, PI4KA, PI4KB, PLCD4* |
| **brown** | oas04660:T cell receptor signaling pathway | 57 | 0.90 | 0.00 | *GSK3B, ITK, PIK3CD, CBLB, PIK3CB, CD3E, IKBKB, PPP3CA, PPP3CB, PPP3CC, GRAP2, AKT3, NCK2, AKT1, CTLA4, MAP3K8, VAV3, IL10, PDPK1, CD8A, PRKCQ, LCP2, SOS1, RAF1, SOS2, CARD11, BCL10, PIK3R1, RASGRP1, MALT1, MAPK9, PPP3R1, MAPK8, PAK1, MAPK1, PAK6, FYN, PLCG1, PAK3, PAK2, PAK5, NFATC3, NFATC2, MAPK14, NFKB1, IL4, MAPK11, DLG1, CD4, CD40LG, TEC, PTPRC, CD28, GRB2, KRAS, CD247, MAP3K14* |
| **brown** | oas04371:Apelin signaling pathway | 57 | 0.90 | 0.00 | *PDE3B, PRKAG2, PLAT, CALML4, PRKAG3, SLC8A1, MYLK3, RYR3, MAP1LC3C, AKT3, AKT1, NOS1, PRKACB, MEF2A, PRKAB2, MEF2C, MEF2B, PRKCE, RRAS2, TGFBR1, MRAS, PLCB4, ADCY9, PIK3C3, RAF1, PLCB1, PLCB2, HDAC4, PRKAA2, ITPR1, ITPR2, ADCY3, ADCY2, ITPR3, NRF1, ADCY8, PIK3R6, PIK3R5, GNAI2, GNA13, MAPK1, PPARGC1A, MEF2D, SMAD2, SMAD4, SMAD3, JAG1, NOS2, GNG12, MTOR, RPS6KB1, CAMK4, GNB1, GNB4, GNB3, KRAS, GNB5* |
| **brown** | oas04910:Insulin signaling pathway | 57 | 0.90 | 0.00 | *GSK3B, PYGB, IRS1, PDE3B, PIK3CD, PRKAG2, CBLB, IRS2, CALML4, PIK3CB, PTPRF, PRKAG3, IKBKB, PPP1CB, HK3, AKT3, AKT1, FLOT2, PRKACB, PRKAB2, PRKCI, PDPK1, TSC2, TSC1, PHKA1, PHKA2, PPP1R3C, RAPGEF1, SOS1, RAF1, SOS2, RHOQ, SHC4, PRKAA2, SHC3, PIK3R1, CBL, PRKCZ, FOXO1, ACACA, MAPK9, MAPK8, INPP5A, MKNK1, MAPK1, PPARGC1A, SOCS4, PTPN1, INSR, PHKB, BRAF, SORBS1, MTOR, RPS6KB1, RHEB, GRB2, KRAS* |
| **brown** | oas05135:Yersinia infection | 57 | 0.90 | 0.00 | *GSK3B, WIPF1, WIPF3, ARPC5L, PIK3CD, WASL, PIK3CB, IKBKB, RPS6KA3, TBK1, RPS6KA2, AKT3, RAC2, AKT1, VAV3, IL10, ACTR3, ACTR2, MAP2K4, ARHGEF12, ITGA4, IRAK4, CD8A, TRAF6, ELMO1, ELMO2, LCP2, DOCK1, SKAP2, ROCK1, ROCK2, SRC, ARHGEF28, PXN, PIK3R1, ACTR3B, MAPK9, MAPK8, PIP5K1A, MAPK1, PTK2B, PIP5K1B, PLCG1, WASF2, NFATC3, FN1, NFATC2, MAPK14, NFKB1, MAPK11, IL6, CD4, PKN2, TAB2, TAB1, MYD88, BCAR1* |
| **brown** | oas04728:Dopaminergic synapse | 58 | 0.92 | 0.00 | *GSK3B, ATF2, ARRB1, CALML4, PPP1CB, PPP3CA, PPP3CB, PPP3CC, KIF5C, PPP2R5E, CREB3L1, KIF5B, CREB3L2, AKT3, AKT1, PRKACB, SLC18A1, SLC18A2, PRKCB, PPP2R5A, PRKCA, PPP2R5C, PLCB4, CREB1, PLCB1, CLOCK, PLCB2, CREB5, GRIA1, GRIA2, DDC, CAMK2D, ITPR1, ITPR2, ITPR3, CACNA1D, CACNA1C, GNAI2, PPP2CA, MAPK9, GRIN2A, MAPK8, PPP2R3C, DRD3, CAMK2G, GRIA3, GRIA4, KCNJ3, PPP2R3A, GNG12, MAPK14, MAPK11, GNAL, PPP2R2B, GNB1, GNB4, GNB3, GNB5* |
| **brown** | oas04919:Thyroid hormone signaling pathway | 59 | 0.94 | 0.00 | *GSK3B, THRB, PIK3CD, ATP2A2, PIK3CB, MED16, MED17, CASP9, MED14, MED13, SIN3A, AKT3, AKT1, PLCE1, EP300, ITGAV, PRKACB, NCOA1, NCOA2, PDPK1, PRKCB, NCOA3, TSC2, PRKCA, MED4, MED27, ATP1B1, KAT2B, PLCB4, NCOR1, TBC1D4, RAF1, PLCB1, PLCB2, NOTCH2, PFKFB2, NOTCH1, SRC, GATA4, PIK3R1, MED12L, FOXO1, PLCG2, MAPK1, PLCG1, RXRG, WNT4, STAT1, SLC16A10, ESR1, MTOR, MED13L, BMP4, PFKL, RHEB, RCAN2, CTNNB1, KRAS, PLCD4* |
| **brown** | oas04722:Neurotrophin signaling pathway | 59 | 0.94 | 0.00 | *GSK3B, PRDM4, IRS1, PIK3CD, FASLG, CALML4, PIK3CB, IKBKB, RPS6KA3, RPS6KA5, RPS6KA2, AKT3, AKT1, MAP3K5, PDPK1, RIPK2, GAB1, MATK, FRS2, IRAK4, NGF, TRAF6, RAPGEF1, SOS1, RAF1, SOS2, SHC4, CAMK2D, SHC3, PSEN1, PIK3R1, FOXO3, RAP1B, MAPK9, MAPK8, RAP1A, NTF3, PLCG2, ABL1, MAPK1, PLCG1, CAMK2G, MAP2K5, NGFR, NTRK2, MAP3K3, MAP3K1, SORT1, BDNF, KIDINS220, NTRK3, BRAF, MAPK14, NFKB1, MAPK11, CAMK4, BCL2, GRB2, KRAS* |
| **brown** | oas04068:FoxO signaling pathway | 60 | 0.95 | 0.00 | *CDKN1B, IRS1, FBXO25, PTEN, PIK3CD, PRKAG2, IRS2, FASLG, PIK3CB, PRKAG3, IGF1R, IKBKB, STK11, CCND2, AKT3, TNFSF10, AKT1, EP300, IL10, PRKAB2, USP7, PDPK1, SIRT1, TGFBR1, TGFBR2, RBL2, CAT, SOS1, RAF1, SOS2, SGK2, PRKAA2, PIK3R1, NLK, FOXO3, FOXO1, EGFR, MAPK9, MAPK8, BCL2L11, MAPK1, PLK4, SMAD4, TGFB2, SMAD3, HOMER1, HOMER2, INSR, PLK1, STAT3, BRAF, IGF1, FBXO32, MAPK14, MAPK11, IL6, BCL6, GRB2, KRAS, ATM* |
| **brown** | oas04921:Oxytocin signaling pathway | 60 | 0.95 | 0.00 | *PRKAG2, CALML4, PRKAG3, MYLK3, RYR3, PPP1CB, PPP3CA, PPP3CB, PPP3CC, EEF2K, PRKACB, GUCY1A2, PRKAB2, MEF2C, PLA2G4F, PPP1R12A, CAMK1D, PRKCB, CACNA2D1, CACNA2D3, PRKCA, CACNB1, CACNB2, PLCB4, CACNB4, ADCY9, RAF1, PPP1R12B, PLCB1, PLCB2, CAMK1G, CAMK2D, PRKAA2, ROCK1, ROCK2, SRC, NPR2, ITPR1, ITPR2, ADCY3, ADCY2, ITPR3, CACNA1D, CACNA1C, ADCY8, EGFR, PIK3R6, PIK3R5, GNAI2, PPP3R1, MAPK1, CAMK2G, MAP2K5, CACNG4, KCNJ3, NFATC3, NFATC2, CAMK4, KRAS, CAMK1* |
| **brown** | oas04110:Cell cycle | 60 | 0.95 | 0.02 | *RB1, GSK3B, CDKN1B, CCNH, SMC3, CDC14A, CDC14B, NIPBL, CCND3, CCND2, CDC23, PPP2R5E, CHEK1, CDC27, EP300, ESCO1, ANAPC7, ATRX, PPP2R5A, PPP2R5C, KNL1, YWHAZ, RBL2, MAU2, DBF4, RBL1, CCNE2, TFDP2, ANAPC4, ANAPC5, MAD1L1, ANAPC1, PRKDC, CUL1, TTK, PDS5B, PDS5A, ANAPC10, PPP2CA, ORC5, ORC4, ORC1, ORC3, ORC2, ABL1, E2F3, E2F5, SMAD2, ZBTB17, SMAD4, TGFB2, SMAD3, PLK1, CDC7, WAPL, STAG1, CDK6, CDC16, ATM, ATR* |
| **brown** | oas04814:Motor proteins | 65 | 1.03 | 0.04 | *BICDL1, KIF17, DNALI1, ACTR1A, CAPZB, KIF5C, KIF5B, TUBB1, KIF13A, KIF13B, MYO18A, KIF1B, KIF21A, KIFAP3, DYNC2H1, STARD9, KIF25, KIF24, KIF23, MYO7A, KIF27, DYNC1LI1, DYNC1LI2, KIF2A, MYO3B, KIF16B, MYH9, KIF20B, DYNC1I2, DNAH1, DNAH7, DNAH8, DNAH5, DCTN4, TUBD1, DNAH9, KLC1, KIF3B, MYO6, MYH11, KIF3C, MYH10, DYNC1I1, DYNC1H1, DNAH17, MYO10, MYO5A, TUBE1, HOOK3, MYO9A, MYO19, MYO1D, MYO1E, CENPE, KIF18A, MYO1B, MYO15B, KIF26A, CAPZA1, MYO5B, DYNLRB2, ACTR10, MYO1F, DNAL1, TUBA8* |
| **brown** | oas04140:Autophagy - animal | 67 | 1.06 | 0.00 | *IRS1, MTMR14, WDR41, PTEN, PIK3CD, IRS2, PIK3CB, ZFYVE1, IGF1R, STK11, TBK1, DEPTOR, MAP1LC3C, AKT3, AKT1, SUPT20H, PRKACB, RAB8A, SH3GLB1, ATG3, RUBCN, DAPK1, PDPK1, DAPK2, TSC2, RRAS2, TSC1, WIPI2, ATG14, ATG13, RAB33B, ERN1, MRAS, RRAGC, TRAF6, ATG4C, PIK3C3, PRKCQ, RAF1, RAB7A, PRKAA2, MTMR3, STX17, ITPR1, AMBRA1, PIK3R1, MTMR4, TANK, PPP2CA, MAPK9, MAPK8, MAPK1, ATG7, ATG5, UVRAG, EIF2AK3, EIF2AK4, CFLAR, MTOR, VMP1, RPS6KB1, RHEB, ATG16L1, BCL2, KRAS, ATG2B, BCL2L1* |
| **brown** | oas04530:Tight junction | 67 | 1.06 | 0.00 | *PATJ, ARPC5L, PRKAG2, CLDN1, PRKAG3, STK11, PRKACB, RAB8A, CGNL1, RAB8B, MAP3K5, MAGI1, ACTR3, ACTR2, PRKAB2, PRKCI, ACTN1, PRKCE, SCRIB, RUNX1, TIAM1, CTTN, PARD3, MYH9, RAPGEF2, CLDN16, RAPGEF6, CFTR, LLGL2, IGSF5, PRKAA2, ROCK1, ROCK2, SRC, NEDD4L, GATA4, CACNA1D, ARHGAP17, PRKCZ, ACTR3B, PPP2CA, MAPK9, PARD6B, MAPK8, RAP1A, MARVELD3, MICALL2, MYH11, WHAMM, MPDZ, MYH10, JAM2, MAP3K1, HSPA4, CGN, TJAP1, TJP1, AFDN, DLG1, DLG2, DLG3, PPP2R2B, NEDD4, AMOTL2, NF2, AMOTL1, TUBA8* |
| **brown** | oas04022:cGMP-PKG signaling pathway | 68 | 1.08 | 0.00 | *ATF2, IRS1, PDE3B, ATP2A2, ADRA1D, IRS2, CALML4, SLC8A1, MYLK3, PPP1CB, PPP3CA, PPP3CB, EDNRA, PPP3CC, CREB3L1, CREB3L2, AKT3, CNGA1, AKT1, PRKG2, PRKG1, MEF2A, GUCY1A2, MEF2C, MEF2B, PPP1R12A, TRPC6, PRKCE, ATP1B1, PLCB4, ADCY9, CREB1, KCNMA1, RAF1, PLCB1, PLCB2, CREB5, OPRD1, ROCK1, ROCK2, NPR2, ITPR1, ITPR2, ADCY3, GATA4, ADCY2, ITPR3, CACNA1D, CACNA1C, ADCY8, KNG1, PIK3R6, PIK3R5, GNAI2, GNA13, PPP3R1, KCNMB1, GNA12, MAPK1, KCNMB4, MEF2D, INSR, NFATC3, NFATC2, ATP2B2, ATP2B1, PDE3A, GTF2IRD1* |
| **brown** | oas04934:Cushing syndrome | 69 | 1.10 | 0.00 | *RB1, SCARB1, GSK3B, ATF2, CDKN1B, WNT2B, ASH2L, AHR, CREB3L1, CREB3L2, PRKACB, PDE8A, APC2, USP8, WNT5A, AXIN1, ARNT, AXIN2, WNT9A, NR5A1, PLCB4, ADCY9, CREB1, CCNE2, ORAI1, PLCB1, PLCB2, CREB5, CAMK2D, KMT2A, LEF1, ITPR1, ITPR2, ADCY3, ADCY2, ITPR3, CACNA1D, CACNA1C, ADCY8, EGFR, GNAI2, RAP1B, PDE11A, MC2R, RAP1A, RBBP5, DVL1, DVL3, MAPK1, E2F3, WNT2, CAMK2G, WNT3, WNT4, FZD1, TCF7L2, TCF7L1, FZD3, FZD5, WNT3A, FZD6, WNT7A, BRAF, PBX1, CDK6, APC, STAR, CTNNB1, KCNK2* |
| **brown** | oas05224:Breast cancer | 70 | 1.11 | 0.00 | *RB1, GSK3B, WNT2B, PTEN, PIK3CD, BRCA1, PIK3CB, BRCA2, FGF2, IGF1R, FGF8, FGF9, HEY2, AKT3, AKT1, POLK, APC2, NCOA1, NCOA3, WNT5A, AXIN1, AXIN2, WNT9A, DDB2, KIT, PGR, SOS1, RAF1, SOS2, NOTCH2, SHC4, NOTCH1, SHC3, LEF1, LRP5, PIK3R1, DLL1, EGFR, LRP6, DVL1, DVL3, MAPK1, E2F3, WNT2, FGF23, WNT3, WNT4, FZD1, TCF7L2, TCF7L1, FZD3, JAG1, FZD5, WNT3A, CSNK1A1, FZD6, WNT7A, BRAF, IGF1, ESR1, ESR2, MTOR, CDK6, APC, RPS6KB1, FGF18, CTNNB1, GRB2, KRAS, FGF10* |
| **brown** | oas05161:Hepatitis B | 70 | 1.11 | 0.00 | *RB1, ATF2, PIK3CD, FASLG, PIK3CB, CASP9, IFIH1, IKBKB, CASP8, TBK1, CASP3, CREB3L1, CREB3L2, AKT3, AKT1, EP300, JAK2, IKBKE, JAK1, MAP2K4, APAF1, PRKCB, PRKCA, IRAK4, YWHAZ, TGFBR1, DDB2, TGFBR2, CREB1, CCNE2, TRAF3, TRAF6, SOS1, RAF1, SOS2, TLR3, CREB5, TLR2, IFNAR1, SRC, PIK3R1, MAPK9, MAPK8, STAT4, MAPK1, PTK2B, E2F3, STAT6, BID, SMAD4, TGFB2, SMAD3, MAP3K1, STAT1, SLC10A1, STAT3, NFATC3, NFATC2, BRAF, MAPK14, NFKB1, MAPK11, IL6, BCL2, FAS, GRB2, KRAS, TAB2, TAB1, MYD88* |
| **brown** | oas04072:Phospholipase D signaling pathway | 71 | 1.13 | 0.00 | *DGKE, DGKD, DGKB, PIK3CD, PIK3CB, GRM3, GRM2, GRM7, GRM8, AKT3, AKT1, PDGFRB, PDGFRA, PLA2G4F, SYK, GAB1, TSC2, RRAS2, TSC1, PRKCA, GAB2, AVPR1A, DNM1, MRAS, PLCB4, ADCY9, KIT, SOS1, PLPP3, RAF1, PLCB1, SOS2, PLCB2, DGKI, DGKH, SHC4, PTGFR, RALA, RALB, SHC3, PDGFB, LPAR1, ADCY3, ADCY2, LPAR3, PIK3R1, ADCY8, PLD1, AGPAT3, EGFR, PIK3R6, AGPAT4, PIK3R5, GNA13, PDGFD, GNA12, PDGFC, PLCG2, PIP5K1A, MAPK1, PTK2B, PIP5K1B, FYN, PLCG1, RALGDS, INSR, MTOR, KITLG, RHEB, GRB2, KRAS* |
| **brown** | oas04218:Cellular senescence | 71 | 1.13 | 0.00 | *RB1, LIN54, TRAF3IP2, PTEN, PIK3CD, CALML4, PIK3CB, ETS1, PPP1CB, PPP3CA, PPP3CB, CCND3, PPP3CC, CCND2, RASSF5, CHEK1, AKT3, AKT1, NBN, BTRC, LIN52, FBXW11, TSC2, RRAS2, HUS1, TSC1, SIRT1, TGFBR1, TGFBR2, RBL2, MRAS, RBL1, CCNE2, RAF1, ITPR1, ITPR2, GATA4, ITPR3, CACNA1D, LIN9, PIK3R1, FOXO3, FOXO1, ZFP36L2, ZFP36L1, PPP3R1, MAPK1, E2F3, TRPM7, E2F5, SMAD2, HIPK4, TGFB2, SMAD3, NFATC3, NFATC2, MAPK14, HIPK1, NFKB1, HIPK3, MTOR, HIPK2, MAPK11, IL6, RAD50, CDK6, RHEB, KRAS, ATM, MCU, ATR* |
| **brown** | oas05226:Gastric cancer | 72 | 1.14 | 0.00 | *RB1, GSK3B, CDKN1B, WNT2B, PIK3CD, PIK3CB, FGF2, FGF8, FGF9, AKT3, AKT1, POLK, APC2, HGF, WNT5A, GAB1, AXIN1, AXIN2, WNT9A, TGFBR1, DDB2, TGFBR2, CCNE2, RARB, SOS1, RAF1, SOS2, MET, SHC4, ABCB1, SHC3, LEF1, LRP5, PIK3R1, EGFR, LRP6, DVL1, CTNNA1, DVL3, MAPK1, CTNNA3, E2F3, WNT2, FGF23, RXRG, WNT3, WNT4, SMAD2, FZD1, TCF7L2, TCF7L1, SMAD4, FZD3, TGFB2, SMAD3, FZD5, WNT3A, CSNK1A1, FZD6, WNT7A, BRAF, MLH1, MTOR, APC, RPS6KB1, FGF18, BCL2, CTNNB1, GRB2, KRAS, FGFR2, FGF10* |
| **brown** | oas04150:mTOR signaling pathway | 72 | 1.14 | 0.00 | *GSK3B, WNT2B, CAB39L, IRS1, PTEN, PIK3CD, PIK3CB, IGF1R, IKBKB, RPS6KA3, FLCN, STK11, DEPTOR, RPS6KA2, AKT3, GRB10, AKT1, RNF152, MIOS, PDPK1, PRKCB, STRADA, WNT5A, TSC2, TSC1, PRKCA, WNT9A, CLIP1, RRAGC, SOS1, DEPDC5, RAF1, SOS2, TELO2, PRKAA2, SEH1L, CAB39, MAPKAP1, NPRL3, LRP5, PRR5L, PIK3R1, LRP6, DVL1, ATP6V1H, DVL3, MAPK1, SLC38A9, RICTOR, FNIP1, WNT2, FNIP2, ATP6V1C1, WNT3, ATP6V1C2, WNT4, FZD1, FZD3, WDR59, FZD5, WNT3A, INSR, FZD6, WNT7A, BRAF, IGF1, MTOR, RPS6KB1, RHEB, GRB2, KRAS, LPIN2* |
| **brown** | oas04062:Chemokine signaling pathway | 73 | 1.16 | 0.00 | *GSK3B, ITK, PIK3CD, ARRB1, PIK3CB, CXCL14, IKBKB, PREX1, AKT3, CCR9, RAC2, AKT1, JAK2, PRKACB, CCR4, VAV3, PRKCB, TIAM1, HCK, PLCB4, ADCY9, PARD3, ELMO1, DOCK2, SOS1, RAF1, PLCB1, SOS2, PLCB2, SHC4, SHC3, ROCK1, ROCK2, SRC, PXN, CXCR5, CXCR4, ADCY3, ADCY2, PIK3R1, ADCY8, FOXO3, PRKCZ, PIK3R6, PIK3R5, GNAI2, RAP1B, PAK1, GRK2, RAP1A, GRK5, GRK4, GRK7, GRK6, PLCG2, MAPK1, PTK2B, PLCG1, CCR10, LYN, STAT1, STAT3, BRAF, GNG12, NFKB1, CXCL12, GNB1, GNB4, GNB3, GRB2, KRAS, GNB5, BCAR1* |
| **brown** | oas04550:Signaling pathways regulating pluripotency of stem cells | 74 | 1.17 | 0.00 | *GSK3B, BMPR2, WNT2B, RIF1, PIK3CD, PIK3CB, BMI1, FGF2, IGF1R, AKT3, AKT1, SMARCAD1, JAK2, JARID2, JAK1, ACVR1, APC2, WNT5A, AXIN1, AXIN2, WNT9A, KAT6A, HAND1, IL6ST, RAF1, PIK3R1, ACVR1B, DVL1, HESX1, DVL3, MAPK1, WNT2, SKIL, WNT3, WNT4, SMAD2, FZD1, SMAD1, PCGF6, SMAD4, FZD3, ZFHX3, SMAD3, FZD5, SETDB1, WNT3A, PCGF5, PCGF2, FZD6, STAT3, PCGF3, LIF, SMAD9, WNT7A, INHBA, IGF1, MAPK14, ACVR2B, SMAD5, ACVR2A, BMP4, MAPK11, MEIS1, APC, ID2, CTNNB1, GRB2, LHX5, KRAS, TCF3, FGFR4, BMPR1B, FGFR2, BMPR1A* |
| **brown** | oas04120:Ubiquitin mediated proteolysis | 75 | 1.19 | 0.00 | *UBE3C, UBE2D2, UBE2D3, UBE2D1, UBE3A, CBLB, BRCA1, UBE2Z, UBE2L3, CDC23, HERC1, CDC27, FBXO4, BTRC, PIAS4, FBXW8, ANAPC7, FBXW7, FBXW11, FBXO2, UBE2E3, UBE4A, UBE4B, PIAS2, DDB2, PIAS1, UBE2R2, TRAF6, ANAPC4, BIRC6, ANAPC5, BIRC2, ANAPC1, CUL5, UBA7, UBA6, MGRN1, CUL3, CUL2, CUL1, NEDD4L, UBE2J2, CBL, RCHY1, RHOBTB1, ANAPC10, UBE2J1, UBR5, UBE2F, UBE2H, MAP3K1, PPIL2, SMURF2, UBE2B, FANCL, SMURF1, SIAH1, WWP1, UBE2G1, WWP2, UBE2G2, PML, UBE2W, ITCH, UBOX5, NEDD4, CDC16, UBE2N, ERCC8, UBE2O, UBA2, TRIP12, TRIM37, UBE2K, CUL4B* |
| **brown** | oas04390:Hippo signaling pathway | 76 | 1.21 | 0.00 | *GSK3B, PATJ, BMPR2, WNT2B, WWC1, ITGB2, GLI2, PPP1CB, CCND3, CCND2, RASSF6, BTRC, TEAD1, TEAD4, APC2, PRKCI, FBXW11, WNT5A, AXIN1, SCRIB, CSNK1D, AXIN2, WNT9A, YWHAZ, TGFBR1, TGFBR2, LATS1, FRMD6, PARD3, BIRC2, LLGL2, YAP1, CRB2, LEF1, PRKCZ, STK3, PPP2CA, PARD6B, DVL1, TP53BP2, CTNNA1, DVL3, CTNNA3, WNT2, WNT3, WNT4, SMAD2, FZD1, WWTR1, SMAD1, TCF7L2, TCF7L1, SMAD4, FZD3, TGFB2, SMAD3, FZD5, WNT3A, FZD6, WNT7A, GDF5, BMP6, SMAD7, MOB1B, BMP4, DLG1, DLG2, APC, DLG3, PPP2R2B, ID2, DLG5, CTNNB1, NF2, BMPR1B, BMPR1A* |
| **brown** | oas05202:Transcriptional misregulation in cancer | 77 | 1.22 | 0.00 | *CDKN1B, CCNT2, CCNT1, FLT3, JMJD1C, PLAT, BMI1, AFF1, IGF1R, ELK4, CCND2, SIN3A, NFKBIZ, POLK, KDM6A, SS18, MEF2C, IL1R2, MITF, ETV1, TRAF1, ETV4, RUNX2, SUPT3H, RUNX1, DDB2, TGFBR2, ETV6, MAF, NCOR1, ZEB1, PAX7, RARA, BMP2K, MET, BIRC2, CSF1R, SLC45A3, BCL2A1, KMT2A, HPGD, MAX, TMPRSS2, MLLT1, MLLT3, FOXO1, FUT8, PROM1, RXRG, GRIA3, SMAD1, NGFR, ZBTB17, ARNT2, TAF15, BCL11B, ZBTB16, SS18L1, PBX3, HMGA2, IGF1, KLF3, NFKB1, FLI1, PBX1, PML, PER2, IL6, MEIS1, BCL6, ID2, IL2RB, REL, ATM, TCF3, CDK14, BCL2L1* |
| **brown** | oas04024:cAMP signaling pathway | 78 | 1.24 | 0.02 | *PDE3B, PIK3CD, ATP2A2, CALML4, PIK3CB, HTR4, GLI3, PPP1CB, EDNRA, CREB3L1, CREB3L2, AKT3, RAC2, PDE4B, CNGA1, AKT1, PLCE1, EP300, PRKACB, VAV3, PPP1R12A, PDE4D, RRAS2, ATP1B1, TIAM1, ADCY9, CREB1, FSHR, CNGB3, ORAI1, PPARA, RAF1, CFTR, CREB5, GRIA1, GRIA2, POPDC3, ADCYAP1R1, CAMK2D, SUCNR1, ROCK1, ROCK2, GPR119, ADCY3, ADCY2, CACNA1D, CACNA1C, PIK3R1, ADCY8, PLD1, GNAI2, RAP1B, MAPK9, GRIN2A, MAPK8, PAK1, MC2R, RAP1A, MAPK1, CAMK2G, GRIA3, GRIA4, GABBR2, BDNF, PTCH1, ATP2B2, BRAF, ATP2B1, NFKB1, TSHR, GRIN1, AFDN, GRIN3B, PDE10A, CAMK4, PDE3A, GHRL, KCNK2* |
| **brown** | oas05206:MicroRNAs in cancer | 79 | 1.25 | 0.00 | *TRIM71, CDKN1B, BMPR2, IRS1, TNC, PTEN, PIK3CD, IRS2, BRCA1, PIK3CB, BMI1, GLS, IKBKB, CCND2, RPS6KA5, TNN, CASP3, DNMT3B, TNR, EP300, TP63, APC2, PDGFRB, PDGFRA, PRKCB, PRKCE, KIF23, PRKCA, DICER1, SIRT1, FOXP1, ZEB2, ZEB1, CCNE2, MMP16, SOS1, ZFPM2, RAF1, SOS2, MET, CD44, NOTCH2, HDAC4, SHC4, NOTCH1, SLC45A3, ABCB1, ROCK1, PDGFB, PIK3R1, EFNA5, THBS1, HOXD10, EGFR, BCL2L11, PLCG2, ABL1, MAPK1, E2F3, BMF, PLCG1, WNT3, FZD3, TGFB2, WNT3A, STAT3, HMGA2, NFKB1, MTOR, CDK6, HNRNPK, APC, BCL2, GRB2, KRAS, ATM, MDM4, RECK, EZH2* |
| **brown** | oas05225:Hepatocellular carcinoma | 81 | 1.29 | 0.00 | *RB1, GSK3B, WNT2B, PTEN, PIK3CD, PIK3CB, IGF1R, AKT3, AKT1, DPF3, POLK, ARID2, APC2, PBRM1, SMARCC1, PRKCB, HGF, ACTL6A, WNT5A, GAB1, AXIN1, PRKCA, AXIN2, WNT9A, ARID1A, ARID1B, TGFBR1, DDB2, TGFBR2, SOS1, RAF1, SOS2, MET, SHC4, SMARCD1, SMARCD2, SHC3, SMARCD3, LEF1, LRP5, MGST2, PIK3R1, EGFR, LRP6, DVL1, PLCG2, DVL3, MAPK1, E2F3, PLCG1, BRD7, WNT2, WNT3, WNT4, SMAD2, FZD1, NQO1, TCF7L2, TCF7L1, SMAD4, FZD3, TGFB2, SMAD3, FZD5, TXNRD3, WNT3A, TXNRD2, CSNK1A1, FZD6, WNT7A, BRAF, SMARCA2, MTOR, CDK6, APC, RPS6KB1, CTNNB1, GRB2, KRAS, BCL2L1, NFE2L2* |
| **brown** | oas05166:Hμman T-cell leukemia virus 1 infection | 81 | 1.29 | 0.01 | *RB1, NRP1, ATF2, ITGB2, PTEN, PIK3CD, PIK3CB, CD3E, ETS1, ELK4, IKBKB, PPP3CA, PPP3CB, CCND3, XPO1, PPP3CC, CCND2, CDC23, CREB3L1, CREB3L2, CHEK1, AKT3, CDC27, AKT1, EP300, PRKACB, JAK1, MAP2K4, TBP, ANAPC7, MSX2, IL15, IL1R2, TGFBR1, TGFBR2, KAT2B, ADCY9, CREB1, CCNE2, CANX, TBPL1, ANAPC4, ANAPC5, TLN2, MAD1L1, ANAPC1, CREB5, VAC14, ADCY3, ADCY2, PIK3R1, ADCY8, IL2RG, ANAPC10, MAPK9, PPP3R1, MAPK8, MAPK1, E2F3, SMAD2, MAP3K3, SMAD4, TGFB2, SMAD3, RANBP3, MAP3K1, NFYB, NFATC3, NFATC2, NFKB1, IL6, DLG1, CD4, IL2RB, CDC16, KRAS, ATM, TCF3, MAP3K14, ATR, BCL2L1* |
| **brown** | oas05417:Lipid and atherosclerosis | 84 | 1.33 | 0.02 | *GSK3B, NCF2, NCF4, PIK3CD, FASLG, CALML4, VLDLR, PIK3CB, CASP9, IKBKB, PPP3CA, PPP3CB, CASP7, CASP8, PPP3CC, TBK1, CASP6, CASP3, AKT3, TNFSF10, AKT1, JAK2, IKBKE, MAP3K5, VAV3, MAP2K4, APAF1, PDPK1, PRKCA, IRAK4, ERN1, PLCB4, TRAF3, TRAF6, PLCB1, ATF6, PLCB2, ABCG1, TLR2, CAMK2D, ROCK2, SRC, ITPR1, LY96, PIK3R1, TANK, HSP90B1, RAP1B, MAPK9, PPP3R1, MAPK8, RAP1A, MAPK1, PLCG1, BID, CAMK2G, RXRG, ABCA1, LYN, HSPA8, POU2F1, HSPA4, STAT3, MIB2, EIF2AK3, NFATC3, NFATC2, MAPK14, POU2F3, NFKB1, MIB1, SELP, MAPK11, IL6, CD40LG, BCL2, FAS, KRAS, TAB2, TAB1, MYD88, NOX1, BCL2L1, NFE2L2* |
| **brown** | oas04310:Wnt signaling pathway | 86 | 1.37 | 0.00 | *INVS, GSK3B, WNT2B, CTNND2, PPP3CA, PPP3CB, CCND3, PPP3CC, CCND2, RAC2, RSPO3, EP300, RSPO1, BTRC, PRKACB, RSPO4, APC2, TLE4, TLE3, RNF43, CSNK2A1, FBXW11, PRKCB, CSNK2A2, WNT5A, CTNNBIP1, AXIN1, PRKCA, AXIN2, WNT9A, SENP2, DKK1, PLCB4, DAAM1, DAAM2, TBL1XR1, ROR1, ROR2, PLCB1, PLCB2, PPARD, CAMK2D, CTBP2, ROCK2, LEF1, CUL1, PRICKLE2, LRP5, PSEN1, PRICKLE1, APCDD1, NLK, CXXC4, LRP6, MAPK9, PPP3R1, MAPK8, WIF1, DVL1, DVL3, WNT2, CAMK2G, WNT3, WNT4, FZD1, TCF7L2, TCF7L1, SMAD4, FZD3, SMAD3, FZD5, WNT3A, RYK, CSNK1A1, FZD6, SIAH1, NFATC3, NFATC2, WNT7A, VANGL1, APC, CCDC88C, CTNNB1, NOTμm, LGR5, LGR4* |
| **brown** | oas05163:Hμman cytomegalovirus infection | 89 | 1.41 | 0.01 | *RB1, GSK3B, ATF2, PIK3CD, FASLG, CALML4, PIK3CB, CASP9, IKBKB, PPP3CA, AKAP13, PPP3CB, CASP8, PPP3CC, TBK1, CASP3, CREB3L1, CREB3L2, AKT3, RAC2, AKT1, ITGAV, IL6R, PRKACB, JAK1, PDGFRA, ARHGEF12, PRKCB, TAP2, TAP1, TSC2, TSC1, PRKCA, PLCB4, ADCY9, CREB1, TRAF5, SOS1, RAF1, PLCB1, SOS2, PLCB2, CREB5, PTGER4, ROCK1, ROCK2, SRC, PXN, ITPR1, CXCR4, ITPR2, ADCY3, ADCY2, ITPR3, PIK3R1, ADCY8, EGFR, GNAI2, GNA13, PPP3R1, GNA12, MAPK1, PTK2B, E2F3, RIPK1, BID, IL10RB, STAT3, NFATC3, NFATC2, GNG12, MAPK14, NFKB1, MTOR, MAPK11, IL6, CDK6, CXCL12, RPS6KB1, RHEB, GNB1, GNB4, CTNNB1, FAS, GNB3, GRB2, KRAS, GNB5, BCAR1* |
| **brown** | oas05205:Proteoglycans in cancer | 94 | 1.49 | 0.00 | *WNT2B, HPSE2, ITGB5, PIK3CD, FASLG, PIK3CB, FGF2, IGF1R, PPP1CB, CASP3, AKT3, KDR, AKT1, PLCE1, ITGAV, PRKACB, VAV3, ARHGEF12, PPP1R12A, PDPK1, PRKCB, HGF, ITGA2, WNT5A, GAB1, RRAS2, FRS2, PRKCA, ANK2, WNT9A, ANK1, TIAM1, MRAS, SMO, CTTN, SOS1, RAF1, PPP1R12B, SOS2, MET, CD44, TLR2, HBEGF, CAMK2D, SDC4, ROCK1, ROCK2, SRC, PXN, ITPR1, ITPR2, TWIST1, ITPR3, PIK3R1, IQGAP1, CBL, THBS1, HOXD10, EGFR, PAK1, ERBB4, PLCG2, DROSHA, MAPK1, FLNB, PLCG1, WNT2, CAMK2G, WNT3, WNT4, SMAD2, FZD1, FZD3, TGFB2, FZD5, WNT3A, PTCH1, CAV1, FZD6, STAT3, FN1, WNT7A, BRAF, IGF1, MAPK14, ESR1, MTOR, MAPK11, RPS6KB1, CTNNB1, FAS, GRB2, KRAS, HPSE* |
| **brown** | oas04020:Calciμm signaling pathway | 97 | 1.54 | 0.00 | *CALML4, HTR4, FGF2, SLC8A1, MYLK3, EDNRA, FGF8, HTR7, FGF9, KDR, PLCE1, PRKACB, PDGFRB, PDGFRA, PRKCB, HGF, PRKCA, PHKA1, PHKA2, TRHR, ADCY9, STIM1, STIM2, ORAI1, PTGFR, PDE1A, ITPR1, PDGFB, ITPR2, ITPR3, CACNA1D, MST1R, CACNA1C, GRIN2A, PPP3R1, HRH1, HRH2, PDGFD, PDGFC, PLCG2, PLCG1, FGF23, NFATC3, NFATC2, PHKB, GRIN3B, GNAL, FGF18, FGFR4, FGFR2, MCU, FGF10, ATP2A2, ADRA1D, HTR2A, RYR3, PPP3CA, PPP3CB, PPP3CC, NOS1, CAMK1D, MST1, TFEB, TACR3, AVPR1A, NGF, ITPKB, PLCB4, ASPH, ADORA2B, PLCB1, MET, PLCB2, CAMK1G, CAMK2D, CXCR4, ADCY3, ADCY2, ADCY8, EGFR, ERBB4, PTK2B, CAMK2G, NTRK2, NOS2, NTRK3, VEGFC, ATP2B2, ATP2B1, MCOLN2, TPCN1, GRIN1, P2RX7, P2RX4, CAMK4, CAMK1, PLCD4* |
| **brown** | oas05132:Salmonella infection | 99 | 1.57 | 0.00 | *CYFIP1, NCKAP1, CSE1L, ARPC5L, GCC2, KIF5C, KIF5B, TUBB1, AKT3, TNFSF10, AKT1, KPNA4, KPNA3, VPS39, KPNA1, DYNC2H1, MAP2K4, VPS33A, DYNC1LI1, DYNC1LI2, ELMO1, ELMO2, RAF1, ARL8B, ARHGEF26, LY96, KLC1, ACTR3B, HSP90B1, MYO6, VPS11, SNX9, RIPK1, NFKB1, IL6, PTPRC, PLEKHM1, BCL2, PLEKHM2, TAB3, TAB2, DYNLRB2, TAB1, ACBD3, RAB5A, MYD88, TUBA8, PIK3CD, PIK3C2G, WASL, PIK3CB, PIK3C2A, PIK3C2B, IKBKB, FYCO1, CASP7, ACTR1A, CASP8, CASP3, ACTR3, ACTR2, RIPK2, RHOH, IRAK4, TRAF6, RHOJ, EXOC4, PIK3C3, EXOC5, TLR5, EXOC2, BIRC2, TLR2, RAB7A, DYNC1I2, RALA, ROCK2, DCTN4, LEF1, NOD1, SNX33, MAPK9, MAPK8, PAK1, MAPK1, FLNB, PAK3, WASF3, DYNC1I1, DYNC1H1, TCF7L2, TCF7L1, AHNAK2, MAPK14, MAPK11, ABI1, VPS41, CTNNB1, ACTR10* |
| **brown** | oas04360:Axon guidance | 102 | 1.62 | 0.00 | *EPHB6, SEMA5B, DPYSL5, PLXNC1, EPHB2, EPHA5, EPHA4, EPHA7, EPHA6, SEMA6A, UNC5A, UNC5B, SEMA6D, WNT5A, PRKCA, UNC5C, UNC5D, SMO, PARD3, RAF1, NGEF, EPHA3, SEMA7A, PIK3R1, EFNA5, PRKCZ, EFNB2, PPP3R1, PLCG2, ABL1, PLXNA2, SRGAP3, PLCG1, SRGAP2, LRRC4C, SRGAP1, WNT4, NTNG1, FZD3, PTCH1, NFATC3, NFATC2, SSH2, CXCL12, FES, PLXNB1, BMPR1B, ROBO2, GSK3B, NRP1, BMPR2, PIK3CD, PIK3CB, ROBO1, PPP3CA, PPP3CB, PPP3CC, BOC, NCK2, RAC2, NEO1, TRPC5, ARHGEF12, TRPC6, PDPK1, TRPC4, TRPC1, RGMA, ENAH, RASA1, MET, CAMK2D, ROCK1, SEMA3C, ROCK2, SRC, SEMA3D, SEMA3G, CXCR4, SEMA3E, NTN1, GNAI2, PARD6B, ABLIM1, PAK1, ABLIM2, ABLIM3, SLIT1, MAPK1, PAK6, FYN, SLIT3, LRIG2, SLIT2, PAK3, CAMK2G, PAK2, PAK5, SEMA4D, RYK, SEMA4B, KRAS* |
| **brown** | oas04015:Rap1 signaling pathway | 103 | 1.63 | 0.00 | *CSF1, ITGA2B, CTNND1, CALML4, FGF2, IGF1R, FGF8, FGF9, RASSF5, AKT3, KDR, AKT1, PLCE1, MAGI1, PDGFRB, PDGFRA, PRKCI, PRKCB, MAGI3, HGF, MAGI2, PRKCA, TIAM1, ADCY9, PRKD3, PARD3, TLN2, PRKD1, RAF1, SKAP1, CSF1R, PDGFB, LPAR1, LPAR3, PIK3R1, EFNA5, PRKCZ, RAP1B, GRIN2A, RAP1A, PDGFD, PDGFC, PLCG1, RALGDS, FGF23, NGFR, INSR, BRAF, IGF1, AFDN, FGF18, FGFR4, FGFR2, FGF10, DOCK4, ITGB2, PIK3CD, PIK3CB, SIPA1L2, SIPA1L1, RAC2, VAV3, NGF, ENAH, MRAS, PLCB4, ADORA2B, KIT, RAPGEF1, RAPGEF2, LCP2, EVL, RAPGEF5, PLCB1, MET, RAPGEF6, PLCB2, RALA, RALB, RGS14, SRC, ADCY3, ADCY2, ADCY8, THBS1, EGFR, GNAI2, PARD6B, P2RY1, KRIT1, MAPK1, FARP2, ANGPT1, VEGFC, MAPK14, GRIN1, MAPK11, KITLG, CTNNB1, KRAS, TEK, BCAR1, F2RL3* |
| **brown** | oas04810:Regulation of actin cytoskeleton | 103 | 1.63 | 0.00 | *CYFIP1, NCKAP1, ITGA2B, ARPC5L, FGF2, ARHGAP35, MYLK3, PPP1CB, FGF8, FGF9, AKT3, AKT1, PIP4K2A, PIP4K2B, PDGFRB, PDGFRA, ACTN1, TIAM1, RAF1, VCL, PXN, PDGFB, LPAR1, PIK3R1, IQGAP1, IQGAP2, ACTR3B, KNG1, C6, C7, PDGFD, PDGFC, PIP5K1A, MYH11, PIP5K1B, MYH10, FGF23, FN1, BRAF, SSH2, GNG12, DIAPH2, DIAPH3, CXCL12, ITGA11, FGF18, FGFR4, FGFR2, FGF10, ITGB5, ITGB4, ITGB2, PIK3CD, ITGAE, WASL, PIK3CB, RAC2, ITGB8, ITGAV, ITGB6, GIT1, APC2, VAV3, ACTR3, ACTR2, ARHGEF12, PPP1R12A, ITGA4, ITGA3, ITGA2, ITGA1, RRAS2, ENAH, MRAS, ITGA8, ARHGEF4, MYH9, ITGA6, SOS1, PPP1R12B, DOCK1, SOS2, ITGA9, ARHGEF6, ROCK1, ROCK2, SRC, CXCR4, EGFR, GNA13, FGD3, PAK1, GNA12, MAPK1, PAK6, PAK3, WASF2, PAK2, PAK5, PIKFYVE, APC, KRAS, BCAR1* |
| **brown** | oas04510:Focal adhesion | 104 | 1.65 | 0.00 | *ITGA2B, TNC, ARHGAP35, MYLK3, IGF1R, PPP1CB, CCND3, CCND2, TNN, AKT3, KDR, AKT1, TNR, PDGFRB, PDGFRA, PRKCB, ACTN1, HGF, PRKCA, COL4A3, COL4A5, TLN2, RAF1, VCL, SHC4, SHC3, PXN, PDGFB, PIK3R1, RAP1B, RAP1A, PDGFD, PDGFC, PIP5K1A, PIP5K1B, CAV1, FN1, PARVA, BRAF, IGF1, ITGA11, BCL2, COL9A1, GRB2, GSK3B, ITGB5, LAMC3, ITGB4, PTEN, PIK3CD, LAMC2, LAMC1, PIK3CB, ARHGAP5, RAC2, ITGB8, ITGAV, ITGB6, VAV3, PPP1R12A, ITGA4, VWF, PDPK1, ITGA3, ITGA2, ITGA1, EMP2, COL2A1, COL6A2, RAPGEF1, ITGA8, ITGA6, COL6A6, SOS1, PPP1R12B, DOCK1, SOS2, MET, BIRC2, ITGA9, ROCK1, LAMA2, ROCK2, SRC, LAMA4, LAMA3, THBS1, EGFR, MAPK9, MAPK8, PAK1, RELN, MAPK1, PAK6, FLNB, FYN, PAK3, PAK2, PAK5, LAMB3, VEGFC, LAMB1, CTNNB1, BCAR1* |
| **brown** | oas04014:Ras signaling pathway | 110 | 1.75 | 0.00 | *CSF1, CALML4, ETS1, FGF2, IGF1R, TBK1, FGF8, HTR7, FGF9, RASSF5, AKT3, KDR, AKT1, PLCE1, PRKACB, PDGFRB, PDGFRA, RALBP1, RALGAPA1, PRKCB, HGF, RALGAPA2, PRKCA, TIAM1, RALGAPB, RAF1, SHC4, CSF1R, SHC3, PDGFB, RASAL1, RASAL2, PIK3R1, PLD1, EFNA5, PLA2G6, RASAL3, RASGRP1, RAP1B, GRIN2A, RAP1A, PDGFD, PDGFC, PLCG2, ABL1, ABL2, PLCG1, RALGDS, FGF23, NGFR, BDNF, INSR, IGF1, GNG12, NFKB1, AFDN, GNB1, FGF18, REL, GNB4, NF1, GNB3, GRB2, GNB5, FGFR4, RAB5A, FGFR2, BCL2L1, FGF10, FLT3, RASGRF2, PIK3CD, FASLG, PIK3CB, IKBKB, RAC2, PLA2G4F, PLA2G12B, GAB1, RRAS2, GAB2, NGF, MRAS, RASA1, RASA2, KIT, RAPGEF5, SOS1, SOS2, MET, EXOC2, RALA, RALB, EGFR, MAPK9, MAPK8, PAK1, NTF3, MAPK1, PAK6, PAK3, PAK2, PAK5, NTRK2, ANGPT1, VEGFC, GRIN1, KITLG, KRAS, TEK* |
| **brown** | oas04144:Endocytosis | 117 | 1.86 | 0.00 | *SH3GL3, TFRC, ZFYVE9, WIPF1, WIPF3, ARPC5L, CBLB, IGF1R, KIF5C, KIF5B, SH3GL2, SH3GLB1, PDGFRA, USP8, PRKCI, IST1, RUFY1, ACAP3, ACAP2, ZFYVE16, HGS, BIN1, PARD3, RUFY2, AMPH, STAM2, NEDD4L, VPS26A, VPS26B, AGAP3, PLD1, PRKCZ, ACTR3B, SNX3, SNX4, SNX1, RAB11FIP1, GRK2, SNX2, GRK5, GRK4, GRK7, GRK6, PIP5K1A, PIP5K1B, RAB11FIP2, RAB11FIP3, SNX5, SNX6, SMAD2, HSPA8, SMAD3, SMURF2, SMURF1, IQSEC1, CAV1, VTA1, STAM, SNF8, IGF2R, EHD4, CHMP2B, FGFR4, RAB5A, FGFR2, CLTC, ARRB1, WASL, RAB22A, EEA1, CAPZB, PSD2, PSD3, LDLRAP1, RAB8A, GIT1, ACTR3, ACTR2, ARAP2, VPS37C, EPS15L1, ARFGAP3, RBSN, DNM1, TGFBR1, ARFGAP2, EPN2, TGFBR2, RABEP1, DNAJC6, TRAF6, RAB7A, SRC, ASAP3, CXCR4, ASAP1, ASAP2, CBL, IL2RG, EGFR, PARD6B, MVB12B, EPS15, ARFGEF1, ARFGEF2, GBF1, WWP1, AP2B1, PML, RAB10, ITCH, CAPZA1, NEDD4, IL2RB, VPS45, SMAP2, SMAP1* |
| **brown** | oas04010:MAPK signaling pathway | 134 | 2.13 | 0.00 | *ATF2, CSF1, FGF2, IGF1R, ELK4, RPS6KA3, RPS6KA5, FGF8, FGF9, RPS6KA2, AKT3, KDR, AKT1, MAP3K8, MAP3K9, PRKACB, MAP3K4, MAP3K5, PDGFRB, PDGFRA, MAP2K4, MEF2C, PRKCB, HGF, CACNA2D1, CACNA2D3, PRKCA, MAPK8IP3, MAPK8IP1, EREG, CACNB1, CACNB2, CACNB4, MAPKAPK3, RAF1, CSF1R, MAX, PDGFB, CACNA1D, CACNA1C, EFNA5, NLK, RASGRP1, STK3, RAP1B, PPP3R1, RAP1A, PDGFD, PDGFC, FGF23, MAP2K5, HSPA8, NGFR, TGFB2, BDNF, INSR, NFATC3, BRAF, IGF1, GNG12, NFKB1, PPP5C, FGF18, NF1, TAB2, GRB2, TAB1, MAP3K13, FGFR4, MAP3K14, MYD88, FGFR2, FGF10, PTPRR, FLT3, RASGRF2, ARRB1, FASLG, ECSIT, DUSP16, IKBKB, PPP3CA, PPP3CB, PPP3CC, CASP3, RAC2, DUSP4, PLA2G4F, RRAS2, IRAK4, NGF, TGFBR1, TGFBR2, PPM1A, PPM1B, MRAS, TRAF6, RASA1, RASA2, KIT, RAPGEF2, MAPT, SOS1, SOS2, MET, EGFR, MAPK9, MAPK8, PAK1, ERBB4, MKNK1, GNA12, NTF3, MAPK1, FLNB, PAK2, CACNG4, MAP4K3, MAP4K4, MAP3K2, NTRK2, MAP3K3, MAP3K1, ANGPT1, VEGFC, MAPK14, MAPK11, KITLG, TAOK3, TAOK1, FAS, KRAS, TEK, PTPN5* |
| **brown** | oas05165:Hμman papillomavirus infection | 150 | 2.38 | 0.00 | *RB1, PATJ, MAML2, MAML1, ITGA2B, TNC, UBE3A, RBPJ, CCND3, TBK1, CCND2, TNN, CREB3L1, CREB3L2, AKT3, AKT1, EP300, TNR, PRKACB, MAGI1, PDGFRB, IFNAR2, PRKCI, WNT5A, TSC2, TSC1, SCRIB, CCNE2, PARD3, COL4A3, TBPL1, COL4A5, MAML3, ATP6V0D1, RAF1, ATP6V0D2, LLGL2, IFNAR1, NOTCH2, NOTCH1, PXN, UBR4, PSEN1, PIK3R1, PRKCZ, FOXO1, DVL1, ATP6V0A2, ATP6V1H, DVL3, WNT2, WNT3, ATP6V1C1, WNT4, ATP6V0A1, ATP6V1C2, FZD1, FZD3, JAG1, FZD5, WNT3A, FZD6, FN1, NFKB1, DLG1, CDK6, DLG2, DLG3, RHEB, ITGA11, COL9A1, GRB2, ATM, ATR, GSK3B, CDKN1B, WNT2B, ITGB5, LAMC3, ITGB4, PTEN, PIK3CD, CHD4, FASLG, LAMC2, LAMC1, PIK3CB, IKBKB, CASP8, PPP2R5E, CASP3, HEY2, ITGB8, ITGAV, ITGB6, IKBKE, JAK1, APC2, TBP, ITGA4, VWF, ITGA3, ITGA2, ITGA1, AXIN1, PPP2R5A, AXIN2, PPP2R5C, WNT9A, RBL2, COL2A1, CREB1, RBL1, NFX1, TRAF3, COL6A2, ITGA8, ITGA6, COL6A6, SOS1, SOS2, TLR3, ITGA9, CREB5, PTGER4, LAMA2, LAMA4, LAMA3, THBS1, EGFR, PPP2CA, PARD6B, RELN, PPP2R3C, MAPK1, TCF7L2, TCF7L1, LAMB3, STAT1, CSNK1A1, WNT7A, LAMB1, PPP2R3A, MTOR, APC, RPS6KB1, PPP2R2B, CTNNB1, FAS, KRAS* |
| **brown** | oas04151:PI3K-Akt signaling pathway | 153 | 2.43 | 0.00 | *ATF2, CSF1, IRS1, ITGA2B, TNC, FGF2, IGF1R, STK11, CCND3, FGF8, CCND2, FGF9, TNN, CREB3L1, CREB3L2, MYB, AKT3, KDR, AKT1, TNR, IL6R, MAGI1, PDGFRB, IFNAR2, PDGFRA, HGF, MAGI2, TSC2, PRKCA, TSC1, EREG, CCNE2, COL4A3, COL4A5, RAF1, IFNAR1, CSF1R, PDGFB, LPAR1, LPAR3, PIK3R1, EFNA5, FOXO3, PIK3R6, HSP90B1, PIK3R5, BCL2L11, PDGFD, PDGFC, FGF23, NGFR, BDNF, INSR, FN1, IGF1, GNG12, NFKB1, IL4, IL6, CDK6, RHEB, ITGA11, GNB1, FGF18, BCL2, COL9A1, GNB4, GNB3, GRB2, GNB5, FGFR4, FGFR2, BCL2L1, FGF10, GSK3B, CDKN1B, ITGB5, FLT3, LAMC3, ITGB4, PTEN, PIK3CD, FASLG, LAMC2, BRCA1, LAMC1, PIK3CB, CASP9, GHR, IKBKB, PPP2R5E, ITGB8, ITGAV, ITGB6, JAK2, JAK1, ITGA4, SYK, VWF, PDPK1, ITGA3, ITGA2, ITGA1, PPP2R5A, PPP2R5C, NGF, YWHAZ, RBL2, COL2A1, CREB1, COL6A2, KIT, ITGA8, ITGA6, COL6A6, SOS1, SOS2, SGK2, MET, TLR2, ITGA9, CREB5, PHLPP2, PRKAA2, PHLPP1, LAMA2, PKN3, LAMA4, LAMA3, IL2RG, THBS1, EGFR, PPP2CA, RELN, ERBB4, PPP2R3C, NTF3, MAPK1, NTRK2, LAMB3, ANGPT1, VEGFC, LAMB1, PPP2R3A, MTOR, KITLG, RPS6KB1, PPP2R2B, IL2RB, PKN2, KRAS, TEK, PIK3AP1* |
| **brown** | oas05200:Pathways in cancer | 230 | 3.65 | 0.00 | *RB1, CALML4, EDNRA, RPS6KA5, TFG, RASSF5, AKT3, AKT1, PRKACB, DAPK1, PRKCB, DAPK2, PRKCA, RUNX1, EML4, ADCY9, CTBP2, PDGFB, LPAR1, MGST2, LPAR3, PIK3R1, PLCG2, PLCG1, RALGDS, FZD1, FZD3, JAG1, FZD5, FZD6, FN1, BRAF, IGF1, GNG12, GRB2, CDKN1B, BRCA2, GLI3, GLI2, IKBKB, SUFU, HEY2, RAC2, JAK2, JAK1, APPL1, APC2, NCOA1, IL15, NCOA3, WNT9A, DDB2, PLCB4, KIT, RARA, RARB, PLCB1, PLCB2, PPARD, PTGER4, CXCR4, ADCY3, ADCY2, ADCY8, EGFR, GNAI2, STAT4, CTNNA1, STAT6, CTNNA3, BID, RXRG, EGLN1, NQO1, ARNT2, STAT1, STAT3, WNT7A, VEGFC, MLH1, KITLG, CCDC6, CTNNB1, FAS, F2RL3, ITGA2B, ETS1, FGF2, IGF1R, CCND3, FGF8, CCND2, FGF9, EP300, IL6R, IL13RA1, PDGFRB, IFNAR2, PDGFRA, RALBP1, HGF, WNT5A, MITF, MSH6, CCNE2, SMO, MSH2, MSH3, COL4A3, COL4A5, RAF1, IL6ST, IFNAR1, NOTCH2, CSF1R, NOTCH1, MAX, CUL2, CUL1, PLD1, RASGRP1, FOXO1, KNG1, HSP90B1, BCL2L11, TPR, DVL1, ABL1, DVL3, WNT2, FGF23, WNT3, WNT4, SMAD2, ZBTB17, SMAD4, TGFB2, SMAD3, WNT3A, ZBTB16, PTCH1, ESR1, NFKB1, ESR2, BMP4, IL4, IL6, CXCL12, CDK6, GNB1, FGF18, BCL2, GNB4, GNB3, GNB5, FGFR4, FGFR2, BCL2L1, FGF10, NFE2L2, ALK, GSK3B, WNT2B, FLT3, LAMC3, PTEN, PIK3CD, FASLG, LAMC2, LAMC1, PIK3CB, CASP9, CASP7, CASP8, CASP3, ITGAV, POLK, ARHGEF12, APAF1, ITGA3, ITGA2, PLEKHG5, AXIN1, ARNT, TRAF1, AXIN2, TGFBR1, TGFBR2, TRAF3, TRAF6, TRAF5, ITGA6, SOS1, SOS2, MET, BIRC2, RALA, CAMK2D, RALB, ROCK1, LAMA2, ROCK2, LAMA4, LEF1, LAMA3, LRP5, CBL, IL2RG, DLL1, LRP6, GNA13, MAPK9, MAPK8, GNA12, MAPK1, E2F3, CAMK2G, TCF7L2, TCF7L1, LAMB3, TXNRD3, NOS2, TXNRD2, LAMB1, PML, MTOR, APC, RPS6KB1, IL2RB, KRAS* |
| **cyan** | oas00140:Steroid hormone biosynthesis | 3 | 0.96 | 0.03 | *SRD5A2, AKR1D1, HSD17B7* |
| **cyan** | oas04080:Neuroactive ligand-receptor interaction | 7 | 2.25 | 0.00 | *POMC, PAQR9, MAS1, GAL, CCKBR, NTS, SSTR5* |
| **grey** | oas00020:Citrate cycle (TCA cycle) | 5 | 0.53 | 0.04 | *FH, PDHA1, IDH1, OGDHL, ACO2* |
| **grey** | oas05033:Nicotine addiction | 6 | 0.63 | 0.02 | *GABRR3, SLC32A1, CHRNA4, CHRNA6, GABRA3, SLC17A6* |
| **grey** | oas03050:Proteasome | 7 | 0.74 | 0.01 | *PSMB4, PSMD7, IFNG, PSMD4, PSMC3, PSMB3, PSMC2* |
| **grey** | oas04975:Fat digestion and absorption | 7 | 0.74 | 0.01 | *FABP1, PLA2G12A, CLPS, PLA2G1B, GOT2, AGPAT2, SLC27A4* |
| **grey** | oas01230:Biosynthesis of amino acids | 8 | 0.84 | 0.02 | *TPI1, PSAT1, IDH1, GOT2, MAT1A, ALDOB, ACO2, OTC* |
| **grey** | oas04721:Synaptic vesicle cycle | 9 | 0.95 | 0.01 | *SLC6A5, ATP6V1A, SLC32A1, SLC17A6, SLC6A11, ATP6V1E1, CPLX1, ATP6V0C, SLC18A3* |
| **grey** | oas03008:Ribosome biogenesis in eukaryotes | 10 | 1.05 | 0.01 | *NOP56, POP5, TBL3, EMG1, DKC1, GAR1, FCF1, SNU13, REXO2, RPP25* |
| **grey** | oas04725:Cholinergic synapse | 10 | 1.05 | 0.02 | *KCNJ4, SLC5A7, CHRM3, KCNJ6, GNG10, CHRNA4, KCNQ2, CHRNA6, CHAT, SLC18A3* |
| **grey** | oas00564:Glycerophospholipid metabolism | 11 | 1.16 | 0.00 | *PLA2G15, CDS1, AGPAT5, PLA2G12A, PLA2G1B, LPCAT3, DGKQ, CHAT, LCAT, GPAT2, AGPAT2* |
| **grey** | oas01200:Carbon metabolism | 12 | 1.27 | 0.01 | *FH, PDHA1, TPI1, ECHS1, PSAT1, IDH1, GOT2, HAO1, OGDHL, ALDOB, ACO2, AGXT* |
| **grey** | oas04080:Neuroactive ligand-receptor interaction | 35 | 3.69 | 0.00 | *CHRM3, PTH, CHRNA4, PTGER2, CHRNA6, ADRB1, TRH, MLN, RXFP3, GABRR3, GRM4, NPVF, GALR1, P2RY2, PENK, DRD1, TAC1, DRD2, QRFPR, NTSR1, DRD5, UTS2R, GHSR, HTR1E, GABRA3, HTR1D, OPRK1, F2, HTR5A, AGT, ADRA2A, P2RX6, MTNR1A, MTNR1B, NMU* |
| **grey** | oas01100:Metabolic pathways | 84 | 8.86 | 0.00 | *DPAGT1, NDUFA11, MPI, NUDT2, MSMO1, OGDHL, FTCD, CNDP1, CNDP2, GYS2, FPGT, PIP5KL1, RFK, CA4, LIPG, HMGCS2, ENPP3, ATP6V1E1, GYG1, LBR, GAMT, CHST8, PLA2G12A, TPI1, ELOVL2, OXSM, ACOD1, NME3, ALG12, MAT1A, MIF, BPGM, KMO, UGDH, ALDH1A3, MINPP1, DCT, DGKQ, CMPK2, ALDOB, AGXT, ATP6V0C, CDS1, ATP6V1A, AGPAT5, NDUFB9, TPH1, FH, ECHS1, PLA2G1B, MGST3, HSD17B3, AGPAT2, NEU2, HSD11B2, NEU4, SI, HSD17B1, LDHD, INPP5K, PCBD1, HAO1, GPAT2, MGAT2, XDH, NADK, DUT, PDHA1, GSS, IDH1, GOT2, B3GALT6, PRDX6, RPE65, UCK1, GNPDA1, ADI1, PSAT1, ACO2, ALDH8A1, PLCD3, GLA, PLCD1, OTC* |
| **lightcyan** | oas05412:Arrhythmogenic right ventricular cardiomyopathy | 3 | 2.08 | 0.03 | *ACTN2, CACNG1, CACNA1S* |
| **lightcyan** | oas04260:Cardiac muscle contraction | 4 | 2.78 | 0.01 | *TNNC1, MYL3, CACNG1, CACNA1S* |
| **lightcyan** | oas04270:Vascular smooth muscle contraction | 4 | 2.78 | 0.01 | *MYLK2, EDN3, CACNA1S, MYLK4* |
| **lightcyan** | oas04921:Oxytocin signaling pathway | 4 | 2.78 | 0.01 | *MYLK2, CACNG1, CACNA1S, MYLK4* |
| **lightcyan** | oas04261:Adrenergic signaling in cardiomyocytes | 4 | 2.78 | 0.01 | *TNNC1, MYL3, CACNG1, CACNA1S* |
| **lightcyan** | oas04024:cAMP signaling pathway | 4 | 2.78 | 0.04 | *EDN3, NPY, CRH, CACNA1S* |
| **lightcyan** | oas05410:Hypertrophic cardiomyopathy | 5 | 3.47 | 0.00 | *TNNC1, MYL3, CACNG1, CACNA1S, TTN* |
| **lightcyan** | oas05414:Dilated cardiomyopathy | 5 | 3.47 | 0.00 | *TNNC1, MYL3, CACNG1, CACNA1S, TTN* |
| **lightcyan** | oas04814:Motor proteins | 5 | 3.47 | 0.00 | *MYL1, TNNC1, MYL3, TNNC2, TNNI1* |
| **lightcyan** | oas04020:Calciμm signaling pathway | 5 | 3.47 | 0.01 | *MYLK2, TNNC1, TNNC2, CACNA1S, MYLK4* |
| **lightcyan** | oas04080:Neuroactive ligand-receptor interaction | 6 | 4.17 | 0.01 | *CHRNA1, CHRNG, GABRA6, EDN3, NPY, CRH* |
| **purple** | oas03430:Mismatch repair | 3 | 1.18 | 0.04 | *RFC5, RFC4, RFC2* |
| **purple** | oas04964:Proximal tubule bicarbonate reclamation | 3 | 1.18 | 0.04 | *SLC25A10, ATP1A1, SLC38A3* |
| **purple** | oas04966:Collecting duct acid secretion | 4 | 1.57 | 0.01 | *ATP6V1G1, ATP6V1B2, ATP6V1G3, ATP6V1D* |
| **purple** | oas03030:DNA replication | 4 | 1.57 | 0.01 | *RFC5, FEN1, RFC4, RFC2* |
| **purple** | oas03410:Base excision repair | 4 | 1.57 | 0.03 | *RFC5, FEN1, RFC4, RFC2* |
| **purple** | oas00260:Glycine, serine and threonine metabolism | 4 | 1.57 | 0.03 | *DMGDH, ALAS1, CTH, GCAT* |
| **purple** | oas00600:Sphingolipid metabolism | 5 | 1.96 | 0.01 | *SMPD2, NEU3, SPHK1, HEXA, CERS2* |
| **purple** | oas04071:Sphingolipid signaling pathway | 6 | 2.35 | 0.03 | *SMPD2, SPHK1, SPTSSA, HRAS, MAPK13, CERS2* |
| **purple** | oas04140:Autophagy - animal | 7 | 2.75 | 0.02 | *MAP1LC3A, TP53INP2, RAB39B, NRBF2, EIF2S1, HRAS, CTSB* |
| **purple** | oas05017:Spinocerebellar ataxia | 7 | 2.75 | 0.02 | *PSMC1, PPIF, NRBF2, SLC25A5, GRIN2C, SLC25A4, GRM1* |
| **purple** | oas04150:mTOR signaling pathway | 7 | 2.75 | 0.02 | *ATP6V1G1, STRADB, ATP6V1B2, WNT8A, ATP6V1G3, HRAS, ATP6V1D* |
| **purple** | oas05022:Pathways of neurodegeneration - multiple diseases | 14 | 5.49 | 0.03 | *MAP2K3, WNT8A, RAB39B, GRIN2C, EIF2S1, GRM1, MAPK13, MAP1LC3A, PSMC1, PPIF, NRBF2, SLC25A5, HRAS, SLC25A4* |
| **purple** | oas01100:Metabolic pathways | 32 | 12.55 | 0.03 | *DMGDH, ALAS1, TAT, HEXA, ATP12A, PIGY, AK9, SMPD2, NEU3, HYAL1, ME1, HMOX1, ST8SIA5, PGM2, ASL, ATP6V1G3, HADH, ATP6V1D, OLAH, ATP6V1G1, FAHD1, SMOX, SPHK1, CTPS1, ASS1, GCAT, NMNAT1, CTH, ATP6V1B2, LAP3, HPD, CERS2* |
| **tan** | oas03060:Protein export | 6 | 0.27 | 0.04 | *SRP19, SRP72, SRPRA, SEC61B, SRP9, SEC11C* |
| **tan** | oas00785:Lipoic acid metabolism | 8 | 0.36 | 0.00 | *GCSH, LIPT2, LIPT1, AMT, PDHB, DLAT, LIAS, DLD* |
| **tan** | oas00630:Glyoxylate and dicarboxylate metabolism | 8 | 0.36 | 0.02 | *GCSH, MDH1, AMT, HYI, GLUL, HAO2, DLD, ACAT2* |
| **tan** | oas00020:Citrate cycle (TCA cycle) | 8 | 0.36 | 0.02 | *MDH1, SUCLG1, PDHB, DLAT, SDHD, SDHA, DLD, IDH3A* |
| **tan** | oas00250:Alanine, aspartate and glutamate metabolism | 9 | 0.40 | 0.02 | *ADSL, CPS1, GOT1, PPAT, GAD1, GFPT2, GAD2, GLUL, DDO* |
| **tan** | oas00513:Various types of N-glycan biosynthesis | 9 | 0.40 | 0.03 | *MGAT4D, RPN2, DAD1, OSTC, MGAT4B, RPN1, STT3A, ALG3, DDOST* |
| **tan** | oas00620:Pyruvate metabolism | 10 | 0.45 | 0.02 | *LDHB, PKM, MDH1, GLO1, PDHB, DLAT, HAGH, DLD, ACAT2, ALDH9A1* |
| **tan** | oas00240:Pyrimidine metabolism | 10 | 0.45 | 0.04 | *NT5C3A, DTYMK, RRM2B, NME6, ENTPD5, CMPK1, CTPS2, NT5M, UPP1, DHODH* |
| **tan** | oas04216:Ferroptosis | 10 | 0.45 | 0.05 | *MAP1LC3B, GPX4, FTH1, NCOA4, ACSL6, VDAC3, VDAC2, GCLM, SAT1, CP* |
| **tan** | oas03050:Proteasome | 11 | 0.49 | 0.01 | *PSMD12, PSMC5, PSMA4, PSMA1, PSMB2, PSMD13, PSMA2, PSME3, ADRM1, PSMB1, PSMD3* |
| **tan** | oas00280:Valine, leucine and isoleucine degradation | 11 | 0.49 | 0.02 | *ALDH6A1, ACAA2, HMGCS1, EHHADH, ACAA1, BCAT1, HMGCLL1, DLD, ACADSB, ACAT2, ALDH9A1* |
| **tan** | oas04979:Cholesterol metabolism | 13 | 0.58 | 0.00 | *LPL, APOA1, APOC3, ABCB11, LIPA, NCEH1, NPC2, APOH, VDAC3, VDAC2, TSPO, VDAC1, ANGPTL4* |
| **tan** | oas00510:N-Glycan biosynthesis | 13 | 0.58 | 0.00 | *RPN2, RPN1, ALG5, ALG3, DOLK, DDOST, DPM2, MGAT4D, DAD1, OSTC, MGAT4B, MGAT3, STT3A* |
| **tan** | oas01230:Biosynthesis of amino acids | 14 | 0.62 | 0.01 | *GOT1, PYCR1, ENO1, MAT2B, SDSL, RPIA, PKM, CPS1, PAH, ALDOC, BCAT1, GLUL, GAPDH, IDH3A* |
| **tan** | oas03320:PPAR signaling pathway | 16 | 0.71 | 0.00 | *HMGCS1, GK, ADIPOQ, ACSL6, ILK, LPL, NR1H3, APOA1, APOC3, DBI, ACADL, EHHADH, FABP7, UBC, ANGPTL4, ACAA1* |
| **tan** | oas01232:Nucleotide metabolism | 16 | 0.71 | 0.00 | *DTYMK, ADSL, ENTPD2, GDA, ENTPD5, AK2, AMPD1, CTPS2, NT5C3A, NME6, RRM2B, CMPK1, NT5M, HPRT1, UPP1, ADA* |
| **tan** | oas04068:FoxO signaling pathway | 18 | 0.80 | 0.05 | *PRKAA1, MAP2K1, GABARAPL1, CDKN1A, TGFB3, IRS4, FOXO6, FOXO4, SOD2, PRKAB1, KLF2, ATG12, GADD45G, CCNB2, G6PC2, CCNG2, S1PR1, IL7R* |
| **tan** | oas04146:Peroxisome | 19 | 0.85 | 0.00 | *PEX16, GSTK1, ABCD2, ACSL6, HSD17B4, SOD2, DDO, SOD1, AMACR, PRDX1, EHHADH, PEX5, PXMP4, MLYCD, PEX11G, ACAA1, DECR2, HMGCLL1, HAO2* |
| **tan** | oas01200:Carbon metabolism | 23 | 1.02 | 0.00 | *MDH1, GOT1, AMT, ENO1, PDHB, SDHD, SDHA, SDSL, ACAT2, GCSH, RPIA, ALDH6A1, PKM, CPS1, ESD, ALDOC, SUCLG1, DLAT, PGLS, DLD, HAO2, GAPDH, IDH3A* |
| **tan** | oas04142:Lysosome | 23 | 1.02 | 0.00 | *ARSA, ASAH1, SLC11A1, CTSZ, FUCA2, LAPTM5, M6PR, NAGA, GNS, LIPA, CLN5, AP3M2, GNPTG, LAPTM4A, LAMP1, NPC2, CTSK, LAMP3, HYAL2, SMPD1, TPP1, GUSB, LGMN* |
| **tan** | oas01240:Biosynthesis of cofactors | 24 | 1.07 | 0.01 | *LIPT2, LIPT1, ADSL, AK2, CTPS2, MAT2B, LIAS, COQ7, PPCDC, PTS, COASY, DHODH, COQ5, GMPPB, NFS1, PHOSPHO2, NME6, UROD, HMBS, CMPK1, GUSB, GCLM, BCAT1, DLD* |
| **tan** | oas00190:Oxidative phosphorylation | 31 | 1.38 | 0.00 | *NDUFB8, NDUFA13, NDUFB6, NDUFB10, NDUFB5, NDUFA12, NDUFB4, NDUFA10, NDUFB3, NDUFB2, NDUFB1, NDUFV3, NDUFV2, NDUFV1, NDUFA9, NDUFA8, NDUFA7, NDUFA6, NDUFA5, NDUFA4, NDUFA2, NDUFA1, NDUFC1, SDHD, SDHA, NDUFS8, NDUFS7, NDUFS5, NDUFAB1, NDUFS3, NDUFS1* |
| **tan** | oas04723:Retrograde endocannabinoid signaling | 35 | 1.56 | 0.00 | *GABRB3, NDUFB8, NDUFA13, NDUFB6, NDUFB10, NDUFB5, NDUFA12, NDUFB4, NDUFA10, NDUFB3, GNAI3, NDUFB2, NDUFB1, ABHD6, GNG5, CNR1, NDUFV3, NDUFV2, NDUFV1, NDUFA9, NDUFA8, NDUFA7, NDUFA6, NDUFA5, NDUFA4, NDUFA2, NDUFA1, NDUFC1, GNG13, NDUFS8, NDUFS7, NDUFS5, NDUFAB1, NDUFS3, NDUFS1* |
| **tan** | oas04714:Thermogenesis | 40 | 1.78 | 0.00 | *PRKAA1, NDUFB8, NDUFA13, NDUFB6, NDUFB10, NDUFB5, NDUFA12, NDUFB4, NDUFA10, NDUFB3, NDUFB2, NDUFB1, ACTB, CNR1, NDUFV3, NDUFV2, NDUFV1, NDUFA9, NDUFA8, NDUFA7, NDUFA6, NDUFA5, NDUFA4, RPS6, NDUFA2, NDUFA1, ACSL6, NDUFC1, GCG, SDHD, SDHA, PRKAB1, NDUFS8, NDUFS7, ADRB3, NDUFS5, NDUFAB1, NDUFS3, NDUFS1, PNPLA2* |
| **tan** | oas04932:Non-alcoholic fatty liver disease | 41 | 1.83 | 0.00 | *PRKAA1, NDUFB8, NDUFA13, NDUFB6, NDUFB10, NDUFB5, NDUFA12, NDUFB4, NDUFA10, NDUFB3, NDUFB2, NDUFB1, ADIPOR1, ADIPOR2, SOCS3, NDUFV3, NDUFV2, NDUFV1, NDUFA9, NDUFA8, NDUFA7, NDUFA6, NDUFA5, NDUFA4, NDUFA2, ADIPOQ, NDUFA1, NR1H3, NDUFC1, FOS, SDHD, SDHA, PRKAB1, NDUFS8, NDUFS7, NDUFS5, DDIT3, NDUFAB1, NDUFS3, NDUFS1, ATF4* |
| **tan** | oas05208:Chemical carcinogenesis - reactive oxygen species | 42 | 1.87 | 0.00 | *NDUFB8, NDUFA13, NDUFB6, NDUFB10, NDUFB5, NDUFA12, NDUFB4, NDUFA10, NDUFB3, MGST1, NDUFB2, NDUFB1, NDUFV3, NDUFV2, ACP1, NDUFV1, NDUFA9, NDUFA8, NDUFA7, MAP2K1, NDUFA6, NDUFA5, NDUFA4, NDUFA2, EPHX4, NDUFA1, NDUFC1, FOS, SDHD, SOD2, SDHA, SOD1, NFKBIA, NDUFS8, NDUFS7, NDUFS5, NDUFAB1, VDAC3, NDUFS3, VDAC2, NDUFS1, VDAC1* |
| **tan** | oas05415:Diabetic cardiomyopathy | 43 | 1.91 | 0.00 | *NDUFB8, NDUFA13, NDUFB6, NDUFB10, NDUFB5, NDUFA12, NDUFB4, NDUFA10, NDUFB3, NDUFB2, NDUFB1, PDHB, MPC1, MPC2, PDK4, NDUFV3, NDUFV2, NDUFV1, PDK2, NDUFA9, NDUFA8, NDUFA7, NDUFA6, NDUFA5, TGFB3, NDUFA4, GFPT2, NDUFA2, NDUFA1, NDUFC1, SDHD, SDHA, NDUFS8, NDUFS7, NDUFS5, NDUFAB1, VDAC3, NDUFS3, AGTR1, VDAC2, NDUFS1, VDAC1, GAPDH* |
| **tan** | oas05020:Prion disease | 51 | 2.27 | 0.00 | *NDUFA13, NDUFA12, NDUFA10, PSMD3, NDUFC1, SDHD, SDHA, PSMA4, NDUFS8, NDUFS7, PSMA1, PSMA2, NDUFS5, DDIT3, VDAC3, NDUFS3, VDAC2, NDUFS1, VDAC1, ATF4, PSMD12, NDUFB8, NDUFB6, NDUFB10, NDUFB5, PSMD13, NDUFB4, NDUFB3, NDUFB2, NDUFB1, KLC4, PSMB2, C9, PSMB1, NDUFV3, NDUFV2, NDUFV1, NDUFA9, NDUFA8, NDUFA7, NDUFA6, NDUFA5, CAV2, NDUFA4, ADRM1, NDUFA2, NDUFA1, HSPA2, SOD1, PSMC5, NDUFAB1* |
| **tan** | oas05171:Coronavirus disease - COVID-19 | 54 | 2.40 | 0.01 | *RPL5, RPL30, RPL3, RPL32, RPL31, RPLP1, RPL8, RPL10A, RPL9, RPL7, RPS14, RPS17, RPS16, RPL18A, RPL35, IL12B, RPS11, RPL39, RPS10, RPS13, RPS12, RPS7, RPL21, RPL23, FGG, RPS6, RPL22, RPS3A, RPSA, FOS, RPL37A, AGTR1, RPL24, RPL12, RPS15A, C9, RPS3, RPL14, RPL15, RPS27A, RPL19, RPL35A, RPL23A, NFKBIA, RPS25, RPS29, RPL27A, RPL22L1, RPS20, RSL24D1, RPS21, RPL26L1, RPS24, RPS23* |
| **tan** | oas05012:Parkinson disease | 60 | 2.67 | 0.00 | *NDUFA13, NDUFA12, NDUFA10, PARK7, TXN2, UCHL1, PSMD3, DUSP1, SLC39A13, NDUFC1, SDHD, SDHA, PSMA4, NDUFS8, NDUFS7, ADORA2A, PSMA1, PSMA2, NDUFS5, DDIT3, VDAC3, NDUFS3, VDAC2, NDUFS1, VDAC1, ATF4, PSMD12, NDUFB8, NDUFB6, NDUFB10, NDUFB5, PSMD13, NDUFB4, NDUFB3, GNAI3, NDUFB2, NDUFB1, TXN, KLC4, PSMB2, UBC, PSMB1, NDUFV3, NDUFV2, RPS27A, NDUFV1, NDUFA9, NDUFA8, NDUFA7, NDUFA6, NDUFA5, NDUFA4, ADRM1, NDUFA2, NDUFA1, SOD1, PINK1, PSMC5, NDUFAB1, CALM2* |
| **tan** | oas04080:Neuroactive ligand-receptor interaction | 61 | 2.72 | 0.00 | *GABRB3, OXTR, VIPR2, CHRM4, PMCH, MLNR, NPY2R, CHRM5, HTR2C, ADM, ADRA1B, QRFP, HTR6, BDKRB2, ADORA1, PRLHR, TSPO, TSHB, INSL5, PTGDR, CHRNB2, EDN2, AVPR1B, SSTR1, OPRM1, ADRA2B, ADCYAP1, MC3R, UTS2B, ADORA2A, ADRB3, AGTR1, AGTR2, PTGER3, CHRNA9, LPAR2, CRHR2, NPS, P2RY6, APELA, CALCR, HRH3, TBXA2R, CNR1, NPBWR2, S1PR1, S1PR3, NMB, HTR1F, GCGR, SCTR, HTR1A, GPR50, GCG, TRPV1, NMBR, HCRTR2, SST, P2RX1, NMUR1, VIP* |
| **tan** | oas03010:Ribosome | 64 | 2.85 | 0.00 | *RPL5, MRPS15, RPL30, RPL3, MRPS16, RPL32, RPL31, RPLP1, MRPS11, MRPS10, RPL8, RPL10A, MRPL35, RPL9, MRPL32, RPL7, MRPL33, RPS14, RPS17, MRPL2, RPS16, RPL18A, RPL35, RPS11, RPL39, RPS10, RPS13, RPS12, RPS7, RPL21, RPL23, RPS6, RPL22, MRPS21, MRPS2, RPS3A, RPSA, RPL37A, RPL24, RPL12, MRPL18, MRPL17, MRPL12, MRPL20, RPS15A, RPS3, RPL14, RPL15, RPS27A, RPL19, MRPL28, RPL35A, RPL23A, MRPL23, RPS25, RPS29, RPL27A, RPL22L1, RPS20, RSL24D1, RPS21, RPL26L1, RPS24, RPS23* |
| **tan** | oas05014:Amyotrophic lateral sclerosis | 64 | 2.85 | 0.00 | *NDUFA13, NDUFA12, NDUFA10, NXT2, ACTB, MAP1LC3B, PSMD3, GPX1, GPX3, NDUFC1, SDHD, SDHA, PSMA4, NDUFS8, NDUFS7, PSMA1, PSMA2, NDUFS5, DDIT3, NDUFS3, NDUFS1, VDAC1, NUP62CL, SQSTM1, PFN2, ATF4, DCTN6, RAB1A, BECN1, DCTN5, PSMD12, NDUFB8, NDUFB6, NDUFB10, NDUFB5, PSMD13, NDUFB4, DCTN3, NDUFB3, SLC1A2, NDUFB2, NDUFB1, KLC4, PSMB2, ATG101, PSMB1, NDUFV3, NDUFV2, DNAI1, NDUFV1, NDUFA9, NDUFA8, NDUFA7, NDUFA6, NDUFA5, NDUFA4, ADRM1, NDUFA2, NDUFA1, CHCHD10, SOD1, PINK1, PSMC5, NDUFAB1* |
| **tan** | oas05010:Alzheimer disease | 64 | 2.85 | 0.00 | *NDUFA13, NDUFA12, NDUFA10, CHRM5, IRS4, PSMD3, MAP2K1, WNT5B, SLC39A13, NDUFC1, SDHD, SDHA, BACE1, PSMA4, NDUFS8, NDUFS7, PSMA1, PSMA2, NDUFS5, DDIT3, VDAC3, NDUFS3, VDAC2, NDUFS1, VDAC1, GAPDH, PPID, ATF4, BECN1, PSMD12, NDUFB8, NDUFB6, NDUFB10, NDUFB5, PSMD13, NDUFB4, NDUFB3, PSEN2, NDUFB2, WNT8B, LPL, NDUFB1, RTN4, KLC4, PSMB2, ATG101, PSMB1, NDUFV3, NDUFV2, NDUFV1, NDUFA9, NDUFA8, NDUFA7, NDUFA6, NDUFA5, NDUFA4, FZD4, ADRM1, NDUFA2, NDUFA1, FZD9, PSMC5, NDUFAB1, CALM2* |
| **tan** | oas05016:Huntington disease | 66 | 2.94 | 0.00 | *NDUFA13, NDUFA12, NDUFA10, PSMD3, AP2M1, TGM2, GPX1, GPX3, NDUFC1, SDHD, SDHA, PSMA4, NDUFS8, NDUFS7, PSMA1, PSMA2, NDUFS5, VDAC3, NDUFS3, VDAC2, TFAM, NDUFS1, VDAC1, STX1A, DCTN6, BECN1, DCTN5, PSMD12, NDUFB8, HDAC2, NDUFB6, NDUFB10, NDUFB5, PSMD13, NDUFB4, DCTN3, NDUFB3, SLC1A2, NDUFB2, NDUFB1, KLC4, PSMB2, ATG101, POLR2B, POLR2D, PSMB1, AP2S1, NDUFV3, NDUFV2, DNAI1, NDUFV1, POLR2J, POLR2K, NDUFA9, NDUFA8, NDUFA7, NDUFA6, NDUFA5, NDUFA4, ADRM1, NDUFA2, NDUFA1, SOD2, SOD1, PSMC5, NDUFAB1* |
| **tan** | oas05022:Pathways of neurodegeneration - multiple diseases | 75 | 3.34 | 0.00 | *NDUFA13, NDUFA12, NDUFA10, CHRM5, PARK7, MAP1LC3B, UCHL1, PSMD3, MAP2K1, GPX1, WNT5B, TRPC3, GPX3, NDUFC1, SDHD, SDHA, PSMA4, NDUFS8, NDUFS7, PSMA1, PSMA2, NDUFS5, DDIT3, VDAC3, NDUFS3, VDAC2, NDUFS1, VDAC1, SQSTM1, PPID, STX1A, ATF4, DCTN6, RAB1A, BECN1, DCTN5, PSMD12, NDUFB8, NDUFB6, NDUFB10, NDUFB5, PSMD13, NDUFB4, DCTN3, NDUFB3, PSEN2, NDUFB2, WNT8B, NDUFB1, KLC4, PSMB2, ATG101, UBC, PSMB1, NDUFV3, NDUFV2, RPS27A, DNAI1, NDUFV1, NDUFA9, NDUFA8, NDUFA7, NDUFA6, NDUFA5, NDUFA4, FZD4, ADRM1, NDUFA2, NDUFA1, FZD9, SOD1, PINK1, PSMC5, NDUFAB1, CALM2* |
| **tan** | oas01100:Metabolic pathways | 190 | 8.46 | 0.00 | *NDUFA13, ACAA2, GALNT15, NDUFA12, GDA, NDUFA10, ENO1, SAT1, GCSH, UROD, PPAT, NAMPT, MLYCD, ACAA1, ENPP6, GLUL, GSTK1, ARSA, ENTPD2, ENTPD5, ALG5, ACSL6, ALG3, SDHD, CEL, SDHA, DPM2, PHOSPHO1, PHOSPHO2, HMBS, CMPK1, SUCLG1, HPRT1, PLPP2, PTGES, LIPT2, LIPT1, NDUFB10, RPN2, GLO1, RPN1, MGST1, AK2, PLA2G3, SDSL, LIAS, GNS, ACAT2, LDHB, PTDSS2, UGT8, FUT7, AANAT, AMDHD2, ESD, SMYD2, HYI, NDUFV3, NDUFV2, HAO2, NDUFV1, HMGCLL1, GGT5, GGT7, GOT1, SRD5A1, GFPT2, PYCR1, DOLK, ACADSB, COQ7, MCAT, CRLS1, COQ5, GGCT, ALDH6A1, G6PC2, AMACR, CPS1, PAH, P4HA3, STT3A, PEMT, DEGS1, GCLM, ADA, SUV39H2, PIGT, MOGAT1, TECR, PIK3CG, PPCDC, GMPPB, ACADL, CA5A, NMRK2, HYAL2, SMPD1, PGLS, CA8, DLAT, UPP1, GUSB, NANS, ACP1, GALNT9, IDH3A, ADSL, PCYT1B, GPX1, GPX4, HMGCS1, GPX3, MARS2, AMT, AMPD1, CTPS2, SIRT5, NDUFC1, COASY, DDOST, NT5C3A, PKM, NDUFS8, NME6, RRM2B, NDUFS7, DAD1, NDUFS5, EHHADH, NDUFS3, ALDOC, NDUFS1, PIGM, HAGH, BCAT1, DLD, GAPDH, GART, ST6GALNAC4, ALDH9A1, ST6GALNAC6, NDUFB8, DTYMK, ASAH1, NDUFB6, NDUFB5, NDUFB4, NDUFB3, NDUFB2, NDUFB1, HSD17B4, NPL, PDHB, MAT2B, GUCY2F, DDO, PTS, SRM, NFS1, EXTL2, MGAT3, NT5M, CKB, NDUFA9, NDUFA8, NDUFA7, NDUFA6, GK, NDUFA5, MDH1, PDE6H, PTGES2, NDUFA4, GAD1, NDUFA2, NDUFA1, GATC, GAD2, NAGA, ETNPPL, DHODH, RPIA, MGAT4D, MGAT4B, NDUFAB1, CHPT1, LPIN1, CDO1, PNPLA2* |
