# Supplementary material for: Transcriptome meta-analysis reveals the hair genetic rules in six animal breeds and genes associated with wool fineness
Source: Front Genet. 2024 Jun 14;15:1401369. doi: 10.3389/fgene.2024.1401369 (PMC11211574; doi:10.3389/fgene.2024.1401369)
Supplement: Supplementary file 1 [file DataSheet1.ZIP › attachments/Table S3.docx]

| Sample | Raw  Reads | Raw  Bases | Clean  Reads | Clean  Bases | Error  Rate | Q20 | Q30 | GC  Content |
| --- | --- | --- | --- | --- | --- | --- | --- | --- |
| N2 | 42314010 | 6.37G | 42113528 | 6.36G | 0.03% | 97.94% | 94.21% | 55.25% |
| N48 | 49166572 | 7.39G | 48849328 | 7.38G | 0.02% | 98.21% | 94.97% | 53.31% |
| N57 | 44364496 | 6.66G | 44017692 | 6.65G | 0.02% | 98.04% | 94.49% | 53.39% |
| N71 | 55376398 | 8.33G | 55040200 | 8.31G | 0.02% | 98.07% | 94.52% | 51.87% |
| N74 | 49016950 | 7.36G | 48631116 | 7.34G | 0.02% | 98.22% | 94.97% | 53.14% |
| N75 | 52302406 | 7.86G | 51966034 | 7.85G | 0.02% | 98.09% | 94.56% | 52.54% |
| N99 | 43743680 | 6.58G | 43519954 | 6.57G | 0.02% | 98.14% | 94.66% | 53.05% |
| N106 | 44007042 | 6.62G | 43770616 | 6.61G | 0.03% | 97.82% | 93.92% | 52.68% |
| N110 | 45593452 | 6.84G | 45252828 | 6.83G | 0.03% | 97.80% | 93.86% | 52.29% |
| N116 | 48010916 | 7.22G | 47732068 | 7.21G | 0.02% | 98.10% | 94.56% | 52.18% |
| N122 | 56871610 | 8.55G | 56532154 | 8.54G | 0.03% | 97.79% | 93.79% | 52.43% |
| N126 | 46401062 | 6.98G | 46123948 | 6.96G | 0.03% | 97.76% | 93.74% | 52.18% |
| N156 | 51316310 | 7.71G | 50978484 | 7.7G | 0.02% | 98.08% | 94.63% | 53.59% |
| N164 | 43343554 | 6.52G | 43119962 | 6.51G | 0.03% | 97.89% | 94.10% | 52.98% |
| N167 | 47091402 | 7.09 | 46842424 | 7.07 | 0.03 | 97.68 | 93.61 | 53.43 |
| N170 | 57951852 | 8.72 | 57644072 | 8.7 | 0.03 | 97.84 | 93.98 | 54.31 |
| N175 | 44586068 | 6.7 | 44283696 | 6.69 | 0.03 | 97.97 | 94.29 | 52.53 |
| N176 | 48829710 | 7.33 | 48472484 | 7.32 | 0.02 | 98.25 | 95.03 | 53.11 |
| N182 | 50251064 | 7.54 | 49823502 | 7.52 | 0.02 | 98.03 | 94.51 | 53.77 |
| N183 | 46660490 | 7.02 | 46394358 | 7.01 | 0.02 | 98.08 | 94.57 | 53.71 |

Table S3.Skin quality control reports for 20 samples.
