# Supplementary material for: Transcriptome meta-analysis reveals the hair genetic rules in six animal breeds and genes associated with wool fineness
Source: Front Genet. 2024 Jun 14;15:1401369. doi: 10.3389/fgene.2024.1401369 (PMC11211574; doi:10.3389/fgene.2024.1401369)
Supplement: Supplementary file 1 [file DataSheet1.ZIP › attachments/Table S4.docx]

| Gene Name | Sequence（5’- 3’） | ID |
| --- | --- | --- |
| *LOC101102194* | F:AGGGACAGAACCGACCTCAG R:TCCTCATCAGCATTATAACTCATGG | XM_004020283.5 |
| *MX2* | F:GAGATTCGCAGAGCCCAGAA R:CGGGAAGGTCAATGAGGGTC | NM_001078652.1 |
| *LOC101117112* | F:TGAAAGGCTCCTTCCTGCTC R:AGCAGCCTTGGCTCCTACT | XM_004017967.4 |
| *LOC105614852* | F:GAAGTTGCCCACGGAAAACC R:TTGCTTGTAGCTTGTCCCCT | XM_027971855.2 |
| *LOC114110058* | F:GGGGACATCCTCAAGGTTTTG R:AGTTCTTGGGGATGAAGCCG | XM_027959622.2 |
| *LOC101116863* | F:GAACATCTCTTTCCCGGCCA R:CCATTCTTCACCCAGAGCGT | XM_042233914.1 |
| *LOC105602911* | F:AGCACTGGTACCAACTGTGTAA R:ATGCTGTTTCCTTGCACCCT | XM_012097882.3 |
| *LOC101112122* | F:GGAAGGTGCCGGAACTGAA R:GACTTGGCCAGGGTATTTCT | XM_027956577.2 |
| *MX1* | F:GCGAGTGGAAAGGCAAAGTC R:CGATGGCAATCTGGGCTTCA | NM_001009753.1 |
| *IFI6* | F:CAAGCTACTTCACCTGCCCA R:AGGCTTTGTCACCGTATCATCA | XM_027965433.2 |
| *LOC114110852* | F:ATTGACACCTACAGCGACGG R:GATGGGGAACCGCACTAGG | XM_042241131.1 |
| *LOC114113348* | F:ACAGCAACTACTACGGTGGC R:GCGGTAGCAGCCATATCCAT | XM_027965699.2 |
| *HBB* | F:GAGAACTTCAGGCTCCTGGG R:CCAGCCACCACCTTCTGAAA | NM_001097648.1 |
| *SERPINB1* | F:GGCACAGCTTTCCAAGGTTC R:GTAGGGAGCACCATGCTTGT | NM_001280707.1 |
| *LOC114118559* | F:ACATTCGGTCCTTTCTATGGAATC R:GGACTTTCCAAAAACTTGGCTGA | XM_042233544.1 |
| *RSAD2* | F:CTGGTTCCAGAAGTACGGTGA R:GCCGATAAGGACGTTGACCT | XM_004005669.5 |
| *ITGB2* | F:CACAGACGATGGGTTCCACT R:CCCACCGATGGGTAGTCAAAT | NM_001009485.1 |
| *C1QTNF6* | F:CTGGGCCGGCAACAAGTTAT R:CATCCCCACGGCGACTCAG | XM_004006731.5 |
| *MYOC* | F:CAGGAGGTAGCAAGCCTGAG R:GAGTTCTCCGCATCCTTCCC | XM_027975610.2 |
| *BRSK2* | F:GAAGTCCATGGAGGTGCTGA R:TGATGGACCTGGACCTCTGG | XM_042237779.1 |
| *KAZALD1* | F:TTTGCCTATCCCATGGCCTC R:GTCCACCCCTAAACTGCACG | XM_027960353.2 |
| *FOS* | F:AGGGGCAAGGTAGAACAGTTG R:AGTTGGTCTGTCTCCGCTTG | NM_001166182.1 |
| *CCL24* | F:CAGACTCCGTGATACTCCCCT R:TCCGGGTGGTGAAGATCACTC | XM_027961615.2 |
| *ITGAM* | F:TCGTGACCCAGTGGTAAAAGG R:TGCTCTGGGTATCTCCTTCTC | NM_001082593.1 |
| *LOC101121216* | F:AGGATGAACCTTTCCACGGG R:TGTCTTTAGCCCCTTGACCA | XM_004019478.5 |
| *LOC114110633* | F:ATCTCTGTTCACAGCACACCT R:TGCTGAGTTCCTCCTCCATCT | XM_042239721.1 |
| *C1QC* | F:CTCCCACCTGTCCAGGAATC R:GGGACCGTTTTTCCCAGGAT | XM_042244594.1 |
| *LOC114112239* | F:ACTACCTGTGGAGGGCAGAT R:AGCGCGGGAAAAGCCTTAAT | XM_042242719.1 |
| *LOC101105154* | F:CTCTTCTGGCCACCCTGG R:ACAGCCTGGAATTCCCACTT | XM_042248800.1 |
| *LOC101118514* | F:GGAGCCAGAAGGGGCTTG R:ATTCTGGGCAAGAACAGGCA | XM_027959117.2 |
| *LOC101117229* | F:CTTTCTGTCCTGTGGAGCGT R:ATACAGGGCTTCATTGGCCC | XM_027961520.2 |
| *LOC114118432* | F:AAGGCACTGGGAGGACATTG R:GAGCACCATGGTTCCAAAGA | XM_042233545.1 |

Table S4.Primer information of 32 key genes
