# Supplementary material for: Transcriptome meta-analysis reveals the hair genetic rules in six animal breeds and genes associated with wool fineness
Source: Front Genet. 2024 Jun 14;15:1401369. doi: 10.3389/fgene.2024.1401369 (PMC11211574; doi:10.3389/fgene.2024.1401369)
Supplement: Supplementary file 1 [file DataSheet1.ZIP › attachments/Table S5.docx]

| Stage | cycle | temperature | time |
| --- | --- | --- | --- |
| predegeneration | 1X | 95℃ | 3min |
| PCR reaction | 40X | 95℃ | 5sec |
|  |  | 60℃ | 10sec |
|  |  | 72℃ | 15sec |
| Melting/Dissociation Curve Stage | | | |

**Table S5 qPCR reaction proced**
