# Supplementary figures and images for: Transcriptome meta-analysis reveals the hair genetic rules in six animal breeds and genes associated with wool fineness
Source: Front Genet. 2024 Jun 14;15:1401369. doi: 10.3389/fgene.2024.1401369 (PMC11211574; doi:10.3389/fgene.2024.1401369)

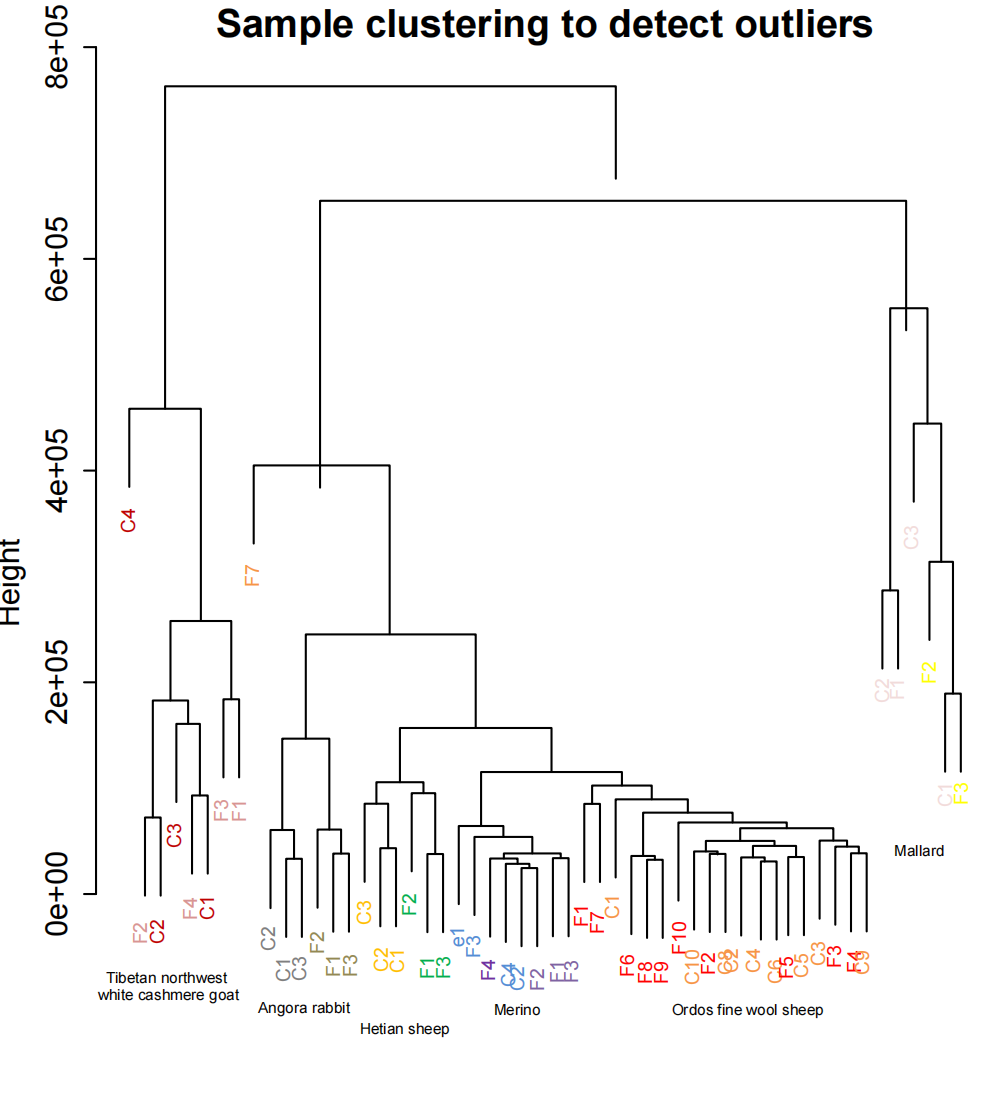


**Figure S2.Cluster tree of 54 RNA-Seq data**

Supplement: Supplementary file 1 [file DataSheet1.ZIP › attachments/Figure S2.docx]
